# The impacts of new antidiabetic drugs on the risk of ischemic and hemorrhagic strokes: a comprehensive review and meta-analysis of clinical trials

**DOI:** 10.3389/fstro.2024.1363954

**Published:** 2024-06-20

**Authors:** Hala F. Azhari, Jesse Dawson

**Affiliations:** ^1^College of Medicine and Pharmacy, Umm Al-Qura University, Makkah, Saudi Arabia; ^2^School of Cardiovascular and Metabolic Health, University of Glasgow, Glasgow, United Kingdom

**Keywords:** sodium-glucose cotransporter 2 inhibitors, glucagon-like peptide-1 receptor agonists, dipeptidyl peptidase-4 inhibitors, type 2 diabetes mellitus, stroke, randomized controlled trials

## Abstract

**Introduction:**

New classes of antidiabetic drugs reportedly lower the risk of cardiovascular events. This review summarizes the evidence for the effects of these drugs on the risk of stroke in diabetic individuals.

**Methods:**

Multiple databases that report stroke outcome data were scrutinized for clinical trials (from inception to June 25, 2023), compared sodium-glucose cotransporter 2 inhibitors (SGLT2-Is), glucagon-like peptide-1 receptor agonists (GLP1-RAs), and dipeptidyl peptidase-4 inhibitors (DPP4-Is), vs. other antidiabetic drugs and placebo.

**Results:**

Among the 960 identified trials, 259 satisfied the eligibility criteria. Among these, 177 and 82 trials reported at least one or no stroke events, respectively. In total, 208, 19, and 32 trials had a low, unclear, and high risk of bias, respectively. SGLT2-Is use did not decrease the risk of non-fatal hemorrhagic or ischemic stroke (risk ratio (RR) 0.96; 95% CI 0.87 to 1.06; *P* = 0.42) vs. either active comparators or placebo. GLP1-RAs use significantly decreased stroke risk (RR: 0.84, 95% CI [0.77, 0.93], *p* = 0.0005) and ischemic stroke (RR: 0.85, 95% CI [0.77, 0.94], *p* = 0.002) vs. placebo. However, GLP1-RAs use did not decrease hemorrhagic events vs. active comparators or placebo. DPP4-Is use did not decrease the risk of non-fatal hemorrhagic or ischemic stroke (RR: 0.91; 95% CI [0.83, 1.01], *p* = 0.07) vs. active comparators or placebo. For all classes, fatal stroke risk did not decrease vs. active comparators or placebo, and the Grading of Recommendations, Assessment, Development and Evaluation (GRADE) Working Group scores were moderate.

**Discussion:**

The use of GLP1-RAs, but not SGLT2-Is or DPP4-Is, may decrease non-fatal stroke risk. Considering these results, the findings may inform the treatment of diabetic people at risk of stroke and the design of new antidiabetic interventional trials.

**Systematic review registration:**

https://www.crd.york.ac.uk/prospero/display_record.php?ID=CRD42017067889, identifier 42017067889.

## 1 Introduction

Diabetes, as an independent risk factor for stroke (Sarwar et al., 2010), accounts for a more than 10% increase in the risk of early stroke recurrences (Appelros et al., 2009) worldwide. Furthermore, diabetes is associated with a two- to fourfold increase in major adverse cardiovascular events (MACEs; Meigs, 2003; Huxley et al., 2006). Therefore, European (European Stroke Organisation Executive Committee, 2008) and U.S. (Kleindorfer et al., 2021) guidelines recommend glycemic control for the prevention of primary (Goldstein et al., 2001) and secondary (Pearson et al., 2002) cardiovascular disease (CVD), suggesting an glycated hemoglobin A1c (HbA1c) < 7% to decrease the macro- and microvascular risks complications (Grundy et al., 2002). However, such aggressive management has led to concerns regarding MACE outcomes. Indeed, the concomitant use of multiple antidiabetic drugs, along with insulin, increases severe hypoglycemia risks that coexist with autonomic cardiovascular neuropathy and might increase the risk of sudden death (Braffett et al., 2020). The U.S. Food and Drug Administration (FDA/CDER, 2023) and the European Medicines Agency (European Medicines Agency, 2022), among other major world health regulatory agencies, have therefore stipulated that any antidiabetic drug should be assessed for their safety regarding cardiovascular risk.

Pioglitazone (Culman et al., 2007), from the thiazolidinedione class of antidiabetic, is reported to decrease the risk of first stroke, stroke recurrence, and MACEs in people with insulin resistance and diabetes (Lee et al., 2017). Clinical and experimental studies have also suggested that sodium-glucose cotransporter 2 inhibitors (SGLT2-Is; Tsai et al., 2021) and incretin-based drugs, including glucagon-like peptide-1 receptor agonists (GLP1-RAs) and dipeptidyl peptidase-4 inhibitors (DPP4-Is; Darsalia et al., 2015), may decrease the risk of stroke. Previous meta-analyses (Mahmouda et al., 2017; Saad et al., 2017) have explored the safety of these drugs regarding CVD risk but were limited by the trials' heterogeneity in terms of studied endpoints, follow-up periods, or population characteristics. Additionally, few stroke events were reported in many of the trials.

To assess the net clinical benefits of the new emerging antidiabetic drugs, herein we reviewed reports of clinical trials to assess stroke risk. We hypothesize that the use of these drugs would decrease stroke risk in people with type 2 diabetes mellitus (T2DM) vs. other antihyperglycemic (AHG) active comparators or placebo.

## 2 Materials and methods

The Preferred Reporting Items for Systematic Reviews and Meta-Analyses (Stewart et al., 2015) guidelines were followed. The study protocol was registered at the University of York, with a regeneration number PROSPERO CRD (42017067889) (Azhari et al., 2023).

### 2.1 Sources of data

We scrutinized the Web of Science, the Cochrane Central Register of Controlled Trials, the Cochrane Database of Systematic Reviews, MEDLINE, EMBASE, and ClinicalTrials.gov (National Library of Medicine NIH U. S., 2023) for clinical trials from their inception to June 25, 2023. Searches were conducted using a range of medical terminology for currently available SGLT2-I, GLP1-RA, and DPP4-I drugs; T2DM; and stroke (Supplementary Appendix 1). Each class of AHG active comparators was reviewed separately.

### 2.2 Study selection

Randomized controlled trials (RCTs) that enrolled adult participants with T2DM, aged ≥18 years, and reported stroke events and treated with SGLT2-I, GLP1-RA, or DPP4-I compared with other AHG active comparators or placebo were selected.

### 2.3 Extraction of data

The extracted data included information about the baseline characteristics of the enrolled population, the study design, the number of participants, the backgrounds of users or non-users of AHG active comparators, and the duration of follow-up.

### 2.4 Quality assessment

To assess the quality of the evidence within RCTs, each trial was appraised according to the described criteria of bias in the Cochrane Tool Assessment (Higgins et al., 2003) for six domains: selection, detection, performance, attrition, reporting, and other. These domains were rated to be either “high risk,” “unclear,” or “low risk” of bias. The trial was considered to have low-quality evidence if any of the selection, detection, or performance domains were judged as a “high risk” of bias.

The uncertainty of RCTs was assessed within a meta-analysis based on the GRADE methodology (Guyatt et al., 2008), specifying a quality of “high,” “moderate,” “low,” or “very low” to each included trial. If the meta-analysis involved several RCTs with low risk, the quality of the trials was considered “high”. However, a trial's quality score might be downrated if there was a concern about the trial's indirectness, inconsistency, risk of bias, publication bias, or imprecision. The quality of each trial was assessed by H.A. and adjudicated by J.D. to resolve any disagreements.

### 2.5 Outcomes measured

The risk of stroke was the primary outcome. The secondary outcomes included non-fatal stroke reported by subtypes (ischemic or hemorrhagic) or fatal stroke. We also included outcomes of stroke reported as serious or non-serious events, which was defined according to the MedDRA Medical Dictionary for Regulatory Activities (2022). Together, these events were used to explore the collective impact of SGLT2-Is, GLP1-RAs, and DPP4-Is by the AHG active comparators' subclass on the risk of stroke.

### 2.6 Synthesis of data

If RCTs were designed to simultaneously evaluate the risk of stroke by subtypes (ischemic and hemorrhagic stroke), one of these outcomes of stroke were excluded from the intervention of interest arm, and the data for stroke were recalculated by subtypes separately and, following that, vs. our treatment of interest with other AHG active comparators or placebo. Stroke outcome data were collected for both long and short durations if an RCT included multiple follow-up periods.

We grouped each AHG treatment of interest, SGLT2-Is, GLP1-RAs, and DPP4-Is drug class, individually for RCTs with variable doses of these drugs to avoid unit-of-analysis errors. If RCTs with ≥2 intervention arms were designed to simultaneously assess SGLT2-Is or/and GLP1-RAs or/and DPP4-Is, one or two of these drugs were excluded from our intervention of interest arm, recalculated the data separately, and then compared with other AHG active comparators' subclass or placebo. We identified whether participants received an AHG active comparator from the following drug classes: sulfonylurea, metformin, thiazolidinediones, DPP4-Is, GLP1-RAs, α-glucosidase inhibitors, SGLT2-Is, and insulin.

### 2.7 Statistical analyses

Meta-analyses were performed for the primary and secondary stroke outcomes. Stroke events were analyzed, through subgroup sensitivity analyses, to explore the net stroke safety effect of relatively small trials (Phase II or III) vs. large trials (Phase IV), comparing the populations with T2DM, those with different baseline characteristics, those not at risk, and those at risk for chronic kidney disease (CKD) or CVD risk across trials.

Fixed and random effect models (Riley et al., 2011) were applied independently. The heterogeneity levels inter-study were quantified to assess the percentage of variability across trials by the *I*^2^ statistic using the Q-statistic. If the *I*^2^ value was ≥75, a considerable heterogeneity not owing to chance was indicated (Higgins et al., 2003). To determine the risk of potential reporting bias, Egger linear regression (Egger et al., 1997) and Begg rank correlation (Begg and Mazumdar, 1994) tests were used, which were visually evaluated with funnel plots. Reporting bias was considered absent if the funnel plot displayed a symmetrical inverted funnel.

For the quantitative evaluation, a summary value using pooled meta-analysis data was set. For RCTs with zero events in any treatment of interest arms, we combined risk of stroke using a Cochran Mantel–Haenszel test with a continuity correction of 0.5. For dichotomous outcomes, we calculated the incidence rate per 100 patient-years, 95% confidence intervals (CIs), risk ratios (RRs), absolute risk difference, and absolute risk. However, to avoid data distortion, trials in both arms with zero events were excluded.

To produce graphical outputs and perform statistical analyses, the GRADEpro GDT (GRADEpro GDT, 2022) and Review Manager (RevMan 5, 2022) software were used. For all outcomes and heterogeneity analyses, the statistically significant levels were set at a *p*-value of less than 0.05, and all tests were two-sided.

## 3 Results

**Figure 1:**
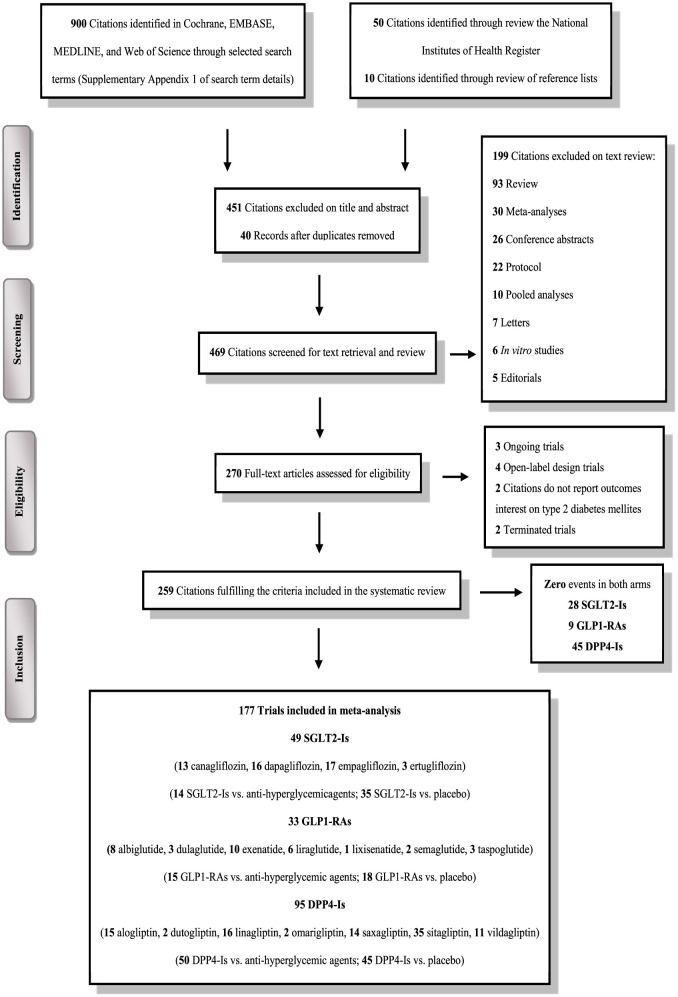
PRISMA flow chart depicting the selection process of the trials included in the systematic review and meta-analysis.

The literature search identified 960 RCTs; of these, only 259 trials satisfied our eligibility criteria (Figure 1). These included 51,691 participants treated with SGLT2-Is, 39,609 were treated with GLP1-RAs, 70,541 were treated with DPP4-Is, and 126,238 participants treated with active comparators. Baseline characteristics are detailed in Supplementary Tables S1–S3.

### 3.1 Study population

Participants were diagnosed previously with T2DM prior to study enrollment. The mean age of the participants was 79 years, and 61.5% of them were male. The control of T2DM was relatively poor, with a glycated hemoglobin A1c (HbA1c) level of approximately 10%. Among 259 RCTs, 55.2% of participants who received SGLT2-Is, 54.1% who received GLP1-RAs, and 43.2% who received DPP4-Is had preexisting CVD. Furthermore, 16.3% of people who received SGLT2-Is, 16.5% who received GLP1-RAs, and 20.3% who received DPP4-Is had CKD. Finally, 10.9% of people who received SGLT2-Is, 5.2% who received GLP1-RAs, and 3.9% who received DPP4-Is had experienced a previous stroke.

### 3.2 Study exposures and interventions

Most trials employed different comparative dosing therapies, which were administered via a subcutaneous injection or orally. Among 259 trials, participants in 59 trials were naïve to AHG active comparators, whereas those in 200 trials were exposed to diverse treatments of AHG agents. The trials' median follow-up durations ranged from 3 to 73 months for SGLT2-Is, 3 to 78 months for GLP1-RAs, and 1 to 99 months for DPP4-Is.

### 3.3 Risk-of-bias assessment

**Figure 2:**
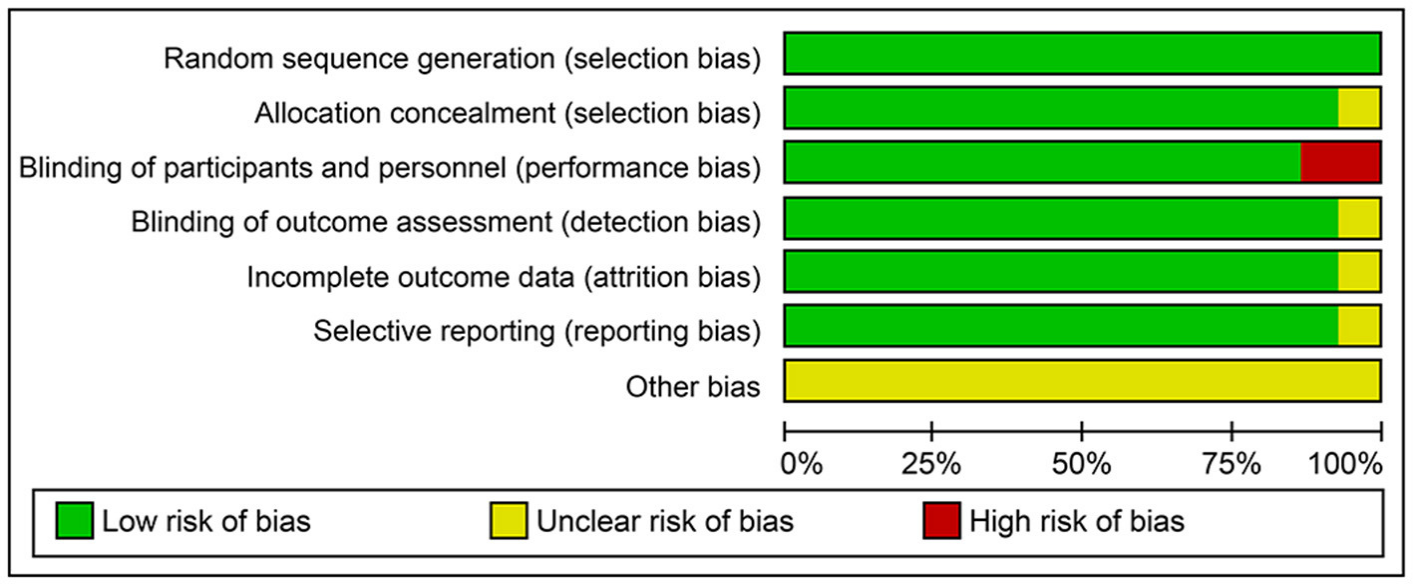
Risk of bias graph: review authors' judgments about each risk of bias item presented as percentages across all included trials in the systematic review and meta-analysis.

In total, 208, 19, and 32 trials were scored with a low-, unclear-, and high-bias risk, respectively. For the random sequence generation, there was an adequate descriptive of stroke outcomes data. In 32% of 32 trials, however, had an open-label design and were scored with a high risk of performance bias. The detection and selection biases had an unclear risk in 19% of trials (Figure 2, Supplementary Figure S1).

### 3.4 Outcome results

In total, 82 trials did not report stroke events, whereas stroke outcome data were reported in 177 trials. For most RCTs, the change in the levels of HbA1c from baseline was the primary outcome. However, 19 RCTs were specifically designed to assess the outcomes of MACEs and included events of stroke as secondary or primary outcomes. Trial results are detailed in Supplementary Tables S4–S6. For 177 RCTs with at least one stroke event, meta-analyses were performed. For all classes, GRADE scores were moderate (Supplementary Tables S7–S24).

### 3.5 SGLT2-Is

In people with T2DM, stroke risk was 4% with the use of SGLT2-Is. Across the SGLT2-Is trials, there was no significant evidence of statistical heterogeneity (*I*^2^ = 0%) or subgroup difference (*p* = 0.58). The overall risk of stroke with the SGLT2-Is class and the comparators class is summarized in Table 1.

**Table 1:** The overall risk of stroke with SGLT2-Is class and comparators class.

**Primary and secondary outcomes**	**Trials**	**Participants**	**Mantel-Haenszel fixed effect**	**Heterogeneity**
**Risk of stroke by subtypes and treatment allocation** • **Follow-up: range 12–318 weeks**	**49**	**82,658**	**RR 0.96; 95% CI [0.86, 1.06]**	***p*** **=** **0.95;** ***I*^2^** **=** **0%**
**Ischemic stroke** • SGLT2-Is vs. placebo • SGLT2-Is vs. AHGs	36 14	65,121 12,067	RR 0.97; 95% CI [0.87, 1.07] RR 0.94; 95% CI [0.58, 1.52]	*p* = 0.89; *I*^2^ = 0% *p* = 0.71; *I*^2^ = 0%
**Hemorrhagic stroke** • SGLT2-Is vs. placebo • SGLT2-Is vs. AHGs	4 3	1,801 3,669	RR 0.50; 95% CI [0.14, 1.85] RR 0.42; 95% CI [0.08, 2.12]	*p* = 0.57; *I*^2^ = 0% *p* = 0.55; *I*^2^ = 0%
**Risk of stroke in T2DM by baseline characteristics** • **Follow-up: range 12–318 weeks**	**49**	**75,738**	**RR 0.96; 95% CI [0.87, 1.06]**	***p*** **=** **0.85;** ***I*^2^** **=** **0%**
**Risk of stroke in T2DM** • SGLT2-Is vs. placebo •SGLT2-Is vs. AHGs	23 14	11,983 12,067	RR 0.63; 95% CI [0.38, 1.03] RR 1.01; 95% CI [0.64, 1.59]	*p* = 0.80; *I*^2^ = 0% *p* = 0.65; *I*^2^ = 0%
**Risk of stroke in T2DM and CVD** • SGLT2-Is vs. placebo	5	20,737	RR 0.93; 95% CI [0.79, 1.09]	*p* = 0.47; *I*^2^ = 0%
**Risk of stroke in T2DM and CKD** • SGLT2-Is vs. placebo	7	30,951	RR 1.01; 95% CI [0.88, 1.15]	*p* = 0.63; *I*^2^ = 0%
**SGLT2-Is by non-SGLT2-Is comparators class** • **Follow-up: range 12–156 weeks**	**9**	**8,845**	**RR 1.00; 95% CI [0.59, 1.71]**	***p*** **=** **0.88;** ***I*^2^** **=** **0%**
• SGLT2-Is vs. metformin •SGLT2-Is vs. sulphonylurea	5 4	4,281 4,564	RR 0.47; 95% CI [0.13, 1.69] RR 1.16; 95% CI [0.64, 2.10]	*p* = 0.91; *I*^2^ = 0% *p* = 0.72; *I*^2^ = 0%

SGLT2-Is, sodium-glucose cotransporter 2 inhibitors; AHGs, antihyperglycemic active comparators; T2DM, type 2 diabetes mellitus; CVD, cardiovascular disease; CKD, chronic kidney disease; RR, risk ratio; CI, confidence interval.

**Figure 3:**
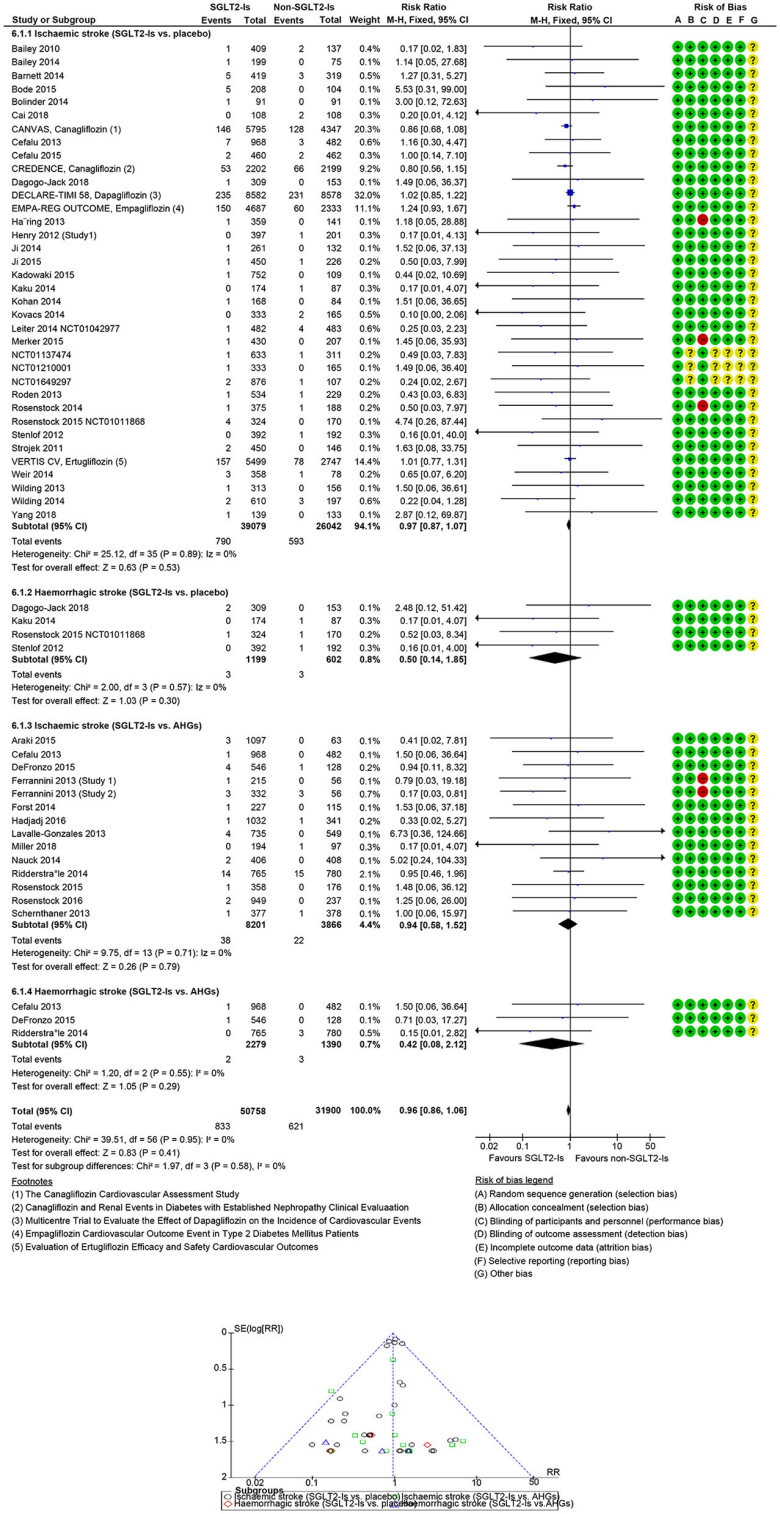
Forest and funnel plot of SGLTS-Is and non-fatal stroke by subtypes, fixed effect model.

In 49 SGLT-Is trials (1,573 strokes; 75,738 participants), SGLT-Is use did not decrease stroke risk (RR: 0.96, 95% CI [0.87, 1.06], *p* = 0.42) vs. non-SGLT-Is use (Supplementary Figures S2, S3). The risk of non-fatal hemorrhagic (RR: 0.50, 95% CI [0.14, 1.85], *p* = 0.30) or non-fatal ischemic (RR: 0.97, 95% CI [0.87, 1.07], *p* = 0.53) events did not decrease vs. placebo. The risk of non-fatal hemorrhagic (RR: 0.42, 95% CI [0.08, 2.12], *p* = 0.29) or non-fatal ischemic (RR: 0.94, 95% CI [0.58, 1.52], *p* = 0.79) events also did not decrease vs. other AHG active comparators (fixed and random effect models; Figure 3, Supplementary Figure S4). Meta-analysis results of the SGLT-Is trials for stroke risk with different baseline characteristics across studies, those diabetic populations not at a risk, or those at risk for CKD or CVD are summarized in the Supplementary Appendix (fixed and random effect models, Supplementary Figures S2, S3).

In SGLT-Is RCTs (6 trials, 124 strokes, 31,327 participants), the risk of fatal hemorrhagic and ischemic stroke did not decrease with SGLT2-Is use (RR: 0.89, 95% CI [0.62, 1.28], *p* = 0.54) vs. non-SGLT2-Is use (fixed and random effect models; Supplementary Figures S5, S6).

### 3.6 GLP1-RAs

In people with T2DM, stroke risk was 16% with the use of GLP1-RAs. Across the GLP1-RAs trials, there was no significant evidence of statistical heterogeneity (*I*^2^ = 0%) or subgroup difference (*p* = 0.94). The overall risk of stroke with the GLP1-RAs class and the comparators class is summarized in Table 2.

**Table 2:** The overall risk of stroke with GLP1-RAs class and comparators class.

**Primary and secondary outcomes**	**Trials**	**Participants**	**Mantel–Haenszel fixed effect**	**Heterogeneity**
**Risk of stroke by subtypes and treatment allocation** • **Follow-up: range 16–339 weeks**	**33**	**76,843**	**RR 0.85; 95% CI [0.77, 0.94]**	***p*** **=** **0.97;** ***I*^2^ = 0%**
**Ischemic stroke** •GLP1-RAs vs. placebo •GLP1-RAs vs. AHGs	18 15	59,696 10,747	RR 0.85; 95% CI [0.77, 0.94] RR 0.80; 95% CI [0.45, 1.44]	*p* = 0.94; *I*^2^ = 0% *p* = 0.69; *I*^2^ = 0%
**Hemorrhagic stroke** •GLP1-RAs vs. placebo •GLP1-RAs vs. AHGs	1 4	3,297 3,103	RR 0.50; 95% CI [0.09, 2.73] RR 1.66; 95% CI [0.41, 6.73]	Not applicable *p* = 0.66; *I*^2^ = 0%
**Risk of stroke in T2DM by baseline characteristics** • **Follow-up: range 16–339 weeks**	**33**	**70,443**	**RR 0.84; 95% CI [0.77, 0.93]**	***p*** **=** **0.91;** ***I*^2^ = 0%**
**Risk of stroke in T2DM** •GLP1-RAs vs. placebo •GLP1-RAs vs. AHGs	11 15	3,692 10,747	RR 0.72; 95% CI [0.36, 1.42] RR 0.85; 95% CI [0.48, 1.49]	*p* = 0.89; *I*^2^ = 0% *p* = 0.68; *I*^2^ = 0%
**Risk of stroke in T2DM and CVD** •GLP1-RAs vs. placebo	4	40,184	RR 0.86; 95% CI [0.76, 0.96]	*p* = 0.35; *I*^2^ = 9%
**Risk of stroke in T2DM and CVD and CKD** •GLP1-RAs vs. placebo	3	15,820	RR 0.82; 95% CI [0.69, 0.98]	*p* = 0.40; *I*^2^ = 0%
**GLP1-RAs by non-GLP1-RAs comparators class** • **Follow-up: range 26–235 weeks**	**15**	**11,051**	**RR 0.91; 95% CI [0.52, 1.57]**	***p*** **=** **0.70;** ***I*^2^ = 0%**
•GLP1-RAs vs. metformin	2	1,367	RR 1.44; 95% CI [0.32, 6.41]	*p* = 0.48; *I*^2^ = 0%
•GLP1-RAs vs. sulphonylurea	3	2,445	RR 0.93; 95% CI [0.30, 2.95]	*p* = 0.19; *I*^2^ = 39%
•GLP1-RAs vs. TZDs (pioglitazone)	2	988	RR 0.21; 95% CI [0.04, 1.20]	*p* = 0.74; *I*^2^ = 0%
•GLP1-RAs vs. GLP1-RAs	3	2,896	RR 1.53; 95% CI [0.40, 5.79]	*p* = 0.52; *I*^2^ = 0%
•GLP1-RAs vs. insulin	5	3,355	RR 1.13; 95% CI [0.41, 3.14]	*p* = 0.69; *I*^2^ = 0%

GLP1-RAs, glucagon-like peptide-1 receptor agonists; AHGs, antihyperglycemic active comparators; T2DM, type 2 diabetes mellitus; CVD, cardiovascular disease; CKD, chronic kidney disease; RR, risk ratio; CI, confidence interval.

**Figure 4:**
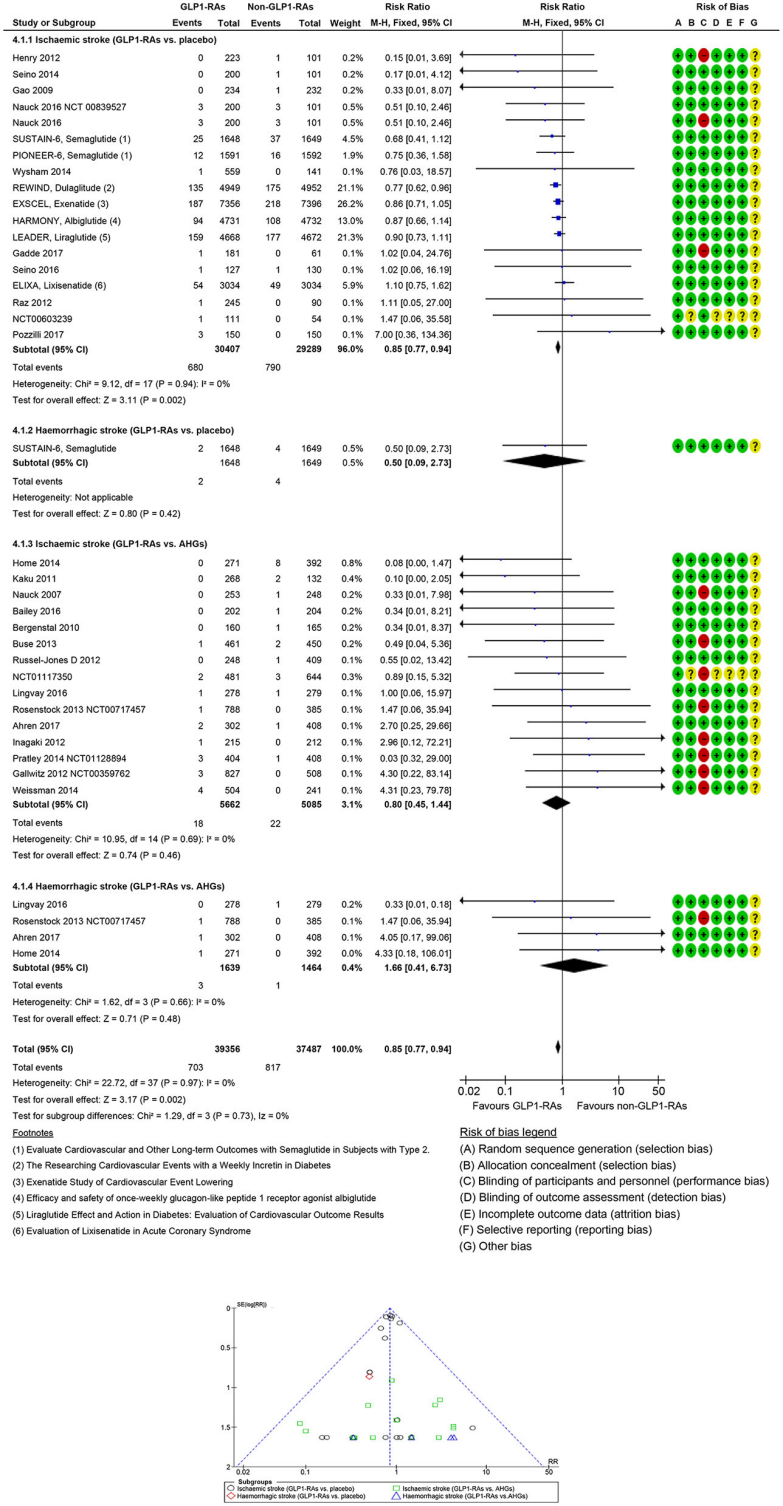
Forest and funnel plot of GLP1-RAs and non-fatal stroke by subtypes, fixed effect model.

In 33 GLP1-RAs trials (1,637 strokes, 70,443 participants), GLP1-RAs use did decrease stroke risk significantly (RR: 0.84, 95% CI [0.77, 0.93], *p* = 0.0005) vs. non-GLP1-RAs use (Supplementary Figures S7, S8). GLP1-RAs use did decrease non-fatal ischemic events (RR: 0.85, 95% CI [0.77, 0.94], *p* = 0.002) but did not decrease non-fatal hemorrhagic events (RR: 0.50, 95% CI [0.09, 2.73], *p* = 0.42) vs. placebo. The risk of non-fatal hemorrhagic (RR: 1.66, 95% CI [0.41, 6.73], *p* = 0.48) or non-fatal ischemic (RR: 0.80, 95% CI [0.45, 1.44], *p* = 0.46) events did not decrease vs. other AHG active comparators (fixed and random effect models; Figure 4, Supplementary Figure S9). Meta-analysis results of GLP1-RAs trials for stroke risk with different baseline characteristics across studies, those diabetic populations not at risk, or those not at risk for CKD or CVD are summarized in the Supplementary material (fixed and random effect models; Supplementary Figures S7, S8).

In the GLP1-RAs RCTs (7 trials, 181 strokes, 46,097 participants), the risk of fatal hemorrhagic or ischemic stroke did not decrease with GLP1-RAs use (RR: 0.77, 95% CI [0.58, 1.03], *p* = 0.08) vs. non-GLP1-RAs use (fixed and random effect models; Supplementary Figures S10, S11).

### 3.7 DPP4-Is

In people with T2DM, stroke risk was 9% with the use of DPP4-Is. Across DPP4-Is trials, there was no significant evidence of statistical heterogeneity (*I*^2^ = 0%) or subgroup difference (*p* = 0.44). The overall risk of stroke with a DPP4-Is class drug and the comparators class is summarized in Table 3.

**Table 3:** The overall risk of stroke with DPP4-Is class and comparators class.

**Primary and secondary outcomes**	**Trials**	**Participants**	**Mantel–Haenszel fixed effect**	**Heterogeneity**
**Risk of stroke by subtypes and treatment allocation** • **Follow-up: range 4–432 weeks**	**95**	**126,177**	**RR 0.92; 95% CI [0.82, 1.02]**	***p*** **=** **1.00;** ***I*^2^ = 0%**
**Ischemic stroke** • DPP4-Is vs. placebo • DPP4-Is vs. AHGs	44 51	65,549 43,694	RR 0.95; 95% CI [0.84, 1.07] RR 0.82; 95% CI [0.67, 1.01]	*p* = 0.97; *I*^2^ = 0% *p* = 0.99; *I*^2^ = 0%
**Hemorrhagic stroke** • DPP4-Is vs. placebo • DPP4-Is vs. AHGs	6 13	3,962 12,972	RR 0.68; 95% CI [0.22, 2.12] RR 1.29; 95% CI [0.58, 2.90]	*p* = 0.84; *I*^2^ = 0% *p* = 1.00; *I*^2^ = 0%
**Risk of stroke in T2DM by baseline characteristics** • **Follow-up: range 4–432 weeks**	**95**	**109,243**	**RR 0.91; 95% CI [0.83, 1.01]**	***p*** **=** **1.00;** ***I*^2^ = 0%**
**Risk of stroke in T2DM** • DPP4-Is vs. placebo • DPP4-Is vs. AHGs	34 44	17,856 35,102	RR 0.74; 95% CI [0.50, 1.11] RR 0.75; 95% CI [0.55, 1.04]	*p* = 0.93; *I*^2^ = 0% *p* = 0.96; *I*^2^ = 0%
**Risk of stroke in T2DM and CVD** • DPP4-Is vs. placebo • DPP4-Is vs. AHGs	6 2	40,912 6,355	RR 0.98; 95% CI [0.85, 1.11] RR 0.85; 95% CI [0.66, 1.10]	*P* = 0.80; *I*^2^ = 0% *P* = 0.35; *I*^2^ = 0%
**Risk of stroke in T2DM and CKD** • DPP4-Is vs. placebo • DPP4-Is vs. AHGs	5 4	7,775 1,243	RR 0.90; 95% CI [0.67, 1.20] RR 1.42; 95% CI [0.62, 3.25]	*p* = 0.93; *I*^2^ = 0% *p* = 0.62; *I*^2^ = 0%
**DPP4-Is by non-DPP4-Is comparators class** • **Follow-up: range 4–432 weeks**	**58**	**50,464**	**RR 0.87; 95% CI [0.72, 1.04]**	***p*** **=** **0.94;** ***I*^2^ = 0%**
•DPP4-Is vs. metformin	11	10,734	RR 1.11; 95% CI [0.58, 2.12]	*p* = 0.90; *I*^2^ = 0%
•DPP4-Is vs. sulphonylurea	18	22,666	RR 0.79; 95% CI [0.63, 0.99]	*p* = 0.53; *I*^2^ = 0%
•DPP4-Is vs. TZDs (pioglitazone)	14	8,587	RR 0.73; 95% CI [0.39, 1.36]	*p* = 0.94; *I*^2^ = 0%
•DPP4-Is vs. α-glucosidase-Is	3	1,515	RR 1.51; 95% CI [0.40, 5.72]	*p* = 1.00; *I*^2^ = 0%
•DPP4-Is vs. DPP4-Is	2	1,443	RR 0.25; 95% CI [0.03, 2.21]	*p* = 0.82; *I*^2^ = 0%
•DPP4-Is vs. GLP1-RAs	3	1,268	RR 3.02; 95% CI [0.82, 11.07]	*p* = 1.00; *I*^2^ = 0%
•DPP4-Is vs. SGLT2-Is	5	3,222	RR 1.34; 95% CI [0.57, 3.11]	*p* = 0.17; *I*^2^ = 37%
•DPP4-Is vs. insulin	2	1,029	RR 2.19; 95% CI [0.33, 14.42]	*p* = 0.90; *I*^2^ = 0%

DPP4-Is, dipeptidyl peptidase-4 inhibitors; AHGs, antihyperglycemic active comparators; T2DM, type 2 diabetes mellitus; CVD, cardiovascular disease; CKD, chronic kidney disease; GLP1-RAs, glucagon-like peptide-1 receptor agonists; SLGT2-Is, Sodium-glucose cotransporter 2 inhibitors; RR, risk ratio; CI, confidence interval.

**Figure 5:**
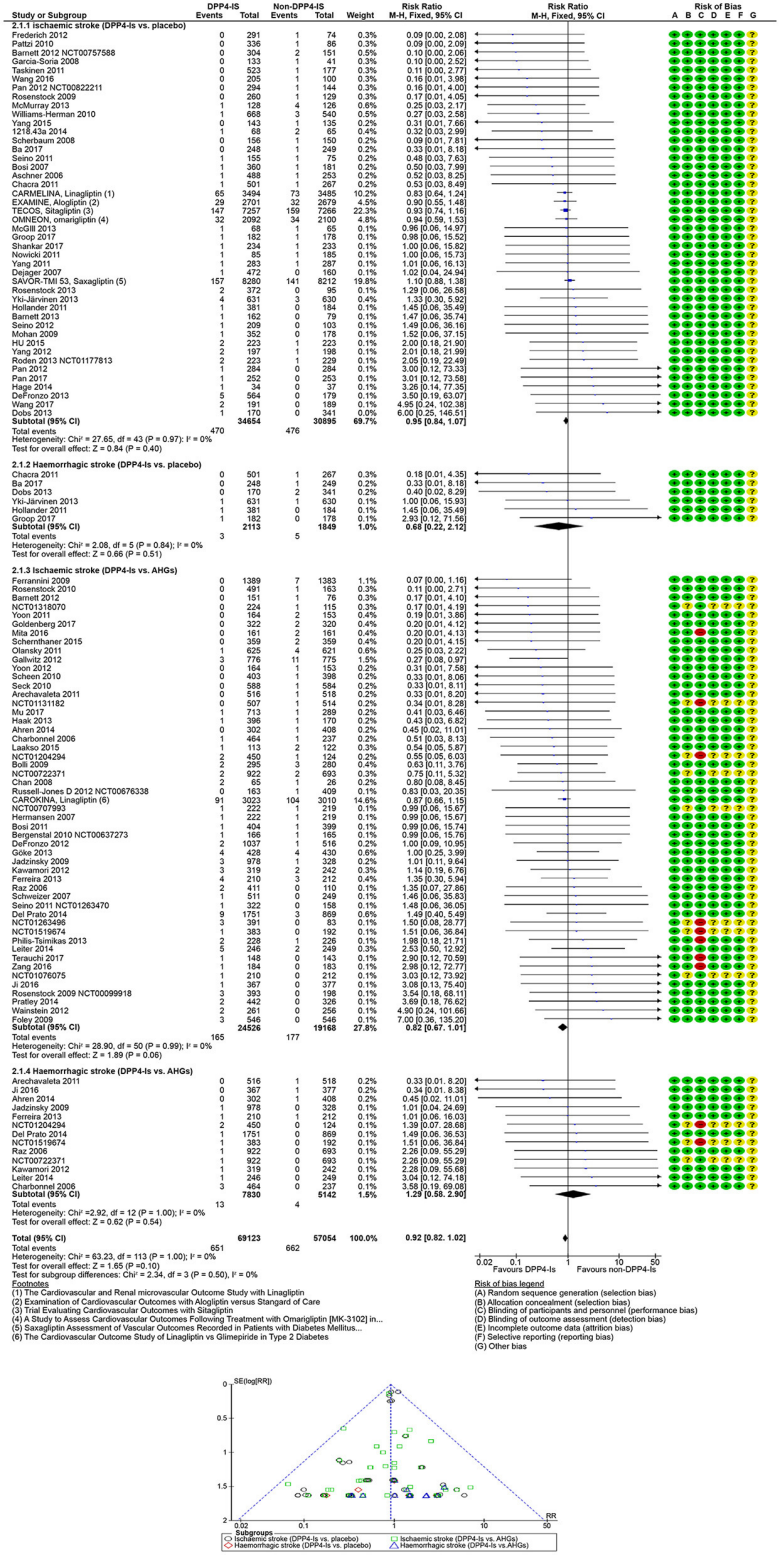
Forest and funnel plot of DPP4-Is and non-fatal stroke by subtypes, fixed effect model.

In 95 DPP4-Is trials (1,488 strokes, 109,243 participants), DPP4-Is use did not decrease stroke risk (RR: 0.91, 95% CI [0.83, 1.01], *p* = 0.07) vs. non-DPP4-Is use (Supplementary Figures S12, S13). The risk of non-fatal hemorrhagic (RR: 0.68, 95% CI [0.22, 2.12], *p* = 0.51) or non-fatal ischemic (RR 0.95; 95% CI [0.84, 1.07]; *p* =0.40) events did not decrease vs. placebo. The risk of non-fatal hemorrhagic (RR: 1.29, 95% CI [0.58, 2.90], *p* =0.54) or non-fatal ischemic (RR: 0.82, 95% CI [0.67, 1.01], *p* = 0.06) events also did not decrease vs. other AHG active comparators (fixed and random effect models; Figure 5, Supplementary Figure S14). Meta-analysis results of DPP4-Is trials for stroke risk with different baseline characteristics across studies, those diabetic populations not at risk, and those at risk for CKD or CVD risk are summarized in the Supplementary material (fixed and random effect models; Supplementary Figures S12, S13).

In the DPP4-Is RCTs (10 trials, 181 strokes, 48,102 participants), the risk of fatal hemorrhagic or ischemic stroke did not decrease with DPP4-Is use (RR: 0.93, 95% CI [0.70, 1.24], *p* = 0.64) vs. non-DPP4-Is use or placebo (fixed and random effect models; Supplementary Figures S15, S16).

Meta-analysis results of trials for risk of stroke by clinical trial size (Supplementary Figures S17–S22), stratification of new AHG active comparators by antidiabetic treatments subclasses (Supplementary Figures S23–S28), and stratification by AHG active comparators of individual subclasses (Supplementary Figures S29–S34) are detailed in the Supplementary material.

## 4 Discussion

Uncertainty surrounding the impacts of new antidiabetic drugs on the risk of strokes has raised questions regarding cerebrovascular safety in the treatment of people with T2DM. To better understand the risk of strokes with these emerging antidiabetic drugs, we conducted a comprehensive review to inform about their effects on the risk of stroke for clinical use or further studies. The main finding was that GLP1-RAs use decreased non-fatal stroke risk vs. placebo, driven by a reduction in ischemic stroke risk in people with T2DM. However, the use of SGLT2-Is or DPP4-Is did not decrease fatal and non-fatal stroke risk vs. placebo.

GLP1-RAs displayed the strongest evidence for an effect on stroke risk. However, we did not observe an effect on fatal stroke in trials in which there was a comparison with other AHG comparators. The observed trend toward less stroke risk with GLP1-RAs use perhaps might be explained by the fact that GLP1 receptors are expressed in a wider range of the body, such as the brain, the heart (Ban et al., 2008), and the pancreas (Darsalia et al., 2015), and that GLP1-RAs, unlike DPP4-Is, cross the blood–brain barrier. The observed (non-significant) increase in the events of stroke with the use of SGLT2-Is was derived mainly from empagliflozin (Zinman et al., 2015). It has been suggested that SGLT2-I alleviates the size of the brain infarct by increasing the levels of ketone (Ferrannini et al., 2016), thereby decreasing oxidative stress to provoke the neuroprotective effects of macrophage (Bazzigaluppi et al., 2018). It is also possible that such a mechanistic difference lies in SGLT2-Is potentially reducing stroke risk through cardiovascular and renal effects, whereas GLP1-RAs may impact stroke risk differently due to their predominantly cardiovascular actions and potential neuroprotective effects. The EMPA-REG OUTCOME (Zinman et al., 2015) trial, however, reported increased levels of hematocrit and viscosity of blood, which they attributed to raised osmotic diuresis, a natriuretic effect, that subsequently increase the risk of dehydration. This could explain the non-significant paradox (Imprialos et al., 2016) of stroke with the use of SGLT2-Is.

Participant characteristics may influence treatment effects. GLP1-RA use, but not SGLT2-I or DPP4-I, appears to decrease non-fatal strokes in diabetic people at risk of CKD or CVD compared with those diabetics without a CKD- or CVD-related risk. Those people represent a growing target population for glycemic controls in the prevention of macro- and microvascular disease progression. Whether these favorable impacts are related to their classes or subclasses directly affect stroke risk would translate differently or similarly if they were initiated early in the prevention of stroke remains uncertain as a limited number of diabetic cases had a previous history of stroke in the trials included in this review. The appearing maximum and minimum magnitude on the risk of stroke therefore may direct us on their secondary cardiovascular and neurovascular preventions independently of their glycemia effects.

To predict the risk of stroke based on the size of RCT, we attempt to stratify the antidiabetic drugs by their subclasses to avoid underestimating the results (Pildal et al., 2008). Even though diabetic people exhibit a stroke risk reduction with GLP1-RAs, the perceived benefits were mainly driven by large trials and placebo. Individual GLP1-RA subclasses in larger trials, however, yielded a non-significant risk of stroke, except for dulaglutide (Gerstein et al., 2019) and semaglutide (Marso et al., 2016). Although pharmacodynamic profiles are similar, differences between individual agents are reflected in their pharmacokinetic properties (Cefalu et al., 2018), including the long-acting effects (once weekly) of semaglutide and dulaglutide in comparison to the short-lasting effects (once daily) of lixisenatide (Aroda, 2018). The pooled post-marketing (Phase IV) trial data revealed that SGLT2-Is and DPP4-Is did not significantly decrease stroke risk vs. placebo, which concurred with Phase II and III trial results. This finding also aligns with an experimental study of ischemic stroke (Darsalia et al., 2015). However, we observed a significant reduction in the risk of stroke with SGLT2-I (canagliflozin) and DPP4-I (dutogliptin) vs. placebo in Phase II and III trials. Even though the review sample size was large, the number of strokes was relatively low in small RCTs, implying that the review analyses across groups may lack statistical power to detect the clinical small difference in the stroke rates. Thus, subgroup analyses should be considered when interpreting these findings.

No evidence of stroke reduction was observed in individual subclasses vs. AHG active comparators. We observed a significant reduction in the risk of stroke with the use of DPP4-I (linagliptin) vs. sulfonylurea in the fixed effect model. Notably, the largest post-marketing (CAROLINA; Rosenstock et al., 2019) trial in this meta-analysis did not observe a stroke risk reduction with DPP4-Is vs. glimepiride. The review meta-analyses of the pre-marketing (Phase II and III) trials were therefore relatively insensitive in the assessment of CVD risk. These findings necessitate the need to conduct a dedicated post-marketing (Phase IV) trial to substantiate any signaling for stroke reduction. The heterogeneity of trial design across trial populations may therefore influence the estimated stroke risk with DPP4-Is vs. sulfonylurea.

### 4.1 Clinical implications

In the management of diabetes, the chemotherapeutic advancement of new antidiabetic drugs has challenged the abilities of researchers to demonstrate their favorable neuroprotective effects. The clinical evidence remains uncertain whether the mortality rates of stroke was increased with sulfonylurea (Phung et al., 2013) or if it decreased with the use of metformin (Griffin et al., 2017). In this meta-analysis, when 100 diabetic people at risk of MACEs were treated with GLP1-RAs for 5 years, the risk of non-fatal strokes would be expected to decrease in 2 people (0 to 1 fewer) without an increase in the risk of fatal strokes vs. SGLT2-Is, DDP4-Is, or placebo. Previous meta-analyses on SGLT2-Is (Saad et al., 2017), GLP1-RAs (Darsalia et al., 2015; Mahmouda et al., 2017), and DPP4-Is (Darsalia et al., 2015; Savaresea et al., 2016; Mahmouda et al., 2017; Rehman et al., 2017; Xu et al., 2017) yielded discordant results for the risk of MACEs and stroke. However, unlike the observed neuroprotective putative effects of pioglitazone in diabetic and non-diabetic individuals with a previous history of stroke (Lee et al., 2017). No such impacts were observed with these antidiabetic drugs in stroke populations.

### 4.2 Strengths and limitations

This review evaluates the impacts of SGLT2-I, GLP1-RA, and DDP4-I subclasses and classes in comparison to other AHG active comparators and placebo on the risk of non-fatal and fatal ischemic and hemorrhagic stroke in people with T2DM regardless of their risk for either CVD or CKD. One caveat of this review is that the generalizability of stroke outcome results may only be applicable to a diabetic population at risk of strokes, which may affect the outcomes' generalizability to a population with a previous history of strokes. Even though most of the included studies were conducted on Phase IV trials with a relatively few fatal stroke events and longer follow-ups period, fatal or non-fatal outcomes of stroke were obtained from secondary or primary endpoints of MACEs, as an exposure time to first stroke, or censoring events. None of these included trials stratified or predicted the recurrent stroke events according to etiological stroke type or subtype or by their severity across gender differences. Additionally, findings from older participants with poor metabolic control and an average age of 79 years may not extend to younger populations or those with better initial control of their diabetes. Consequently, the target population with diabetes appeared to exhibit diverse antidiabetic treatment responses, suggesting a need to tailor therapeutic strategies in diabetes management to potentially reduce stroke risk.

### 4.3 Conclusion

The review findings indicate that GLP1-RAs, but not SGLT2-Is or DPP4-Is, decrease non-fatal stroke risk in diabetic people. These findings may guide the treatment decisions of stroke clinicians when treating diabetic people at risk of stroke and MACEs, as well as inform future trial designs for primary and secondary stroke mortality prevention and functional outcomes with these new antidiabetic drugs.

## Data availability statement

The original contributions presented in the study are included in the article/Supplementary material, further inquiries can be directed to the corresponding author.

## Author contributions

HA: Conceptualization, Data curation, Formal analysis, Funding acquisition, Investigation, Methodology, Project administration, Resources, Software, Validation, Visualization, Writing – original draft, Writing – review & editing. JD: Investigation, Supervision, Visualization, Writing – review & editing.
