# Supplementary material for: The impacts of new antidiabetic drugs on the risk of ischemic and hemorrhagic strokes: a comprehensive review and meta-analysis of clinical trials
Source: Front Stroke. 2024 Jun 20;3:1363954. doi: 10.3389/fstro.2024.1363954 (PMC12802736; doi:10.3389/fstro.2024.1363954)
Supplement: Supplementary file 1 [file Data_Sheet_1.PDF]

The impacts of new antidiabetic drugs on the risk of ischemic and hemorrhagic strokes: A comprehensive review and metanalysis of clinical trials

Hala Azhari,[*1,2] M.D., Jesse Dawson,[2] M.D.

[1]College of Medicine and Pharmacy, Umm Al-Qura University, Makkah, Saudi Arabia

[2]School of Cardiovascular and Metabolic Health, University of Glasgow, Glasgow, UK

[*]**Corresponding Author:** Hala F. Azhari, M.D., MSc., Ph.D.

Assistant Professor of Clinical Cardiovascular and Stroke Medicine, Clinical Pharmacology, Department of Pharmacology and Toxicology, College of Medicine and Pharmacy, Umm Al-Qura University, Makkah, Saudi Arabia

Postal address: P.O. Box: 13578, Al-Ta'eef Road, Makkah 24243, Saudi Arabia

Tel. Number: +966 125 501 000

Fax Number: 4200

E-mail: hfazhari@uqu.edu.sa

Table of contents

Supplementary Appendix 1 of search terms details......9

Cochrane Central Register of Controlled Trials (CENTRAL) 9

MEDLINE (Ovid) search strategy 12

EMBASE search strategy......15

Web of Science search strategy......19

Risk of bias outcomes......20

Trial baseline characteristic outcomes......21

DPP4-Is 21

GLP1-RAs......34

SGLT2-Is 39

Stroke outcome results......47

DPP4-Is 47

GLP1-RAs......59

SGLT2-Is 63

Meta-analysis and GRADE outcomes......71

Stroke risk by clinical trial baseline characteristic and stroke risk by subtypes..72

SGLT2-Is and stroke by baseline characteristic 72

GRADE evidence......75

SGLT2-Is and non-fatal stroke subtypes......77

GRADE evidence......80

SGLT2-Is and fatal stroke risk......82

GRADE evidence 83

GLP1-RAs and stroke by baseline characteristic 84

GRADE evidence 87

GLP1-RAs and non-fatal stroke by subtypes 89

GRADE evidence 92

GLP1-RAs and fatal stroke risk 94

GRADE evidence 95

DPP4-Is and stroke by baseline characteristic..98

GRADE evidence 102

DPP4-Is and non-fatal stroke by subtypes...104

GRADE evidence 107

DPP4-Is and fatal stroke risk...109

GRADE evidence 110

Stroke risk by clinical trial size 112

SGLT2-Is and stroke by clinical trial size..112

GRADE evidence 115

GLP1-Ras and stroke by clinical trial size 117

GRADE evidence 119

DPP4-Is and stroke by clinical trial size 121

GRADE evidence 125

Stroke risk by treatment subclass 127

SGLT2-Is and stroke by SGLT2-Is subclass..127

GRADE evidence 131

GLP1-RAs and stroke by GLP1-RAs subclass 134

GRADE evidence **138**

DPP4-Is and stroke by DPP4-I subclass 142

GRADE evidence **147**

Stroke risk by AHG subclass **150**

SGLT2-Is and stroke by AHGs subclass 150

GRADE evidence **151**

GLP1-RAs and stroke by AHG subclass 153

GRADE evidence **156**

DPP4-Is and stroke by AHG subclass 158

GRADE evidence **162**

References **165**

Table of figures

Figure S1. Risk of bias outcomes in individuals' trials included in the meta-analysis........20

Figure S2. Forest and funnel plot of SGLT2-Is and stroke by baseline characteristic, Fixed-effect model...78

Figure S3. Forest and funnel plot of SGLT2-Is and stroke by baseline characteristic, Random-effect model...78

Figure S4. Forest and funnel plot of SGLTS-Is and non-fatal stroke by subtypes, Random-effect model...78

Figure S5. Forest and funnel plot of SGLT2-Is and fatal stroke, Fixed-effect model. 82

Figure S6. Forest and funnel plot of SGLT2-Is and fatal stroke, Random-effect model.....82

Figure S7. Forest and funnel plot of GLP1-RAs and stroke by baseline characteristic, Fixed-effect model...84

Figure S8. Forest and funnel plot of GLP1-RAs and stroke by baseline characteristic, Random-effect model...85

Figure S9. Forest and funnel plot of GLP1-RAs and non-fatal stroke by subtypes, Random-effect model...90

Figure S10. Forest and funnel plot of GLP1-RAs and fatal stroke, Fixed-effect model. 94

Figure S11. Forest and funnel plot of GLP1-RAs and fatal stroke, Random-effect model. 94

Figure S12. Forest and funnel plot of DPP4-Is and stroke by baseline characteristic, Fixed-effect model...99

Figure S13. Forest and funnel plot of DPP4-Is and stroke by baseline characteristic, Random-effect model...100

Figure S14. Forest and funnel plot of DPP4-Is and non-fatal stroke by subtypes, Random-effect model...105

Figure S15. Forest and funnel plot of DPP4-Is and fatal stroke, Fixed-effect model........109

Figure S16. Forest and funnel plot of DPP4-Is and fatal stroke, Random-effect model. ..109

Figure S17. Forest and funnel plot of SGLT2-Is and stroke by clinical trial size, Fixed-effect model..113

Figure S18. Forest and funnel plot of SGLT2-Is and stroke by clinical trial size, Random-effect model...114

Figure S19. Forest and funnel plot of GLP1-RAs and stroke by clinical trial size, Fixed-effect model...117

Figure S20. Forest and funnel plot of GLP1-RAs and stroke by clinical trial size, Random-effect model...118

Figure S21. Forest and funnel plot of DPP4-Is and stroke by clinical trial size, Fixed-effect model..122

Figure S22. Forest and funnel plot of DPP4-Is and stroke by clinical trial size, Random-effect model...123

Figure S23. Forest and funnel plot of SGLT2-Is and stroke by subclass, Fixed-effect model. 128

Figure S24. Forest and funnel plot of SGLT2-Is and stroke by subclass, Random-effect model..129

Figure S25. Forest and funnel plot of GLP1-RAs and stroke by GLP1-RAs subclass, Fixed-effect model...135

Figure S26. Forest and funnel plot of GLP1-RAs and stroke by GLP1-RAs subclass, Random-effect model...136

Figure S27. Forest and funnel plot of DPP4-Is and stroke by DPP4-Is subclass, Fixed-effect model..143

Figure S28. Forest and funnel plot of DPP4-Is and stroke by DPP4-Is subclass, Random-effect model...144

Figure S29. Forest and funnel plot of SGLT2-Is and stroke by AHGs subclass, Fixed-effect model.......150
Figure S30. Forest and funnel plot of SGLT2-Is and stroke by AHGs subclass, Random-effect model.......150
Figure S31. Forest and funnel plot of GLP1-RAs and stroke by AHGs subclass, Fixed-effect model.......153
Figure S32. Forest and funnel plot of GLP1-RAs and stroke by AHGs subclass, Random-effect model.......154
Figure S33. Forest and funnel plot of DPP4-Is and stroke by AHGs subclass, Fixed-effect model.......159
Figure S34. Forest and funnel plot of DPP4-Is and stroke by AHGs subclass, Random-effect model.......160

Table of tables

Table S1. Trial baseline characteristic of DPP4-Is 21

Table S2. Trial baseline characteristic of GLP1-RAs...34

Table S3. Trial baseline characteristic of SGLT2-Is...39

Table S4. Trial stroke outcomes with DPP4-Is...47

Table S5. Trial stroke outcomes with GLP1-RAs 59

Table S6. Trial stroke outcomes with SGLT2-Is 63

Table S7. GRADE evidence profile of SGLT2-Is and stroke by baseline characteristic 74

Table S8. GRADE evidence profile of SGLT2-Is and non-fatal stroke subtypes 79

Table S9. GRADE evidence profile of SGLT2-Is and fatal stroke risk...............................83

Table S10. GRADE evidence profile of GLP1-RAs by baseline characteristic 86

Table S11. GRADE evidence profile of GLP1-RAs and non-fatal stroke by subtypes 91

Table S12. GRADE evidence profile of GLP1-RAs and fatal stroke risk...........................95

Table S13. GRADE evidence profile of DPP4-Is and baseline characteristic.....................95

Table S14. GRADE evidence profile of DPP4-Is and non-fatal stroke by subtypes.........101

Table S15. GRADE evidence profile of DPP4-Is and fatal stroke risk 110

Table S16. GRADE evidence profile of SGLT2-Is and stroke by clinical trial size 115

Table S17. GRADE evidence profile of GLP1-RAs and stroke by clinical trial size 119

Table S18. GRADE evidence profile of DPP4-Is and stroke by clinical trial size............124

Table S19. GRADE evidence profile of SGLT2-Is and stroke by subclass 130

Table S20. GRADE evidence profile of GLP1-RAs and stroke by GLP1-RAs subclass..137

Table S21. GRADE evidence profile of DPP4-Is and stroke by DPP4-Is subclass 145

Table S22. GRADE evidence profile of SGLT2-Is and stroke by AHGs subclass...........151

Table S23. GRADE evidence profile of GLP1-RAs and stroke by AHGs subclass 155

Table S24. GRADE evidence profile of DPP4-Is and stroke by AHGs subclass.............161

Supplementary Appendix 1 of search terms details

Computer assisted literature searches were perform using the Cochrane Library, Ovid Medline, Embase, PubMed and Web of Science databases.

Cochrane Central Register of Controlled Trials (CENTRAL)

#1 [mh ^"cerebrovascular disorders"] or [mh ^"basal ganglia cerebrovascular disease"] or [mh "brain ischemia"] or [mh ^"carotid artery diseases"] or [mh ^"carotid artery thrombosis"] or [mh ^"intracranial arterial diseases"] or [mh ^"cerebral arterial diseases"] or [mh "intracranial embolism and thrombosis"] or [mh stroke]

#2 (isch*emi* near/6 (stroke* or apoplex* or cerebral next vasc* or cerebrovasc* or cva or attack*)):ti,ab,kw

#3 ((brain or cerebr* or cerebell* or vertebrobasil* or hemispher* or intracran* or intracerebral or infratentorial or supratentorial or middle next cerebr* or mca* or "anterior circulation") near/5 (isch*emi* or infarct* or thrombo* or emboli* or occlus* or hypoxi*)):ti,ab,kw

#4 (tia or tias):ti,ab,kw

#5 [mh ^"embolism, paradoxical"]

#6 [mh ^"heart atria"] and ([mh ^embolism] or [mh ^thromboembolism])

#7 ((paradoxic* or crossed) near/5 embolism*):ti,ab,kw

#8 (cryptogenic near/5 stroke):ti,ab,kw

#9 (#1 or #2 or #3 or #4 or #5 or #6 or #7 or #8)

#10 MeSH descriptor: [Diabetes Mellitus] this term only

#11 ((diabetes or diabetic* or "diabetes mellitus") near/1 ("type 2" or "type II" or "type ii" or "non-insulin dependent" or "non insulin dependent" or "noninsulin dependent" or "adult onset" or "mature onset" or "late onset")).ti,ab,kw

#12 ("diabetes mellitus" not "insulin dependent diabetes mellitus"):kw

#13 NIDDM.ti,ab,kw.

#14 ((diabetic next nephropath*) or "diabetic kidney disease"):ti,ab,kw

#15 (#10 or #11 or # 12 or #13 or #14)

#16 ("dipeptidyl-peptides IV inhibitor" or "dipeptidyl-peptides 4 inhibitor" or "dipeptidyl-peptides IV inhibitors" or "dipeptidyl-peptides 4 inhibitors"):ti,ab,kw

#17 ((DPP4 or DPP 4 or DPP IV) next "inhibitors"):ti,ab,kw

#18 ("alogliptin" or "anagliptin" or "camegliptin" or "dutogliptin" or "gemigliptin" or "linagliptin" or "omarigliptin" or "saxagliptin" or "septagliptin" or "sitagliptin" or "teneligliptin" or "vildagliptin"):ti,ab,kw

#19 gliptin*. ti,ab,kw

#20 incretin*. ti,ab,kw

#21 (#16 or #17 or #18 or #19 or #20)

#22 MeSH descriptor: [Receptors, Glucagon] explode all trees and with qualifier(s): [Agonist - AG]

#23 MeSH descriptor: [Glucagon-Like Peptide 1] explode all trees and with qualifier(s): [Agonists & inhibitors - Al]

#24 ("glucagon-like peptide 1 receptor inhibitors" or "glucagon-like peptide 1 receptor agonists" or "glucagon-like peptide 1 inhibitors" or "glucagon-like peptide 1 agonists" or "GLP-1 receptor inhibitors" or "GLP-1 receptor agonists" or "GLP-1 inhibitors" or "GLP-1 agonists"):ti,ab,kw

#25 ("albiglutide" or "dulaglutide" or "exenatide" or "liraglutide" or "lixisenatide" or "semaglutide" or "taspoglutide"):ti,ab,kw

#26 (#22 or #23 or #24 or #25)

#27 MeSH descriptor: [Sodium-Glucose Linked cotransporter 2] explode all trees and with qualifier(s): [Agonists & inhibitors - Al]

#28 ("sodium glucose linked cotransporter 2 inhibitor" or "sodium glucose linked cotransporter ii inhibitor" or "SGLT 2 inhibitor" or "sodium glucose linked cotransporter 2 inhibitors" or "sodium glucose linked cotransporter ii inhibitors" or "SGLT 2 inhibitors"):ti,ab,kw

#29 ("sodium glucose linked cotransporter" near/3 inhibitor*):ti,ab,kw

#30 ("sodium glucose linked co-transporter" near/3 inhibitor*):ti,ab,kw

#31 (canagliflozin or dapagliflozin or empagliflozin or ertugliflozin or ipragliflozin or luseogliflozin or remogliflozin or sergliflozin or tofogliflizin).kw.

#32 gliflozin*.kw.

#35 (#27 or #28 or #29 or #30 or #31 or #32)

#36 randomized controlled trial.pt.

#37 random*.kw.

#38 randomly.ab.

#39 placebo.ab

#40 clinical trial; as topic/

#41 trial.ti,ab,kw

#42 (#36 or #37 or #38 or #39 or #40)

#43 (#9 and #15 and #21 and #42)

#44 limit #43 to humans

#45 (#9 and #15 and #26 and #42)

#46 limit #45 to humans

#47 (#9 and #15 and #35 and #42)

#48 limit #47 to humans

CENTRAL search syntax

The ‘near’ operator defaults to within 6 words.

'*' indicates truncation.

MEDLINE (Ovid) search strategy

1. cerebrovascular disorders/ or basal ganglia cerebrovascular disease/ or exp brain ischemia/ or carotid artery diseases/ or carotid artery thrombosis/ or intracranial arterial diseases/ or cerebral arterial diseases/ or exp "intracranial embolism and thrombosis"/ or exp stroke/

2. (isch?emi$ adj6 (stroke$ or apoplex$ or cerebral vasc$ or cerebrovasc$ or cva or attack$)).tw.

3. ((brain or cerebr$ or cerebell$ or vertebrobasil$ or hemispher$ or intracran$ or intracerebral or infratentorial or supratentorial or middle cerebr$ or mca$ or anterior circulation) adj5 (isch?emi$ or infarct$ or thrombo$ or emboli$ or occlus$ or hypoxi$)).tw.

4. tia$1.tw.

5. embolism, paradoxical/

6. heart atria/ and (embolism/ or thromboembolism/)

7. ((paradoxic$ or crossed) adj5 embolism$).tw.

8. (cryptogenic adj5 stroke).tw.

9. or/1-8

10. exp Diabetes Mellitus Type 2/

11. Diabetes Mellitus/

12. ((diabetes or diabetes mellitus or diabetic*) adj1 (type 2 or type II or type ii or non-insulin dependent or noninsulin dependent or adult onset or mature onset or late onset)).tw.

13. NIDDM.tw.

14. Diabetic Nephropathies/

15. (diabetic nephropath* or diabetic kidney disease).tw.

16. or/10-15

17. exp Dipeptidyl Peptides-4 Inhibitors/

18. (dipeptidyl peptide-4* or DPP-4).tw.

19. (alogliptin or anagliptin or camegliptin or dutogliptin or gemigliptin or linagliptin or omarigliptin or saxagliptin or septagliptin or sitagliptin or teneligliptin or vildagliptin).tw.

20. gliptin*.tw.

21. incretin*.tw.

22. (dpp adj (4 or IV)).tw.

23. or/17-22

24. exp Glucagon-Like Peptides/

25. (glucagon-like peptide 1 receptor inhibitor* or glucagon-like peptide 1 receptor agonist* or glucagon-like peptide 1 inhibitor* or glucagon-like peptide 1 agonist* or GLP-1 receptor inhibitor* or GLP-1 receptor agonist* or GLP-1 inhibitor* or GLP-1 agonist*).tw.

26. (albiglutide or dulaglutide or exenatide or liraglutide or lixisenatide or semaglutide or taspoglutide).tw.

27. glutide*.tw.

28. or/24-27

29. exp sodium-glucose linked cotransporter-2/

30. (sodium-glucose linked cotransporter 2 inhibitor* or sodium-glucose linked cotransporter ii inhibitor* or SGLT-2 inhibitor*).tw.

31. (sodium-glucose linked cotransporter adj3 inhibitor*).tw.

32. (sodium-glucose linked co transporter adj3 inhibitor*).tw.

33. (canagliflozin or dapagliflozin or empagliflozin or ertugliflozin or ipragliflozin or luseogliflozin or remogliflozin or sergliflozin or tofogliflizin).tw.

34. gliflozin*.tw.

35. or/29-34

36. Randomised Controlled Trials as Topic/

37. random allocation/

38. Controlled Clinical Trials as Topic/

39. control groups/

40. clinical trials as topic/ or clinical trials, phase i as topic/ or clinical trials, phase ii as topic/ or clinical trials, phase iii as topic/ or clinical trials, phase iv as topic/

41. double-blind method/

42. single-blind method/

43. Placebo/

44. placebo effect/

45. Drug Evaluation/

46. Research Design/

47. randomised controlled trial.pt.

48. controlled clinical trial.pt.

49. (clinical trial or clinical trial phase i or clinical trial phase ii or clinical trial phase iii or clinical trial phase iv).pt.

50. random$.tw.

51. (controlled adj5 (trial$ or stud$)).tw.

52. (clinical$ adj5 trial$).tw.

53. ((control or treatment or experiment$ or intervention) adj5 (group$ or subject$ or patient$)).tw.

54. (quasi-random$ or quasi random$ or pseudo-random$ or pseudo random$).tw.

56. ((singl$ or doubl$ or tripl$ or trebl$) adj5 (blind$ or mask$)).tw.

57. placebo$.tw.

58. controls.tw.

59. (RCT or RCTs).tw. or trial.ti.

60. or/36-59

61. 9 and 16 and 23 and 42

62. limit 61 to humans

63. 9 and 16 and 28 and 42

64. limit 63 to humans

65. 9 and 16 and 35 and 42

66. limit 65 to humans

Ovid search syntax

.pt. denotes a Publication Type term;

.ab. denotes a word in the abstract;

.sh. denotes a Medical Subject Heading (MeSH) term;

.ti. denotes a word in the title;

The ‘adj6’ operator indicates within 6 words;

.mp. indicates a search of title, original title, abstract, name of substance word and subject heading word;

‘$’ the dollar sign ($) stands for any character(s) indicates truncation;

tw. text word.

EMBASE search strategy

1. cerebrovascular disease/ or brain infarction/ or brain stem infarction/ or cerebellum infarction/ or exp brain ischemia/ or carotid artery disease/ or exp carotid artery obstruction/ or cerebral artery disease/ or exp cerebrovascular accident/ or exp occlusive cerebrovascular disease/ or stroke patient/

2. (isch?emi$ adj6 (stroke$ or apoplex$ or cerebral vasc$ or cerebrovasc$ or cva or attack$)).tw.

3. ((brain or cerebr$ or cerebell$ or vertebrobasil$ or hemispher$ or intracran$ or intracerebral or infratentorial or supratentorial or middle cerebr$ or mca$ or anterior circulation) adj5 (isch?emi$ or infarct$ or thrombo$ or emboli$ or occlus$ or hypoxi$)).tw.

4. tia$1.tw.

5. paradoxical embolism/

6. exp heart atrium/ and (embolism/ or thromboembolism/)

7. ((paradoxic$ or crossed) adj5 embolism$).tw.

8. (cryptogenic adj5 stroke).tw.

9. or/1-8

10. diabetes mellitus/

11. non insulin dependent diabetes mellitus/

12. ((diabetes or diabetes mellitus or diabetic*) adj1 (type 2 or type II or type ii or non-insulin dependent or noninsulin dependent or adult or adult onset or mature onset or late onset)).tw.

13. NIDDM.tw.

14. diabetic nephropathy/

15. (diabetic nephropath* or diabetic kidney disease).tw.

16. or/10-15

17. dipeptidyl peptidase IV inhibitor/

18. (dipeptidyl-peptidase IV inhibitor* or dipeptidyl-peptidase 4 inhibitor* or ((DPP4 or DPP 4 or DPP IV) adj inhibitor*)).tw.

19. (alogliptin or anagliptin or camegliptin or dutogliptin or gemigliptin or linagliptin or omarigliptin or saxagliptin or sitagliptin or teneligliptin or vildagliptin).tw.

20. or/17-19

21. glucagon like peptide 1 receptor agonist/

22. (glucagon-like peptide 1 receptor inhibitor* or glucagon-like peptide 1 receptor agonist* or glucagon-like peptide 1 inhibitor* or glucagon-like peptide 1 agonist* or GLP-1 receptor inhibitor* or GLP-1 receptor agonist* or GLP-1 inhibitor* or GLP-1 agonist*).tw.

23. (albiglutide or dulaglutide or exenatide or liraglutide or lixisenatide or semaglutide or taspoglutide).tw.

24. 21 or 23

25. sodium glucose linked cotransporter 2 inhibitor/

26. (sodium glucose linked cotransporter 2 inhibitor* or sodium glucose linked cotransporter ii inhibitor* or SGLT 2 inhibitor*).tw.

27. (sodium glucose linked cotransporter adj3 inhibitor*).tw.

28. (sodium glucose linked co transporter adj3 inhibitor*).tw.

29. (canagliflozin or dapagliflozin or empagliflozin or ertugliflozin or ipragliflozin or luseogliflozin or remogliflozin or sergliflozin or tofogliflizin).tw.

30. gliflozin*.tw.

31. or/25-30

32. Randomized Controlled Trial/ or "randomized controlled trial (topic)"/

33. Randomization/

34. Controlled clinical trial/ or "controlled clinical trial (topic)"/

35. control group/ or controlled study/

36. clinical trial/ or "clinical trial (topic)"/ or phase 1 clinical trial/ or phase 2 clinical trial/ or phase 3 clinical trial/ or phase 4 clinical trial/ or controlled clinical trial/

37. Double Blind Method/

38. Single Blind method/ or triple blind method/

39. (random$ or RCT or RCTs).tw.

40. (controlled adj5 (trial$ or stud$)).tw.

41. (clinical$ adj5 trial$).tw.

42. ((control or treatment or experiment$ or intervention) adj5 (group$ or subject$ or patient$)).tw.

43. (quasi-random$ or quasi random$ or pseudo-random$ or pseudo random$).tw.

44. ((control or experiment$ or conservative) adj5 (treatment or therapy or procedure or manage$)).tw.

45. ((singl$ or doubl$ or tripl$ or trebl$) adj5 (blind$ or mask$)).tw.

46. trial.ti

47. assign$.tw.

48. random allocation/

49. allocat$.tw.

50. controls.tw.

51. placebo$.tw.

52. placebo effect/

53. Drug Evaluation/

54. Research Design/

55. or/31-54

56. 9 and 16 and 20 and 55

57. limit 56 to humans

58. 9 and 16 and 24 and 55

59. limit 58 to humans

60. 9 and 16 and 31 and 55

61. limit 60 to humans

Unless otherwise stated, search terms are free text terms; MeSH or [mh] = Medical subject heading (MEDLINE medical index term); exp = exploded MeSH; the dollar sign ($) stands for any character(s); the question mark (?) substitutes one or no characters; tw = text word; pt = publication type; sh = MeSH; adj = adjacent (i.e. number of words within range of search term).

Web of Science search strategy

1. Stroke AND diabetes mellitus type 2 AND dipeptidyl peptidase-4 inhibitors.

2. Stroke AND diabetes mellitus type 2 AND glucagon-like peptide-1 agonist.

3. Stroke AND diabetes mellitus type 2 AND sodium-glucose cotransporter-2 inhibitors.

Articles identified through Cochrane Library **18**, EMBASE **662**, MEDLINE **62**, Web of Science **229**, and **94** publications through Grey Literature.

Risk of bias outcomes

Figure S1. Risk of bias outcomes in individuals' trials included in the meta-analysis

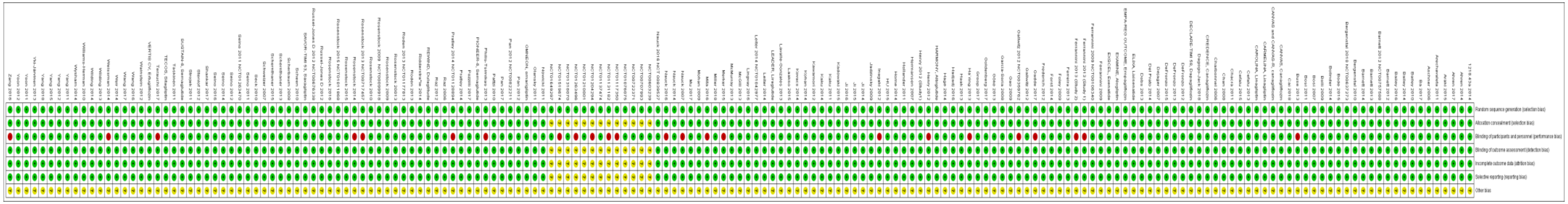

Trial baseline characteristic outcomes

DPP4-Is

Table S1. Trial baseline characteristic of DPP4-Is

Study<br>Trial registry	Population<br>Sample size	T2DM† mean<br>duration (years)	HbA_{1C} levels %	Prior stroke /<br>TIA (*n.*, %)	Mean age<br>(years)[‡]	Males (%)	Study design and country	Parallel<br>arms	DPP4-Is vs. comparators	Background<br>AHGs therapy	DPP4-Is dosage[‡]<br>mg/day	Duration<br>(weeks)
**Alogliptin therapy**												
DeFronzo 2008[1]<br>NCT00286455	T2DM (*n*=328)	6.9±5.9/6.9±5.9	7.9±0.1/7.9±0.1	NR	53.4/53.4	53.9/50.7	Phase III, double-blind, parallel-group, RCT, in 67 centres in 18 countries	3	Alogliptin vs. placebo	None	12.5-25	26
Nauck 2009[2]<br>NCT00286442	T2DM (*n*=527)	6.0±5.0/6.0±5.0	7.9±0.7/8.0±0.9	NR	54.5/56.0	50.8/50.0	Double-blind, parallel-group, RCT, in 115 centres in 15 countries	3	Alogliptin vs. placebo	Metformin	12.5-25	26
Pan 2017[3]<br>NCT01289119	T2DM (*n*=505)	4.3±2.8/2.1±2.8	8.0/8.0	NR	78.6/52.7	52.4/56.3	Phase III, double-blind, parallel-group, RCT, in 30 centres in 3 countries	6	Alogliptin vs. placebo	None ± pioglitazone ± metformin	25	16
Pratley 2009[4]<br>NCT00286494	T2DM (*n*=434)	7.5±5.5/7.8±6.7	8.1±0.9/8.0±0.8	NR	55.4/55.2	59.1/54.6	Phase III, double-blind, parallel-group, RCT, 125 centres in 8 countries	3	Alogliptin vs. placebo	Metformin ± Pioglitazone ± sulfonylurea	12.5-25	24
White 2013[5]<br>NCT00968708	T2DM with ACS (*n*=5380)	7.3±2.8/8.0±1.1[**]	7.1±2.6/8.0±1.1[**]	Stroke 193 (7.2%) vs. 195 (7.2%)	61.0/61.0[**]	67.7/68.0	Double-blind, parallel-group, RCT, in 918 centres in 50 countries, EXAMINE trial	2	Alogliptin[a] vs. placebo	Metformin + sulfonylurea + TZDs +insulin	6.25, 12.5, 25	78
Rosenstock 2009[6]<br>NCT00286429	T2DM (*n*=389)	12.7±6.7/9.3±1.1	12.2±7.1/9.3±1.1	NR	56.0/55.0	41.3/47.7	Phase III, double-blind, parallel-group, RCT, in 60 centres in 16 countries	3	Alogliptin vs. placebo	Insulin ± metformin	12.5, 25	26
Seino 2012[7]<br>NCT01318083;<br>NCT01318135	T2DM (*n*=312)	9.9±6.9/8.5±0.8	9.4±7.4/8.6±0.8	NR	60.1/60.3	68.5/71.0	Phase II/III, double-blind, parallel-group, RCT, in Japan	3	Alogliptin vs. placebo	Sulfonylurea (glimepiride)	12.5, 25	24
Pratley 2009[8]<br>NCT00286468	T2DM (*n*=500)	7.7±6.1/7.7±5.3	8.0/8.0	NR	56.5/57.1	52.4/51.5	Phase III, double-blind, parallel-group, RCT, in 124 centres in 16 countries	3	Alogliptin vs. placebo	Sulfonylurea	12.5-25	26

Seino 2011[9] NCT01263483	T2DM (*n*=230)	7.9±5.5/7.5±6.0	7.9±0.9/8.1±1.2	NR	61.5/62.3	60.6/64.0	Phase II/III, double-blind, parallel-group, RCT	3	Alogliptin vs. placebo	α-glucosidase Is + voglibose	12.5-25	12
DeFronzo 2012[10] NCT00328627	T2DM (*n*=1553)	5.9±5.3/6.0±5.0	8.6±0.7/8.5±0.6	NR	54.0/54.9	41.0/46.0	Phase III, double-blind, parallel-group, RCT, in 90 centres in 19 countries	12	Alogliptin vs. placebo + pioglitazone	Metformin	12.5-25	26
Seino 2011[11] NCT01263470	T2DM (*n*=480)	6.6±6.2/6.4±6.0	7.9±0.9/7.9±0.9	NR	58.7/59.1	58.2/56.0	Phase II, double-blind, parallel-group, RCT, in 54 centres in Japan	6	Alogliptin vs. placebo + α-glucosidase-Is (voglibose)	None	Alogliptin 6.25, 12.5, 25, 50	52
NCT01263496[12]	T2DM (*n*=474)	NR	NR	NR	64.0/64.0	82/67.5	Phase III, open-label, parallel-group, RCT, in Japan	5	Alogliptin vs. α-glucosidase-Is (voglibose)	None	Alogliptin 6.25, 12.5, 25, 50	52
Pratley 2014[13] NCT01023581	T2DM (*n*=768)	3.9/4.0	8.5/8.5	NR	53.6/53.3	55.2/50.5	Phase III, double-blind, parallel-group, RCT, in 201 centres in 13 countries, AM7D trial	7	Alogliptin vs. placebo + metformin	None	Alogliptin 12.5, 25 Alogliptin + metformin¶ 12.5/500, 12.5/1000	34
Bosi 2011[14] NCT00432276	T2DM (*n*=803)	7.2±5.2/6.9±4.6	8.3±0.8/8.1±0.8	NR	54.3/55.9	51.9/51.1	Phase III, double-blind, parallel-group, RCT, in 86 centres in USA	2	Alogliptin vs. metformin + pioglitazone	Metformin + pioglitazon	25	52
NCT00707993[15]	T2DM (*n*=441)	6.3±6.3/5.9±6.3	NR	NR	70.1/69.8	45.9/43.8	Double-blind, parallel-group, RCT, in 80 centres in 11 countries	2	Alogliptin vs. glipizide	None	25	52
Del Prato 2014[16] NCT00856284	T2DM (*n*=2620)	5.5±5.0/5.5±4.9	7.6±0.6/7.6±0.6	NR	55.4/55.4	49.4/50.5	Phase III, double-blind, parallel-group, RCT, ENDURE trial in 262 centres in 31 countries	3	Alogliptin vs. glipizide	Metformin	12.5-25	104
Rosenstock 2010[17] NCT00395512	T2DM (*n*=654)	3.2±3.5/3.2±3.7	8.8±1.0/8.8±1.0	NR	53.0/51.5	46.7/55.2	Phase III, double-blind, parallel-group, RCT, in 161 centres in 23 countries	4	Alogliptin vs. pioglitazone	Pioglitazone	12.5-25	26
NCT01318070[18]	T2DM (*n*=339)	NR	NR	NR	≥/≤65	61.2/66.1	Phase II/III, double-blind, parallel-group, RCT, in 52 centres in Japan	3	Alogliptin vs. pioglitazone	Pioglitazone	12.5-25	12
Mita 2016[19] UMIN000005311	T2DM with CVD (carotid atherosclerosis) (*n*=322)	9.0(5.0-15.0)/8.2(4.0-15.0)**	7.3±0.8/7.2±0.8	NR	64.4/64.8	63.0/61.0	Open-label, blind-end point (PROBE), in 11 centres in Japan, SPEAD-A	2	Alogliptin vs. conventional treatment (other than DPP-4 Is)	Metformin + sulfonylurea + glinides + TZDs + α-glucosidase-Is	25	104
**Dutogliptin therapy**												
Pattzi 2010[20]	T2DM (*n*=422)	NR	8.5±1.0/8.4±1.1	NR	53.0/53.0	53.5/52.3	Phase III, double-blind, parallel-group, RCT, in 73 centres in 5 countries	3	Dutogliptin vs. placebo	Metformin ± TZDs	200, 400	12

Garcia-Soria 2008[21]	T2DM (*n*=174)	5.5±3.5/5.2±2.7	8.7±1.0/8.7±1.0	NR	52.6/51.0	42.0/39.0	Double-blind, parallel-group, RCT, in 26 centres in USA, Mexico and Australia	4	Dutogliptin vs. placebo	Metformin ± glitazone	100, 200, 400	4
**Linagliptin therapy**												
Rosenstock 2019[22] NCT01897532	T2DM with CVD and/or CKD (*n*=6979)	14.8±9.6/14.3±9.2	7.9±1.0/7.9±1.0	NR	66.2/65.7	60.7/63.9	Phase IV, double-blind, parallel-group, RCT, in 605 centres in 27 countries, CARMELINA trial	2	Linagliptin vs. placebo	Metformin ± sulfonylurea ± insulin	5	224**
Del Prato 2010[23] NCT00621140	T2DM (*n*=503)	NR	8.0±0.9/8.0±0.9	NR	56.4/54.4	48.8/47.3	Phase III, double-blind, parallel-group, RCT, in 69 centres in 11 countries	2	Linagliptin vs. placebo	None	5	24
Taskinen 2011[24] NCT00601250	T2DM (*n*=700)	≤1->5/≤1->5	8.1±0.9/8.0±0.9	NR	56.5/56.6	53.2/57.1	Phase III, double-blind, parallel-group, RCT, in 82 centres in 10 countries	2	Linagliptin vs. placebo	Metformin	5	24
Haak 2012[25] NCT00798161	T2DM (*n*=757)	≤1->5/≤1->5	8.7±1.0/8.6±1.0	NR	50.4/45.1	50.4/45.1	Phase III, double-blind, parallel-group, RCT, in 138 centres in 14 countries	7	Linagliptin vs. placebo	Metformin	2.5, 5	24
Søfteland 2017[26] NCT01734785	T2DM (*n*=716)	≤1->10/≤1->10	7.9±0.8/7.9±0.8	NR	54.9/55.9	61.7/55.5	Phase III, double-blind, parallel-group, RCT, in 90 centres in 11 countries	2	Linagliptin vs. placebo	Metformin	5	24
Owens 2011[27] NCT00602472	T2DM (*n*=1055)	≤1->5/≤1->5	8.2±0.0/8.1±0.1	NR	58.3/57.6	46.8/48.3	Phase III, 24 week double-blind, 52 week open-label, parallel-group, RCT, in 100 centres in 11 countries	2	Linagliptin vs. placebo	Metformin + sulfonylurea	5	76
Bajaj 2014[28] NCT00996658	T2DM (*n*=272)	≤1->5/≤1->5	8.4±0.8/8.5±0.7	NR	53.1/55.2	45.4/55.1	Phase III, double-blind, parallel-group, RCT, in 52 centres in 3 countries	2	Linagliptin vs. placebo	Metformin + pioglitazone	5	24
Barnett 2013[29] NCT01084005	T2DM (*n*=241)	≤1->5/≤1->5	7·8±0·8/7·7±0·7	NR	74.9/74.9	71.6/62.0	Phase III, double-blind, parallel-group, RCT, in 34 centres in 5 countries	2	Linagliptin vs. placebo	Metformin ± sulfonylurea ± basal insulin	5	24
Lewin 2012[30] NCT00819091	T2DM (*n*=245)	≤1->5/≤1->5	8.60.9/8.60.7	NR	57.2/56.2	47.8/61.9	Phase III, double-blind, parallel-group, RCT, in 45 centres in 7 countries	2	Linagliptin vs. placebo	Sulfonylurea	5	18
Gomis 2011[31] NCT00641043	T2DM (*n*=389)	NR	8.6±0.9/8.6±0.8	NR	57.7/57.1	58.7/65.4	Phase III, double-blind, parallel-group, RCT, in 43 centres in 7 countries	2	Linagliptin vs. placebo	Pioglitazone	5	24
Yki-Järvinen 2013[32] Duran-Garcia 2015 NCT00954447	T2DM (*n*=1261)	≤1->5/≤1->5	8.3±0.8/8.3±0.9	NR	59.7/60.4	52.1/52.2	Phase III, double-blind, parallel-group, RCT, in 169 centres in 19 countries	2	Linagliptin vs. placebo	Basal insulin + metformin ± pioglitazone	5	52

Wang 2016[33] NCT01215097	T2DM (*n*=305)	NR	7.9±0.8/8.0±0.8	NR	55.1/56.5	49.8/50.0	Phase III, double-blind, parallel-group, RCT, in 19 centres in 3 countries	2	Linagliptin vs. placebo	Metformin ± sulfonylurea ± α-glucosidase-Is ± other AHGs	5	24
1218.43[a] 2014[34] NCT00800683	T2DM with CKD[d] (*n*=133)	NR	8.2±1.1/8.2±0.9	NR	64.0/64.9	66.2/53.8	Phase III, double-blind, parallel-group, RCT, in 53 centres in 6 countries	2	Linagliptin vs. placebo	Pioglitazone, sulfonylurea, glinides, α-glucosidase-Is + insulin	5	52
McGIll 2013[35] PMCID: PMC3554278	T2DM with CKD[d] (*n*=133)	≤1->5/≤1->5	8.2±1.1/8.2±0.9	NR	64.0/54.9	66.2/53.8	Phase III, double-blind, parallel-group, RCT, in 53 centres in 6 countries	2	Linagliptin vs. placebo	Sulfonylurea, glinides, pioglitazone, α-glucosidase-Is, insulin	5	55
Groop 2017[36] NCT01792518	T2DM with CKD on ACE ± ARB (*n*=360)	≤1->10/≤1->10	7.8±0.9/7.9±0.9	NR	61.0/60.1	63.7/63.5	Phase III b, double-blind, parallel-group, RCT, in 74 centres in 12 countries, MARLINA-T2D trial	2	Linagliptin vs. placebo	NR	5	24
Kawamori 2012[37] NCT00654381	T2DM (*n*=561)	≤1->5/≤1->5	8.0±0.7/7.9±0.5/8.0±0.7	CVA 168 (52.7%) vs. 90 (55.6%) vs. 36 (45.0%) vs.	60.8/59.7/58.5	69.9/71.3/70.9	Phase IIb/III, double-blind, parallel-group, RCT, in 47 centres in Japan	4	Linagliptin vs. placebo + α-glucosidase-Is (voglibose)	None	5, 10	26
Mu 2017[38] NCT01708902	T2DM (*n*=1002)	≤1-≤10/≤1-≤10	10.3±0.9/8.7±1.0	NR	51.0/51.8	69.0/63.0	Phase III, double-blind, parallel-group, RCT, in 56 centres in 4 countries	6	Linagliptin/metformin[§] vs. metformin	None	2.5-5	24
Haak 2013[39] NCT00915772	T2DM (*n*=566)	≤1->5/≤1->5	7.5±1.1/7.5±0.9	NR	55.9/55.6	54.8/54.7	Phase III, double-blind, parallel-group, RCT, in 112 centres in 14 countries	3	Linagliptin vs. metformin	Metformin	2.5/500 2.5/1000	54
NCT01204294; 2104[40]	T2DM (*n*=574)	NR	NR	NR	60.8/61.2	69.7/69.8	Phase III, open-label, parallel-group, RCT, in 43 centres in Japan	7	Linagliptin vs. metformin	AHG agents	5	52
Barnett 2012[41] NCT00740051	T2DM (*n*=227)	≤1->5/≤1->5	8.1±1.0/8.1±0.9	NR	56.4/56.7	36.4/43.4	Phase III, double-blind, parallel-group, RCT, 53 centres in 7 countries	2	Linagliptin vs. placebo + sulfonylurea	None	5	52

Laakso 2015[42] NCT01087502	T2DM with CKD (*n*=235)	NR	8.4±0.9/8.0±0.9	NR	67.3/65.9	61.9/64.8	Phase III, double-blind, parallel-group, RCT, in 52 centres in 9 countries	2	Linagliptin vs. placebo + glimepiride	None or TZDs + α-glucosidase-Is ± insulin	5	52
Gallwitz 2012[43] NCT00622284	T2DM (*n*=1551)	≤1->5/≤1->5	7·7±0·9/7·7±0·9	NR	59.8/59.8	60.0/61.0	Double-blind, parallel-group, RCT, in 209 centres in 16 countries	2	Linagliptin vs. glimepiride	Metformin	5	104
Rosenstock 2019[44] NCT01243424	T2DM with high risk CVD (*n*=6072)	6.3 (3.0,11.1)/6.2 (2.9,10.9)**	7.2/7.2	CVD 371 (12.3%) vs. 356 (11.9%)	603.9/64.2	60.8/59.2	Phase III, double-blind, RCT, in 612 centres in 43 countries, CAROLINA trial	4	Linagliptin vs. glimepiride	NR	5	432
**Omarigliptin therapy**												
Gantz 2017[45] NCT01703208	T2DM with established CVD (*n*= 4202)	12.0±7.6/12.1±8.0	8.0±0.9/8.0±0.9	NR	63.7/63.6	69.6/70.0	Phase III, double -blind, RCT, multicentre, OMNEON trial	2	Omarigliptin vs. placebo	Metformin ± pioglitazone ± SGLT-2I ± α-glucosidase inhibitor	25	156
Goldenberg 2017[46] NCT01841697	T2DM (*n*=642)	7.0±4.5/7.5±5.6	7.5±0.7/7.5±0.8	NR	57.0/57.6	46.9/54.7	Phase III, double-blind, parallel-group, RCT	2	Omarigliptin vs. sitagliptin	Metformin	Omarigliptin 25 Sitagliptin 100	24
**Saxagliptin therapy**												
Pan 2012[47] NCT00698932	T2DM (*n*=496)	0.8±1.4/8.1±0.8	1.2±2.6/8.2±0.8	NR	51.2/51.6	56.3/54.6	Phase III, double-blind, parallel-group, RCT, in Asian patients	2	Saxagliptin vs. placebo	None	5	24
Rosenstock 2008[48] NCT00950599	T2DM (*n*=423)	1.0±15.5/1.8±23.0 **	7.9±1.2/8.0±0.9	NR	53.3/54.0	57.7/59.4	Phase II, double-blind, parallel-group, RCT, 152 centres in USA	8	Saxagliptin vs. placebo	None	2.5, 5, 10, 20, 40, 100	18
Kumar 2014[49] NCT00918879	T2DM (*n*=213)	0.8±1.2/1.0±1.4	8.3±0.8/8.3±0.7	NR	49.1/48.3	53.3/59.4	Phase III-b, double-blind, parallel-group, RCT, in India	2	Saxagliptin vs placebo	None	5	24
Frederich 2012[50] NCT00316082	T2DM (*n*=365)	1.7±3.0/1.7±2.8	7.9±0.9/7.8±1.0	NR	55.0/55.6	46.0/47.3	Phase III, double-blind, parallel-group, RCT, in 74 centres in 4 countries	5	Saxagliptin vs. placebo	None	2.5, 5	76
Rosenstock 2013[51] NCT00121641	T2DM (*n*=467)	2.7±3.4/2.3±2.7	8.6±0.9/7.9±0.9	NR	53.3/53.9	51.2/49.5	Phase III, double-blind, open-label saxagliptin (10mg/d), parallel-group, RCT, in 135 centres in 6 countries, study 11	5	Saxagliptin vs. placebo	None	2.5, 5, 10	53

DeFronzo 2013[51] NCT00121667	T2DM (*n*=743)	6.4±5.5/6.7±0.9	8.0±5.6/8.1±0.9	NR	54.5/54.8	49.7/53.6	Phase III, double-blind, parallel-group, RCT, in 154 centres in 9 countries, study 14	4	Saxagliptin vs. placebo	Metformin	2.5, 5, 10	206
Yang 2011[52] NCT00661362	T2DM (*n*=570)	5.1±5.0/5.1±4.0	7.9±0.8/7.9±0.8	NR	53.8/54.4	48.1/48.4	Phase III, double-blind, parallel-group, RCT, in Asian patients	2	Saxagliptin vs. placebo	Metformin	5	24
Stenlöf 2010[53] NCT00683657	T2DM (*n*=93)	NR	NR	NR	54.5/55.8	56.5/49.0	Phase III, double-blind, parallel-group, RCT, 27 centres in 8 countries	2	Saxagliptin vs. placebo	Metformin	5	4
White 2014[54] NCT00885378	T2DM (*n*=160)	5.8±6.4/6.2±4.2	7.9±0.9/7.9±0.8	NR	54.0/56.6	54.1/52.3	Phase III, double-blind, parallel-group, RCT, 41 centres in 4 countries	2	Saxagliptin vs. placebo	Metformin	5	12
Chacra 2011[55] NCT00313313	T2DM (*n*=768)	6.9±5.8/6.8±5.7	8.4±0.9/8.4±0.9	NR	55.1/55.1	44.6/46.1	Phase III, double-blind, parallel-group, RCT, in 115 centres in 13 countries	3	Saxagliptin vs. placebo	Glyburide	2.5, 5	76
Nowicki 2011[56] NCT00614939	T2DM with CKD[e] (*n*=170)	15.1±7.5/18.2±8.5	8.5±1.2/8.1±1.1	NR	66.8/66.2	37.6/48.2	Phase III, double-blind, parallel-group, RCT, in 75 centres in 14 countries	2	Saxagliptin vs. placebo	Glinide ± sulfonylurea ± TZDs ± α-glucosidase-Is + insulin	2.5	52
Hollander 2011[57] NCT00295633	T2DM (*n*=565)	5.2±5.1/5.1±5.4	8.3±1.1/8.2±1.1	NR	54.1/54.0	51.1/46.2	Phase III, double-blind, parallel-group, RCT, in 133 centres in 8 countries	3	Saxagliptin vs. placebo	TZDs (pioglitazone + rosiglitazone)	2.5, 5	76
Barnett 2012[58] NCT00757588	T2DM (*n*=455)	11.8±6.9/12.2±7.3	8.7±0.9/8.6±0.8	NR	57.2/57.3	40.0/45.0	Phase III, double-blind, parallel-group, RCT, in 80 centres in 10 countries	2	Saxagliptin vs. placebo	Insulin ± metformin	5	52
Scirica 2013[59] NCT01107886	T2DM with CVD or CRF[b] (*n*=16,492)	10.3±2.8/10.3±2.8	8.0±1.4/8.0±1.4[**]	NR	65.1/65.0	66.6/67.3	Phase IV, double-blind, parallel-group, RCT, in 788 centres in 26 countries, SAVOR–TIMI 53	2	Saxagliptin vs. placebo	None + metformin + sulfonylurea + TZDs +insulin + other AHGs	2.5, 5	109
Henry 2011[60] NCT00374907	T2DM (*n*=36)	2.7±4.4/3.7±4.0	6.9±0.5/6.6±0.6	NR	58.0/55.0	40.0/37.5	Phase III, double-blind, parallel-group, RCT, 3 centres in USA	2	Saxagliptin vs. placebo + metformin	None	5	116
Jadzinsky 2009	T2DM (*n*=1306)	1.7±2.9/1.7±3.1	9.5±1.2/9.4±1.3	NR	52.1/51.8	49.1/49.7	Phase III, double-blind, parallel-group, RCT, 211 centres in 13 countries	4	Saxagliptin vs. metformin	Metformin	5-10	76

Pfützner 2011[61]<br>NCT00327015											
Hermans 2012[62]<br>NCT01006590	T2DM (*n*=286)	6.0±5.3/6.9±6.0	7.7±0.9/7.8±0.8	NR	58.7/58.6	59.9/54.7	Phase III, double-blind, parallel-group, RCT, 50 centres in 7 countries, PROMPT trial	2	Saxagliptin vs. metformin	Metformin	5	24
Neutel 2013[63]<br>NCT00918138	T2DM (*n*=93)	6.2±4.5/5.1± 3.9	8.6±0.9/8.4± 0.9	NR	53.9/50.6	45.7/53.2	Phase III, double-blind, parallel-group, RCT, 21 centres in 4 countries	2	Saxagliptin vs. metformin	Metformin	5	4
Fonseca 2012[64]<br>NCT00960076	T2DM (*n*=282)	6.5±5.4/5.9±5.2	8.4±0.9/8.3±0.9	NR	55.2/55.5	41.3/50.7	Phase III b, double-blind, parallel-group, RCT, 35 centres in 5 countries,	2	Saxagliptin vs. metformin	Metformin	5	18
Göke 2013[65]<br>NCT00575588	T2DM (*n*=858)	5.5±4.5/5.5±4.7	7.7±0.9/7.7±0.9	NR	57.5/57.6	49.5/54.0	Phase III, double-blind, parallel-group, RCT, in 95 centres in 11 countries	2	Saxagliptin vs glipizide	Metformin	5	104
Schernthaner 2015[66]<br>NCT01006603	T2DM (*n*=718)	7.6±6.4/7.6±6.0	7.6±0.7/7.6±0.7	CVA<br>19 (5.3%) vs.<br>21 (5.8%)	72.5/72.7	60.3/63.3	Phase IIIb/IV, double-blind, parallel-group, RCT, in 131 centres in 13 countries	2	Saxagliptin vs. glimepiride	Metformin	5	52
Scheen 2010[67]<br>NCT00666458	T2DM (*n*=801)	6.3±5.0/6.3±4.7	7.7±0.1/7.7±0.0[‡]	NR	58.8/58.1	47.1/50.8	Phase III b, double-blind, parallel-group, RCT, 86 centres in 9 countries	2	Saxagliptin vs. sitagliptin	Metformin	Saxagliptin 5<br>Sitagliptin 100	18
**Sitagliptin therapy**												
Hage 2014[68]<br>NCT00627744	T2DM or IGT with ACS (*n*=71)	IGT=24.0±7.1/23.0±62.0<br>T2DM=10.0±29.0/14.0±38.0	6.8/6.8	Stroke/TIA<br>2 (6.0%) vs.<br>3 (8.0%)	69.0/66.0	85.0/78.0	Phase IV, double-blind, parallel-group, RCT, In Sweden, BEGAMI study	2	Sitagliptin vs. placebo	None	100	12
Mohan 2009[69]<br>NCT00289848	T2DM (*n*=530)	2.1±1.7/8.7±1.0	1.9±1.6/8.8±1.1	NR	51.0/51.0	56.8/59.6	Phase III, double-blind, parallel-group, RCT, in 3 countries	2	Sitagliptin vs. placebo	None	100	18
Barzilai 2011[70]<br>NCT00305604	T2DM (*n*=206)	7.2±35/7.0±47	7.8±10/7.8±9.7	NR	71.6/72.1	47.1/47.1	Phase III, double-blind, parallel-group, RCT	2	Sitagliptin vs. placebo	None	50-100	24
Ji 2016[71]<br>NCT01076088	T2DM (*n*=744)	1.1±0.3/1.1±0.2	NR	NR	53.0/53.0	75.0/61.2	Phase III, double-blind, parallel-group, RCT, in China	6	Sitagliptin vs. placebo	None	50-100	24
Scott 2007[72]<br>NCT00482079	T2DM (*n*=620)	NR	NR	NR	NR	NR	Phase II, double-blind, parallel-group, RCT	6	Sitagliptin vs. placebo	None	NR	21

HU 2015 [73] NCT01289990	T2DM (*n*=446)	≤1->10/≤1->10	8.1±0.8/8.2±0.8	NR	55.1/55.6	63.2/51.0	Phase III, parallel-group, RCT, in 69 centres in 8 countries	2	Sitagliptin vs. placebo	None	100	76
Aschner 2006[74] NCT00087516	T2DM (*n*=741)	NR	8.0±0.9/8.0±0.8	NR	54.2/54.3	52.0/51.4	Double-blind, parallel-group, RCT	3	Sitagliptin vs. placebo	None	100-200	24
Roden 2013[75] NCT01177813	T2DM (*n*=452)	≤1->10/≤1->10	7·9±0·8/7·9±0·8	NR	53.4/55.0	67.2/58.6	Phase III, double-blind, parallel-group RCT, in 124 centres in 9 countries	2	Sitagliptin vs. placebo	None	100	24
Hanefeld 2007[76] NCT00481663	T2DM (*n*=552)	NR	NR	NR	NR	NR	Phase II, double-blind, parallel-group, RCT	5	Sitagliptin vs. placebo	NR	25-50-100	12
Wang 2017[77] NCT01177384	T2DM (*n*=380)	7.4±4.9/8.2±5.7	8.1±0.8/8.1±0.9	NR	56.5/57.8	50.8/51.3	Phase III, double-blind, parallel-group, RCT, in India	2	Sitagliptin vs. placebo	Acarbose (glucobay)	100	24
Yang 2012[78] NCT00813995	T2DM (*n*=395)	NR	8.5±0.9/8.5±0.9	NR	54.1/55.1	46.7/54.5	Phase III, double-blind, parallel-group, RCT	2	Sitagliptin vs. placebo	Metformin	100	24
Raz 2008[79] NCT00337610	T2DM (*n*=190)	NR	9.3±0.9/9.1±0.8	NR	53.6/56.1	51.0/41.5	Phase III, double-blind, parallel-group, RCT	2	Sitagliptin vs. placebo	Metformin	100	30
NCT00420511[80]	T2DM (*n*=21)	2.8±4.0/3.5±8.0	6.2±6.9/6.1±6.9	NR	61.3/60.8	60.0/72.7	Phase II, double-blind, parallel-group, RCT, in Canada, BEST trial	2	Sitagliptin vs. placebo	Metformin	100	48
Bergenstal 2012[81] NCT00754988	T2DM (*n*=274)	6.0±5.0/5.5±3.9	7.9±0.9/8.0±0.8	NR	55.5/56.1	59.3/52.2	Phase III, double-blind, parallel-group RCT in 149 centres in 23 countries, T-Emerge 4 trials	2	Sitagliptin vs. placebo	Metformin	100	156
Gadde 2017[82] NCT01652729	T2DM (*n*=183)	7.9±4.6/8.7±5.8	8.5±1.0/8.5±1.0	NR	53.4/54.3	60.7/54.1	Phase III, open-label, parallel-group, RCT, in 60 centres in USA, DURATION-NEO-2 trial	2	Sitagliptin vs. placebo	Metformin	100	28
Rosenstock 2012[83] NCT00642278	T2DM (*n*=130)	6.0±4.9/6.4±5.0	7.6±0.9/7.8±0.8	NR	53.1/52.5	51.0/53.1	Phase II, double-blind, parallel-group RCT, in 85 centres in 13 countries	2	Sitagliptin vs. placebo	Metformin	100	12
Rosenstock 2013[84] NCT00749190	T2DM (*n*=51)	NR	8.0±0.7/8.1±0.9	NR	58.1/58.7	50.7/50.0	Phase II, open-label parallel-group RCT, in 116 centres in 16 countries	2	Sitagliptin vs. placebo	Metformin	100	12
Ba 2017[85] NCT01590771	T2DM (*n*=497)	7.1±5.4/6.9±4.9	8.6±1.1/8.5±0.9	NR	57.5/56.5	47.0/53.0	Phase III, double-blind, parallel-group, RCT, in China	2	Sitagliptin vs. placebo	Metformin ± sulfonylurea	100	24
Dobs 2013[86] NCT00350779	T2DM (*n*=262)	9.3±5.9/9.4±6.8	8.8±1.0/8.7±1.0	NR	54.4/54.8	56.0/60.0	Double-blind, parallel-group, RCT, in 41 centres in USA, Europe and Asia	2	Sitagliptin vs. placebo	Rosiglitazone + metformin	100	77

Fonseca 2013[87] NCT00885352	T2DM (*n*=313)	9.4±5.8/10.2±6.1	8.8±1.0/8.7±1.0	NR	55.7/65.4	61.8/62.8	Phase III, double-blind, parallel-group, RCT	2	Sitagliptin vs. placebo	Metformin + pioglitazone	100	26
Rosenstock 2006 [88] NCT00086502	T2DM (*n*=353)	6.1±5.4/6.1±5.7	8.1±0.8/8.0±0.8	NR	55.6/56.9	53.1/57.9	Phase III, double-blind, parallel-group, RCT	2	Sitagliptin vs. placebo	Pioglitazone	100	24
Green 2015[89] NCT00790205	T2DM with established CVD (*n*=14,523)	11.6±8.1/7.2±0.5	11.6±8.1/7.2±0.5	CVD 1806 (24.6%) 1782 (42.3%)	65.4/65.5	70.9/70.5	Double-blind, parallel-group, RCT, in 673 centres in 38 countries, TECOS trial	2	Sitagliptin vs. placebo	Metformin + sulfonylurea + TZDs +insulin	50, 100	157[**]
Shankar 2017[90] NCT01590797	T2DM (*n*=467)	11.0±5.0/11.3±5.8	8.7±0.9/8.8±0.9	NR	58.6/56.7	65.6/49.8	Phase III, double-blind, parallel-group, RCT, in China	2	Sitagliptin vs. placebo	± Metformin + insulin	100	24
Vilsbøll 2010[91] NCT00395343	T2DM (*n*=641)	13.0±7.0/12.0±6.0	<8%-≥9%/<8%-≥9%	NR	58.3/57.2	48.8/53.0	Phase III, double-blind, parallel-group, RCT	2	Sitagliptin vs. placebo	Insulin ± metformin	100	24
Mathieu 2015[92] NCT01462266	T2DM (*n*=658)	13.2±6.0/13.7±6.4	8.7±1.0/8.8±1.0	NR	59.3/58.3	45.9/49.8	Phase III, double-blind, parallel-group, RCT	2	Sitagliptin vs. placebo	Insulin ± metformin	100	24
Williams-Herman 2010[93] NCT00103857	T2DM (*n*=1208)	5.9±6.7/5.9±6.9	8.6±1.0/8.6±0.9	NR	53.3/53.4	51.7/48.9	Phase III, double-blind, factorial design RCT	7	Sitagliptin vs. placebo + metformin	Metformin	100	104
Amin 2015[94] NCT01059825	T2DM + hypertension (*n*=484)	6.4±0.9/6.4±0.9	8.2±0.1/8.2±0.1	NR	55.0/55.1	72.7/63.2	Phase II, double-blind, parallel-group, RCT	3	Sitagliptin vs. placebo + metformin	AHG agents	100	12
Aschner 2010[95] NCT00449930	T2DM (*n*=1050)	2.6±3.9/2.1±3.5	7.2±0.7/7.3±0.7	NR	56.0/56.0	47.3/44.8	Phase III, double-blind, parallel-group, RCT	2	Sitagliptin vs. metformin	None	100	24
Olansky 2011[96] NCT00482729	T2DM (*n*=1246)	NR	9.9±1.8/9.8±1.8	NR	49.4/50.0	56.5/57.2	Phase III, double-blind, parallel-group, RCT	2	Sitagliptin/metformin[§] vs. metformin	None	100-200	44
Perez-Monteverde 2011[97] NCT00541450	T2DM (*n*=472)	2.9±2.8/ 3.5±3.7	9.0±1.4/9.1±1.4	NR	50.5/51.7	62.3/59.7	Phase III, parallel placebo-controlled trial	2	Sitagliptin/metformin[§] vs. pioglitazone	None	100-200	40
Wainstein 2012[98] NCT00532935	T2DM (*n*=517)	3.2±4.0/3.3±3.5	9.0±1.3/8.9±1.3	NR	52.4/52.2	54.8/52.3	Phase III, double-blind, parallel-group, RCT	2	Sitagliptin/metformin[§] vs. pioglitazone	None	100-200	32
Russel-Jones 2012[99] NCT00676338	T2DM (*n*=572)	2.7±3.7/2.7±3.7	8.5±1.3/8.6±1.2	NR	52.3/54.5	56.0/59.6	Phase III, double-blind, parallel-group, RCT, in 106 centres in 22 countries, DURATION-4 trial	3	Sitagliptin vs. metformin + pioglitazone	None	100	36

Raz 2006[100] NCT00094757	T2DM (*n*=521)	4.5±4.1/4.7±5.0	8.1±0.9/8.1±0.9	NR	55.0/55.5	52.1/62.7	Double-blind, parallel-group, RCT, multinational	3	Sitagliptin vs. placebo + pioglitazone	None	100-200	54
NCT01076075[101]	T2DM (*n*=422)	NR	8.4±0.8/8.4±0.9	NR	54.4/55.4	45.2/46.2	Phase III, parallel-group, RCT, in 161 centres in 23 countries	2	Sitagliptin vs. placebo + pioglitazone	Metformin + sulfonylurea	100	54
Hermansen 2007[102] NCT00106704	T2DM (*n*=441)	8.3±5.3/9.3±6.7	8.3±0.7/8.3±0.7	NR	55.6/56.5	52.7/53.4	Double-blind, crossover RCT, multinational	2	Sitagliptin vs. placebo + pioglitazone	± Metformin + glimepiride	100	24
Yoon 2012[103] NCT01028391	T2DM (*n*=317)	2.6±4.0/9.4±1.1	1.6±3.7/9.4±1.4	NR	51.4/52.3	52.4/58.8	Double-blind, parallel-group, RCT, in 28 centres in 15 countries	2	Sitagliptin vs. pioglitazone	Pioglitazone	100	54
Yoon 2011[103] NCT00397631	T2DM (*n*=317)	2.6±4.0/1.6±3.7	7.0±1.2/7.6±1.2	NR	50.2/51.6	52.5/56.0	Phase III, double-blind, parallel-group, RCT	2	Sitagliptin + pioglitazone vs. pioglitazone	NR	100	54
Bergenstal 2010[104] NCT00637273	T2DM (*n*=497)	5.0±4.0/6.0±5.0	8·5±1·2/8·5±1·1	NR	52.7/52.2	51.8/51.8	Phase III, double-blind, parallel-group, RCT, in 62 centres in USA, Mexico and India, DURATION-2 trial	2	sitagliptin vs. pioglitazone	Metformin	100	26
NCT00722371[105]	T2DM (*n*=1615)	NR	NR	NR	57.0/57.3	56.8/56.0	Phase III, double-blind, parallel-group, RCT	7	Sitagliptin vs. pioglitazone	Metformin + sulfonylurea	100	54
Chan 2008[106] NCT00095056	T2DM with CKD (*n*=91)	3.6±9.7/13.2±8.9	7.6±0.9/7.8±0.9	NR	68.9/65.3	47.7/61.5	Phase III, double-blind, parallel-group, RCT, in 69 centres in 11 countries	2	Sitagliptin vs. placebo + glipizide	None	25	54
Charbonnel 2006[107] NCT00086515	T2DM (*n*=701)	NR	7.96±0.8/8.0±0.8	NR	54.4/54.7	55.8/59.5	Double-blind, parallel-group, RCT	2	Sitagliptin vs. placebo + glipizide	Metformin	100	24
Ahrén 2014[108] NCT00838903	T2DM (*n*=710)	5.8±4.8/6.7±6.6	8.1±0.8/8.2±0.9	NR	54.3/56.1	46.0/49.5	Phase III, double-blind, parallel-group, RCT, in 289 centres in 10 countries, HARMONY 3 trial	3	Sitagliptin vs. placebo + glimepiride	Metformin	100	104
Arjona Ferreira 2013[109] NCT00509236	T2DM with CKD on dialysis (*n*=129)	19±6.0/16±6.0	7.9±0.7/7.8±0.7	NR	60.5/58.5	62.5/56.9	Phase III, double-blind, parallel-group, RCT	2	Sitagliptin vs. glipizide	NR	25	54
Ferreira 2013[110] NCT00509262	T2DM with CKD (*n*=422)	10.7±7.5/10.1±7.8	7.8±0.7/7.8±0.7	NR	64.2/64.2	62.1/57.5	Phase III, double-blind, parallel-group, RCT	2	Sitagliptin vs. glipizide	NR	25-50	54
Seck 2010[111] NCT00094770	T2DM (*n*=1172)	5.8±5.7/5.7±4.9	7.3±0.6/7.3±0.7	NR	56.8/56.6	57.1/61.3	Double-blind, parallel-group, RCT	2	Sitagliptin vs. glipizide	Metformin	100	52

Arechavaleta 2011[112] NCT00701090	T2DM (*n*=1034)	6.8±4.6/6.7±4.8	7.5±0.7/7.5±0.8	NR	56.3/56.2	55.0/53.8	Phase III, double-blind, parallel-group, RCT	2	Sitagliptin vs. glimepiride	Metformin	100	30
NCT01131182[113]	T2DM (*n*=1021)	NR	NR	NR	55.0/55.0	53.1/49.6	Open-label, parallel-group, RCT	2	Sitagliptin vs. sulfonylurea	Metformin	100	4
Terauchi 2017[114] NCT01183104	T2DM (*n*=291)	NR	7.5±0.7/7.5±0.7	NR	70.2/70.8	51.7/61.2	Open-label, RCT, parallel-group, in Japan, START-J trial	2	Sitagliptin vs. glimepiride	None ± α-glucosidase-Is ± biguanide	50	52
Leiter 2014[115] NCT01098539	T2DM with CKD (*n*=495)	11.6±8.5/10.8±7.4	8.2±0.9/8.1±1.0	NR	63.2/63.5	54.6/52.8	Phase III, double-blind, parallel-group, RCT, Harmony 8 trial	2	Sitagliptin vs. albiglutide	± AHG agents	Sitagliptin 30-50 Albiglutide 30-50	52
Pratley 2011[116] NCT00700817	T2DM (*n*=658)	6.3±5.4/6.2±4.9	NR	NR	55.0/55.5	54.8/52.1	Phase III, double-blind, parallel-group, RCT, in 166 centres in 13 countries	3	Sitagliptin vs. liraglutide	Metformin	Sitagliptin 100 Liraglutide 1.2-1.8	78
Zang 2016[117] NCT02008682	T2DM (*n*=367)	5.2±5.4/5.3±4.4	8.1±0.8/8.1±0.8	NR	51.4/51.7	63.6/55.7	Phase IV, parallel-group, open-label RCT, in 10 centres in China	2	Sitagliptin vs. liraglutide	Metformin	Sitagliptin 100 Liraglutide 1.8	26
NCT01519674[118]	T2DM (*n*=575)	NR	8.4±0.8/8.4±0.8	NR	56.0/54.8	48.9/57.2	Phase IV, open-label, parallel-group, RCT, in 69 centres in 10 countries	3	Sitagliptin + biphasic insulin aspart vs. biphasic insulin aspart	Metformin	100-200	24
Philis-Tsimikas 2013[119] NCT01046110	T2DM (*n*=454)	7.7±5.9/7.8±6.2	9.0±1.0/8.8±1.0	NR	54.9/56.4	54.5/62.7	Phase III, open-label, parallel-group, RCT, in 93 centres in 7 countries	2	Sitagliptin vs. insulin degludec	Metformin, sulfonylurea, glindes, pioglitazone	100	26
NCT00875394[120]	T2DM (*n*=68)	8.9±7.1/6.8±8.5	8.8±1.5/8.0±1.8	NR	54.2/54.7	30.6/25.4	Phase III, open-label, parallel-group, RCT	3	Sitagliptin vs. any AHGs agents other DPP4-Is	Metformin	100	24
**Vildagliptin therapy**												
NCT00646542[121]	T2DM with CKD (*n*=519)	NR	NR	NR	NR	NR	Phase III, double-blind, parallel-group, RCT, in 12 countries	2	Vildagliptin vs. placebo	None	50	52
NCT00821977[122]	T2DM (*n*=451)	NR	NR	NR	NR	NR	Phase II/III, double-blind, parallel-group, RCT, in 176 centres in 8 countries	3	Vildagliptin vs. placebo	None	NR	76
Scherbaum 2008[123] NCT00300287	T2DM (*n*=306)	2.1±2.1/2.5±2.6	6.6±0.4/6.7±0.4	NR	63.3/62.8	59.6/59.3	Phase III, double-blind, parallel-group, RCT	2	Vildagliptin vs. placebo	None	50	52

Dejager 2007[124] NCT00099905	T2DM (*n*=632)	NR	NR	NR	53.0/53.0	NR	Phase III, double-blind, parallel-group, RCT, in USA	4	Vildagliptin vs. placebo	None	50, 100	24
McMurray 2013[125] NCT00894868	T2DM with CHF class I-III (*n*=254)	9.5±8.1/9.1±7.8	7.8±0.9/7.8±1.1	Stroke 12 (9.4%) vs. 11 (8.7%)	63.0/63.4	76.0/70.0	Phase IV, double-blind, parallel-group, RCT in 94 centres in 15 countries, VIVIDD trial	2	Vildagliptin vs. placebo	None	100	52
Pan 2012[126] NCT00822211	T2DM (*n*=438)	4.9±4.6/5.2±4.6	8.1±0.8/8.0±0.8	NR	53.9/54.5	27.0/18.0	Double-blind, parallel-group, RCT, in China	3	Vildagliptin vs. placebo	Metformin	50, 100	24
CLAF237ADE02[127]	T2DM (*n*=402)	NR	NR	NR	61.0/61.0	59.4/53.1	Double-blind, parallel-group, RCT	2	Vildagliptin vs. placebo	Metformin	50, 100	24
Bosi 2007[128] NCT00099892	T2DM (*n*=541)	6.3±5.1/8.4±1.0	6.2±5.3/8.3±0.9	NR	54.1/54.5	59.4/53.1	Phase III, double-blind, parallel-group, RCT, 109 centres in 4 countries	3	Vildagliptin vs. placebo	Metformin	50, 100	24
Yang 2015[129] NCT01357252	T2DM (*n*=278)	6.9±4.6/7.0±10.0	6.9±4.1/7.2±11.0	NR	58.3/58.7	55.2/58.1	Double-blind, parallel-group, RCT, in China	2	Vildagliptin vs. placebo	Sulfonylurea	50	24
Garber 2008[130] NCT00099944	T2DM (*n*=515)	6.8±5.3/7.8±5.8	8.5±0.9/8.5±1.0	NR	58.4/57.9	59.5/58.3	Phase III, double-blind, parallel-group, RCT, in 12 countries	3	Vildagliptin vs. placebo	Sulfonylurea	50-100	24
Fonseca 2009[131] NCT00099931	T2DM (*n*=296)	14.4±8.6/14.9±8.4	8.4±1.0/8.4±1.1	NR	59.6/58.9	47.9/54.6	Phase III, double-blind, parallel-group, RCT, in 68 centres 10 in Germany, 5 Finland, 4 Spain and 49 USA	2	Vildagliptin vs. placebo	Insulin	100	24
Bosi 2009[132] NCT00468039	T2DM (*n*=1171)	23.7±35.2/6.3±39.9	8.7±1.0/8.6±0.9	NR	44.4/52.4	58.4/58.2	Phase III, open-label trial, in 250 centres in USA, Canada and India	4	Vildagliptin vs. placebo + metformin	None	50-100	24
Schweizer 2007[133] NCT00099866	T2DM (*n*=760)	1.1±3.5/1.0±3.3**	8.7±1.1/8.7±1.1	NR	52.8/53.6	52.9/57.5	Phase III, double-blind, parallel-group, RCT, in 183 centres in 10 countries, CLAF237A2309E1 trial	2	Vildagliptin vs. metformin	None	100	104
Pan 2008[134] NCT00110240	T2DM (*n*=660)	1.2±2.4/1.3±2.4	8.6±0.9/8.6±1.0	NR	51.8/51.9	60.1/63.2	Phase III, double-blind, parallel-group, RCT, in 31 centres in China, Romania and Spain	2	Vildagliptin vs. α-glucosidase-Is (acarbose)	None	100	24
Rosenstock 2009[135] NCT00138619	T2DM (*n*=591)	2.3±3.4/2.7±4.2	8.7±1.1/8.7±1.1	NR	54.5/54.2	57.5/57.6	Phase III, double-blind, parallel-group, RCT, in 202 centres in 11 countries in USA and Europe	2	Vildagliptin vs. rosiglitazone	None	100	104

Bolli 2009[136] NCT00237237	T2DM (*n*=575)	6.4±4.9/6.4±5.2	8.4±1.0/8.4±0.9	NR	56.3/57.0	61.7/64.1	Double-blind, parallel-group, RCT, in USA and Germany	2	Vildagliptin vs. pioglitazone	Metformin	100	52
Foley 2009[137] NCT00102388	T2DM (*n*=1092)	NR	NR	NR	55.2/54.3	58.8/52.7	Double-blind, parallel-group, RCT	2	Vildagliptin vs. gliclazide	None	100	104
Ferrannini 2009[138] NCT00106340	T2DM (*n*=2772)	5.7±5.2/5.8±5.0	7.3±0.7/7.3±0.7	NR	57.5/57.5	52.8/54.1	Double-blind, parallel-group, RCT, in USA and Germany	2	Vildagliptin vs. glimepiride	Metformin	100	52

Abbreviations: *ACS*, acute coronary syndrome; *ACE*, angiotensin-converting enzyme inhibitor; *ARB*, angiotensin receptors blocker; *AHG*, anti-hyperglycaemic drugs; *CVA*, cardiovascular accident; *CVD*, cardiovascular disease; *CVD*, cerebrovascular disease; *CKD*, chronic kidney disease; C*RF*, chronic renal failure; *CHF*, congestive heart failure; *IGT*, impaired glucose tolerance; *mµ/d*, microgram pear day; *mg/d*, milligram pear day; NR, not report; *RCT*, randomized controlled trial; *TZDs*, thiazolidinedione; *T2DM*, type 2 diabetes mellitus.

Note: ‡ Mean age and dosages present for intervention drugs; ** Median age; **Median year; **Median (interquartile range); ¶Novel treatment of fixed-dose combination of 2 different classes of AHG; †values are expressed as mean ± SD or ⁱmean ± SE.

a 25 mg in patients with an estimated glomerular filtration rate (eGFR) of ≥60 ml/min/1.73 m^2 of body surface area; 12.5 mg with an eGFR of 30 to<60 ml/min/1.73 m^2; and 6.25 mg with an eGFR of<30 ml/min/1.73 m^2.

b or 50 mg daily if the baseline eGFR was ≥30 and<50 ml/min/1.73 m^2.

c 2.5 mg daily in patients with an eGFR ≤50 ml/min/1.73 m^2.

d <30 ml/min/1.73 m^2 not on chronic dialysis.

e Degree of renal impairment: moderate (CrCl ≥30 and <50 ml⁄min), severe (CrCl <30 ml⁄min and not receiving dialysis) or end-stage renal disease (ESRD) on hemodialysis.

GLP1-RAs

Table S2. Trial baseline characteristic of GLP1-RAs

Study<br>Trial registry	Population<br>Sample size	T2DM† Mean<br>duration (years)	HbA$_{1C}$ levels %	Prior stroke /<br>TIA (n., %)	Mean age<br>(years)‡	Males (%)	Study design and country	Parallel<br>arms	GLP1-RAs vs. comparators	Background<br>AHGs therapy	GLP1-RAs dosage‡	Duration<br>(weeks)
**Albiglutide therapy**												
Nauck 2016[139]<br>NCT00849017	T2DM (*n*=301)	3.8±3.9/4.3±4.0	8.1±0.9/8.0±0.9	NR	52.8/53.1	53.9/57.4	Phase III, open-label, parallel-group, RCT, in 262 centres in 3 countries	3	Albiglutide vs. placebo	None	30-50 mg/week	156
Hernandez 2018[140]<br>NCT02465515	T2DM and CVD (*n*=9463)	14.1±8.6/7.9±6.1	8.7±1.5/8.7±1.5	Stroke=827 (17%) vs. 854 (18%)	64.1/64.2	70.0/69.0	Phase III, double-blind, parallel-group, RCT, in 610 centres in 28 countries, HARMONY Outcomes trial	2	Albiglutide vs. placebo	AHG agents	30-50 mg/week	84[**]
Reusch 2014[141]<br>NCT00849056	T2DM (*n*=301)	8.0±5.6/7.9±6.1	8.1±1.0/8.1±0.9	NR	55.2/54.9	61.3/58.3	Phase III, double-blind, parallel-group, RCT, in 331 centres in 6 countries, HARMONY 1	2	Albiglutide vs. placebo	Pioglitazone ± metformin	30 mg/week	156
Nauck 2016[139]<br>NCT00839527	T2DM (*n*=301)	3.8±4.2/4.3±4.0	8.1±0.8/8.0±0.9	NR	52.8/53.1	53.9/57.4	Phase III, double-blind, parallel-group, RCT, in 143 centres USA and Mexico, HARMONY 2	3	Albiglutide vs. placebo	Metformin + insulin preferred	30-50 mg/week	156
Seino 2014[142]<br>NCT01098461	T2DM (*n*=301)	7.1±5.2/6.7±7.8	< or ≥ 8.4/< or ≥ 8.4	NR	56.8/57.5	69.7/69.8	Phase II, double-blind, parallel-group, RCT, in 30 centres in Japan	4	Albiglutide vs. placebo	Insulin	30-50 mg/week	24
Ahrén 2017[108]<br>NCT00838903	T2DM (*n*=710)	6.0±4.3/6.7±6.6	8.1±0.8/8.2±0.9	NR	54.3/56.1	44.7/49.5	Phase III, double-blind RCT, HARMONY-3	3	Albiglutide vs. placebo + glimepiride	± AHG agents	30 mg/week	156
Home 2014[143]<br>NCT00839527	T2DM (*n*=663)	8.5±6.3/9.2±6.1	8.2±0.9/8.2±0.9	NR	54.5/55.7	49.8/57.2	Phase III, double-blind, parallel-group, RCT, in 234 centres in 9 countries, HARMONY 5	3	Albiglutide vs. placebo + pioglitazone	Metformin + glimepiride	30 mg/week	156
Pratley 2014[144]<br>NCT01128894	T2DM (*n*=812)	8·4±6·1/8·3±5·6	8·2±0·9/15±0·8	NR	55.4/55.8	47.3/53.4	Phase III, open-label, parallel-group, RCT, in 174 centres in 8 countries, HARMONY 7	2	Albiglutide vs. liraglutide	Metformin ± sulfonylurea ± TZDs	Albiglutide 50 mg/d<br>Liraglutide 1.8 mg/d	32

Weissman 2014[145] NCT00838916	T2DM (*n*=745)	8.9±6.5/8.4±5.7	8.3±0.9/8.4±0.9	NR	55.8/54.7	56.7/54.8	Phase III, open-label, parallel-group, RCT, in 338 centres in 4 countries, HARMONY 4	2	Albiglutide vs. insulin glargine	Metformin ± sulfonylurea	30 mg/week	156
Rosenstock 2014[146] NCT00976391	T2DM (*n*=566)	11.0±7.0/11.0±6.0	8.5±0.9/8.4±0.9	NR	54.8/56.3	46.0/48.0	Phase III, open-label, parallel-group, RCT, HARMONY 6	2	Albiglutide vs. insulin lispro	Metformin ± pioglitazone ± α-glucosidase-Is	50 mg/d	52
**Dulaglutide therapy**												
Gerstein 2019[147] NCT01394952	T2DM with CVD (*n*=9622)	9.5 (5.5-14.5)/9.5 (5.5-14.5)	7.3±1.1/7.4±1.1	NR	66.2/66.2	53.4/53.9	Phase III, double-blind, RCT, in 320 centres in 26 countries, REWIND trial	2	Dulaglitude vs. placebo	NR	1.5 mg/d	339
Wysham 2014[148] NCT01064687	T2DM (*n*=700)	9.0±6.0/9.0±6.0	8.1±1.3/8.1±1.3	NR	54.6/56.0	58.8/59.2	Phase III, double-blind, parallel-group, RCT, in 89 centres in 4 countries, AWARD-1 trial	2	Dulaglutide vs. placebo	Metformin + pioglitazone	0.75-1.5mg/d	52
Pozzilli 2017[149] NCT02152371	T2DM (*n*=300)	13.0±7.5/13.3±7.7	8.4±0.9/8.3±0.8	NR	60.2/60.6	56.6/58.6	Phase III, double-blind, parallel-group, RCT, in 40 centres in 7 countries, AWARD-9 trial	2	Dulaglutide vs. placebo	Basal insulin glargine ± metformin	1.5 mg/d	28
**Exenatide therapy**												
Holman 2017[150] NCT01144338	T2DM ± CVD (*n*=14752)	12.0(7.0-18.0)/12.0(7.0-18.0)**	8.0(7.3-8.9)/8.0(7.3-8.9)**	NR	63.0/63.0**	62.0/62.0	Double-blind, parallel-group, RCT, in 687 centres in 35 countries. EXSCEL trial	2	Exenatide vs. placebo	Metformin ± sulfonylurea ± DPP4-Is	2 mg/week	167**
Gao 2009[151] NCT00324363	T2DM (*n*=466)	8.0±6.0/8.0±5.0	8.3±1.0/8.3±1.0	NR	55.0/54.0	48.0/41.0	Double-blind, parallel-group, RCT, in 4 countries	2	Exenatide vs. placebo	Metformin ± sulfonylurea	10-20 µg/d	16
Gill 2010[152] NCT00516074	T2DM (*n*=54)	7.0±4.0/6.0±4.0	7.5±0.9/7.1±0.7	NR	57.0/54.0	68.0/42.0	Double-blind, parallel-group RCT, in Canada and Netherlands	2	Exenatide vs. placebo	Metformin ± TZDs	5-10 µg/d	12
NCT00603239[153]	T2DM (*n*=165)	NR	NR	NR	54.9/54.1	60.3/57.4	Phase III, double-blind, parallel-group, RCT, in 25 centres in 5 countries	2	Exenatide vs. placebo	Metformin + TZDs	10-20 µg/week	26
Wysham 2014[148] NCT01064687	T2DM (*n*=417)	9.0±6.0/9.0±6.0	8.1±1.3/8.1±1.3	NR	55.5/56.0	56.5/59.2	Phase III, double-blind, parallel-group, RCT, in 89 centres in 4 countries, AWARD-1 trial	2	Exenatide vs. placebo	Metformin + pioglitazone	10-20 µg/d	52

Gadde 2017[82] NCT01652729	T2DM (*n*=242)	8.5±6.3/8.7±5.8	8.4±0.9/8.5±1.0	NR	53.4/54.3	49.2/54.1	Phase III, open-label, parallel-group, RCT, in 60 centres in USA, DURATION-NEO-2 trial	2	Exenatide vs. placebo	Metformin	2 mg/d	28
Gallwitz 2012[154] NCT00359762	T2DM (*n*=1335)	5·8±4·8/5·5±4·3	7·5±0·7/7·4±0·7	NR	56.1/56.8	55.5/51.7	Phase III, open-label, parallel-group, RCT, in 128 centres in 14 countries, EUREXA trial	4	Exenatide vs. glimepiride	Metformin	10-50 µg/d	235
Buse 2013[155] NCT01029886	T2DM (*n*=911)	8.0±6.0/9.0±6.0	8·5±1·0/8·4±1·0	NR	56.6/56.7	55.1/54.4	Phase III, open-label, parallel-group, RCT, in 110 centres in 21 countries, DURATION-6	2	Exenatide vs. liraglutide	Metformin ± sulfonylurea ± pioglitazone	Exenatide 2 mg/week Liraglutide 1.8 mg/d	26
Russel-Jones 2012[99] NCT00676338	T2DM (*n*=657)	2.7±3.2/2.7±3.7	8.5±1.2/8.6±1.2	NR	53.7/54.5	56.0/61.1	Phase III, double-blind, parallel-group, RCT, in 106 centres in 22 countries, DURATION-4 trial	3	Exenatide vs. metformin + pioglitazone	None	2 mg/week	36
Bergenstal 2010[104] NCT00637273	T2DM (*n*=497)	6.0±5.0/6.0±5.0	8·6±1·2/8·5±1·1	NR	52.7/52.2	51.8/51.8	Phase III, double-blind, parallel-group, RCT, in 62 centres in USA, Mexico and India, DURATION-2 trial	2	Exenatide vs. pioglitazone	Metformin	100 mg/d	26
Nauck 2007[156] NCT00082407	T2DM (*n*=501)	9.8±6.3/10.0±6.2	8.6±1.0/8.6±1.1	NR	58.8/58.5	46.6/50.8	Phase III, open-label, parallel-group RCT, in 69 centres in 12 countries	2	Exenatide vs. insulin aspart	Metformin ± sulfonylurea	5-10 µg/d	52
Inagaki 2012[157] NCT00935532	T2DM (*n*=427)	8.9±6.1/9.2±5.9	8.5±0.8/8.5±0.8	NR	57.1/56.4	66.0/69.8	Phase III, open-label, parallel-group, RCT, in 22 centres in Japan	2	Exenatide vs. insulin glargine	Biguanide ± sulfonylurea ± TZDs	2 mg/week	26
**Liraglutide therapy**												
Marso 2016[158] NCT01179048	T2DM with high risk CVD and/or CKD (*n*=9340)	12.7±8.0/8.7±1.5	12.9±8.1/8.7±1.5	Stroke/TIA 730 (15.6%) vs. 777 (16.6%)	64.2/64.4	64.5/64.0	Double-blind, parallel-group, RCT, in 410 centres in 32 countries, LEADER trial	2	Liraglutide vs. placebo	≤2 AHG agents + insulin	0.6-1.8 mg/d	198**
Seino 2016[159] NCT01572740	T2DM (*n*=257)	14.3±8.9/14.7±8.6	8.8±0.9/8.8±0.9	NR	61.3/59.8	54.3/57.7	Phase III, double-blind, parallel-group, RCT, in 23 centres in Japan	2	Liraglutide vs. placebo	Insulin	0.3, 0.6, 0.9 mg/d	36
Kaku 2011[160] NCT00393718	T2DM (*n*=400)	8.1±6.7/8.5±6.8	9.3±1.1/9.2±0.9	NR	58.2/58.5	68.3/65.2	Phase III, double-blind, parallel-group, RCT, in Japan	2	Liraglutide vs. glibenclamide	NR	0.9 mg/d	52

Bailey 2016[161] NCT01907854	T2DM (*n*=406)	7.9±5.7/7.6±6.2	8.3±0.6/8.2±0.6	NR	56.3/56.5	57.9/61.3	Phase IV, double-blind, parallel-group, RCT, in 106 centres in 7 countries, LIRA-SWITCH trial	2	Liraglutide vs. sitagliptin	Metformin + sitagliptin	Liraglutide 0.6-1.8 mg/d Sitagliptin 100 mg/d	26
Lingvay 2016[162] NCT01952145	T2DM (*n*=557)	11.6±7.4/11.3±6.6	8.4±0.9/8.2±0.9	NR	58.4/59.1	51.4/49.1	Phase III, double-blind, parallel-group, RCT, in 91 centres in 10 countries, DUAL-V trial	2	Liraglutide/insulin degludec vs. insulin glargine	Metformin	0.6 mg/d/16 units IDeg	27
NCT01117350[163]	T2DM (*n*=1125)	8.4(4.8-11.7)/8.5(5.2-12.4)**	9.1±1.1/9.0±1.1	NR	57.4/57.1	55.9/52.7	Phase IV, open-label, parallel-group, RCT, in 136 centres in 17 countries, EAGLE trial	2	Liraglutide vs. insulin glargine	Metformin	0.6, 1.2, 1.8 mg/d	48
**Lixisenatide therapy**												
Pfeffer 2015[164] NCT01147250	T2DM with recent ACS (*n*=6068)	9.2±8.2/9.4±8.3	7.7±1.3/7.6±1.3	Stroke 143 (4.7%) vs. 188 (6.2%)	59.9/60.6	69.6/69.1	Phase III, double -blind, parallel-group, RCT, 49 countries, ELIXA trial	2	Lixisenatide vs. placebo	AHG agents	10-20 mg/week	109
**Semaglutide therapy**												
Marso 2016[165] NCT01720446	T2DM with established CVD and/or CKD (*n*=3297)	13.9±8.1/8.7±1.5	8.7±1.4/8.7±1.5	IS=178 (10.8%) vs. 205 (12.3%) HS=52 (4.9%) vs. 56 (3.4%)	64.7/64.6	61.5/60.0	Double-blind, parallel-group, RCT, in 230 centres in 20 countries, SUSTAIN-6 trial	4	Semaglutide vs. placebo	≤2 AHG agents	0.5-1 mg/week	104
Husain 2019[166] NCT02692716	T2DM with established CVD and/or CKD (*n*=3183)	14.7±8.5/15.1±8.5	8.2±1.6/8.2±1.6	NR	66.0/66.0	61.5/60.0	Double-blind, parallel-group, RCT, in 214 centres in 21 countries, PIONEER-6 trial	4	Semaglutide vs. placebo	AHG agents	14 mg/d	69**
**Taspoglutide therapy**												
Raz 2012[167] NCT00744926	T2DM (*n*=368)	2.5±2.7/2.3±1.9	7.6±1.0/7.6±1.0	NR	54.2/55.8	36.5/37.0	Phase III, double-blind, parallel-group, RCT, 53 countries, T-emerge 1 trial	3	Taspoglutide vs. placebo	None	10-20 mg/week	24
NCT01018173[168]	T2DM with established CVD (*n*=2118)	NR	NR	NR	63.3/63.3	65.0/65.0	Double-blind, parallel-group RCT, in 288 centres in 25 countries, T-EMERGE-8 trial	2	Taspoglutide vs. placebo	NR	10-20 mg/week	104

Bergenstal 2012[81] NCT00754988	T2DM (*n*=469)	5.9±4.8/5.5±3.9	7.9±0.9/8.0±0.8	NR	54.2/56.1	55.3/52.2	Phase III, double-blind, parallel-group RCT in 149 centres in 23 countries, T-Emerge 4 trials	2	Taspoglutide vs. placepo	Metformin	10-20 mg/week	156
Hollander 2012[169] NCT00823992	T2DM obese (*n*=304)	5.2±4.3/4.9±4.1	7.5±40.8/7.6±0.8	NR	53.0/54.0	42.0/39.0	Phase III, double-blind 24 weeks, 28 week open-label extension phase, parallel-group RCT, in 63 centres in 8 countries, T-Emerge 7 trial	2	Taspoglutide vs. placebo	Metformin ± sulfonylurea	20 mg/week	52
Henry 2012[170] NCT00744367	T2DM (*n*=324)	7.8±4.9/7.5±5.8	8.2±1.0/8.1±0.9	NR	54.0/54.3	56.0/50.0	Phase III, double-blind 24 weeks, 28 week open-label extension phase, parallel-group RCT in 113 centres in 8 countries T-Emerge 3 trial	3	Taspoglutide vs. placebo	Metformin + pioglitazone	10-20 mg/week	24
Pratley 2012[171] NCT00909597	T2DM (*n*=751)	8.9±6.3/8.6±6.1	8.3±0.9/8.3±0.8	NR	56.4/57.1	47.5/51.0	Double-blind, parallel-group RCT in 130 centres in 17 countries, T-Emerge 6 trial	3	Taspoglutide vs. pioglitazone	Sulfonylurea ± metformin	10-20 mg/week	24
Rosenstock[172] 2013 NCT00717457	T2DM (*n*=1173)	6.6±5.5/6.5±5.4	8.1±0.9/8.1±0.9	NR	56.0/55.0	55.0/49.0	Phase III, open-label, parallel-group, RCT, in 189 centres in 23 countries, T-emerge 2 trial	3	Taspoglutide vs. exenatide	Metformin ± TZDs	10-20 md/week	104
Nauck 2013[173] NCT00755287	T2DM (*n*=1037)	9.0±6.0/10.0±6.0	5.5±10.0/5.6±10.0	NR	58.0/NR	53.0/NR	Phase III, open-label 24 weeks, 28 weeks single blind extension phase, parallel-group RCT in 187 centres in 25 countries, T-Emerge 4 trials	3	Taspoglutide vs. insulin glargine	Metformin + sulfonylurea	10-20 mg/week	24

Abbreviations*: ACS*, acute coronary syndrome; *ACE*, angiotensin-converting enzyme inhibitor; *ARB*, angiotensin receptors blocker; *AHG*, anti-hyperglycaemic drugs; *CVD*, cardiovascular disease; *CKD*, chronic kidney disease; *CRF*, chronic renal failure; *CHF*, congestive heart failure; *IGT*, impaired glucose tolerance; *IS*, ischaemic stroke; *HS*, haemorrhagic stroke; *mµ/d*, microgram pear day; *mg/d*, milligram pear day; *NR*, not report; *RCT*, randomized controlled trial; *TZDs*, thiazolidinedione; *T2DM*, type 2 diabetes mellitus.

Note: ‡ Mean age and dosages present for intervention drugs; ** Median age; **Median year; **Median (interquartile range); ¶Novel treatment of fixed-dose combination of 2 different classes of AHG; †values are expressed as mean ± SD or ˡmean ± SE.

SGLT2-Is

Table S3. Trial baseline characteristic of SGLT2-Is

Study<br>Trial registry	Population<br>Sample size	T2DM† Mean duration (years)	HbA_{1C} levels %	Prior stroke / TIA (n., %)	Mean age (years)‡	Males (%)	Study design and country	Parallel arms	Intervention vs. comparator	Background AHGs therapy	SGLT2-Is dosage‡ mg/day	Duration (weeks)
**Canagliflozin therapy**												
Inagaki 2013[174]<br>NCT01022112	T2DM (*n*=382)	NR	8.1±0.8/7.9±0.8	NR	57.3/57.7	67.4/72.0	Phase II, double-blind, parallel-group RCT, in 6 centres in Japan	5	Canagliflozin vs. placebo	None	50, 100, 200, 300	12
Inagaki 2014[175]<br>NCT01413204	T2DM (*n*=272)	5.3±5.3/5.6±5.8	8.0±0.8/8.0±0.7	NR	57.9/58.2	73.8/64.5	Phase III, double-blind, parallel-group RCT, in 31 centres in Japan	3	Canagliflozin vs. placebo	None	100-200	24
Stenlöf 2012[176]<br>NCT01081834	T2DM (*n*=584)	4.4±4.5/4.2±4.1	8.0±1.0/8.0±1.0	NR	55.0/55.7	44.7/45.8	Phase III, double-blind, parallel-group RCT, in 79 centres in 18 countries	3	Canagliflozin vs. placebo	None	100-200	26
Weir 2014[177]<br>NCT01064414	T2DM with CKD (*n*=436)	NR	NR	NR	68.7/68.2	59.2/63.3	Phase III, double-blind, parallel-group RCT, in 109 centres in 18 countries	3	Canagliflozin vs. placebo	None	100-200	52
Sha 2014[178]<br>NCT01483781	T2DM (*n*=35)	8.6±4.0/8.4±4.6	7.6±0.5/7.7±0.6	NR	63.3/62.3	89.0/83.0	Phase I, double-blind, parallel-group RCT, in Germany	2	Canagliflozin vs. placebo	Metformin	300	12
Qiu 2014[179]<br>NCT01340664	T2DM (*n*=279)	7.05±.5/7.0±6.4	7.6±0.9/7.7±0.9	NR	57.7/57.0	45.2/49.5	Phase II, double-blind, parallel-group RCT, in 64 centres in 7 countries	3	Canagliflozin vs. placebo	Metformin	50-150	18
Rosenstock 2012[83]<br>NCT00642278	T2DM (*n*=386)	5.9±5.0/6.4±5.0	7.7±0.9/7.8±0.8	NR	53.1/52.5	51.0/53.1	Phase II, double-blind, parallel-group RCT, in 85 centres in 13 countries	6	Canagliflozin vs. placebo	Metformin	50, 100, 200, 300, 600	12
Ji 2015[180]<br>NCT01381900	T2DM (*n*=676)	6.8±4.7/6.4±4.6	8.0±0.9/7.9±0.9	NR	56.5/55.8	52.7/55.3	Phase III, double-blind, parallel-group RCT, in 25 centres in China, Malaysia and Vietnam	3	Canagliflozin vs. placebo	Metformin ± sulfonylurea	100-300	18
Wilding 2013[181]<br>NCT01106625	T2DM (*n*=469)	9.2±5.1/10.3±6.7	8.1±0.9/8.1±0.9	NR	56.7/56.7	52.1/48.7	Phase III, double-blind, parallel-group RCT, in 76 centres in 12 countries, CANTATA-MSU Trial	3	Canagliflozin vs. placebo	Metformin + sulfonylurea	100-300	52
Neal 2017[182]<br>NCT01032629<br>NCT01989754	T2DM with CVD (*n*=10,142)	13.5±7.7/13.7±7.8	8.2±0.9/8.2±0.9	CVD=1113 (19.2) vs. 845 (19.4)	63.2/63.4	64.9/63.3	Phase IV, double-blind, parallel-group, RCT, in 667 centres in 30 countries, CANVAS Trial	2	Canagliflozin vs. placebo	Sulfonylurea	100-300	188.2

Fulcher 2015[183] NCT01032629	T2DM at risk of CVD (*n*=127)	9.5±6.1/11.4±6.7	8.2±0.9/8.5±1.1	NR	64.8/64.8	56.0/58.0	Double-blind, parallel-group, RCT, CANVAS trial	3	Canagliflozin vs. placebo	Sulfonylurea	100-300	72-84
Cai 2018[184] NCT02025907	T2DM (*n*=216)	9.6±6.1/6.9±5.3	8.2±0.9/7.9±0.9	NR	57.4/57.5	61.7/51.9	Phase IV, double-blind, parallel-group, RCT, in 57 centres in 5 countries	2	Canagliflozin vs. placebo	Metformin + sitagliptin	100-300	28
Perkovic 2019[185] NCT02065791	T2DM with CKD[a] (*n*=4,401)	15.5±8.7/16.0±8.6	8.3±1.3/8.3±1.3	CVD 342 (15.5) vs. 358 (16.3)	62.9/63.2	65.4/66.7	Phase IV, double-blind, parallel-group, RCT, in 690 centres in 34 countries, CREDENCE Trial	2	Canagliflozin vs. placebo	Metformin ± sulfonylurea ± DPP4-Is ± GLP1-RAs ± insulin	100	136.6
Bode 2015[186] NCT01106651	T2DM (*n*=312)	NR	NR	NR	63.9/63.2	53.1/60.3	Phase III, double-blind, parallel-group RCT, in 90 centres in 17 countries	3	Canagliflozin vs. placebo	± AHG agents	100-300	104
Lavalle-González 2013[187] NCT01106677	T2DM (*n*=549)	6.9±5.4/6.8±5.3/6.9±5.4	7.9±0.9/8.0±0.9/7.9±0.9	NR	55.4/46.2	55.3/51.4	Phase III, double-blind, parallel-group, RCT, in 128 centres in 22 countries	4	Canagliflozin vs. placebo + sitagliptin	Metformin	Canagliflozin 100-300 Sitagliptin 100	24
Forst 2014[188] NCT01106690	T2DM (*n*=342)	10.8±7.1/10.1±6.6	8.0±0.9/8.0±1.0	NR	56.9/58.3	61.7/66.1	Phase III, double-blind, parallel-group RCT, in 74 centres in 11 countries	3	Canagliflozin vs. placebo + sitagliptin	Metformin + pioglitazone	Canagliflozin 100-300 mg/d Sitagliptin 100	26
Rosenstock 2016[189] NCT01809327	T2DM (*n*=1186)	4.1±4.0/3.3±4.5	8.8±1.2/8.8±1.2	NR	54.9/55.2	47.7/48.9	Phase III, double-blind, parallel-group, RCT, in 134 centres in 12 countries	5	Canagliflozin vs. metformin	None	100-300	12
Cefalu 2013[190] NCT00968812	T2DM (*n*=1450)	6.6±5.5/6.6±5.0	7.8±0.8/7.8 ±0.8	NR	56.1/56.3	51.0/55.0	Phase III, double-blind, parallel-group RCT, in 157 centres in 19 countries, CANTATA-SU trial	3	Canagliflozin vs. glimepiride	Metformin	100-300	52
Schernthaner 2013[191] NCT01137812	T2DM (*n*=755)	9.7±6.3/9.4±6.1	8.1±0.9/8.1±0.9	NR	56.5/56.6	55.0/56.9	Phase III, double-blind, parallel-group RCT, in 184 centres in 17 countries	2	Canagliflozin vs. sitagliptin	Metformin + sulfonylurea	Canagliflozin 300 Sitagliptin 100	52
**Dapagliflozin therapy**												
Wiviott 2019[192] NCT01730534	T2DM at risk of CVD (*n*=17160)	11.0(6.0-16.0)/10.0(6.0-16.0)**	8.3±1.2/8.3±1.2	CVD=653 (7.6) vs. 648 (7.6%)	63.9/64.0	63.1/62.1	Phase III, double-blind, parallel-group RCT, in 882 centres in 33 countries, DECLARE-TIMI58 trial	5	Dapagliflozin vs. placebo	None	10	219**
Kaku 2013[193] NCT00972244	T2DM (*n*=279)	4.8±4.4/4.7±3.8	8.1±0.7/8.1±0.7	NR	57.0/58.4	76.3/79.6	Phase IIb, double-blind, parallel-group RCT, in 26 centres in Japan	5	Dapagliflozin vs. placebo	None	1, 2.5, 5, 10	12

Bailey 2012[194] NCT00736879	T2DM (*n*=282)	1.5±2.7/1.1±1.9	7.9±1.0/7.8±1.1	NR	52.8/53.5	48.6/54.4	Phase III, double-blind, parallel-group RCT, in 63 centres in 7 countries	4	Dapagliflozin vs. placebo	None	1, 2.5, 5	24
Kaku 2014[195] NCT01294423	T2DM (*n*=261)	4.5±5.0/5.3±6.2	7.5±0.7/7.5±0.6	NR	58.1/60.4	59.2/59.8	Phase III, double-blind, parallel-group RCT, in 27 centres in Japan	3	Dapagliflozin vs. placebo	None	5-10	24
Bailey 2014[196] NCT00528372	T2DM (*n*=274)	1.8±2.8/2.1±3.1	7.9±0.9/7.8±0.8	NR	52.1/52.7	50.8/41.3	Phase III, double-blind, parallel-group RCT, in 85 centres in 4 countries	4	Dapagliflozin vs. placebo	None	2.5, 5, 10	102
Schumm-Draeger 2015[197] NCT01217892	T2DM (*n*=400)	5.1±4.0/5.5±4.2	7.8±0.7/7.9±0.8	NR	57.4/58.5	44.4/46.5	Phase III, double-blind, parallel-group RCT, in 54 centres in 7 countries	4	Dapagliflozin vs. placebo	Metformin	5-10	16
Bailey 2010[198] NCT00528879	T2DM (*n*=546)	6.2±5.8/5·85±·1	8.0±0.9/8·1±0·9	NR	54.0/53.7	52.8/55.5	Phase III, double-blind, parallel-group RCT, in 75 centres in 5 countries	4	Dapagliflozin vs. placebo	Metformin	2.5, 5, 10	104
Bolinder 2014[199] NCT00855166	T2DM (*n*=182)	6.0±4.5/5.5±5.3	7.2±0.4/7.2±0.5	NR	60.6/60.8	55.1/56.0	Phase III, double-blind, parallel-group RCT, in 40 centres in 5 countries	2	Dapagliflozin vs. placebo	Metformin	10	102
Ji 2014[200] NCT01095653	T2DM (*n*=393)	1.4±2.6/1.3±2.0	8.2±0.8/8.4±0.9	NR	53.0/51.2/49 .9	65.6/64.6/ 65.9	Phase III, double-blind, parallel-group, RCT, in 39 centres in 4 countries	3	Dapagliflozin vs. placebo	Metformin	5-10	24
Henry 2012[201] (Study 1) NCT00643851	T2DM (*n*=598)	1.6±2.8/1.6±2.6	9.2±1.3/9.2±1.3	NR	52.0/51.8	42.8/47.3	Phase III, double-blind, parallel-group RCT, in 105 centres in 4 countries	3	Dapagliflozin vs. placebo	Metformin	5	24
Henry 2012 (Study 2)[201] NCT00859898	T2DM (*n*=638)	2.2±3.6/1.9±4.0	9.1±1.3/9.1±1.3	NR	51.1/52.7	49.1/46.6	Phase III, double-blind, parallel-group RCT, in 131 centres in 4 countries	3	Dapagliflozin vs. placebo	Metformin	10	24
Heerspink 2013[202] NCT00976495	T2DM (*n*=49)	6.5±4.4/6.5±5.0	7.7±0.6/7.5±1.0	NR	53.7/58.0	66.7/72.0	Phase II, double-blind, parallel-group RCT, in 12 centres in USA, Canada and Netherlands	2	Dapagliflozin vs. placebo	Metformin + sulfonylurea	10	12
Matthaei 2015[203] NCT01392677	T2DM (*n*=218)	9.3±6.5/9.6±6.2	8.1±0.9/8.2±0.9	NR	61.1/60.9	42.6/55.6	Phase III, double-blind, parallel-group RCT, in 47 centres in 6 countries	2	Dapagliflozin vs. placebo	Metformin + sulfonylurea	10	52
Mathieu 2015[92] NCT01646320	T2DM (*n*= 320)	7.2±5.7/8.0±6.6	8.2±0.9/8.2±0.9	NR	55.2/55.0	43.8/47.5	Phase III, double-blind, parallel-group RCT, in 67 centres in 8 countries	2	Dapagliflozin vs. placebo	Metformin + sitagliptin	10	24

Jabbour 2014[204] NCT00984867	T2DM (*n*=451)	5.7±4.9/5.6±5.4	7.9±0.8/8.0±0.8	NR	54.8/55.0	57.0/52.7	Phase III, double-blind, parallel-group RCT, in 88 centres in 6 countries	2	Dapagliflozin vs. placebo	± Metformin + sitagliptin	10	24
Wilding 2012[205] NCT00357370	T2DM (*n*=71)	13.6±7.2/13.5±7.3	8.5±0.8/8.5±0.8	NR	56.0/58.4	50.0/69.6	Phase II/III, double-blind, parallel-group, un-controlled RCT, in 23 centres in USA and Canada	4	Dapagliflozin vs. placebo	Metformin ± TZDs + insulin	10-20	12
Mudaliar 2014[206]	T2DM (*n*=44)	9.9±6.5/6.4±5.0	7.5±0.8/7.5±0.7	NR	56.2/53.3	65.2/66.7	Phase IIb, double-blind, parallel-group RCT	2	Dapagliflozin vs. placebo	Metformin ± insulin secretagogue	5	12
NCT00831779[207]	T2DM (*n*=44)	NR	NR	NR	56.2/53.3	65.2/66.7	Phase II, double-blind, parallel-group, RCT, in 3 centres in USA	2	Dapagliflozin vs. placebo	Metformin ± insulin	5	24
Strojek 2011[208] NCT00680745	T2DM (*n*=596)	7.4±5.7/7.4±5.7	8.1±0.8/8.2±0.7	NR	59.7/60.3	48.0/49.0	Phase III, double-blind, parallel-group RCT, in 66 centres in 7 countries	4	Dapagliflozin vs. placebo	Sulfonylurea	2.5, 5, 10	24
Kohan 2014[209] NCT00663260	T2DM with CKD (*n*=252)	17.6±9.5/15.7±9.5	8.3±1.0/8.5±1.3	NR	67.0/67.0	66.1/63.1	Phase II/III, double-blind, parallel-group RCT, in 96 centres in 13 countries	3	Dapagliflozin vs. placebo	Sulfonylurea ± TZDs ± insulin	5-10	104
Rosenstock 2012[210] NCT00683878	T2DM (*n*=420)	5.7±5.9/5.1±5.0	8.4±0.9/8.3±1.0	NR	53.5/53.5	48.7/51.1	Phase III, double-blind, parallel-group RCT, in 89 centres in 9 countries	3	Dapagliflozin vs. placebo	Pioglitazone	5-10	48
Weber 2016[211] NCT01195662	T2DM (*n*=582)	7·7±5·9/7·3±5·0	8·1±0·9/8·0±1·0	NR	56.0/57.0	52.4/57.6	Phase III, double-blind, parallel-group RCT, in 298 centres in 16 countries	2	Dapagliflozin vs. placebo	TZDs ± insulin	10	12
NCT01137474[212]	T2DM CKD on ACE ± ARB (*n*=944)	NR	NR	NR	NR	55.8/55.0	Phase III, double-blind, parallel-group RCT, in 329 centres in 16 countries	4	Dapagliflozin vs. placebo	TZDs ± insulin	2.5, 5, 10	12
Wilding 2014[213] NCT00673231	T2DM (*n*=807)	13.6±7.2/13.5±7.3	8.6±0.8/8.5±0.8	NR	59.5/58.8	45.5/49.2	Phase III, double-blind, parallel-group RCT, in 96 centres in 13 countries	4	Dapagliflozin vs. placebo	Insulin	2.5, 5, 10	104
Yang 2018[214] NCT02096705	T2DM (*n*=272)	12.7±7.2/12.2±6.7	8.5±0.7/8.5±0.8	NR	56.5/58.6	47.5/48.1	Phase III, double-blind, parallel-group, RCT, in 29 centres in 7 countries	2	Dapagliflozin vs. placebo	Insulin	10	24
Cefalu 2015[215] NCT01031680	T2DM with CVD and hypertension (*n*=922)	12.6±8.7/12.3±8.2	8.2±0.8/8.1±0.8	Stroke/TIA 100 (22%) vs. 89 (19.4%)	62.8/63.0	67.9/68.6	Phase III, double-blind, parallel-group RCT, in 141 centres in 5 countries	2	Dapagliflozin vs. placebo	AHG agents	10	104

Leiter 2014[216] NCT01042977	T2DM with CVD (*n*=965)	13.5±8.2/13.0±8.4	8.0±0.8/8.1±0.8	Stroke/TIA 105 (21.9%) vs. 84 (17.4%)	63.9/63.6	66.8/67.0	Phase III, double-blind, parallel-group RCT, in 173 centres in 6 countries	2	Dapagliflozin vs. placebo	AHG agents	10	52
List 2009[217] NCT00263276	T2DM (*n*=359)	NR	7.8±0.9/7.8±0.9	NR	55.0/53.5	49.8/52.0	Phase II, double-blind, parallel-group RCT, in 145 centres in USA, Canada, Mexico and Puerto Rico	7	Dapagliflozin vs. placebo + metformin	None	2.5, 5, 10, 20, 50	12
Nauck 2014[218] NCT00660907	T2DM (*n*=814)	6.0±5.0/7.0±6.0	7.7±0.9/7.7±0.9	NR	58.1/58.6	55.3/54.9	Phase III, double-blind, parallel-group RCT, in 95 centres in 10 countries	2	Dapagliflozin vs. glipizide	Metformin	10	156
Rosenstock 2015[219] NCT01606007	T2DM (*n*=534)	7.1±5.0/7.4±5.4/8.2±5.5	8.9±1.2/8.9±1.2/9.0±1.1	NR	53.5/54.0	49.7/50.5	Phase III, double-blind, parallel-group RCT, in 139 centres in 8 countries	3	Dapagliflozin vs. saxagliptin	Metformin	Dapagliflozin 10 Saxagliptin 5	24
**Empagliflozin therapy**												
Kadowaki 2015[220] NCT01193218	T2DM (*n*=861)	NR	7.9±0.7/NR	NR	57.3/58.7	75.4/73.4	Phase II, double-blind, parallel-group RCT, in 32 centres in Japan	5	Empagliflozin vs. placebo	None	5, 10, 25, 50	12
NCT01649297[221]	T2DM (*n*=983)	NR	NR	NR	58.3/57.9	54.2/55.0	Phase II, double-blind, parallel-group RCT, in 139 centres in 18 countries	5	Empagliflozin vs. placebo	Metformin	5, 10, 12.5, 25	16
Merker 2015[222] NCT01289990; NCT01159600	T2DM (*n*=637)	≤1->10/≤1->10	8.1±0.8/8.2±0.8	NR	54.7/56.4	54.9/52.8	Phase III, double-blind, open-label extension period, parallel-group, RCT, in 148 centres in 12 countries, EMPA-REG EXTEND™ MET trial	3	Empagliflozin vs. placebo	Metformin	10-25	76
Ha¨ring 2013[223] NCT01159600	T2DM (*n*=500)	≤1->10/≤1->10	9.1±0.9/8.2±0.8	NR	54.8/56.5	55.0/53.0	Phase III, double-blind, empagliflozin open-label, parallel-group RCT, in 148 centres in 12 countries	4	Empagliflozin vs. placebo	Metformin ± sulfonylurea	10-25	24
Rosenstock 2015[224] NCT01011868	T2DM (*n*=494)	≤1->5/≤1->5	8.3±0.8/8.2±0.8	NR	59.3/58.1	54.5/53.0	Phase IIb, double-blind, parallel-group, RCT, in 99 centres in 7 countries	3	Empagliflozin vs. placebo	± Metformin ± sulfonylurea ± insulin	10-25	78
Zinman 2015[225] NCT01131676	T2DM with established CVD and CKD (*n*=7020)	≤1->10/≤1->10	8.1±0.9/8.1±0.8	Stroke 1084 (23.1%) vs. 553 (23.7%)	63.0/63.2	71.2/72.0	Double-blind, parallel-group, RCT in at 590 sites in 42 countries, EMPA-REG OUTCOME trial	3	Empagliflozin vs. placebo	Metformin ± sulfonylurea ± insulin	10-25	161**

Barnett 2014[176] NCT01164501	T2DM with CKD (*n*=738)	≤1->10/≤1->10	7.9±0.8/8.1±0.8	NR	63.6/64.1	60.1/56.7	Phase III, double-blind, parallel-group RCT, in 127 centres in 15 countries	3	Empagliflozin vs. placebo	Metformin ± sulfonylurea ± insulin	10-25	24
Rosenstock 2014[226] NCT01306214	T2DM (*n*=563)	≤1->10/≤1->10	8.3±0.7/8.3±0.7	NR	57.4/55.3	39.9/48.3	Phase III, double-blind, parallel-group RCT, in 103 centres in 14 countries	3	Empagliflozin vs. placebo	Metformin ± insulin	10-25	52
Kovacs 2014[227] NCT01210001	T2DM (*n*=498)	>10.0/>10.0	8.1±0.8/8.2±0.9	NR	54.5/54.6	50.4/44.2	Phase III, double-blind, parallel-group, RCT, in 68 centres in 7 countries	3	Empagliflozin vs. placebo	± Metformin + pioglitazone	10-25	24
Kovacs 2014[227] NCT01289990	T2DM (*n*=498)	≤1->10/≤1->10	8.1±0.8/8.2±0.9	NR	54.0/55.6	55.6/51.0	Phase III, double-blind, parallel-group, RCT, in 69 centres in 8 countries, EMPA-REG PIO™ trial	3	Empagliflozin vs. placebo	Pioglitazone	10-25	76
Tikkanen 2015[228] NCT01370005	T2DM with hypertension (*n*=824)	≤1->10/≤1->10	7.8±0.7/7.9±0.7	NR	60.3/60.3	59.2/62.0	Phase III, double-blind, parallel-group RCT, in 120 centres in 12 countries, EMPA-REG BP trial	3	Empagliflozin vs. placebo	AHG agents	10-25	12
Roden 2013[75] NCT01177813	T2DM (*n*=763)	≤1->10≤1->10	9.1±1.0/7·9±0·8	NR	53.4/55.0	67.2/58.6	Phase III, double-blind, parallel-group RCT, in 124 centres in 9 countries	4	Empagliflozin vs. placebo	None	10-25	24
Søfteland 2017[26] NCT01734785	T2DM (*n*=332)	≤1->10/≤1->10	7.9±0.8/7.9±0.8	NR	54.9/55.9	61.7/55.5	Phase III, double-blind, parallel-group, RCT, in 90 centres in 11 countries	3	Empagliflozin vs. placebo	Metformin	10-25	24
Rosenstock 2013[84] NCT00749190	T2DM (*n*=162)	NR	7.9±0.7/8.1±0.9	NR	58.1/58.7	50.7/50.0	Phase II, double-blind, parallel-group RCT, in 116 centres in 16 countries	6	Empagliflozin vs. placebo	Metformin	1, 5, 10, 25, 50	12
Ferrannini 2013[229] NCT00789035	T2DM (*n*=406)	NR	7.9±0.8/7.9±0.9	NR	57.7/57.3	52.1/51.9	Phase IIb, double-blind, parallel-group RCT, open-label metformin, in 74 centres in 13 countries	5	Empagliflozin vs. placebo + metformin	None ± AHGs agents	5, 10, 25	12
Ferrannini 2013 (Study 1)[229] NCT00881530	T2DM (*n*=271)	≤1->5/≤1->5	7.9±0.8/8.2±0.9	NR	58.8/56.8	48.9/50.0	Phase IIb, open-label, parallel-group RCT, in 132 centres in 21 countries	3	Empagliflozin vs. metformin	None	5, 10, 25	78
Hadjadj 2016[230] NCT01719003	T2DM (*n*=1373)	≤1->10/≤1->10	7.1±1.2/7.0±1.2	NR	52.6/52.5	57.3/53.7	Phase III, double-blind, parallel-group, RCT, in 190 centres in 21 countries	9	Empagliflozin vs. metformin	None	10-25	26
Araki 2015[231] NCT01368081	T2DM (*n*=1160)	≤1->10/≤1->10	7.8±0.8/7.9±0.8	NR	61.0/60.0	70.6/74.6	Phase III, double-blind, parallel-group, RCT, in 86 centres in Japan	13	Empagliflozin vs. metformin	Sulfonylurea ± glinide ± biguanide ± TZDs	10-25	52

										± α-glucosidase-Is<br>± DPP-IV		
Ridderstra°le 2014[232]<br>NCT01167881	T2DM<br>(*n*=1545)	≤1->10/≤1->10	7·9±0·8/7·9±0·9	NR	56.2/55.7	56.5/54.0	Phase III, double-blind, parallel-group, RCT, in 182 centres in 23 countries	2	Empagliflozin vs. glimepiride	Metformin	25	104
DeFronzo 2015[233]<br>NCT01422876	T2DM (*n*=674)	≤1->10/≤1->10	8.0±0.8/8.0±0.9	NR	56.2/56.2	54.6/50.0	Phase III, double-blind, parallel-group RCT, in 197 centres in 22 countries	5	Empagliflozin vs. linagliptin	Metformin	10-25	52
Ferrannini 2013<br>(Study 2)[229]<br>NCT00881530	T2DM (*n*=271)	≤1->5/≤1->5	7.8±0.8/8.0±0.9	NR	58.8/58.6	48.9/51.9	Phase IIb, double-blind empagliflozin, open-label sitagliptin, parallel-group RCT, in 132 centres in 21 countries	3	Empagliflozin vs. sitagliptin	Metformin	Empagliflozin<br>10-25<br>Sitagliptin 100	78
**Ertugliflozin therapy**												
Cannon 2020[234]<br>NCT01986881	T2DM with atherosclerosis<br>(*n*=8246)	12.9±8.3/13.1±8.4	8.2±1.0/8.2±0.9	CVD<br>1276 (23.2%) vs. 613 (22.3%)<br>Stroke<br>1181 (21.5%) vs. 558 (20.3%)	64.4±8.1/<br>64.4±8.0	70.3/69.3	Phase III, double-blind, RCT, in 149 centres in 23 countries, VERTIS CV trial	3	Ertugliflozin vs. placebo	None ± Metformin ± sulfonylurea ± DPP4-Is ± GLP1-RAs	5-15	183
Dagogo-Jack 2018[235]<br>NCT02036515	T2DM with atherosclerosis<br>(*n*=462)	9.5±5.7/9.4±5.6	8.1±0.9/8.0±0.9	NR	59.5/58.3	52.8/65.4	Phase III, double-blind, RCT, in 149 centres in 23 countries, VERTIS CV trial	3	Ertugliflozin vs. placebo	Metformin + sitagliptin	5-15	318
Miller 2018[236]<br>NCT02226003	T2DM (*n*=291)	6.1±5.7/6.8±6.5	8.9±0.9/9.0±0.9	NR	56.3/54.3	56.7/58.8	Phase III, double-blind, parallel-group, RCT	3	Ertugliflozin + sitagliptin[¶] vs. placebo	None or metformin, α-glucosidase-Is, sulfonylureas and glinides	Ertugliflozin 5-15<br>Sitagliptin 100	26
Amin 2015[94]<br>NCT01059825	T2DM with hypertension<br>(*n*=648)	6.3±0.8/6.4±0.9	8.2±0.1/8.2±0.1	NR	53.6/55.1	48.2/63.2	Phase II, double-blind, parallel-group, RCT	6	Ertugliflozin vs. placebo + metformin	AHG agents	1, 5, 10, 25	12
**Ipragliflozin therapy**												
Wilding 2013[237]<br>NCT01117584	T2DM (*n*=342)	6.0±5.3/5.7±3.2	7.8±0.7/7.7±0.6	NR	57.4/75.3	50.4/54.5	Phase II, double-blind, parallel-group, RCT, in 45 centres in 6 countries	5	Ipragliflozin vs. placebo	Metformin	12.5, 50, 150, 300	12

Kashiwagi 2015[238] NCT01135433	T2DM (*n*=168)	8.1±8.1/8.1±1.9	8.3±0.7/8.4±0.7	NR	56.2/57.7	58.9/58.9	Phase III, double-blind, parallel-group, RCT, in 7 centres in Japan, ILLUMINATE trial	2	Ipragliflozin vs. placebo	Metformin	50	24
Lu 2016[239] NCT01505426	T2DM (*n*=170)	NR	NR	NR	NR	NR	Phase III, double-blind, parallel-group, RCT, in 12 centres in Korea and Taiwan	2	Ipragliflozin vs. placebo	Metformin	NR	24

Abbreviations: *ACS*, acute coronary syndrome; *ACE*, angiotensin-converting enzyme inhibitor; *ARB*, angiotensin receptors blocker; *AHG*, anti-hyperglycaemic drugs; *CVD*, cardiovascular disease; *CVD*, cerebrovascular disease; *CKD*, chronic kidney disease; C*RF*, chronic renal failure; *CHF*, congestive heart failure; *IGT*, impaired glucose tolerance; *mμ/d*, microgram pear day; *mg/d*, milligram pear day; NR, not report; *RCT*, randomized controlled trial; *TZDs*, thiazolidinedione; *T2DM*, type 2 diabetes mellitus.

Note: ‡ Mean age and dosages present for intervention drugs; ** Median age; **Median year; **Median (interquartile range); ¶Novel treatment of fixed-dose combination of 2 different classes of AHG; †values are expressed as mean ± SD or ǂmean ± SE.

a Estimated glomerular filtration rate (eGFR) of 30 to <60 ml per minute per 1.73 m^2.

Stroke outcome results

DPP4-Is

Table S4. Trial stroke outcomes with DPP4-Is

Study<br>Trial registry	Type of stroke index event	Intervention of interest		Number of strokes				Ischaemic stroke				Haemorrhagic stroke				Fatal stroke			
		DPP4-Is (mg/day)	Controls (mg/day)	DPP4-Is		Controls		DPP4-Is		Controls		DPP4-Is		Controls		DPP4-Is		Controls	
				Event	Sample size	Event	Sample size	Event	Sample size	Event	Sample size	Event	Sample size	Event	Sample size	Event	Sample size	Event	Sample size
**Alogliptin therapy**																			
DeFronzo 2008[1]	SAEs	Alogliptin 12.5	Placebo	0	264	0	64	0	133	0	64	NR				NR			
NCT00286455		Alogliptin 25						0	131										
Nauck 2009[2]	SAEs	Alogliptin 12.5	Placebo	0	423	0	104	0	213	0	104	NR				NR			
NCT00286442		Alogliptin 25						0	210										
Pan 2017[3]	SAEs	Alogliptin 25	Placebo	1	252	0	253	0	92	0	92	NR				NR			
NCT01289119		Alogliptin 25 + metformin 1000	Metformin					0	99	0	98								
		Alogliptin 25 + pioglitazone	Pioglitazone					1	61	0	63								
Pratley 2009[4]	SAEs	Alogliptin 12.5	Placebo	0	337	0	97	0	138	0	97	NR				NR			
NCT00286494		Alogliptin 25						0	199										
White 2013[5]	Non-fatal 1[ry]	Alogliptin	Placebo	29	2701	32	2679	29	2701	32	2679	NR				NR			
NCT00968708	and 2[ry] outcome																		
Rosenstock 2009[6]	SAEs	Alogliptin 12.5	Placebo	0	260	1	129	0	131	1	129	NR				NR			
NCT00286429		Alogliptin 25						0	129										
Seino 2012[7]	SAEs	Alogliptin 12.5	Placebo	1	209	0	103	1	105	0	103	NR				NR			
NCT01318083;		Alogliptin 25						0	104										
NCT01318135																			
Pratley 2009[8]	SAEs	Alogliptin 12.5	Placebo	0	401	0	99	0	203	0	99	NR				NR			
NCT00286468		Alogliptin 25						0	198										
Seino 2011[9]	SAEs	Alogliptin 12.5	Placebo	1	155	1	75	0	76	1	75	NR				NR			

NCT01263483		Alogliptin 25						1								
DeFronzo 2012[10]	SAEs	Alogliptin 12.5 + pioglitazone 15	Placebo	2	1037	1	516	0	130	0	128					
NCT00328627		Alogliptin 25 + pioglitazone 15	Placebo					0	130	0	129	NR				NR
		Alogliptin 12.5 + pioglitazone 30	Placebo					0	130	0	129					
		Alogliptin 25+ pioglitazone 30	Placebo					1	130	0	129					
		Alogliptin 12.5 + pioglitazone 45	Placebo					0	130	0	129					
		Alogliptin 25 + pioglitazone 45	Placebo					1	130	1	129					
Seino 2011[11]	SAEs	Alogliptin 6.25	Placebo	1	322	0	158	1	79	0	75	NR				NR
NCT01263470		Alogliptin 12.5	Voglibose 0.6					0	84	0	83					
		Alogliptin 25						0	80							
		Alogliptin 50						0	79							
NCT01263496[12]	SAEs	Alogliptin 6.25	Voglibose 0.2	3	391	0	83	1	96	83	83	NR				NR
		Alogliptin 12.5						0	101							
		Alogliptin 25						1	97							
		Alogliptin 50						1	97							
Pratley 2014[13]	SAEs	Alogliptin 12.5	Placebo	2	442	0	326	2	110	0	106	NR				NR
NCT01023581		Alogliptin 25	Metformin 500					0	112	0	109					
		Alogliptin + metformin¶ 12.5/500	Metformin 1000					0	106	0	111					
		Alogliptin + metformin¶ 12.5/1000						0	114							
Bosi 2011[14]	SAEs	Alogliptin 25 + metformin 1500 +	Metformin 1500 +	1	404	1	399	1	404	1	399	NR				NR
NCT00432276		pioglitazone 30	pioglitazone 4													
NCT00707993[15]	SAEs	Alogliptin 25	Glipizide 5	1	222	1	219	1	222	1	219	NR				NR
Del Prato 2014[16]	SAEs	Alogliptin 12.5 + metformin	Metformin +	10	1751	3	869	4	873	3	869	1	873	0	869	NR
NCT00856284		Alogliptin 25 + metformin	Glipizide					5	878			0	878			
Rosenstock 2010[17]	SAEs	Alogliptin 25	Pioglitazone 30	0	491	1	163	0	164	1	163	NR				NR
NCT00395512		Alogliptin 12.5 + pioglitazone 30						0	163							
		Alogliptin 25+ pioglitazone 30						0	164							
NCT01318070[18]	SAEs	Alogliptin 12.5	Pioglitazone	0	224	1	115	0	111	1	115	NR				NR
		Alogliptin 25						0	113							

Mita 2016[19]	AEs and SAEs	Alogliptin 25	Conventional	0	161	2	161	0	161	2	161	NR	NR			
UMIN000005311			treatment													
**Dutogliptin therapy**																
Pattzi 2010[20]	SAEs	Dutogliptin 200	Placebo	0	336	1	86	0	174	1	86	NR	NR			
		Dutogliptin 400						0	162							
Garcia-Soria 2008[21]	SAEs	Dutogliptin 100	Placebo	0	133	1	41	0	43	1	41	NR	NR			
		Dutogliptin 200						0	44							
		Dutogliptin 400						0	46							
**Linagliptin therapy**																
Rosenstock 2019[22]	Fatal and non-	Linagliptin 5	Placebo	81	3494	88	3485	65	3494	73	3485	NR	17	3494	16	3485
NCT01897532	fatal 1^{ry} and 2^{ry}															
	outcome															
Del Prato 2010[23]	SAEs	Linagliptin 5	Placebo	0	336	0	167	0	336	0	167	NR	NR			
NCT00621140																
Taskinen 2011[24]	SAEs	Linagliptin 5	Placebo	0	523	1	177	0	523	1	177	NR	NR			
NCT00601250																
Haak 2012[25]	SAEs	Linagliptin 5	Metformin 1000	0	394	0	363	0	42	0	144					
NCT00798161		Linagliptin 2.5 + metformin 1000	Metformin 2000					0	143	0	147	NR	NR			
		Linagliptin 2.5 + metformin 2000	Placebo					0	143	0	72					
		Linagliptin 2.5 + metformin 2000						0	66							
		(open-label)														
Søfteland 2017[26]	SAEs	Linagliptin 5	Placebo	0	606	0	110	0	606	0	110	NR	NR			
NCT01734785																
Owens 2011[27]	SAEs	Linagliptin 5	Placebo	0	792	0	263	0	792	0	263	NR	NR			
NCT00602472																
Bajaj 2014[28]	SAEs	Linagliptin 5	Placebo	0	183	0	89	0	183	0	89	NR	NR			
NCT00996658																
Barnett 2013[29]	SAEs	Linagliptin 5	Placebo	1	162	0	79	1	162	0	79	NR	NR			
NCT01084005																

Lewin 2012[30]	SAEs	Linagliptin 5	Placebo	0	161	0	84	0	161	0	84		NR			NR
NCT00819091																
Gomis 2011[31]	SAEs	Linagliptin 5	Placebo	0	259	0	130	0	259	0	130		NR			NR
NCT00641043																
Yki-Järvinen 2013[32]	SAEs	Linagliptin 5	Placebo	5	631	4	630	4	631	3	630	1	631	1	630	
Duran-Garcia 2015																NR
NCT00954447																
Wang 2016[33]	SAEs	Linagliptin 5	Placebo	0	205	1	100	0	205	1	100		NR			NR
NCT01215097																
1218.43[a] 2014[34]	SEs	Linagliptin 5	Placebo	1	68	3	65	1	68	3	65		NR			NR
NCT00800683																
McGIll 2013[35]	AEs	Linagliptin 5	Placebo	1	68	1	65	1	68	1	65		NR			NR
PMCID: PMC3554278																
Groop 2017[36]	SAEs	Linagliptin 5	Placebo	1	182	1	178	0	182	1	178	1	182	0	170	NR
NCT01792518																
Kawamori 2012[37]	SAEs	Linagliptin 5	Placebo	4	319	2	242	2	159	0	80	1	159	0	80	NR
NCT00654381		Linagliptin 10	Voglibose 0.6					1	160	2	162	0	160	0	162	
Mu 2017[38]	SAEs	Linagliptin 5	Metformin 1000	1	713	1	289	1	251	1	145		NR			NR
NCT01708902		Linagliptin 5 / linagliptin 2.5	Metformin 2000					0	31	0	144					
		Linagliptin 2.5 + metformin 1000						0	212							
		Linagliptin 2.5 + metformin 2000						0	219							
Haak 2013[39]	SAEs	Linagliptin 2.5 + metformin 500	Metformin 1000	1	396	1	170	1	225	1	170		NR			NR
NCT00915772		Linagliptin 2.5 + metformin 100						0	171							
NCT01204294; 2014[40]		Linagliptin 5 + biguanide	Sulfonylure +	3	450	1	124	1	82	1	63	0	82	0	63	NR
		Linagliptin 5 + glinide	metformin					0	66	0	61	0	66	0	61	
		Linagliptin 5 + Sulfonylure	α-glucosidase -Is +					0	143			1	143			
		Linagliptin 5 + glitazone	metformin					0	74			0	74			
		Linagliptin 5 + α-glucosidase-Is						0	85			1	85			

Barnett 2012[41] NCT00740051	SAEs	Linagliptin 5	Placebo + glimepiride 1-4	0	151	1	76	0	151	1	76	NR	NR			
Laakso 2015[42] NCT01087502	SAEs	Linagliptin 5	Placebo + glimepiride 1-4	1	113	2	122	1	113	2	122	NR	NR			
Gallwitz 2012[43] NCT00622284	Non-fatal SAEs	Linagliptin 5	Glimepiride	3	776	11	775	3	776	11	775	NR	NR			
Rosenstock 2019[44] NCT01243424	Non-fatal SAEs	Linagliptin 5	Glimepiride	104	3023	120	3010	91	3023	104	3010	NR	13	3023	16	3010
**Omarigliptin therapy**																
Gantz 2017[45] NCT01703208	Fatal and non-fatal stroke	Omarigliptin 25	Placebo	32	2092	34	2100	32	2092	34	2100	NR	NR			
Goldenberg 2017[46] NCT01841697	SAEs	Omarigliptin 25	Sitagliptin 100	0	322	2	320	0	322	2	320	NR	NR			
**Saxagliptin therapy**																
Pan 2012[47] NCT00698932	SAEs	Saxagliptin 5	Placebo	1	284	0	284	1	284	0	284	NR	NR			
Rosenstock 2008[48] NCT00950599	SAEs	Saxagliptin 2.5	Placebo	0	315	0	108	0	55	0	108	NR	NR			
		Saxagliptin 5						0	47							
		Saxagliptin 10						0	63							
		Saxagliptin 20						0	54							
		Saxagliptin 40						0	52							
		Saxagliptin 100						0	44							
Kumar 2014[49] NCT00918879	SAEs	Saxagliptin 5	Placebo	0	107	0	106	0	107	0	106	NR	NR			
Frederich 2012[50] NCT00316082	SAEs	Saxagliptin 2.5 (A.M.)	Placebo	0	291	1	74	0	74	1	74	NR	NR			
		Saxagliptin 2.5/5 (A.M.)						0	74							
		Saxagliptin 5 (A.M.)						0	71							
		Saxagliptin 5 (P.M)						0	72							
Rosenstock 2013[51] NCT00121641	SAEs	Saxagliptin 2.5	Placebo	2	372	0	95	0	102	0	95	NR	NR			
		Saxagliptin 5						1	106							

		Saxagliptin 10						0	98										
		Saxagliptin 10 (open-label)						0	66										
DeFronzo 2013[51]	SAEs	Saxagliptin 2.5 + metformin	Placebo + metformin	5	564	0	179	1	192	0	179	NR				NR			
NCT00121667		Saxagliptin 5 + metformin						1	191										
		Saxagliptin 10 + metformin						3	181										
Yang 2011[52]	SAEs	Saxagliptin 5 + metformin	Placebo + metformin	1	283	1	287	1	283	1	287	NR				NR			
NCT00661362																			
Stenlöf 2010[53]	SAEs	Saxagliptin 5 + metformin	Placebo + metformin	0	46	0	47	0	46	0	47	NR				NR			
NCT00683657																			
White 2014[54]	SAEs	Saxagliptin 5 + metformin	Placebo + metformin	0	74	0	86	0	74	0	86	NR				NR			
NCT00885378																			
Chacra 2011[55]	SAEs	Saxagliptin 2.5 + glyburide 7.5	Placebo + glyburide 7.5	1	501	2	267	1	248	1	267	0	248	1	267	NR			
NCT00313313		Saxagliptin 5 + glyburide 7.5						0	253			0	253						
Nowicki 2011[56]	SAEs	Saxagliptin 2.5	Placebo	1	85	1	85	1	85	1	85	NR				NR			
NCT00614939																			
Hollander 2011[57]	SAEs	Saxagliptin 2.5	Placebo	4	381	1	184	3	195	1	184	0	195	0	184	NR			
NCT00295633		Saxagliptin 5						0	186			1	186						
Barnett 2012[58]	SAEs	Saxagliptin 5	Placebo	0	304	2	151	0	304	2	151	NR				NR			
NCT00757588																			
Scirica 2013[59]	Fatal and non-fatal 1ry and 2ry outcome	Saxagliptin	Placebo	179	8280	176	8212	157	8280	141	8212	NR				22	8280	35	8212
NCT01107886																			
Henry 2011[60]	SAEs	Saxagliptin 5	Placebo + metformin	0	20	0	16	0	20	0	16	NR				NR			
NCT00374907																			
Jadzinsky 2009	SAEs	Saxagliptin 10	Metformin	4	978	1	328	2	335	1	328	0	335	0	328	NR			
Pfützner 2011[61]		Saxagliptin 5 + metformin						1	320			0	320						
NCT00327015		Saxagliptin 10 + metformin						0	323			1	323						
Hermans 2012[62]	SAEs	Saxagliptin 5	Metformin 1500	0	147	0	139	0	147	0	139	NR				NR			
NCT01006590																			
Neutel 2013[63]	SAEs	Saxagliptin 5	Metformin 1500	0	46	0	47	0	46	0	47	NR				NR			

NCT00918138																			
Fonseca 2012[64]	SAEs	Saxagliptin 5	Metformin 1500	0	138	0	144	0	138	0	144	NR				NR			
NCT00960076																			
Göke 2013[65]	SAEs	Saxagliptin 5 + metformin	Glipizide 15 +	4	428	4	430	4	428	4	430	NR				1	428	0	430
NCT00575588			metformin																
Schernthaner 2015[66]	SAEs	Saxagliptin 5	Glimepiride ≤6	0	359	2	359	0	359	2	359	NR				NR			
NCT01006603																			
Scheen 2010[67]	SAEs	Saxagliptin 5 + metformin	Sitagliptin 100 +	0	403	1	398	0	403	1	398	NR				NR			
NCT00666458			metformin																
**Sitagliptin therapy**																			
Hage 2014[68]	AEs	Sitagliptin 100	Placebo	1	34	0	37	1	34	0	37	NR				NR			
NCT00627744																			
Mohan 2009[69]	SAEs	Sitagliptin 100	Placebo	1	352	0	178	1	352	0	178	NR				NR			
NCT00289848																			
Barzilai 2011[70]	SAEs	Sitagliptin 50-100	Placebo	0	102	0	104	0	102	0	104	NR				NR			
NCT00305604																			
Ji 2016[71]	SAEs	Sitagliptin 100	Metformin 100	1	367	1	377	1	120	0	126	0	120	0	126	NR			
NCT01076088		Sitagliptin 100 + metformin 1000	Metformin 1700					0	122	0	124	0	122	1	124				
		Sitagliptin 100 + metformin 1700	Placebo					0	125	0	127	0	125	0	127				
Scott 2007[72]	SAEs	Sitagliptin	Placebo	0	495	0	125	0	495	0	125	NR				NR			
NCT00482079																			
HU 2015[73]	SAEs	Sitagliptin 100	Placebo	2	223	1	223	2	223	1	223	NR				NR			
NCT01289990																			
Aschner 2006[74]	SAEs	Sitagliptin 100	Placebo	1	488	1	253	0	238	1	253	NR				NR			
NCT00087516		Sitagliptin 200						1	250										
Roden 2013[75]	SAEs	Sitagliptin 100	Placebo	2	223	1	229	2	223	1	229	NR				NR			
NCT01177813																			
Hanefeld 2007[76]	SAEs	Sitagliptin	Placebo	0	441	0	111					NR				NR			
NCT00481663																			

Wang 2017[77] NCT01177384	SAEs	Sitagliptin 100	Placebo	2	191	0	189	2	191	0	189	NR				NR			
Yang 2012[78] NCT00813995	SAEs	Sitagliptin 100 + metformin 1500-1700	Placebo + metformin 1500-1700	2	197	1	198	2	197	1	198	NR				NR			
Raz 2008[79] NCT00337610	SAEs	Sitagliptin 100	Placebo	0	96	0	94	0	96	0	94	NR				NR			
NCT00420511[80]	SAEs	Sitagliptin 100	Placebo	0	10	0	11	0	10	0	11	NR				NR			
Bergenstal 2012[81] NCT00754988	SAEs	Sitagliptin 100	Placebo	0	184	0	90	0	184	0	90	NR							
Gadde 2017[82] NCT01652729	SAEs	Sitagliptin 100	Placebo	0	122	0	61	0	122	0	61	NR				NR			
Rosenstock 2012[83] NCT00642278	SAEs	Sitagliptin 100	Placebo	0	65	0	65	0	65	0	65	NR				NR			
Rosenstock 2013[84] NCT00749190	SAEs	Sitagliptin 100	Placebo	0	25	0	26	0	25	0	26	NR				NR			
Ba 2017[85] NCT01590771	SAEs	Sitagliptin 100	Placebo	0	248	2	249	0	248	1	249	0	248	1	249	NR			
Dobs 2013[86] NCT00350779	SAEs	Sitagliptin 100	Placebo	1	170	2	341	1	170	0	341	0	170	2	341	NR			
Fonsec 2013[87] NCT00885352	SAEs	Sitagliptin 100	Placebo	0	157	0	156	0	157	0	156	NR				NR			
Rosenstock 2006[88] NCT00086502	SAEs	Sitagliptin 100 + pioglitazone 30-45	Placebo + pioglitazone 30-45	0	175	0	178	0	175	0	178								
Green 2015[89] NCT00790205	Fatal or non-fatal 1^{ry} and 2^{ry} outcome	Sitagliptin	Placebo	147	7257	159	7266	147	7257	159	7266	NR				31	7257	24	7266
Shankar 2017[90] NCT01590797	SAEs	Sitagliptin 100	Placebo	1	234	1	233	1	234	1	233	NR				NR			

Vilsbøll 2010[91] NCT00395343	SAEs	Sitagliptin 100mg + insulin ≥15IU ± metformin 1500	Placebo + insulin ≥15IU ± metformin 150	0	322	0	319	0	322	0	319	NR				NR
Mathieu 2015[92] NCT01462266	SAEs	Sitagliptin 100	Placebo	0	329	0	329	0	329	0	329	NR				NR
Williams-Herman 2010[93] NCT00103857	SAEs	Sitagliptin 100<br>Sitagliptin 1000 + metformin 100<br>Sitagliptin 1000 + metformin 2000<br>Sitagliptin 100 + metformin 2000	Metformin 1000<br>Metformin 2000<br>Placebo + metformin 2000	1	668	3	540	1<br>0<br>0<br>0	179<br>190<br>182<br>117	0<br>0<br>3	182<br>182<br>176	NR				NR
Amin 2015[94] NCT01059825	SAEs	Sitagliptin 100<br>Metformin	Placebo	0	55	0	429	0<br>0	55<br>375	0	54	NR				NR
Aschner 2010[95] NCT00449930	SAEs	Sitagliptin 100	Metformin 2000	0	528	0	522	0	528	0	522	NR				NR
Olansky 2011[96] NCT00482729	SAEs	Sitagliptin 100-200 + metformin 1000-2000§	Metformin 1000-2000	1	625	4	621	1	625	4	621	NR				NR
Perez-Monteverde 2011[97] NCT00541450	SAEs	Sitagliptin 100-200 + metformin 1000-2000§	Pioglitazone 30-45	0	224	0	248	0	224	0	248	NR				NR
Wainstein 2012[98] NCT00532935	SAEs	Sitagliptin 100-200 + metformin 1000-2000§	Pioglitazone	2	261	0	256	2	261	0	256	NR				NR
Russel-Jones 2012[99] NCT00676338	SAEs	Sitagliptin 100	Metformin<br>Pioglitazone	0	163	1	409	0	163	1<br>0	246<br>163	NR				NR
Raz 2006[100] NCT00094757	SAEs	Sitagliptin 100<br>Sitagliptin 200	Placebo + pioglitazone	5	411	0	110	2<br>0	205<br>206	0	110	3<br>0	205<br>206	0	110	NR
NCT01076075[101]	SAEs	Sitagliptin 100	Placebo + pioglitazone 30	1	210	0	212	1	210	0	212	NR				NR
Hermansen 2007[102] NCT00106704	SAEs	Sitagliptin 100	Placebo	2	222	1	219	2	222	1	219	NR				NR
Yoon 2012[103] NCT01028391	SAEs	Sitagliptin 100 + pioglitazone 45	Pioglitazone 45	0	164	1	153	0	164	1	153	NR				NR

Yoon 2011[103] NCT00397631	SAEs	Sitagliptin 100 + pioglitazone 45	Pioglitazone 45	0	164	2	153	0	164	2	153	NR				NR			
Bergenstal 2010[104] NCT00637273	SAEs	Sitagliptin 100	Pioglitazone 45	1	166	1	165	1	166	1	165	NR				NR			
NCT00722371[105]	SAEs	Sitagliptin 100	Pioglitazone 15	3	922	2	693	1	231	1	230	0	231	0	230	NR			
		Sitagliptin 100 + pioglitazone 15	Pioglitazone 30					0	230	1	233	1	230	0	233				
		Sitagliptin 100 + pioglitazone 30	Pioglitazone 45					1	231	0	230	0	231	0	230				
		Sitagliptin 100 + pioglitazone 45						0	230			0	230						
Chan 2008[106] NCT00095056	SAEs	Sitagliptin 25	Placebo + glipizide 5-20	2	65	1	26	2	65	1	26	NR				NR			
Charbonnel 2006[107] NCT00086515	SAEs	Sitagliptin 100	Placebo + glipizide 5	3	464	1	237	0	464	1	237	3	464	0	237				
Ahrén 2014[108] NCT00838903	SAEs	Sitagliptin 100	Placebo	0	302	2	408	0	302	1	101	0	302	1	101	NR			
			Glimepiride 2							0	307			0	307				
Arjona Ferreira 2013[109] NCT00509236	SAEs	Sitagliptin 25	Glipizide 2.5-10	0	64	0	65	0	64	0	65	NR				NR			
Ferreira 2013[110] NCT00509262	SAEs	Sitagliptin 25-50	Glipizide 2.5-20	5	210	4	212	4	210	3	212	1	210	1	212	1	210	1	212
Seck 2010[111] NCT00094770	SAEs	Sitagliptin 100 + metformin >1500	Glipizide 5-20 + metformin >1500	0	588	1	584	0	588	1	584	NR				NR			
Arechavaleta 2011[112] NCT00701090	SAEs	Sitagliptin 100	Glimepiride 1-6	0	516	3	518	0	516	2	518	0	516	1	518	0	516	1	518
NCT01131182[113]	SAEs	Sitagliptin 100	Sulfonylurea	0	507	1	514	0	507	1	514	NR				NR			
Terauchi 2017[114] NCT01183104	SAEs	Sitagliptin 50	Glimepiride 0.5	1	148	0	143	1	148	0	143	NR				NR			
Leiter 2014[115] NCT01098539	SAEs	Sitagliptin 30-50	Albiglutide 30-50	6	246	2	249	5	246	2	249	1	246	0	249	1	246	0	249
Pratley 2011[116] NCT00700817	SAEs	Sitagliptin 100	Liraglutide 1.2	0	219	0	439	0	219	0	221	NR				NR			
			Liraglutide 1.8							0	218								

Zang 2016[117]	SAEs	Sitagliptin 100	Liraglutide 1.8	1	184	0	183	1	184	0	183	NR				NR
NCT02008682																
NCT01519674[118]	SAEs	Sitagliptin + biphasic insulin aspart	Biphasic insulin	2	383	0	192	1	383	0	192	1	190	0	192	NR
			aspart									0	193			
Philis-Tsimikas 2013[119]	SAEs	Sitagliptin 100	Insuline degludec	2	228	1	226	2	228	1	226	NR				NR
NCT01046110			100IU													
NCT00875394[120]	SAEs	Sitagliptin 100 + metformin	Metformin + any	0	36	0	32	0	36	0	9	NR				NR
			non-DPP4-Is					0	23							
			Metformin													
**Vildagliptin therapy**																
NCT00646542[121]	SAEs	Vildagliptin 50	Placebo	0	292	0	227	0	292	0	227	NR				NR
NCT00821977[122]	SAEs	Vildagliptin	Placebo	0	301	0	150	0	301	0	150					NR
Scherbaum 2008[123]	SAEs	Vildagliptin 50	Placebo	0	156	1	150	0	156	1	150	NR				NR
NCT00300287																
Dejager 2007[124]	SAEs	Vildagliptin 50	Placebo	1	472	0	160	0	236	0	160	NR				NR
NCT00099905		Vildagliptin 100						1	236							
McMurray 2013[125]	SAEs	Vildagliptin 100	Placebo	1	128	4	126	1	128	4	126	NR				NR
NCT00894868																
Pan 2012[126]	SAEs	Vildagliptin 50	Placebo	0	294	1	144	0	148	1	144	NR				NR
NCT00822211		Vildagliptin 100						0	146							
CLAF237ADE02[127]	SAEs	Vildagliptin 100	Placebo	0	268	0	134	0	268	0	134					NR
Bosi 2007[128]	SAEs	Vildagliptin 50	Placebo	1	360	1	181	0	177	1	181	NR				NR
NCT00099892		Vildagliptin 100						1	183							
Yang 2015[129]	SAEs	Vildagliptin 50	Placebo	0	143	1	135	0	143	1	135	NR				NR
NCT01357252																
Garber 2008[130]	SAEs	Vildagliptin 50	Placebo	0	339	0	176	0	170	0	113	NR				NR
NCT00099944		Vildagliptin 100						0	169							
Fonseca 2009[131]	SAEs	Vildagliptin 100	Placebo	0	144	0	152	0	144	0	152	NR				NR
NCT00099931																
Bosi 2009[132]	SAEs	Vildagliptin 100	Metformin 1000	0	879	0	292	0	297	0	292	NR				NR

NCT00468039		Vildagliptin 100 + metformin 1000						0	290										
		Vildagliptin 100 + metformin 2000						0	292										
Schweizer 2007[133] NCT00099866	SAEs	Vildagliptin 100	Metformin 2000	1	511	0	249	1	511	0	249	NR				NR			
Pan 2008[134] NCT00110240	SAEs	Vildagliptin 100	Placebo	0	440	0	220	0	440	0	220	NR				NR			
Rosenstock 2009[135] NCT00138619	SAEs	Vildagliptin 100	Rosiglitazone 8	3	393	0	198	3	393	0	198	NR				1	393	0	198
Bolli 2009[136] NCT00237237	SAEs	Vildagliptin 100	Pioglitazone 30	2	295	3	280	2	295	3	280	NR				NR			
Foley 2009[137] NCT00102388	SAEs	Vildagliptin 100	Gliclazide 320	3	546	0	546	3	546	0	546	NR				NR			
Ferrannini 2009[138] NCT00106340	SAEs	Vildagliptin 100	Glimepiride 2	0	1389	7	1383	0	1389	7	1383	NR				NR			
**Total sample size (*n*= 127,424) Total stroke outcomes = 1,188**				734	70,541	754	65,883												

Abbreviations: *AEs*, adverse events; *AHG, anti*- hyperglycaemic drugs; *NR*, not report; *SAEs*, serious adverse events; *VS.*, versus.

Note: ¶ Novel treatment of fixed-dose combination of 2 different class of AHG.

GLP1-RAs

Table S5. Trial stroke outcomes with GLP1-RAs

Study<br>Trial registry	Type of stroke index event	Intervention of interest		Number of strokes				Ischaemic stroke				Haemorrhagic stroke				Fatal stroke			
		GLP1-RAs (mg/day)	Controls (mg/day)	GLP1-RAs		Controls		GLP1-RAs		Controls		GLP1-RAs		Controls		GLP1-RAs		Controls	
				Event	Sample size	Event	Sample size	Event	Sample size	Event	Sample size	Event	Sample size	Event	Sample size	Event	Sample size	Event	Sample size
**Albiglutide therapy**																			
Nauck 2016[139]<br>NCT00849017	SAEs	Albiglutide 30<br>Albiglutide 50	Placebo	3	200	3	101	3	200	3	101	NR				NR			
Hernandez 2018[140]<br>NCT02465515	Fatal or non-fatal 2^{ry} outcome	Albiglutide 30<br>Albiglutide 50	Placebo	94	4731	108	4732	94	4731	108	4732	NR				18	4731	17	4732
Reusch 2014[141]<br>NCT00849056	SAEs	Albiglutide 30	Placebo	0	150	0	151	0	150	0	151	NR				NR			
Nauck 2016[139]<br>NCT00839527	SAEs	Albiglutide 30<br>Albiglutide 50	Placebo	3	200	3	101	3	200	3	101	NR				NR			
Seino 2014[142]<br>NCT01098461	SAEs	Albiglutide 30<br>Albiglutide 50	Placebo	0	200	1	101	0	200	1	101	NR				NR			
Ahrén 2017[108]<br>NCT00838903	SAEs	Albiglutide 30 + metformin	Placebo + metformin<br>Glimepiride 2 + metformin	3	302	2	408	2	302	2	408	0<br>1	302<br>101	0	307	NR			
Home 2014[143]<br>NCT00839527	SAEs	Albiglutide 30	Pioglitazone<br>Placebo	1	271	8	392	0	271	8	392	0<br>1	271<br>277	0	392	NR			
Pratley 2014[144]<br>NCT01128894	SAEs	Albiglutide 50	Liraglutide 1.8	3	404	1	408	3	404	1	408	NR				NR			
Weissman 2014[145]<br>NCT00838916	SAEs	Albiglutide 30	Insulin glargine	4	504	0	241	4	504	0	241	NR				NR			
Rosenstock 2014[146]<br>NCT00976391	SAEs	Albiglutide 50 + insulin glargine	Insulin lispro 4-8 IU + insulin glargine	0	285	0	281	0	285	0	281	NR				NR			

**Dulaglutide therapy**																
Gerstein 2019[147] NCT01394952	Fatal or non-fatal 1^{ry} outcome	Dulaglutide 1.5	Placebo	158	4949	205	4952	135	4949	175	4952	NR	26	4949	33	4952
Wysham 2014[148] NCT01064687	SAEs	Dulaglutide 0.75<br>Dulaglutide 1.5	Placebo	1	559	0	141	1	559	0	141	NR	NR			
Pozzilli 2017[149] NCT02152371	SAEs	Dulaglutide 1.5	Placebo	3	150	0	150	3	150	0	150	NR	NR			
**Exenatide therapy**																
Holman 2017[150] NCT01144338	Fatal or non-fatal 1^{ry} and 2^{ry} outcome	Exenatide 2	Placebo	187	7356	218	7396	187	7356	218	7396	NR	18	7356	25	7396
Gao 2009[151] NCT00324363	SAEs	Exenatide 10-20µg	Placebo	0	234	1	232	0	234	1	232	NR	NR			
Gill 2010[152] NCT00516074	SAEs	Exenatide 5-10µg<br>Placebo	Placebo	0	28	0	26	0	28	0	26	NR	NR			
NCT00603239[153]	SAEs	Exenatide 10-20µg<br>Placebo	Placebo	1	111	0	54	1	111	0	54	NR	NR			
Wysham 2014[148] NCT01064687	SAEs	Exenatide 10-20µg<br>Placebo	Placebo	0	276	0	141	0	276	0	141	NR	NR			
Gadde 2017[82] NCT01652729	SAEs	Exenatide 2µg<br>Placebo	Placebo	1	181	0	61	1	181	0	61	NR	NR			
Gallwitz 2012[154] NCT00359762	SAEs	Exenatide 10-50µg<br>Exenatide + TZDs<br>Exenatide + glimepiride 1	Glimepiride	3	827	0	508	3	827	0	508	NR	NR			
Buse 2013[155] NCT01029886	SAEs	Exenatide 2µg	Liraglutide 1.8	1	461	2	450	1	461	2	450	NR	0	461	1	450
Russel-Jones 2012[99] NCT00676338	SAEs	Exenatide 2µg	Metformin<br>Pioglitazone	0	248	1	409	0	248	1	409	NR	NR			

Bergenstal 2010[104] NCT00637273	SAEs	Exenatide 100μg	Pioglitazone 45	0	160	1	165	0	160	1	165	NR				NR			
Nauck 2007[156] NCT00082407	SAEs	Exenatide 5-10μg	Insulin aspart	0	253	1	248	0	253	1	248	NR				NR			
Inagaki 2012[157] NCT00935532	SAEs	Exenatide 2μg	Insulin glargine 4 IU	1	215	0	212	1	215	0	212	NR				NR			
**Liraglutide therapy**																			
Marso 2016[158] NCT01179048	Fatal and non-fatal 1ry and 2ry outcome	Liraglutide	Placebo	173	4668	199	4672	159	4668	177	4672	NR				16	4668	25	4672
Seino 2016[159] NCT01572740	SAEs	Liraglutide	Placebo	1	127	1	130	1	127	1	130	NR				NR			
Kaku 2011[160] NCT00393718	SAEs	Liraglutide 0.9μg	Glibenclamide	0	268	2	132	0	268	2	132	NR				NR			
Bailey 2016[161] NCT01907854	SAEs	Liraglutide 0.6-1.8μg	Sitagliptin 100	0	202	1	204	0	202	1	204	NR				NR			
Lingvay 2016[162] NCT01952145	SAEs	Liraglutide 0.6μg/insulin degludec 16 units	Insulin degludec 16 units	1	278	2	279	1	278	1	279	0	278	1	279	0	278	1	279
NCT01117350[163]	SAEs	Liraglutide 0.6-1.8μg	Insulin glargine	2	481	3	644	2	481	3	644	NR				NR			
**Lixisenatide therapy**																			
Pfeffer 2015[164] NCT01147250	Fatal and non-fatal 1ry and 2ry outcome	Lixisenatide	Placebo	67	3034	60	3034	54	3034	49	3034	NR				NR			
**Semaglutide therapy**																			
Marso 2016[165] NCT01720446	Non-fatal 1ry and 2ry outcome	Semaglutide	Placebo	27	1648	44	1649	25	1648	37	1649	2	1648	4	1649	NR			
Husain 2019[166] NCT02692716	Non-fatal 1ry and 2ry outcome	Semaglutide	Placebo	12	1591	16	1592	12	1591	16	1592	NR				NR			
**Taspoglutide therapy**																			
Raz 2012[167]	SAEs	Taspoglutide 10	Placebo	1	245	0	90	1	245	0	90	NR				NR			

NCT00744926		Taspoglutide 20																	
NCT01018173[168]	SAEs	Taspoglutide 10	Placebo	0	1059	0	1059	0		0		NR				NR			
		Taspoglutide 20																	
Bergenstal 2012[81]	SAEs	Taspoglutide 10	Placebo	0	379	0	90	0	379	0	90	NR				NR			
NCT00754988		Taspoglutide 20																	
Hollander 2012[169]	SAEs	Taspoglutide 20	Placebo	0	154	0	150	0	154	0	150	NR				NR			
NCT00823992																			
Henry 2012[170]	SAEs	Taspoglutide 10	Placebo	0	223	1	101	0	223	1	101	NR				NR			
NCT00744367		Taspoglutide 20																	
Pratley 2012[171]	AEs	Taspoglutide 10	Placebo	0	494	0	257	0	494	0	257	NR				NR			
NCT00909597		Taspoglutide 20	Pioglitazone																
Rosenstock 2013[172]	Non-fatal 1ry	Taspoglutide 10	Exenatide 20µg	2	788	0	385	1	788	0	385	1	394	0	385	1	394	0	385
NCT00717457	and 2ry outcome	Taspoglutide 20										0	394			0	394		
Nauck 2013[173]	SAEs	Taspoglutide 10	Insulin glargine 10 IU	0	715	0	322	0	715	0	322	NR				NR			
NCT00755287		Taspoglutide 20																	
**Total sample size (*n*=76,460)**				753	39,609	884	36,851												
**Total stroke outcomes = 1,637**																			

Abbreviations: *AEs*, adverse events; *AHG, anti-* hyperglycaemic drugs; *NR*, not report; *SAEs*, serious adverse events; *VS.*, versus.

Note: ¶novel treatment of fixed-dose combination of 2 different class of AHG.

SGLT2-Is

Table S6. Trial stroke outcomes with SGLT2-Is

Study<br>Trial registry	Type of stroke index event	Intervention of interest		Number of strokes				Ischaemic stroke				Haemorrhagic stroke				Fatal stroke			
		SGLT2-Is (mg/day)	Controls (mg/day)	SGLT2-Is		Controls		SGLT2-Is		Controls		SGLT2-Is		Controls		SGLT2-Is		Controls	
				Event	Sample size	Event	Sample size	Event	Sample size	Event	Sample size	Event	Sample size	Event	Sample size	Event	Sample size	Event	Sample size
**Canagliflozin therapy**																			
Inagaki 2013[174]	SAEs	Canagliflozin 50	Placebo	0	307	0	75	0	82	0	75	NR				NR			
NCT01022112		Canagliflozin 100						0	74										
		Canagliflozin 200							76										
		Canagliflozin 300							75										
Inagaki 2014[175]	SAEs	Canagliflozin 100	Placebo	0	179	0	93	0	90	0	93	NR				NR			
NCT01413204		Canagliflozin 200							89										
Stenlöf 2012[176]	SAEs	Canagliflozin 100	Placebo	0	392	2	192	0	392	1	192	0	195	1	192	NR			
NCT01081834		Canagliflozin 200										0	197						
Weir 2014[177]	SAEs	Canagliflozin 100	Placebo	3	358	1	78	1	80	1	78	NR				NR			
NCT01064414		Canagliflozin 200						2	178										
Sha 2014[178]	SAEs	Canagliflozin 300	Placebo	0	17	0	18	0	17	0	18	NR				NR			
NCT01483781																			
Qiu 2014[179]	SAEs	Canagliflozin 50	Placebo	0	186	0	93	0	93	0	93	NR				NR			
NCT01340664		Canagliflozin 150							93										
Rosenstock 2012[83]	SAEs	Canagliflozin 50	Placebo	0	321	0	65	0	64	0	65	NR				NR			
NCT00642278		Canagliflozin 100						0	64										
		Canagliflozin 200						0	65										
		Canagliflozin 300						0	64										
		Canagliflozin 600						0	64										
Ji 2015[180]	SAEs	Canagliflozin 100	Placebo	1	450	1	226	1	223	1	226	NR				NR			
NCT01381900		Canagliflozin 300						0	227										

Wilding 2013[181]	SAEs	Canagliflozin 100	Placebo	1	313	0	156	1	157	0	156	NR				NR			
NCT01106625		Canagliflozin 300							156										
Neal 2017[182]	Non-fatal 1^ry	Canagliflozin 100	Placebo	166	5795	147	4347	146	5795	128	4347	NR				20	5795	19	4347
NCT01032629	and 2^ry outcome	Canagliflozin 300																	
NCT01989754																			
Fulcher 2015[183]	SAEs	Canagliflozin 100	Placebo	0	82	0	45	0	42	0	45	NR				NR			
NCT01032629		Canagliflozin 300						0	40										
Cai 2018[184]	SAEs	Canagliflozin 100	Placebo	0	108	2	108	0	108	2	108	NR				NR			
NCT02025907		Canagliflozin 300																	
Perkovic 2019[185]	Non-fatal 1^ry	Canagliflozin 100	Placebo	62	2202	80	2199	53	2202	66	2199	NR				9	2202	14	2199
NCT02065791	and 2^ry outcome																		
Bode 2015[186]	SAEs	Canagliflozin 100	Placebo	5	208	0	104	4	104	0	104	NR				NR			
NCT01106651		Canagliflozin 300						1	104										
Lavalle-González	SAEs	Canagliflozin 100	Placebo	4	735	1	549	2	368	1	366	NR				NR			
2013[187]		Canagliflozin 300	Sitagliptin 100 +					2	367	0	183								
NCT01106677			placebo																
Forst 2014[188]	SAEs	Canagliflozin 100	Placebo + sitagliptin 100	1	227	0	115	0	113	0	115	NR				NR			
NCT01106690		Canagliflozin 300						1	114										
Rosenstock 2016[189]	SAEs	Canagliflozin 100	Metformin	2	949	0	237	0	237	0	237	NR				NR			
NCT01809327		Canagliflozin 300						1	238										
		Canagliflozin 100 + metformin						0	237										
		Canagliflozin 300 + metformin						1	237										
Cefalu 2013[190]	SAEs	Canagliflozin 100	Glimepiride	9	968	3	482	3	483	3	482	0	483	0	482	NR			
NCT00968812		Canagliflozin 300						5	485			1	485						
Schernthaner 2013[191]	SAEs	Canagliflozin 300	Sitagliptin 100	1	377	1	378	1	377	1	378	NR				NR			
NCT01137812																			
**Dapagliflozin therapy**																			
Wiviott 2019[192]	Non-fatal 1^ry	Dapagliflozin 5	Placebo	235	8582	231	8578	235	8582	231	8578	NR				NR			
NCT01730534	and 2^ry outcome																		
Kaku 2013[193]	SAEs	Dapagliflozin 1	Placebo	0	225	0	54	0	59	0	54	NR				NR			

NCT00972244		Dapagliflozin 2.5						0	56							
		Dapagliflozin 5						0	58							
		Dapagliflozin 10						0	52							
Bailey 2012[194]	SAEs	Dapagliflozin	Placebo	0	214	0	68	0	72	0	68	NR				NR
NCT00736879		Dapagliflozin 2.5						0	74							
		Dapagliflozin 5						0	68							
Kaku 2014[195]	SAEs	Dapagliflozin 5	Placebo	0	174	2	87	0	86	1	87	0	86	1	87	NR
NCT01294423		Dapagliflozin 10						0	88			0	88			
Bailey 2014[196]	SAEs	Dapagliflozin 2.5	Placebo	1	199	0	75	1	165	0	75	NR				NR
NCT00528372		Dapagliflozin 5						0	64							
		Dapagliflozin 10						0	70							
Schumm-Draeger 2015[197]	SAEs	Dapagliflozin 5 + metformin	Placebo + metformin	0	299	0	101	0	100	0	101	NR				NR
NCT01217892		Dapagliflozin 10 + metformin						0	100							
		Dapagliflozin 10 + metformin						0	99							
Bailey 2010[198]	SAEs	Dapagliflozin 2.5	Placebo	1	409	2	137	1	137	2	137	NR				NR
NCT00528879		Dapagliflozin 5						0	137							
		Dapagliflozin 10						0	135							
Bolinder 2014[199]	SAEs	Dapaglilozin 10	Placebo	1	91	0	91	1	91	0	91	NR				NR
NCT00855166																
Ji 2014[200]	SAEs	Dapagliflozin 5	Placebo	1	261	0	132	0	128	0	132	NR				NR
NCT01095653		Dapagliflozin 10						1	133							
Henry 2012[201] (Study1)	SAEs	Dapagliflozin 5	Metformin + placebo	0	397	1	201	0	203	1	201	NR				NR
NCT00643851		Dapagliflozin 5 + metformin						0	194							
Henry 2012 (Study2)[201]	SAEs	Dapagliflozin 10	Metformin + placebo	0	430	0	208	0	219	0	208	NR				NR
NCT00859898		Dapagliflozin 10 + metformin							211							
Heerspink 2013[202]	SAEs	Dapaglilozin 10	Placebo	0	24	0	25	0	24	0	25	NR				NR
NCT00976495																
Matthaei 2015[203]	SAEs	Dapaglilozin 10	Placebo	0	109	0	109	0	109	0	109	NR				NR
NCT01392677																

Mathieu 2015[92]	SAEs	Dapaglilozin 10	Placebo	0	160	0	160	0	160	0	160	NR	NR			
NCT01646320																
Jabbour 2014[204]	SAEs	Dapaglilozin 10	Placebo	0	225	0	226	0	225	0	226	RN	NR			
NCT00984867																
Wilding 2012[205]	SAEs	Dapagliflozin 10 + insulin	Placebo	0	48	0	23	0	48	0	23	RN	NR			
NCT00357370		Dapagliflozin 20 + insulin														
Mudaliar 2014[206]	SAEs	Dapaglilozin 5	Placebo	0	23	0	21	0	23	0	21	NR	NR			
NCT00831779[207]	SAEs	Dapaglilozin 5	Placebo	0	23	0	21	0	23	0	21	NR	NR			
Strojek 2011[208]	SAEs	Dapagliflozin 2.5 + insulin	Placebo	2	450	0	146	1	154	0	146	NR	0	154	0	146
NCT00680745		Dapagliflozin 5 + insulin						0	145				0	145		
		Dapagliflozin 10 + insulin						1	151				1	151		
Kohan 2014[209]	SAEs	Dapagliflozin 5	Placebo	1	168	0	84	0	83	0	84	NR	NR			
NCT00663260		Dapagliflozin 10						1	85							
Rosenstock 2012[210]	SAEs	Dapagliflozin 5	Placebo	0	281	0	139	0	141	0	139	NR	NR			
NCT00683878		Dapagliflozin 10							140							
Weber 2016[211]	SAEs	Dapaglilozin 10	Placebo	0	225	0	224	0	225	0	224	NR	RN			
NCT01195662																
NCT01137474[212]	SAEs	Dapagliflozin 2.5	Placebo	1	633	1	311	1	166	1	311	NR	NR			
		Dapagliflozin 5						0	165							
		Dapagliflozin 10						0	302							
Wilding 2014[213]	SAEs	Dapagliflozin 2.5	Placebo	2	610	3	197	1	202	3	197	NR	NR			
NCT00673231		Dapagliflozin 5						0	212							
		Dapagliflozin 10						1	196							
Yang 2018[214]	SAEs	Dapagliflozin 10	Placebo	1	139	0	133	1	139	0	133	NR	NR			
NCT02096705																
Cefalu 2015[215]	SAEs	Dapagliflozin 10	Placebo	2	460	2	462	2	460	2	462	NR	0	460	1	462
NCT01031680																
Leiter 2014[216]	SAEs	Dapagliflozin 10	Placebo	1	482	4	483	1	482	4	483	NR	NR			
NCT01042977																
List 2009[217]	SAEs	Dapagliflozin 2.5	Placebo	0	253	0	106	0	53	0	55	NR	NR			

NCT00263276		Dapagliflozin 5	Metformin					0	55	0	51								
		Dapagliflozin 10						0	40										
		Dapagliflozin 20						0	55										
		Dapagliflozin 50						0	50										
Nauck 2014[218] NCT00660907	SAEs	Dapagliflozin 10 + metformin	Glipizide 20 + metformin	2	406	0	408	2	406	0	408	NR				NR			
Rosenstock 2015[219] NCT01606007	SAEs	Dapagliflozin 10 + saxagliptin 10 + metformin	Saxagliptin 5 + metformin	1	358	0	176	1	358	0	176	NR				NR			
		Dapagliflozin 10 + metformin																	
**Empagliflozin therapy**																			
Kadowaki 2015[220] NCT01193218	SAEs	Empagliflozin 5	Placebo	1	752	0	109	0	110	0	109	NR				NR			
		Empagliflozin 10						0	267										
		Empagliflozin 25						1	265										
		Empagliflozin 50						0	110										
NCT01649297[221]	SAEs	Empagliflozin 5	Placebo	2	876	1	107	0	219	1	107	NR				NR			
		Empagliflozin 10						0	220										
		Empagliflozin 12.5						0	219										
		Empagliflozin 25						2	213										
Merker 2015[222] NCT01289990; NCT01159600	Non-fatal 1ry and 2ry outcome	Empagliflozin 10	Placebo	1	430	0	207	0	217	0	207	NR				NR			
		Empagliflozin 25						1	213										
Ha¨ring 2013[223] NCT01159600	SAEs	Empagliflozin 10	Placebo	1	359	0	141	1	152	0	141	NR				NR			
		Empagliflozin 25						0	139										
		Empagliflozin 25 (open-label)						0	68										
Rosenstock 2015[224] NCT01011868	SAEs	Empagliflozin 10	Placebo	5	324	1	170	1	169	0	170	1	169	1	170	NR			
		Empagliflozin 25						3	155			0	155						
Zinman 2015[225] NCT01131676	Fatal and non-fatal 1ry and 2ry outcome	Empagliflozin 10	Placebo	164	4687	69	2333	150	4687	60	2333	NR				14	4687	9	2333
		Empagliflozin 25																	
Barnett 2014[176]	SAEs	Empagliflozin 10	Placebo	5	419	3	319	0	98	3	319	NR				NR			

NCT01164501		Empagliflozin 25						5	321				
Rosenstock 2014[226]	SAEs	Empagliflozin 10	Placebo	1	375	1	188	1	186	1	188	NR	NR
NCT01306214		Empagliflozin 25						0	189				
Kovacs 2014[227]	SAEs	Empagliflozin 10	Placebo	1	333	0	165	0	165	0	165	NR	NR
NCT01210001		Empagliflozin 25						1	168				
Kovacs 2014[227]	SAEs	Empagliflozin 10	Placebo	0	333	2	165	0	165	2	165	NR	NR
NCT01289990		Empagliflozin 25						0	168				
Tikkanen 2015[228]	SAEs	Empagliflozin 10	Placebo	0	552	0	272	0	276	0	272	NR	NR
NCT01370005		Empagliflozin 25						0	276				
Ferrannini 2013[229]	SAEs	Empagliflozin 5	Placebo	0	244	0	162	0	81	0	162	NR	NR
NCT00789035		Empagliflozin 10						0	81				
		Empagliflozin 25						0	82				
Roden 2013[75]	SAEs	Empagliflozin 10	Placebo	1	534	1	229	1	224	1	229	NR	NR
NCT01177813		Empagliflozin 25						0	223				
		Empagliflozin 25						0	87				
Søfteland 2017[26]	SAEs	Empagliflozin 10	Placebo	0	222	0	110	0	112	0	110	NR	NR
NCT01734785		Empagliflozin 25							110				
Rosenstock 2013[84]	SAEs	Empagliflozin 1	Placebo	0	136	0	26	0	21	0	26	NR	NR
NCT00749190		Empagliflozin 5						0	26				
		Empagliflozin 10						0	30				
		Empagliflozin 25						0	25				
		Empagliflozin 50						0	34				
Ferrannini 2013[229]	SAEs	Empagliflozin 10	Metformin	1	215	0	56	1	106	0	56	NR	NR
(Study 1)		Empagliflozin 25						0	109				
NCT00881530													
Hadjadj 2016[230]	SAEs	Empagliflozin 10	Metformin 1000	1	1032	1	341	0	172	0	171	NR	NR
NCT01719003		Empagliflozin 25	Metformin 2000					0	167	1	170		
		Empagliflozin 10 + metformin 1000						0	129				
		Empagliflozin 10 + metformin 2000						0	171				
		Empagliflozin 2 + metformin 1000						0	170				

		Empagliflozin 25 + metformin 2000						1	170							
		Empagliflozin 25+ metformin						0	53							
Araki 2015[231]	SAEs	Empagliflozin 10 + sulfonylurea	Metformin	3	1097	0	63	0	136	0	63	NR				NR
NCT01368081		Empagliflozin 25 + sulfonylurea						0	137							
		Empagliflozin 10 + glinide						0	70							
		Empagliflozin 25 + glinide						0	70							
		Empagliflozin 10 + biguanide						0	68							
		Empagliflozin 25 + biguanide						0	65							
		Empagliflozin 10 + TZDs						1	137							
		Empagliflozin 25 + TZDs						1	136							
		Empagliflozin 10 + α-glucosidase-Is						0	69							
		Empagliflozin 25 + α-glucosidase-Is						0	70							
		Empagliflozin 10 + DPP4-Is						1	68							
		Empagliflozin 25 + DPP4-Is						0	71							
Ridderstra°le 2014[232]	SAEs	Empagliflozin 25	Glimepiride 1-4mg	14	765	15	780	14	765	12	780	0	765	3	780	NR
NCT01167881																
DeFronzo 2015[233]	SAEs	Empagliflozin 10	Linagliptin 5	6	546	1	128	2	137	1	128	0	137	0	128	NR
NCT01422876		Empagliflozin 25						0	140			0	140			
		Empagliflozin 10 + linagliptin 5						2	135			1	135			
		Empagliflozin 25 + linagliptin 5						1	134			0	134			
Ferrannini 2013[229]	SAEs	Empagliflozin 10	Sitagliptin	3	332	3	56	1	166	3	56	NR				NR
(Study 2)		Empagliflozin 25						2	166							
NCT00881530																
**Ertugliflozin therapy**																
Cannon 2020[234]	Fatal or non-fatal 2ry outcome	Ertugliflozin 5-10	Placebo	185	5499	87	2747	157	5499	78	2747	NR				NR
NCT01986881	SAEs	Ertugliflozin 5						29	2746	41	2745					
		Ertugliflozin 15						40	2747							
Dagogo-Jack 2018[235]	SAEs	Ertugliflozin 5	Placebo	2	309	0	153	1	156	0	153	0	156	0	153	NR
NCT02036515		Ertugliflozin 15							153			1	153			

Miller 2018[236]	SAEs	Ertugliflozin 5 + sitagliptin 100	Placebo	0	219	0	429	0	98	1	97	NR	NR
NCT02226003		Ertugliflozin 15 + sitagliptin 100						0	96				
Amin 2015[94]	SAEs	Ertugliflozin 1	Placebo	0	194	0	97	0	54	0	54	NR	NR
NCT01059825		Ertugliflozin 5	Metformin					0	55	0	375		
		Ertugliflozin 10						0	55				
		Ertugliflozin 25						0	55				
Ipragliflozin therapy													
Wilding 2013[237]	SAEs	Ipragliflozin 12.5	Placebo	0	276	0	66	0	69	0	66	NR	NR
NCT01117584		Ipragliflozin 50						0	68				
		Ipragliflozin 150						0	67				
		Ipragliflozin 300						0	72				
Kashiwagi 2015[238]	SAEs	Ipragliflozin 50	Placebo	0	112	0	56	0	112	0	56	NR	NR
NCT01135433													
Lu 2016[239]	SAEs	Ipragliflozin	Placebo	0	87	0	83	0	87	0	83	NR	NR
NCT01505426													
**Total sample size**				904	59,688	669	39,050						
**(*n*=84,195)**													
**Total stroke outcomes =**													
**1,573**													

Abbreviations: *AEs*, adverse events; *AHG*, *anti*- hyperglycaemic drugs; *NR*, not report; *SAEs*, serious adverse events; *vs.*, versus.

Note: [¶]novel treatment of fixed-dose combination of 2 different class of AHG.

Meta-analysis and GRADE outcomes

There were no clinically meaningful differences in stroke rate in the 158 trials that reported stroke outcomes as non-serious or serious events (87 trials with dipeptidyl peptidase-4 inhibitors (DPP4-Is), 28 trials with glucagon-like peptide-1 receptor agonists (GLP1-RAs), and 43 trials with sodium-glucose cotransporter-2 inhibitors (SGLT2-Is)). Most of these events were unlikely to be related to trial interventions in the judgement of the study investigators. However, in the 16 trials that reported stroke from composite major adverse cardiovascular events (MACEs) endpoints, the magnitude of difference for ischaemic or haemorrhagic events was low without significant benefit of reduction across groups or placebo for DPP4-Is (alogliptin,[5] linagliptin,[22] linagliptin,[44] omarigliptin,[45] saxagliptin,[59] and sitagliptin),[89] GLP1-RAs, (albiglutide,[140] exenatide,[150] liraglutide,[158] lixisenatide,[164] and semaglutide),[166] and SGLT2-Is (canagliflozin,[182,185] dapagliflozin,[192] empagliflozin,[225] and ertugliflozin).[234] In the other remaining three trials, a significant reduction in non-fatal stroke rate was observed with DPP4-Is (linagliptin)[43] and in MACE rates, including fatal and non-fatal strokes with GLP1-RAs (dulaglutide[147] and semaglutide).[165]

Stroke risk by clinical trial baseline characteristic and stroke risk by subtypes

SGLT2-Is and stroke by baseline characteristic

Use of SGLT2-Is did not decrease stroke risk in patients with T2DM at risk of CVD or CKD (RR, 0.96; 95% CI, 0.87−1.06; P=0.42) versus non-SGLT2-Is using fixed- and random-effect models (Figure S and Figure S). The GRADE scores were moderate (Table S7).

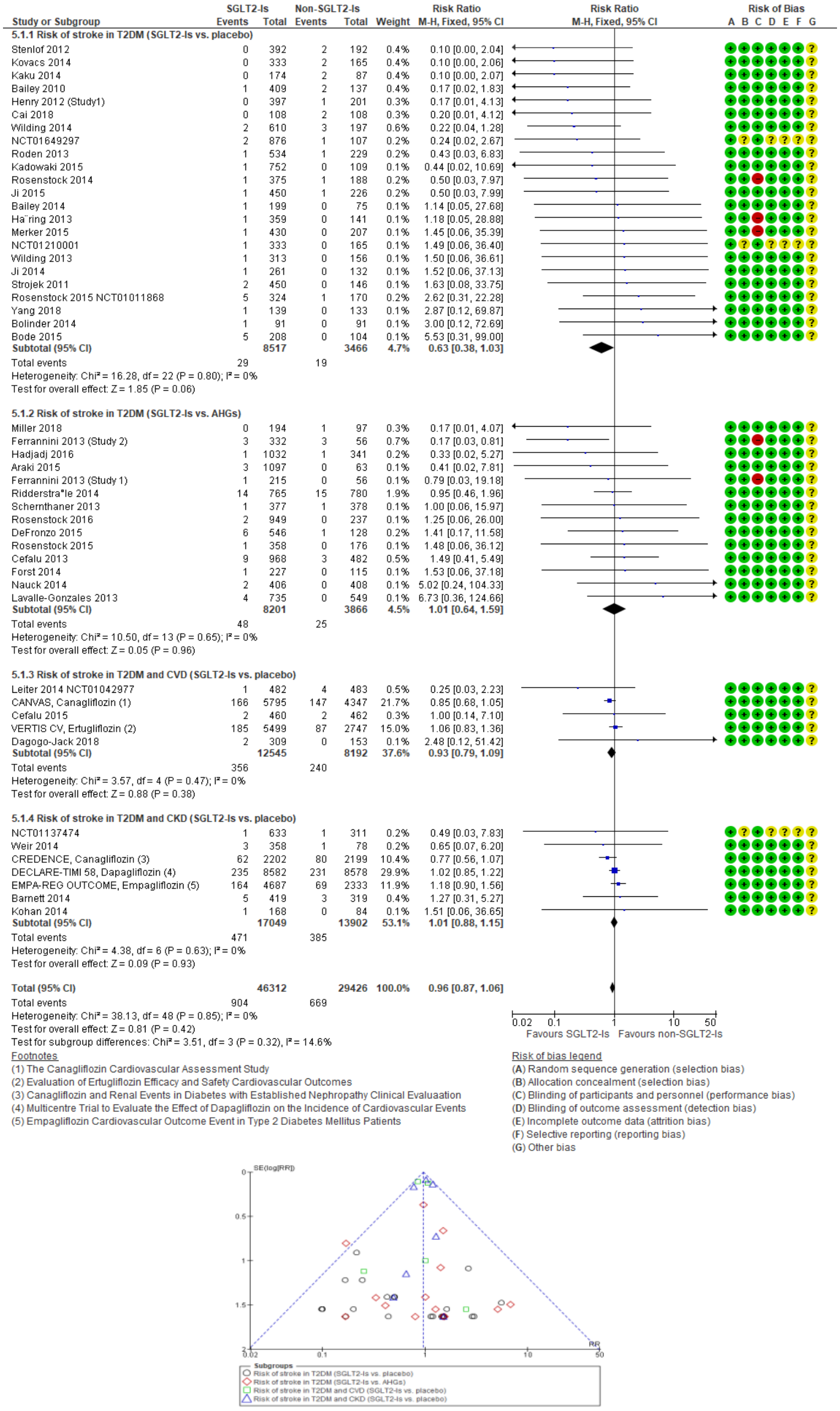

Figure S2. Forest and funnel plot of SGLT2-Is and stroke by baseline characteristic, Fixed-effect model.

Study or Subgroup	SGLT2-Is Events	SGLT2-Is Total	Non-SGLT2-Is Events	Non-SGLT2-Is Total	Weight	Risk Ratio M-H, Random, 95% CI
**5.1.1 Risk of stroke in T2DM (SGLT2-Is vs. placebo)**						
Stenlof 2012	0	392	2	192	0.1%	0.10 [0.00, 2.04]
Kovacs 2014	0	333	2	165	0.1%	0.10 [0.00, 2.06]
Kaku 2014	0	174	2	87	0.1%	0.10 [0.00, 2.07]
Bailey 2010	1	409	2	137	0.2%	0.17 [0.02, 1.83]
Henry 2012 (Study1)	0	397	1	201	0.1%	0.17 [0.01, 4.13]
Cai 2018	0	108	2	108	0.1%	0.20 [0.01, 4.12]
Wilding 2014	2	610	3	197	0.3%	0.22 [0.04, 1.28]
NCT01649297	2	876	1	107	0.2%	0.24 [0.02, 2.67]
Roden 2013	1	534	1	229	0.1%	0.43 [0.03, 6.83]
Kadowaki 2015	1	752	0	109	0.1%	0.44 [0.02, 10.69]
Rosenstock 2014	1	375	1	188	0.1%	0.50 [0.03, 7.97]
Ji 2015	1	450	1	226	0.1%	0.50 [0.03, 7.99]
Bailey 2014	1	199	0	75	0.1%	1.14 [0.05, 27.68]
Ha¨ring 2013	1	359	0	141	0.1%	1.18 [0.05, 28.88]
Merker 2015	1	430	0	207	0.1%	1.45 [0.06, 35.39]
NCT01210001	1	333	0	165	0.1%	1.49 [0.06, 36.40]
Wilding 2013	1	313	0	156	0.1%	1.50 [0.06, 36.61]
Ji 2014	1	261	0	132	0.1%	1.52 [0.06, 37.13]
Strojek 2011	2	450	0	146	0.1%	1.63 [0.08, 33.75]
Rosenstock 2015 NCT01011868	5	324	1	170	0.2%	2.62 [0.31, 22.28]
Yang 2018	1	139	0	133	0.1%	2.87 [0.12, 69.87]
Bolinder 2014	1	91	0	91	0.1%	3.00 [0.12, 72.69]
Bode 2015	5	208	0	104	0.1%	5.53 [0.31, 99.00]
**Subtotal (95% CI)**		**8517**		**3466**	**2.9%**	**0.57 [0.32, 1.02]**
Total events	29		19			
Heterogeneity: Tau² = 0.00; Chi² = 16.28, df = 22 (P = 0.80); I² = 0%						
Test for overall effect: Z = 1.89 (P = 0.06)						
**5.1.2 Risk of stroke in T2DM (SGLT2-Is vs. AHGs)**						
Miller 2018	0	194	1	97	0.1%	0.17 [0.01, 4.07]
Ferrannini 2013 (Study 2)	3	332	3	56	0.4%	0.17 [0.03, 0.81]
Hadjadj 2016	1	1032	1	341	0.1%	0.33 [0.02, 5.27]
Araki 2015	3	1097	0	63	0.1%	0.41 [0.02, 7.81]
Ferrannini 2013 (Study 1)	1	215	0	56	0.1%	0.79 [0.03, 19.18]
Ridderstra°le 2014	14	765	15	780	1.9%	0.95 [0.46, 1.96]
Schernthaner 2013	1	377	1	378	0.1%	1.00 [0.06, 15.97]
Rosenstock 2016	2	949	0	237	0.1%	1.25 [0.06, 26.00]
DeFronzo 2015	6	546	1	128	0.2%	1.41 [0.17, 11.58]
Rosenstock 2015	1	358	0	176	0.1%	1.48 [0.06, 36.12]
Cefalu 2013	9	968	3	482	0.6%	1.49 [0.41, 5.49]
Forst 2014	1	227	0	115	0.1%	1.53 [0.06, 37.18]
Nauck 2014	2	406	0	408	0.1%	5.02 [0.24, 104.33]
Lavalle-Gonzales 2013	4	735	0	549	0.1%	6.73 [0.36, 124.66]
**Subtotal (95% CI)**		**8201**		**3866**	**4.3%**	**0.90 [0.55, 1.46]**
Total events	48		25			
Heterogeneity: Tau² = 0.00; Chi² = 10.50, df = 13 (P = 0.65); I² = 0%						
Test for overall effect: Z = 0.42 (P = 0.67)						
**5.1.3 Risk of stroke in T2DM and CVD (SGLT2-Is vs. placebo)**						
Leiter 2014 NCT01042977	1	482	4	483	0.2%	0.25 [0.03, 2.23]
CANVAS, Canagliflozin (1)	166	5795	147	4347	21.1%	0.85 [0.68, 1.05]
Cefalu 2015	2	460	2	462	0.3%	1.00 [0.14, 7.10]
VERTIS CV, Ertugliflozin (2)	185	5499	87	2747	16.1%	1.06 [0.83, 1.36]
Dagogo-Jack 2018	2	309	0	153	0.1%	2.48 [0.12, 51.42]
**Subtotal (95% CI)**		**12545**		**8192**	**37.8%**	**0.93 [0.79, 1.10]**
Total events	356		240			
Heterogeneity: Tau² = 0.00; Chi² = 3.57, df = 4 (P = 0.47); I² = 0%						
Test for overall effect: Z = 0.87 (P = 0.39)						
**5.1.4 Risk of stroke in T2DM and CKD (SGLT2-Is vs. placebo)**						
NCT01137474	1	633	1	311	0.1%	0.49 [0.03, 7.83]
Weir 2014	3	358	1	78	0.2%	0.65 [0.07, 6.20]
CREDENCE, Canagliflozin (3)	62	2202	80	2199	9.5%	0.77 [0.56, 1.07]
DECLARE-TIMI 58, Dapagliflozin (4)	235	8582	231	8578	31.4%	1.02 [0.85, 1.22]
EMPA-REG OUTCOME, Empagliflozin (5)	164	4687	69	2333	13.2%	1.18 [0.90, 1.56]
Barnett 2014	5	419	3	319	0.5%	1.27 [0.31, 5.27]
Kohan 2014	1	168	0	84	0.1%	1.51 [0.06, 36.65]
**Subtotal (95% CI)**		**17049**		**13902**	**55.0%**	**1.01 [0.88, 1.15]**
Total events	471		385			
Heterogeneity: Tau² = 0.00; Chi² = 4.38, df = 6 (P = 0.63); I² = 0%						
Test for overall effect: Z = 0.08 (P = 0.94)						
**Total (95% CI)**		**46312**		**29426**	**100.0%**	**0.96 [0.86, 1.06]**
Total events	904		669			
Heterogeneity: Tau² = 0.00; Chi² = 38.13, df = 48 (P = 0.85); I² = 0%						
Test for overall effect: Z = 0.89 (P = 0.37)						
Test for subgroup differences: Chi² = 3.73, df = 3 (P = 0.29), I² = 19.6%						

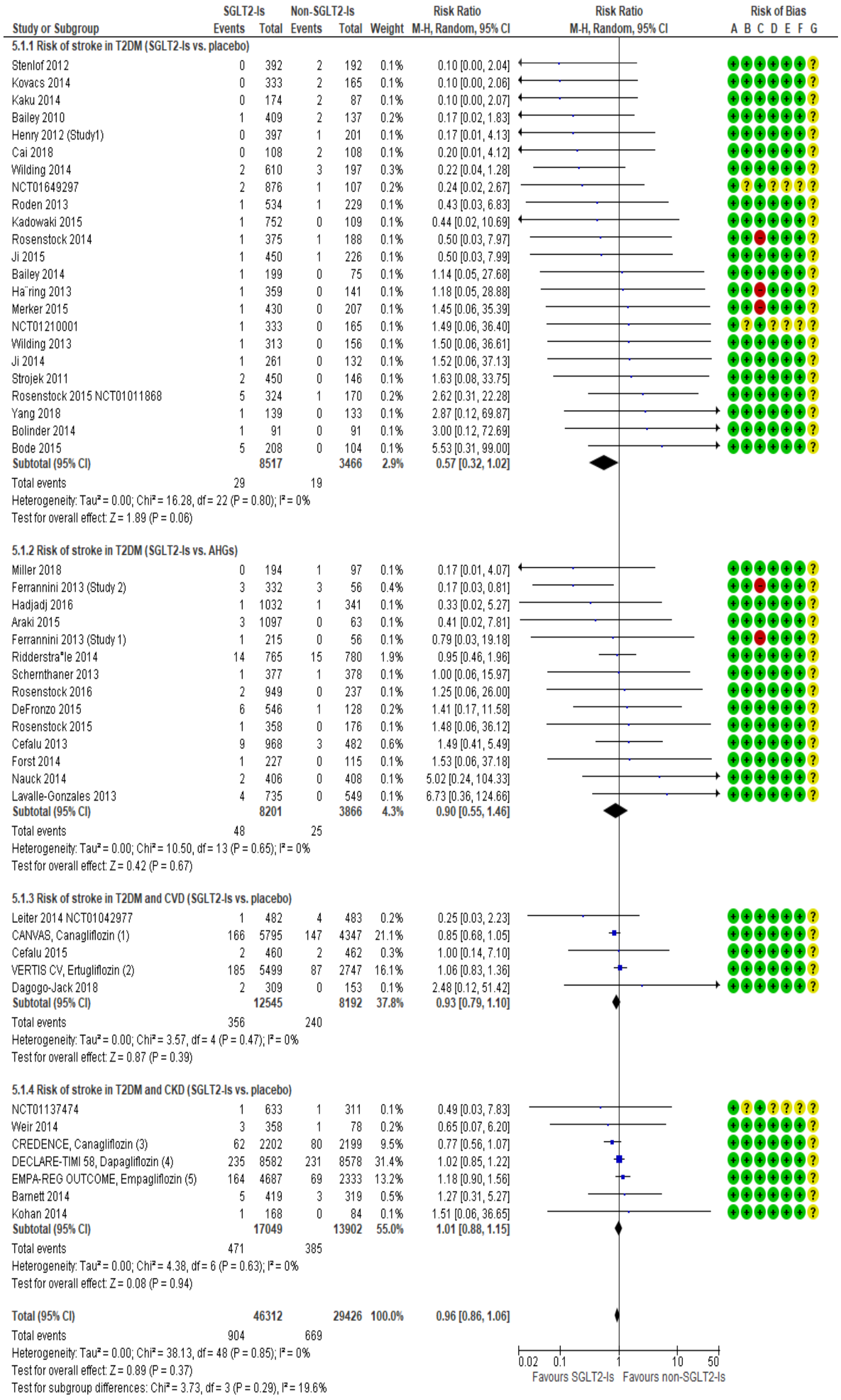

Footnotes
(1) The Canagliflozin Cardiovascular Assessment Study
(2) Evaluation of Ertugliflozin Efficacy and Safety Cardiovascular Outcomes
(3) Canagliflozin and Renal Events in Diabetes with Established Nephropathy Clinical Evaluaation
(4) Multicentre Trial to Evaluate the Effect of Dapagliflozin on the Incidence of Cardiovascular Events
(5) Empagliflozin Cardiovascular Outcome Event in Type 2 Diabetes Mellitus Patients

Risk of bias legend
(A) Random sequence generation (selection bias)
(B) Allocation concealment (selection bias)
(C) Blinding of participants and personnel (performance bias)
(D) Blinding of outcome assessment (detection bias)
(E) Incomplete outcome data (attrition bias)
(F) Selective reporting (reporting bias)
(G) Other bias

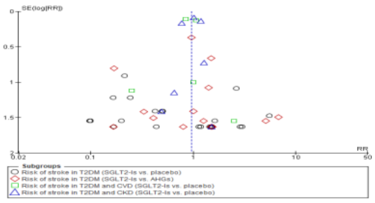

Figure S3. Forest and funnel plot of SGLT2-Is and stroke by baseline characteristic, Random-effect model.

Table S7. GRADE evidence profile of SGLT2-Is and stroke by baseline characteristic

Certainty assessment							Summary of findings				
№ of participants (trials)	Risk of bias	Inconsistency	Indirectness	Imprecision	Publication bias	Overall certainty of evidence	Study event rates (%)		Relative effect (95% CI)	Anticipated absolute effects	
							With placebo / controls	With SGLT2-Is		Risk with placebo / controls	Risk difference with SGLT2-Is
**Risk of stroke in T2DM by baseline characteristic (follow-up: range 12-318 weeks)**											
75538 (49 RCTs)	serious[a]	not serious	not serious	not serious	none	⊕⊕⊕◯ MODERATE	669/29426 (2.3%)	904/46312 (2.0%)	RR 0.96 (0.87 to 1.06)	2 per 100	0 fewer per 100 (from 0 fewer to 0 fewer)
**Risk of stroke in T2DM with SGLT2-Is vs. placebo (follow-up: range 12-104 weeks)**											
11983 (23 RCTs)	serious[a]	not serious	not serious	very serious[b]	none	⊕◯◯◯ VERY LOW	19/3466 (0.5%)	29/8517 (0.3%)	RR 0.63 (0.38 to 1.03)	1 per 100	0 fewer per 100 (from 0 fewer to 0 fewer)
**Risk of stroke in T2DM with SGLT2-Is vs. AHGs (follow-up: range 12-156 weeks)**											
12067 (14 RCTs)	serious[a]	not serious	not serious	very serious[c]	none	⊕◯◯◯ VERY LOW	25/3866 (0.6%)	48/8201 (0.6%)	RR 1.01 (0.64 to 1.59)	1 per 100	0 fewer per 100 (from 0 fewer to 0 fewer)
**Risk of stroke in T2DM and CVD with SGLT2-Is vs. placebo (follow-up: range 52-318 weeks)**											
20737 (5 RCTs)	not serious	not serious	not serious	serious[d]	none	⊕⊕⊕◯ MODERATE	240/8192 (2.9%)	356/12545 (2.8%)	RR 0.93 (0.79 to 1.09)	3 per 100	0 fewer per 100 (from 0 fewer to 0 fewer)
**Risk of stroke in T2DM and CKD with SGLT2-Is vs. placebo (follow-up: range 12-219 weeks)**											
30951 (7 RCTs)	not serious	not serious	not serious	serious[e]	none	⊕⊕⊕◯ MODERATE	385/13902 (2.8%)	471/17049 (2.8%)	RR 1.01 (0.88 to 1.15)	3 per 100	0 fewer per 100 (from 0 fewer to 0 fewer)

Abbreviations: **AHGs,** anti-hyperglycaemic agents; **CVD,** cardiovascular disease; **CI,** confidence interval; **CKD,** chronic kidney disease; **RCTs,** randomised controlled trials; **RR,** risk ratio; **SGLT2-Is,** sodium-glucose cotransporter-2 inhibitors; **T2DM,** type 2 diabetes mellitus.

Note: The GRADE scores were from the fixed-effect model.

GRADE evidence

a. Five trials with small weight SGLT2-Is vs. placebo (0.2%, 0.1%, and 0.1%) and SGLT2-Is vs. SGLT2-I AHGs (ischaemic stroke [7.0% and 0.1%]) with the overall EE rated as high risk of bias due to lack of blinding (open-label design) out of 49 trials.

b. The overall imprecision was precise, with no significant effect size difference (P=0.06). However, all trials reported overlapping CIs, in which 11 trials reported wide CIs. The 95% CI was consistent with the possibility of a benefit not exceeding the MID, including only 48 events with a large sample size.

c. The overall imprecision was precise, with no significant effect size difference (P=0.96). However, all trials reported overlapping CIs, in which eight trials reported wide CIs. The 95% CI was consistent with the possibility of a benefit not exceeding the MID, including only 73 events with a large sample size.

d. The overall imprecision was precise, with no significant effect size difference (P=0.38). However, all trials reported overlapping CIs, in which one trial reported a wide CI. The 95% CI was consistent with the possibility of a benefit exceeding the MID, including only 596 events with a large sample size.

e. The overall imprecision was precise, with no significant effect size difference (P=0.93). However, all trials reported overlapping CIs, in which one trial reported a wide CI. The 95% CI was consistent with the possibility of a benefit exceeding the MID, including only 856 events with a large sample size.

In summary, the overall certainty of the pooled EE had moderate imprecision without a significant effect size different in the level of evidence (P=0.42). However, the majority of trials reported overlapping CIs, in which 21 trials reported wide CIs. In total, only one small trial (0.7%) did not cross the line of no difference (1), in which there was a significant reduction in stroke in the direction of SGLT2-Is favouring active comparators of AHGs. The 95% CI was

consistent with the possibility of a large benefit exceeding the MID, including a total of 1,573 events with a large sample size (75,738). There was no evidence of inconsistency, statistically significant heterogeneity (P=0.85; I^2=0%), or subgroup difference (P=0.32; I^2=14.6%). The majority of trials had low risk of bias (98.8%). There was no evidence of detection bias. However, the magnitude of the impact of some trials that suffered from limitations likely resulted in biased assessment of the intervention effect due to performance and selection bias (five trials with open-label design out of 49). The indirectness of the trials indicated a high level of evidence, with an individual trial PICOS element aligned closely to the review PICOS. Finally, there was no evidence of reporting bias. However, one trial reported an outlier outside the pyramid edge due to multiple interventional groups, consequently resulting in a very wide CI.

SGLT2-Is and non-fatal stroke subtypes

The incidence of stroke with SGLT2-Is in patients with T2DM did not decrease (RR, 0.95; 95% CI, 0.86−1.06; P=0.36) for ischaemic (RR, 0.96; 95% CI, 0.87−1.07; P=0.50) or haemorrhagic (RR, 0.46; 95% CI, 0.10−2.09; P=0.32) events versus placebo, or for either ischaemic (RR, 0.82; 95% CI, 0.49−1.38; P=0.46) or haemorrhagic (RR, 0.50; 95% CI, 0.08−3.02; P=0.45) events versus AHGs using random-effect model (Figure S2). The GRADE scores were moderate (Table S8).

Study or Subgroup	SGLT2-Is Events	SGLT2-Is Total	Non-SGLT2-Is Events	Non-SGLT2-Is Total	Weight	Risk Ratio M-H, Random, 95% CI
**6.1.1 Ischaemic stroke (SGLT2-Is vs. placebo)**						
Bailey 2010	1	409	2	137	0.2%	0.17 [0.02, 1.83]
Bailey 2014	1	199	0	75	0.1%	1.14 [0.05, 27.68]
Barnett 2014	5	419	3	319	0.5%	1.27 [0.31, 5.27]
Bode 2015	5	208	0	104	0.1%	5.53 [0.31, 99.00]
Bolinder 2014	1	91	0	91	0.1%	3.00 [0.12, 72.69]
Cai 2018	0	108	2	108	0.1%	0.20 [0.01, 4.12]
CANVAS, Canagliflozin (1)	146	5795	128	4347	19.8%	0.86 [0.68, 1.08]
Cefalu 2013	7	968	3	482	0.6%	1.16 [0.30, 4.47]
Cefalu 2015	2	460	2	462	0.3%	1.00 [0.14, 7.10]
CREDENCE, Canagliflozin (2)	53	2202	66	2199	8.5%	0.80 [0.56, 1.15]
Dagogo-Jack 2018	1	309	0	153	0.1%	1.49 [0.06, 36.37]
DECLARE-TIMI 58, Dapagliflozin (3)	235	8582	231	8578	33.9%	1.02 [0.85, 1.22]
EMPA-REG OUTCOME, Empagliflozin (4)	150	4687	60	2333	12.5%	1.24 [0.93, 1.67]
Ha¨ring 2013	1	359	0	141	0.1%	1.18 [0.05, 28.88]
Henry 2012 (Study1)	0	397	1	201	0.1%	0.17 [0.01, 4.13]
Ji 2014	1	261	0	132	0.1%	1.52 [0.06, 37.13]
Ji 2015	1	450	1	226	0.1%	0.50 [0.03, 7.99]
Kadowaki 2015	1	752	0	109	0.1%	0.44 [0.02, 10.69]
Kaku 2014	0	174	1	87	0.1%	0.17 [0.01, 4.07]
Kohan 2014	1	168	0	84	0.1%	1.51 [0.06, 36.65]
Kovacs 2014	0	333	2	165	0.1%	0.10 [0.00, 2.06]
Leiter 2014 NCT01042977	1	482	4	483	0.2%	0.25 [0.03, 2.23]
Merker 2015	1	430	0	207	0.1%	1.45 [0.06, 35.39]
NCT01137474	1	633	1	311	0.1%	0.49 [0.03, 7.83]
NCT01210001	1	333	0	165	0.1%	1.49 [0.06, 36.40]
NCT01649297	2	876	1	107	0.2%	0.24 [0.02, 2.67]
Roden 2013	1	534	1	229	0.1%	0.43 [0.03, 6.83]
Rosenstock 2014	1	375	1	188	0.1%	0.50 [0.03, 7.97]
Rosenstock 2015 NCT01011868	4	324	0	170	0.1%	4.74 [0.26, 87.44]
Stenlof 2012	0	392	1	192	0.1%	0.16 [0.01, 4.00]
Strojek 2011	2	450	0	146	0.1%	1.63 [0.08, 33.75]
VERTIS CV, Ertugliflozin (5)	157	5499	78	2747	15.2%	1.01 [0.77, 1.31]
Weir 2014	3	358	1	78	0.2%	0.65 [0.07, 6.20]
Wilding 2013	1	313	0	156	0.1%	1.50 [0.06, 36.61]
Wilding 2014	2	610	3	197	0.3%	0.22 [0.04, 1.28]
Yang 2018	1	139	0	133	0.1%	2.87 [0.12, 69.87]
**Subtotal (95% CI)**		**39079**		**26042**	**95.1%**	**0.96 [0.87, 1.07]**
Total events	790		593			
Heterogeneity: Tau² = 0.00; Chi² = 25.12, df = 35 (P = 0.89); I² = 0%						
Test for overall effect: Z = 0.67 (P = 0.50)						
**6.1.2 Haemorrhagic stroke (SGLT2-Is vs. placebo)**						
Dagogo-Jack 2018	2	309	0	153	0.1%	2.48 [0.12, 51.42]
Kaku 2014	0	174	1	87	0.1%	0.17 [0.01, 4.07]
Rosenstock 2015 NCT01011868	1	324	1	170	0.1%	0.52 [0.03, 8.34]
Stenlof 2012	0	392	1	192	0.1%	0.16 [0.01, 4.00]
**Subtotal (95% CI)**		**1199**		**602**	**0.5%**	**0.46 [0.10, 2.09]**
Total events	3		3			
Heterogeneity: Tau² = 0.00; Chi² = 2.00, df = 3 (P = 0.57); I² = 0%						
Test for overall effect: Z = 1.00 (P = 0.32)						
**6.1.3 Ischaemic stroke (SGLT2-Is vs. AHGs)**						
Araki 2015	3	1097	0	63	0.1%	0.41 [0.02, 7.81]
Cefalu 2013	1	968	0	482	0.1%	1.50 [0.06, 36.64]
DeFronzo 2015	4	546	1	128	0.2%	0.94 [0.11, 8.32]
Ferrannini 2013 (Study 1)	1	215	0	56	0.1%	0.79 [0.03, 19.18]
Ferrannini 2013 (Study 2)	3	332	3	56	0.4%	0.17 [0.03, 0.81]
Forst 2014	1	227	0	115	0.1%	1.53 [0.06, 37.18]
Hadjadj 2016	1	1032	1	341	0.1%	0.33 [0.02, 5.27]
Lavalle-Gonzales 2013	4	735	0	549	0.1%	6.73 [0.36, 124.66]
Miller 2018	0	194	1	97	0.1%	0.17 [0.01, 4.07]
Nauck 2014	2	406	0	408	0.1%	5.02 [0.24, 104.33]
Ridderstra°le 2014	14	765	15	780	2.1%	0.95 [0.46, 1.96]
Rosenstock 2015	1	358	0	176	0.1%	1.48 [0.06, 36.12]
Rosenstock 2016	2	949	0	237	0.1%	1.25 [0.06, 26.00]
Schernthaner 2013	1	377	1	378	0.1%	1.00 [0.06, 15.97]
**Subtotal (95% CI)**		**8201**		**3866**	**4.1%**	**0.82 [0.49, 1.38]**
Total events	38		22			
Heterogeneity: Tau² = 0.00; Chi² = 9.75, df = 13 (P = 0.71); I² = 0%						
Test for overall effect: Z = 0.74 (P = 0.46)						
**6.1.4 Haemorrhagic stroke (SGLT2-Is vs. AHGs)**						
Cefalu 2013	1	968	0	482	0.1%	1.50 [0.06, 36.64]
DeFronzo 2015	1	546	0	128	0.1%	0.71 [0.03, 17.27]
Ridderstra°le 2014	0	765	3	780	0.1%	0.15 [0.01, 2.82]
**Subtotal (95% CI)**		**2279**		**1390**	**0.3%**	**0.50 [0.08, 3.02]**
Total events	2		3			
Heterogeneity: Tau² = 0.00; Chi² = 1.20, df = 2 (P = 0.55); I² = 0%						
Test for overall effect: Z = 0.75 (P = 0.45)						
**Total (95% CI)**		**50758**		**31900**	**100.0%**	**0.95 [0.86, 1.06]**
Total events	833		621			
Heterogeneity: Tau² = 0.00; Chi² = 39.51, df = 56 (P = 0.95); I² = 0%						
Test for overall effect: Z = 0.91 (P = 0.36)						
Test for subgroup differences: Chi² = 1.73, df = 3 (P = 0.63), I² = 0%						

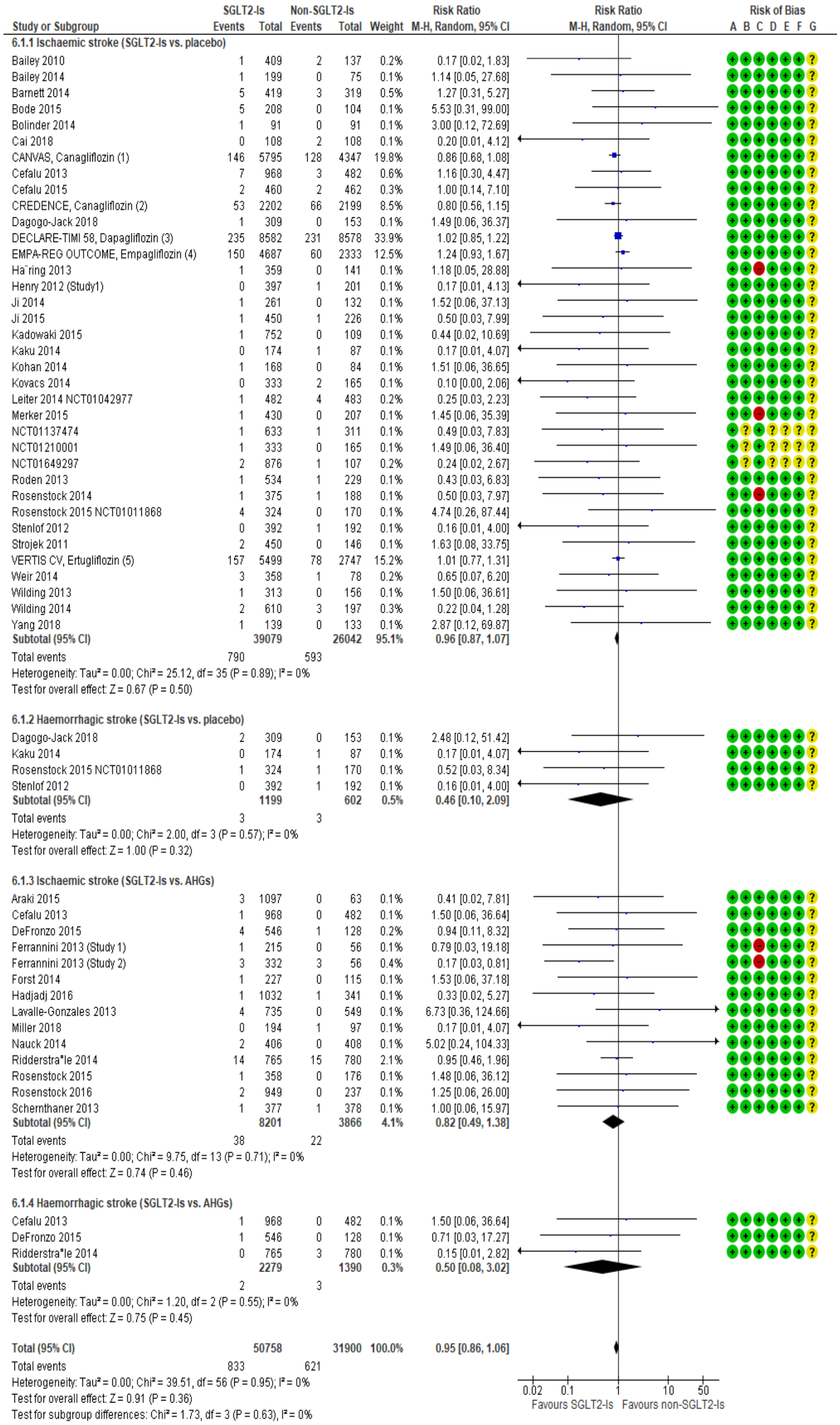

Footnotes
(1) The Canagliflozin Cardiovascular Assessment Study
(2) Canagliflozin and Renal Events in Diabetes with Established Nephropathy Clinical Evaluaation
(3) Multicentre Trial to Evaluate the Effect of Dapagliflozin on the Incidence of Cardiovascular Events
(4) Empagliflozin Cardiovascular Outcome Event in Type 2 Diabetes Mellitus Patients
(5) Evaluation of Ertugliflozin Efficacy and Safety Cardiovascular Outcomes

Risk of bias legend
(A) Random sequence generation (selection bias)
(B) Allocation concealment (selection bias)
(C) Blinding of participants and personnel (performance bias)
(D) Blinding of outcome assessment (detection bias)
(E) Incomplete outcome data (attrition bias)
(F) Selective reporting (reporting bias)
(G) Other bias

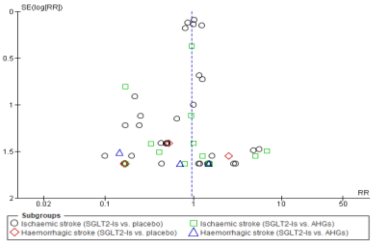

Figure S2. Forest and funnel plot of SGLTS-Is and non-fatal stroke by subtypes, Random-effect model.

Table S8. GRADE evidence profile of SGLT2-Is and non-fatal stroke subtypes

Certainty assessment							Summary of findings				
							Study event rates (%)		Relative effect (95% CI)	Anticipated absolute effects	
№ of participants (trials)	Risk of bias	Inconsistency	Indirectness	Imprecision	Publication bias	Overall certainty of evidence	With placebo / controls	With SGLT2-Is		Risk with placebo / controls	Risk difference with SGLT2-Is
**SGLT2-Is and non-fatal stroke by subtypes (follow-up: range 12-318 weeks)**											
82658 (49 RCTs)	serious[a]	not serious	not serious	not serious	none	⨁⨁⨁◯ MODERATE	621/31900 (1.9%)	833/50758 (1.6%)	RR 0.96 (0.86 to 1.06)	2 per 100	0 fewer per 100 (from 0 fewer to 0 fewer)
**Ischaemic stroke with SGLT2-Is vs. placebo (follow-up: range 12-318 weeks)**											
65121 (36 RCTs)	serious[a]	not serious	not serious	serious[b]	none	⨁⨁◯◯ LOW	593/26042 (2.3%)	790/39079 (2.0%)	RR 0.97 (0.87 to 1.07)	2 per 100	0 fewer per 100 (from 0 fewer to 0 fewer)
**Haemorrhagic stroke with SGLT2-Is vs. placebo (follow-up: range 24-318 weeks)**											
1801 (4 RCTs)	not serious	not serious	not serious	very serious[c]	none	⨁⨁◯◯ LOW	3/602 (0.5%)	3/1199 (0.3%)	RR 0.50 (0.14 to 1.85)	0 per 100	0 fewer per 100 (from 0 fewer to 0 fewer)
**Ischaemic stroke with SGLT2-Is vs. AHGs (follow-up: range 12-156 weeks)**											
12067 (14 RCTs)	serious[a]	not serious	not serious	very serious[d]	none	⨁◯◯◯ VERY LOW	22/3866 (0.6%)	38/8201 (0.5%)	RR 0.94 (0.58 to 1.52)	1 per 100	0 fewer per 100 (from 0 fewer to 0 fewer)
**Haemorrhagic stroke with SGLT2-Is vs. AHGs (follow-up: range 52-104 weeks)**											
3669 (3 RCTs)	not serious	not serious	not serious	very serious[e]	none	⨁⨁◯◯ LOW	3/1390 (0.2%)	2/2279 (0.1%)	RR 0.42 (0.08 to 2.12)	0 per 100	0 fewer per 100 (from 0 fewer to 0 fewer)

Abbreviations: **AHGs,** anti-hyperglycaemic agents; **CI,** confidence interval; **RCTs,** randomised controlled trials; **RR,** risk ratio; **SGLT2-Is,** sodium-glucose cotransporter-2 inhibitors.

Note: The GRADE scores were from the fixed-effect model.

GRADE evidence

a. Five trials with small weight SGLT2-Is vs. placebo (ischaemic stroke [0.2%, 0.1%, and 0.1%]) and SGLT2-Is vs. AHGs (ischaemic stroke [0.7% and 0.1%]) with the overall EE rated as high risk of bias due to lack of blinding (open-label design) out of 49 trials.

b. The overall imprecision was precise, with no significant effect size difference (P=0.53). However, all trials reported overlapping CIs, in which 13 trials reported wide CIs. The 95% CI was consistent with the possibility of a benefit exceeding the MID, including only 1,383 events with a large sample size.

c. The overall imprecision was precise, with no significant effect size difference (P=0.30). However, all trials reported overlapping CIs, in which one trial reported a wide CI. The 95% CI was consistent with the possibility of a benefit not exceeding the MID, including only six events with a small sample size (1,801).

d. The overall imprecision was precise, with no significant effect size difference (P=0.79). However, all trials reported overlapping CIs, in which eight trials reported wide CIs. The 95% CI was consistent with the possibility of a benefit not exceeding the MID, including only 60 events with a large sample size.

e. The overall imprecision was precise, with no significant effect size difference (P=0.29). However, all trials reported overlapping CIs, in which two trials reported wide CIs. The 95% CI was consistent with the possibility of a benefit not exceeding the MID, including only five events with a large sample size.

In summary, the overall certainty of the pooled EE had moderate imprecision without a significant effect size different in the level of evidence (P=0.41). However, the majority of trials reported overlapping CIs, in which 24 trials reported wide CIs. In total, only one small trial (0.7%) did not cross the line of no difference (1), in which there was a significant reduction in ischaemic stroke in the direction of SGLT2-Is favouring active comparators of AHGs. The

95% CI was consistent with the possibility of a large benefit exceeding the MID, including 1,454 events in total with a large sample size (82,658). There was no evidence of inconsistency, statistically significant heterogeneity (P=0.95; I^2=0%), or subgroup difference (P=0.58; I^2=0%). The majority of trials had low risk of bias (98.8%). There was no evidence of detection bias. However, the magnitude of the impact of some trials that suffer from limitations likely resulted in biased assessment of the intervention effect due to performance and selection bias (five trials with open-label design out of 49). The indirectness of the trials indicated a high level of evidence, with an individual trial PICOS element aligned closely to the review PICOS. Finally, there was no evidence of reporting bias. However, one trial reported an outlier outside the pyramid edge due to multiple interventional groups, consequently resulting in a very wide CI.

Fatal stroke risk

SGLT2-Is and fatal stroke risk

In six trials (31,327 participants with 124 stroke events), use of SGLT2-Is did not decrease fatal stroke risk (RR, 0.89; 95% CI, 0.62−1.28; P=0.54) versus placebo using fixed- and random-effect models (Figure S3 and Figure S4). The GRADE scores were moderate (Table S9).

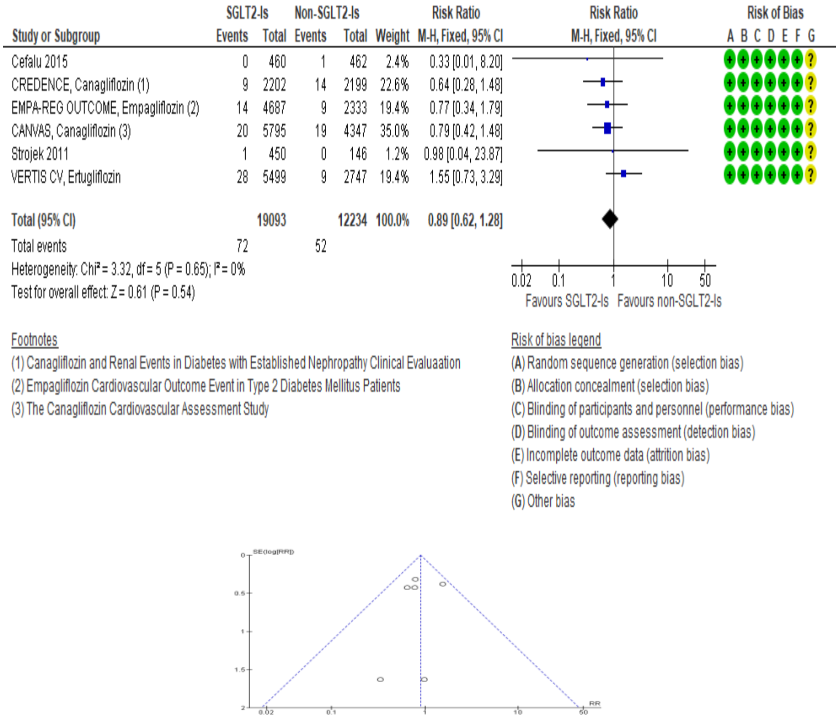

Figure S3. Forest and funnel plot of SGLT2-Is and fatal stroke, Fixed-effect model.

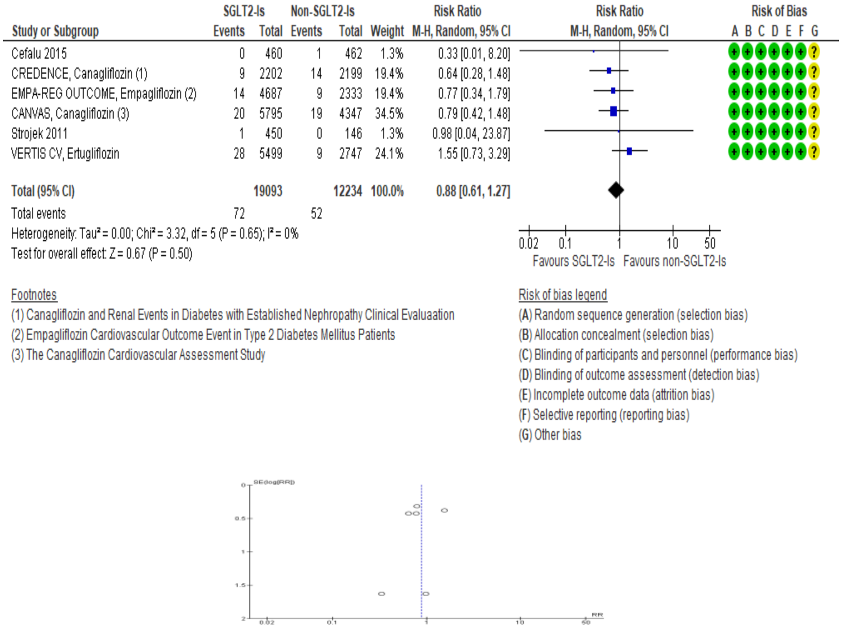

Figure S4. Forest and funnel plot of SGLT2-Is and fatal stroke, Random-effect model.

Table S9. GRADE evidence profile of SGLT2-Is and fatal stroke risk

Certainty assessment							Summary of findings				
№ of participants (trials)	Risk of bias	Inconsistency	Indirectness	Imprecision	Publication bias	Overall certainty of evidence	Study event rates (%)		Relative effect (95% CI)	Anticipated absolute effects	
							With placebo	With SGLT2-Is		Risk with placebo	Risk difference with SGLT2-Is
**SGLT2-Is and fatal stroke vs. placebo (follow-up: range 24-219 weeks)**											
31327 (6 RCTs)	not serious	not serious	not serious	serious[a]	none	⊕⊕⊕◯ MODERATE	52/12234 (0.4%)	72/19093 (0.4%)	RR 0.89 (0.62 to 1.28)	0 per 100	0 fewer per 100 (from 0 fewer to 0 fewer)

Abbreviations: **AHGs,** anti-hyperglycaemic agents; **CI,** confidence interval; **RCTs,** randomised controlled trials; **RR,** risk ratio; **SGLT2-Is,** sodium-glucose cotransporter-2 inhibitors.

Note: The GRADE scores were from the fixed-effect model.

GRADE evidence

a. The overall imprecision was precise, with no significant effect size difference (P=0.54). However, all trials reported overlapping CIs, in which one trial reported a wide CI. The 95% CI was consistent with the possibility of a benefit not exceeding the MID, including only 124 events with a large sample size (31,327).

In summary, the overall certainty of the pooled EE had moderate imprecision without a significant effect size difference in the level of evidence (P=0.54). However, the majority of trials reported overlapping CIs, in which one trial reported a wide CI. All trials crossed the line of no difference (1). The 95% CI was consistent with the possibility of a benefit not exceeding the MID, including a total of 124 events with a large sample size. There was no evidence of inconsistency or statistically significant heterogeneity (P=0.65; I^2=0%). All trials had low risk of bias (100%). There was no evidence of detection or selection bias. The indirectness of the trials indicated a high level of evidence, with an individual trial PICOS element aligned closely to the review PICOS. Finally, there was no evidence of reporting bias; all included trials were within the two boundaries of the pyramid.

GLP1-RAs and stroke by baseline characteristic

Use of GLP1-RAs decreased stroke risk in patients with T2DM at risk of CVD (RR, 0.86; 95% CI, 0.76−0.96; P=0.01) or in patients with T2DM at risk of CVD and/or CKD (RR, 0.82; 95% CI, 0.69−0.98; P=0.03) versus placebo. However, use of GLP1-RAs did not decrease stroke risk in patients with T2DM (RR, 0.72; 95% CI, 0.36−1.42; P=0.34) versus placebo or AHGs (RR, 0.85; 95% CI, 0.48−1.49; P=0.56) using fixed- and random-effect models (Figure S5 and Figure S6). The GRADE scores were moderate (Table S10).

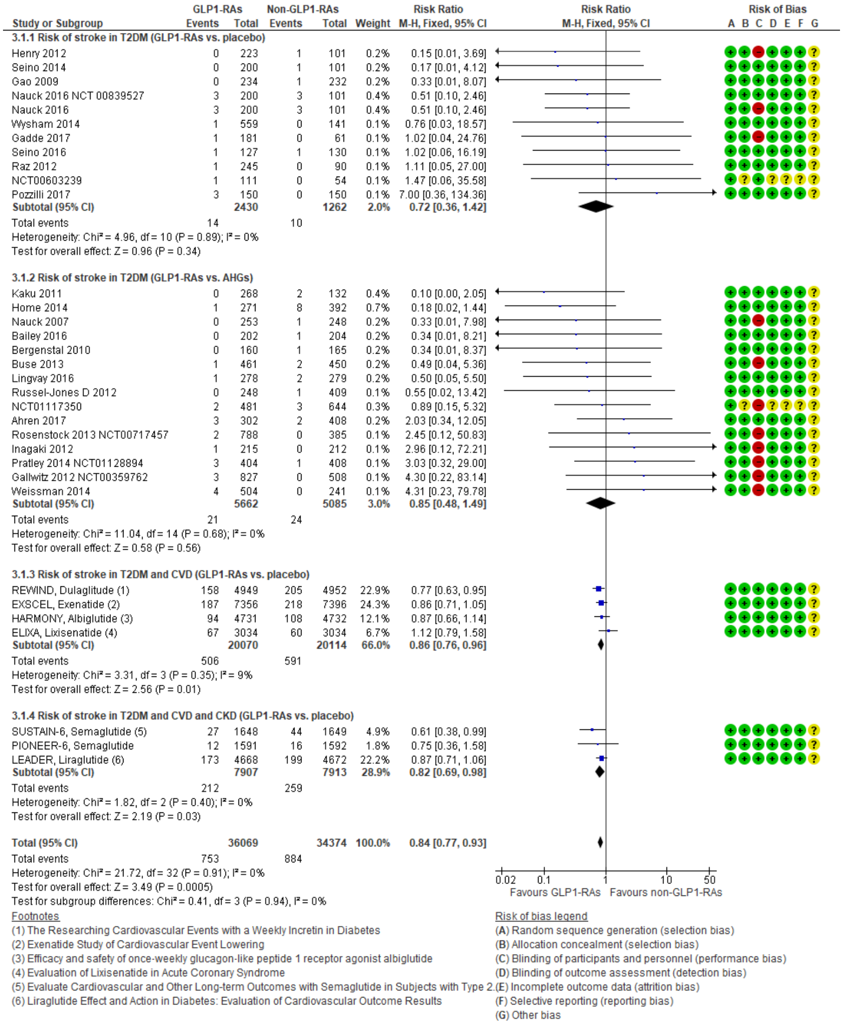

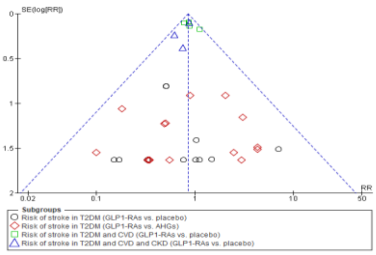

Figure S5. Forest and funnel plot of GLP1-RAs and stroke by baseline characteristic, Fixed-effect model.

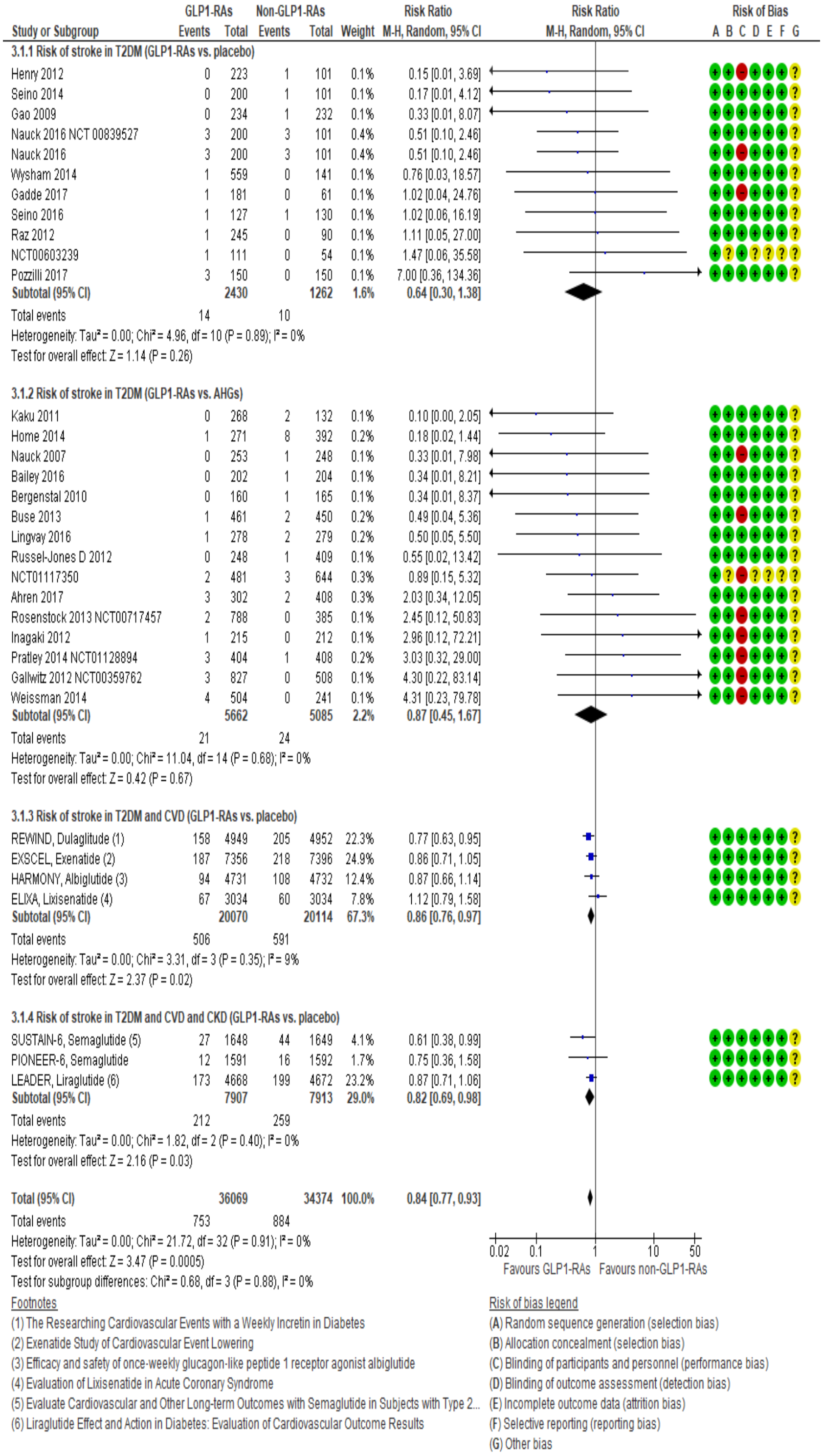

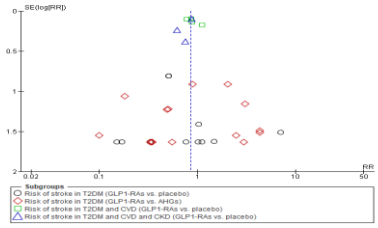

Figure S6. Forest and funnel plot of GLP1-RAs and stroke by baseline characteristic, Random-effect model.

Table S10. GRADE evidence profile of GLP1-RAs by baseline characteristic

Certainty assessment							Summary of findings				
№ of participants (trials)	Risk of bias	Inconsistency	Indirectness	Imprecision	Publication bias	Overall certainty of evidence	Study event rates (%)		Relative effect (95% CI)	Anticipated absolute effects	
							With placebo / controls	With GLP1-RAs		Risk with placebo / controls	Risk difference with GLP1-RAs
**Risk of stroke in T2DM by baseline characteristic (follow-up: range 16-339 weeks)**											
70443 (33 RCTs)	serious[a]	not serious	not serious	not serious	none	⊕⊕⊕◯ MODERATE	884/34374 (2.6%)	753/36069 (2.1%)	RR 0.84 (0.77 to 0.93)	3 per 100	0 fewer per 100 (from 1 fewer to 0 fewer)
**Risk of stroke in T2DM with GLP1-RAs vs. placebo (follow-up: range 16-156 weeks)**											
3692 (11 RCTs)	serious[a]	not serious	not serious	very serious[b]	none	⊕◯◯◯ VERY LOW	10/1262 (0.8%)	14/2430 (0.6%)	RR 0.72 (0.36 to 1.42)	1 per 100	0 fewer per 100 (from 1 fewer to 0 fewer)
**Risk of stroke in T2DM with GLP1-RAs vs. AHGs (follow-up: range 26-235 weeks)**											
10747 (15 RCTs)	serious[a]	not serious	not serious	very serious[c]	none	⊕◯◯◯ VERY LOW	24/5085 (0.5%)	21/5662 (0.4%)	RR 0.85 (0.48 to 1.49)	0 per 100	0 fewer per 100 (from 0 fewer to 0 fewer)
**Risk of stroke in T2DM and CVD with GLP1-RAs vs. placebo (follow-up: range 84-339 weeks)**											
40184 (4 RCTs)	not serious	not serious	not serious	not serious	none	⊕⊕⊕⊕ HIGH	591/20114 (2.9%)	506/20070 (2.5%)	RR 0.86 (0.76 to 0.96)	3 per 100	0 fewer per 100 (from 1 fewer to 0 fewer)
**Risk of stroke in T2DM with CVD and CKD with GLP1-RAs vs. placebo (follow-up: range 104-198 weeks)**											
15820 (3 RCTs)	not serious	not serious	not serious	not serious	none	⊕⊕⊕⊕ HIGH	259/7913 (3.3%)	212/7907 (2.7%)	RR 0.82 (0.69 to 0.98)	3 per 100	1 fewer per 100 (from 1 fewer to 0 fewer)

Abbreviations: **AHG,** anti-hyperglycaemic agent; **CVD,** cardiovascular disease; **CI,** confidence interval; **CKD,** chronic kidney disease; **GLP1-RAs,** glucagon-like peptide-1 receptor agonists; **RCTs,** randomised controlled trials; **RR,** risk ratio; **T2DM,** type 2 diabetes mellitus.

Note: The GRADE scores were from the fixed-effect model.

GRADE evidence

a. Eleven trials with small weight GLP1-RAs vs. placebo (in T2DM [0.4%, 0.2%, and 0.1%]) and GLP1-RAs vs. AHGs (in T2DM [0.3%, 0.2%, 0.2%, 0.1%, 0.1%, 0.1%, 0.1%, and 0.1%]) with the overall EE rated as high risk of bias due to lack of blinding (open-label design) out of 33 trials, one of which was an unpublished RCT.

b. The overall imprecision was precise, with no significant effect size difference (P=0.34). However, all trials reported overlapping CIs, in which six trials reported wide CIs. The 95% CI was consistent with the possibility of a benefit not exceeding the MID, including only 24 events with a large sample size.

c. The overall imprecision was precise, with no significant effect size difference (P=0.56). However, all trials reported overlapping CIs, in which seven trials reported wide CIs. The 95% CI was consistent with the possibility of a benefit not exceeding the MID, including only 45 events with a large sample size.

In summary, the overall certainty of the pooled EE had moderate imprecision with a significant effect size different in the level of evidence (P=0.0005). However, the majority of trials reported overlapping CIs, in which 13 trials reported wide CIs. In total, only one large (22.9%) and small (4.9%) trials did not cross the line of no difference (1), in which a significant reduction of stroke in the direction of GLP1-RAs favoured placebo in patients with T2DM and with CVD and CKD. The 95% CI was consistent with the possibility of a large benefit exceeding the MID, including a total of 1,637 events with a large sample size (70,443). There was no evidence of inconsistency, statistically significant heterogeneity (P=0.91; I^2=0%), or subgroup difference (P=0.94; I^2=0%). The majority of trials had low risk of bias (98.1%). There was no evidence of detection bias. However, the magnitude of the impact of some trials that suffered from limitations likely resulted in biased assessment of the intervention effect due to performance and selection bias (13 trials with open-label design out of 33, one of which was

an unpublished RCT). The indirectness of the trials indicated a high level of evidence, with an individual trial PICOS element aligned closely to the review PICOS. Finally, there was no evidence of reporting bias; all included trials were within the two boundaries of the pyramid.

GLP1-RAs and non-fatal stroke by subtypes

The incidence of stroke with GLP1-RAs in patients with T2DM was significantly decreased (RR, 0.85; 95% CI, 0.77−0.94; P=0.002) for ischaemic (RR, 0.85; 95% CI, 0.77−0.94; P=0.002) events versus placebo. However, the incidence of haemorrhagic stroke did not decrease (RR, 0.50; 95% CI, 0.09−2.73; P=0.42) versus placebo, or for either ischaemic (RR, 0.90; 95% CI, 0.44−1.81; P=0.76) or haemorrhagic (RR, 1.71; 95% CI, 0.35−8.47; P=0.51) events versus AHGs using random-effect model (Figure S7). The GRADE scores were moderate (Table S11).

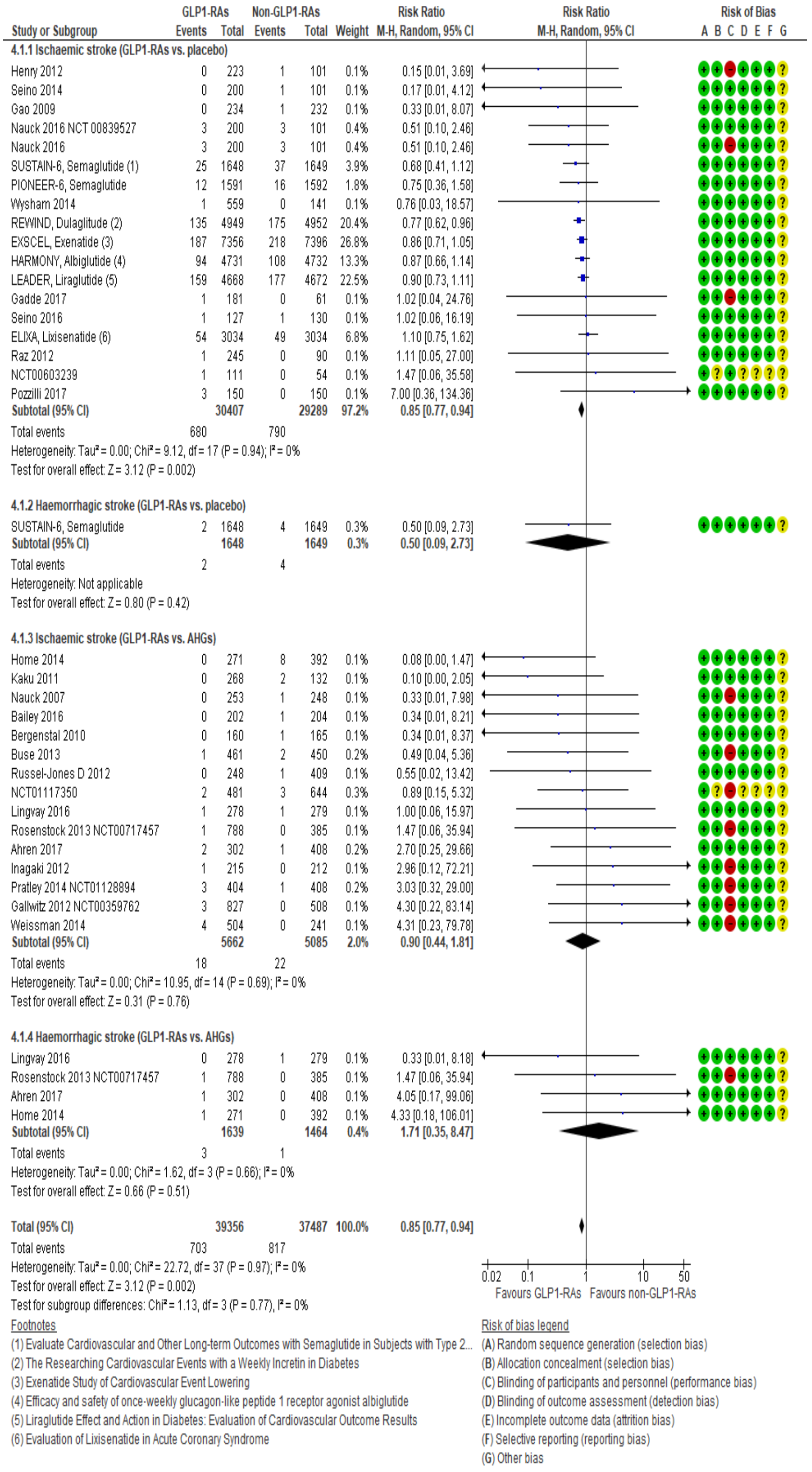

Study or Subgroup	GLP1-RAs Events	GLP1-RAs Total	Non-GLP1-RAs Events	Non-GLP1-RAs Total	Weight	Risk Ratio M-H, Random, 95% CI
**4.1.1 Ischaemic stroke (GLP1-RAs vs. placebo)**						
Henry 2012	0	223	1	101	0.1%	0.15 [0.01, 3.69]
Seino 2014	0	200	1	101	0.1%	0.17 [0.01, 4.12]
Gao 2009	0	234	1	232	0.1%	0.33 [0.01, 8.07]
Nauck 2016 NCT 00839527	3	200	3	101	0.4%	0.51 [0.10, 2.46]
Nauck 2016	3	200	3	101	0.4%	0.51 [0.10, 2.46]
SUSTAIN-6, Semaglutide (1)	25	1648	37	1649	3.9%	0.68 [0.41, 1.12]
PIONEER-6, Semaglutide	12	1591	16	1592	1.8%	0.75 [0.36, 1.58]
Wysham 2014	1	559	0	141	0.1%	0.76 [0.03, 18.57]
REWIND, Dulaglitude (2)	135	4949	175	4952	20.4%	0.77 [0.62, 0.96]
EXSCEL, Exenatide (3)	187	7356	218	7396	26.8%	0.86 [0.71, 1.05]
HARMONY, Albiglutide (4)	94	4731	108	4732	13.3%	0.87 [0.66, 1.14]
LEADER, Liraglutide (5)	159	4668	177	4672	22.5%	0.90 [0.73, 1.11]
Gadde 2017	1	181	0	61	0.1%	1.02 [0.04, 24.76]
Seino 2016	1	127	1	130	0.1%	1.02 [0.06, 16.19]
ELIXA, Lixisenatide (6)	54	3034	49	3034	6.8%	1.10 [0.75, 1.62]
Raz 2012	1	245	0	90	0.1%	1.11 [0.05, 27.00]
NCT00603239	1	111	0	54	0.1%	1.47 [0.06, 35.58]
Pozzilli 2017	3	150	0	150	0.1%	7.00 [0.36, 134.36]
**Subtotal (95% CI)**		**30407**		**29289**	**97.2%**	**0.85 [0.77, 0.94]**
Total events	680		790			
Heterogeneity: Tau² = 0.00; Chi² = 9.12, df = 17 (P = 0.94); I² = 0%						
Test for overall effect: Z = 3.12 (P = 0.002)						
**4.1.2 Haemorrhagic stroke (GLP1-RAs vs. placebo)**						
SUSTAIN-6, Semaglutide	2	1648	4	1649	0.3%	0.50 [0.09, 2.73]
**Subtotal (95% CI)**		**1648**		**1649**	**0.3%**	**0.50 [0.09, 2.73]**
Total events	2		4			
Heterogeneity: Not applicable						
Test for overall effect: Z = 0.80 (P = 0.42)						
**4.1.3 Ischaemic stroke (GLP1-RAs vs. AHGs)**						
Home 2014	0	271	8	392	0.1%	0.08 [0.00, 1.47]
Kaku 2011	0	268	2	132	0.1%	0.10 [0.00, 2.05]
Nauck 2007	0	253	1	248	0.1%	0.33 [0.01, 7.98]
Bailey 2016	0	202	1	204	0.1%	0.34 [0.01, 8.21]
Bergenstal 2010	0	160	1	165	0.1%	0.34 [0.01, 8.37]
Buse 2013	1	461	2	450	0.2%	0.49 [0.04, 5.36]
Russel-Jones D 2012	0	248	1	409	0.1%	0.55 [0.02, 13.42]
NCT01117350	2	481	3	644	0.3%	0.89 [0.15, 5.32]
Lingvay 2016	1	278	1	279	0.1%	1.00 [0.06, 15.97]
Rosenstock 2013 NCT00717457	1	788	0	385	0.1%	1.47 [0.06, 35.94]
Ahren 2017	2	302	1	408	0.2%	2.70 [0.25, 29.66]
Inagaki 2012	1	215	0	212	0.1%	2.96 [0.12, 72.21]
Pratley 2014 NCT01128894	3	404	1	408	0.2%	3.03 [0.32, 29.00]
Gallwitz 2012 NCT00359762	3	827	0	508	0.1%	4.30 [0.22, 83.14]
Weissman 2014	4	504	0	241	0.1%	4.31 [0.23, 79.78]
**Subtotal (95% CI)**		**5662**		**5085**	**2.0%**	**0.90 [0.44, 1.81]**
Total events	18		22			
Heterogeneity: Tau² = 0.00; Chi² = 10.95, df = 14 (P = 0.69); I² = 0%						
Test for overall effect: Z = 0.31 (P = 0.76)						
**4.1.4 Haemorrhagic stroke (GLP1-RAs vs. AHGs)**						
Lingvay 2016	0	278	1	279	0.1%	0.33 [0.01, 8.18]
Rosenstock 2013 NCT00717457	1	788	0	385	0.1%	1.47 [0.06, 35.94]
Ahren 2017	1	302	0	408	0.1%	4.05 [0.17, 99.06]
Home 2014	1	271	0	392	0.1%	4.33 [0.18, 106.01]
**Subtotal (95% CI)**		**1639**		**1464**	**0.4%**	**1.71 [0.35, 8.47]**
Total events	3		1			
Heterogeneity: Tau² = 0.00; Chi² = 1.62, df = 3 (P = 0.66); I² = 0%						
Test for overall effect: Z = 0.66 (P = 0.51)						
**Total (95% CI)**		**39356**		**37487**	**100.0%**	**0.85 [0.77, 0.94]**
Total events	703		817			
Heterogeneity: Tau² = 0.00; Chi² = 22.72, df = 37 (P = 0.97); I² = 0%						
Test for overall effect: Z = 3.12 (P = 0.002)						
Test for subgroup differences: Chi² = 1.13, df = 3 (P = 0.77), I² = 0%						

Footnotes
(1) Evaluate Cardiovascular and Other Long-term Outcomes with Semaglutide in Subjects with Type 2...
(2) The Researching Cardiovascular Events with a Weekly Incretin in Diabetes
(3) Exenatide Study of Cardiovascular Event Lowering
(4) Efficacy and safety of once-weekly glucagon-like peptide 1 receptor agonist albiglutide
(5) Liraglutide Effect and Action in Diabetes: Evaluation of Cardiovascular Outcome Results
(6) Evaluation of Lixisenatide in Acute Coronary Syndrome

Risk of bias legend
(A) Random sequence generation (selection bias)
(B) Allocation concealment (selection bias)
(C) Blinding of participants and personnel (performance bias)
(D) Blinding of outcome assessment (detection bias)
(E) Incomplete outcome data (attrition bias)
(F) Selective reporting (reporting bias)
(G) Other bias

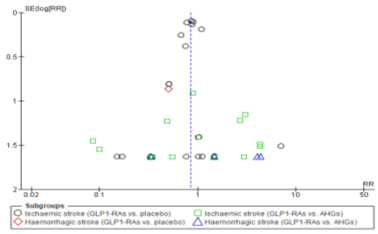

Figure S7. Forest and funnel plot of GLP1-RAs and non-fatal stroke by subtypes, Random-effect model.

Table S11. GRADE evidence profile of GLP1-RAs and non-fatal stroke by subtypes

Certainty assessment							Summary of findings				
№ of participants (trials)	Risk of bias	Inconsistency	Indirectness	Imprecision	Publication bias	Overall certainty of evidence	Study event rates (%)		Relative effect (95% CI)	Anticipated absolute effects	
							With placebo / controls	With GLP1-RAs		Risk with placebo / controls	Risk difference with GLP1-RAs
**GLP1-RAs non-fatal stroke by subtypes (follow-up: range 16-339 weeks)**											
76843 (33 RCTs)	serious[a]	not serious	not serious	not serious	none	⊕⊕⊕◯ MODERATE	817/37487 (2.2%)	703/39356 (1.8%)	RR 0.85 (0.77 to 0.94)	2 per 100	0 fewer per 100 (from 1 fewer to 0 fewer)
**Ischaemic stroke with GLP1-RAs vs. placebo (follow-up: range 16-339 weeks)**											
59696 (18 RCTs)	serious[a]	not serious	not serious	serious[b]	none	⊕⊕◯◯ LOW	790/29289 (2.7%)	680/30407 (2.2%)	RR 0.85 (0.77 to 0.94)	3 per 100	0 fewer per 100 (from 1 fewer to 0 fewer)
**Haemorrhagic stroke with GLP1-RAs vs. placebo (follow-up: mean 104 weeks)**											
3297 (1 RCT)	not serious	not serious	not serious	very serious[c]	none	⊕⊕◯◯ LOW	4/1649 (0.2%)	2/1648 (0.1%)	RR 0.50 (0.09 to 2.73)	0 per 100	0 fewer per 100 (from 0 fewer to 0 fewer)
**Ischaemic stroke with GLP1-RAs vs. AHGs (follow-up: range 26-235 weeks)**											
10747 (15 RCTs)	serious[a]	not serious	not serious	very serious[d]	none	⊕◯◯◯ VERY LOW	22/5085 (0.4%)	18/5662 (0.3%)	RR 0.80 (0.45 to 1.44)	0 per 100	0 fewer per 100 (from 0 fewer to 0 fewer)
**Haemorrhagic stroke with GLP1-RAs vs. AHGs (follow-up: range 27-156 weeks)**											
3103 (4 RCTs)	serious[a]	not serious	not serious	very serious[e]	none	⊕◯◯◯ VERY LOW	1/1464 (0.1%)	3/1639 (0.2%)	RR 1.66 (0.41 to 6.73)	0 per 100	0 fewer per 100 (from 0 fewer to 0 fewer)

Abbreviations: **AHGs,** anti-hyperglycaemic agents; **CI,** confidence interval; **GLP1-RAs,** glucagon-like peptide-1 receptor agonist; **RCTs,** randomised controlled trials; **RR,** risk ratio.

Note: The GRADE scores were from the fixed-effect model.

GRADE evidence

a. Twelve trials with small weight GLP1-RAs vs. placebo (0.5%, 0.2%, and 0.1%) and GLP1-RAs vs. AHGs (ischaemic stroke [0.3%, 0.2%, 0.2%, 0.1%, 0.1%, 0.1%, 0.1%, 0.1%, and 0.1%]) with the overall EE rated as high risk of bias due to lack of blinding (open-label design) out of 33 trials, one of which was an unpublished RCT.

b. The overall imprecision was precise, with a significant effect size difference (P=0.002). However, all trials reported overlapping CIs, in which six trials reported wide CIs. The 95% CI was consistent with the possibility of a large benefit exceeding the MID, including 1,470 events with a large sample size.

c. The overall imprecision was precise, with no significant effect size difference (P=0.42). However, all trials reported overlapping and narrow CIs. The 95% CI was consistent with the possibility of a benefit not exceeding the MID, including only six events with a large sample size.

d. The overall imprecision was precise, with no significant effect size difference (P=0.46). However, all trials reported overlapping CIs, in which eight trials reported wide CIs. The 95% CI was consistent with the possibility of a benefit not exceeding the MID, including only 40 events with a large sample size.

e. The overall imprecision was precise, with no significant effect size difference (P=0.48). However, all trials reported overlapping CIs, in which three trials reported wide CIs. The 95% CI was consistent with the possibility of a small benefit not exceeding the MID, including only four events with a large sample size.

In summary, the overall certainty of the pooled EE had moderate imprecision with a significant effect size difference in the level of evidence (P=0.002). However, the majority of trials reported overlapping CIs, in which 17 trials reported wide CIs. In total, only one large trial (21.1%) did not cross the line of no difference (1), in which there was a significant reduction

in non-fatal stroke in the direction of GLP1-RAs favouring placebo. The 95% CI was consistent with the possibility of a large benefit exceeding the MID, including a total of 1,520 events with a large sample size. There was no evidence of inconsistency, statistically significant heterogeneity (P=0.97; I^2=0%), or subgroup difference (P=0.73; I^2=0%). The majority of trials had low risk of bias (97.9%). There was no evidence of detection bias. However, the magnitude of the impact of some trials that suffered from limitations likely resulted in biased assessment of the intervention effect due to performance and selection bias (12 trials with open-label design out of 33, one of which was an unpublished RCT). The indirectness of the trials indicated a high level of evidence, with an individual trial PICOS element aligned closely to the review PICOS. Finally, there was no evidence of reporting bias; all included trials were within the two boundaries of the pyramid.

Fatal stroke risk

GLP1-RAs and fatal stroke risk

In seven trials (46,097 participants; 181 strokes), use of GLP1-RAs did not decrease risk of fatal ischaemic or haemorrhagic stroke (RR, 0.77; 95% CI, 0.58−1.03; P=0.08) versus non-GLP1-RAs using fixed- and random-effect models (Figure S8 and Figure S9). The GRADE scores were low (Table S12).

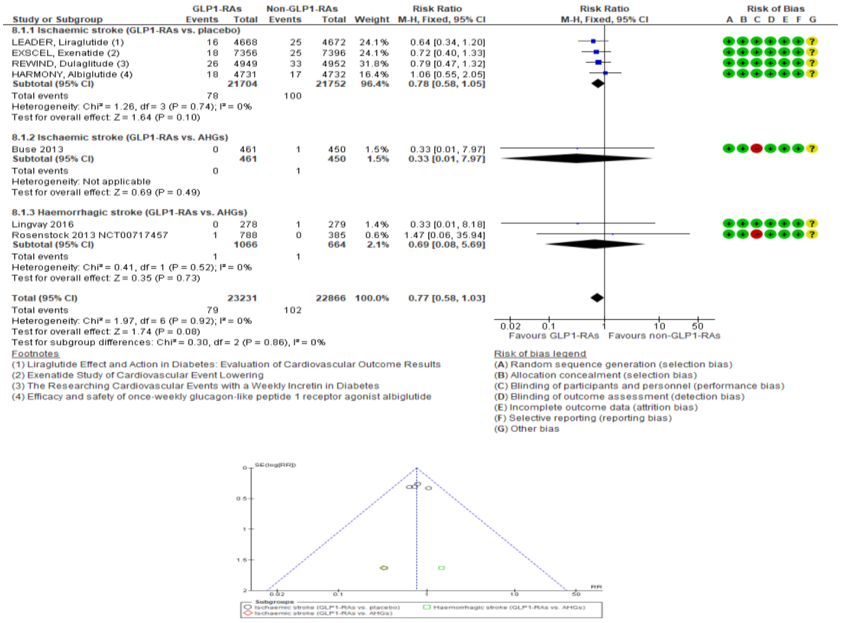

Figure S8. Forest and funnel plot of GLP1-RAs and fatal stroke, Fixed-effect model.

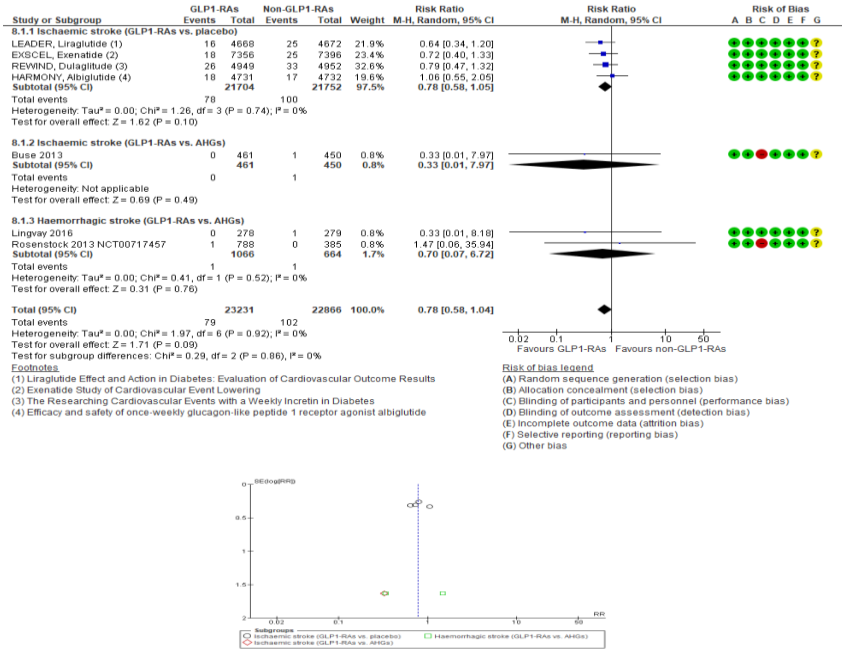

Figure S9. Forest and funnel plot of GLP1-RAs and fatal stroke, Random-effect model.

Table S12. GRADE evidence profile of GLP1-RAs and fatal stroke risk

Certainty assessment							Summary of findings				
№ of participants (trials)	Risk of bias	Inconsistency	Indirectness	Imprecision	Publication bias	Overall certainty of evidence	Study event rates (%)		Relative effect (95% CI)	Anticipated absolute effects	
							With placebo / controls	With GLP1-RAs		Risk with placebo / controls	Risk difference with GLP1-RAs
**GLP1-RAs fatal stroke (follow-up: range 16-339 weeks)**											
46097 (7 RCTs)	serious[a]	not serious	not serious	serious[b]	none	⊕⊕◯◯ LOW	102/22866 (0.4%)	79/23231 (0.3%)	RR 0.77 (0.58 to 1.03)	0 per 100	0 fewer per 100 (from 0 fewer to 0 fewer)
**Ischaemic fatal stroke GLP1-RAs vs. placebo (follow-up: range 84-339 weeks)**											
43456 (4 RCTs)	not serious	not serious	not serious	very serious[c]	none	⊕⊕◯◯ LOW	100/21752 (0.5%)	78/21704 (0.4%)	RR 0.78 (0.58 to 1.05)	0 per 100	0 fewer per 100 (from 0 fewer to 0 fewer)
**Ischaemic fatal stroke GLP1-RAs vs. AHGs (follow-up: mean 26 weeks)**											
911 (1 RCT)	serious[a]	not serious	not serious	very serious[d]	none	⊕◯◯◯ VERY LOW	1/450 (0.2%)	0/461 (0.0%)	RR 0.33 (0.01 to 7.97)	0 per 100	0 fewer per 100 (from 0 fewer to 2 more)
**Haemorrhagic fatal stroke GLP1-RAs vs. AHGs (follow-up: range 26-104 weeks)**											
1730 (2 RCTs)	serious[a]	not serious	not serious	very serious[e]	none	⊕◯◯◯ VERY LOW	1/664 (0.2%)	1/1066 (0.1%)	RR 0.69 (0.08 to 5.69)	0 per 100	0 fewer per 100 (from 0 fewer to 1 more)

Abbreviations: **AHGs,** anti-hyperglycaemic agents; **CI,** confidence interval; **GLP1-RAs,** glucagon-like peptide-1 receptor agonists; **RCTs,** randomised controlled trials; **RR,** risk ratio.

Note: The GRADE scores were from the fixed-effect model.

GRADE evidence

a. Two trials with small weight GLP1-RAs vs. AHGs (ischaemic stroke [1.5%] and haemorrhagic stroke [0.6%]) with the overall EE rated as high risk of bias due to lack of blinding (open-label design) out of seven trials.

b. The overall imprecision was precise, with no significant effect size difference (P=0.08). However, all trials reported narrow CIs. The 95% CI was consistent with the possibility of a benefit not exceeding the MID, including only 181 events with a large sample size.

c. The overall imprecision was precise, with no significant effect size difference (P=0.10). However, all trials reported overlapping and narrow CIs. The 95% CI was consistent with the possibility of a benefit not exceeding the MID, including only 178 events with a large sample size.

d. The overall imprecision was precise, with no significant effect size difference (P=0.49). However, all trials reported overlapping and narrow CIs. The 95% CI was consistent with the possibility of a benefit not exceeding the MID, including only one event with a small sample size (911).

e. The overall imprecision was precise, with no significant effect size difference (P=0.73). However, all trials reported overlapping CIs, in which one trial reported a wide CI. The 95% CI was consistent with the possibility of a benefit not exceeding the MID, including only two events with a small sample size (1,730).

In summary, the overall certainty of the pooled EE had low imprecision without a significant effect size difference in the level of evidence (P=0.08). However, the majority of trials reported overlapping CIs, among which one trial reported a wide CI. All trials crossed the line of no difference (1). The 95% CI was consistent with the possibility of a benefit not exceeding the MID, including a total of 181 events with a large sample size (46,097). There was no evidence of inconsistency, statistically significant heterogeneity (P=0.92; I^2=0%), or subgroup difference (P=0.86; I^2=0%). The majority of trials had low risk of bias (97.9%). There was no evidence of detection or selection bias. The indirectness of the trials indicated a high level of evidence, with an individual trial PICOS element aligned closely to the review PICOS. Finally,

there was no evidence of reporting bias; all included trials were within the two boundaries of the pyramid.

DPP4-Is and stroke by baseline characteristic

Use of DPP4-Is did not decrease stroke risk in patients with type 2 diabetes mellitus (T2DM) at risk of cardiovascular disease (CVD) or chronic kidney disease (CKD) (risk ratio (RR), 0.91; 95% confidence interval (CI), 0.83−1.01; P=0.07) versus non-DPP4-Is using fixed- and random-effect models (Figure S110 and Figure S111). The GRADE scores were moderate (Table S).

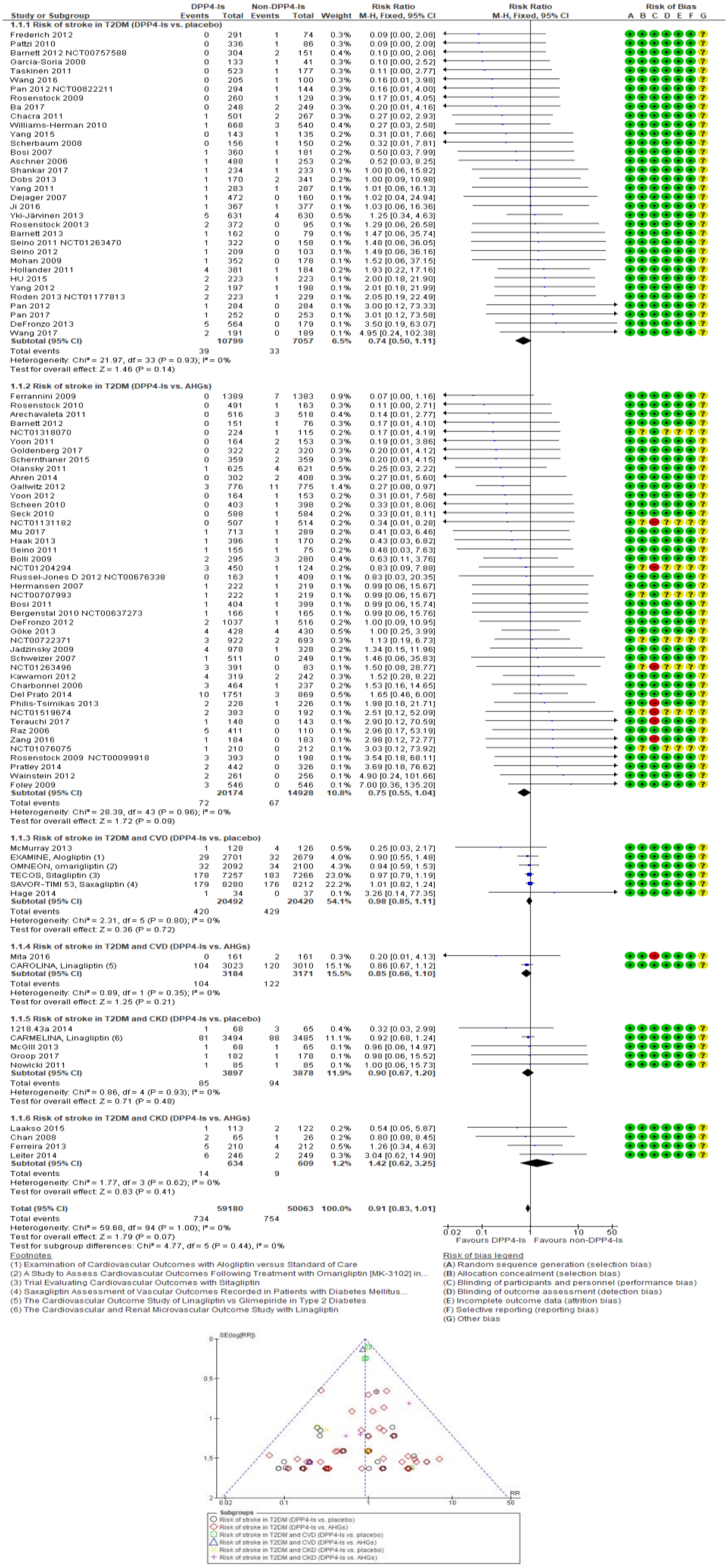

Figure S110. Forest and funnel plot of DPP4-Is and stroke by baseline characteristic, Fixed-effect model.

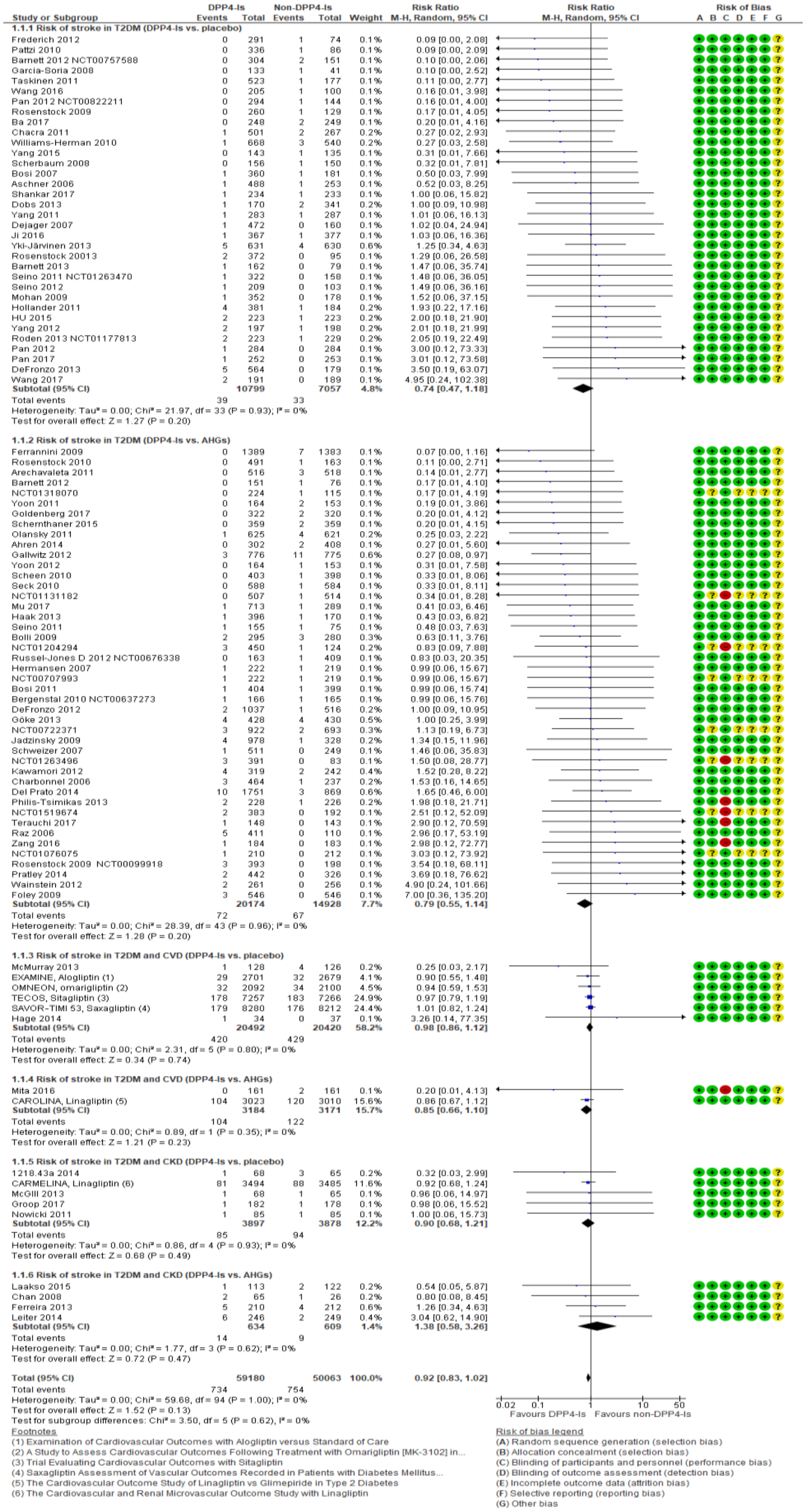

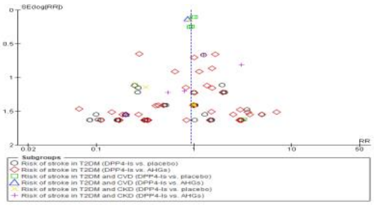

Figure S111. Forest and funnel plot of DPP4-Is and stroke by baseline characteristic, Random-effect model.

Table S13. GRADE evidence profile of DPP4-Is and stroke by baseline characteristic

Certainty assessment							Summary of findings				
№ of participants (trials)	Risk of bias	Inconsistency	Indirectness	Imprecision	Publication bias	Overall certainty of evidence	Study event rates (%)		Relative effect (95% CI)	Anticipated absolute effects	
							With placebo / controls	With DPP4-Is		Risk with placebo / controls	Risk difference with DPP4-Is
**Risk of stroke in T2DM by baseline characteristic (follow-up: range 4-432 weeks)**											
109243 (95 RCTs)	serious[a]	not serious	not serious	not serious	none	⊕⊕⊕◯ MODERATE	754/50063 (1.5%)	734/59180 (1.2%)	RR 0.91 (0.83 to 1.01)	2 per 100	0 fewer per 100 (from 0 fewer to 0 fewer)
**Risk of stroke in T2DM with DPP4-Is vs. placebo (follow-up: range 4-206 weeks)**											
17856 (34 RCTs)	not serious	not serious	not serious	very serious[b]	none	⊕⊕◯◯ LOW	33/7057 (0.5%)	39/10799 (0.4%)	RR 0.74 (0.50 to 1.11)	0 per 100	0 fewer per 100 (from 0 fewer to 0 fewer)
**Risk of stroke in T2DM with DPP4-Is vs. AHGs (follow-up: range 4-104 weeks)**											
35102 (44 RCTs)	serious[a]	not serious	not serious	very serious[c]	none	⊕◯◯◯ VERY LOW	67/14928 (0.4%)	72/20174 (0.4%)	RR 0.75 (0.55 to 1.04)	0 per 100	0 fewer per 100 (from 0 fewer to 0 fewer)
**Risk of stroke in T2DM and CVD with DPP4-Is vs. placebo (follow-up: range 12-157 weeks)**											
40912 (6 RCTs)	not serious	not serious	not serious	serious[d]	none	⊕⊕⊕⊕ MODERATE	429/20420 (2.1%)	420/20492 (2.0%)	RR 0.98 (0.85 to 1.11)	2 per 100	0 fewer per 100 (from 0 fewer to 0 fewer)
**Risk of stroke in T2DM and CVD with DPP4-Is vs. AHGs (follow-up: range 104-432 weeks)**											
6355 (2 RCTs)	serious[a]	not serious	not serious	very serious[e]	none	⊕◯◯◯ VERY LOW	112/3171 (3.8%)	104/3184 (3.3%)	RR 0.58 (0.66 to 1.10)	4 per 100	1 fewer per 100 (from 1 fewer to 0 fewer)
**Risk of stroke in T2DM and CKD with DPP4-Is vs. placebo (follow-up: range 24-224 weeks)**											
7775 (5 RCTs)	not serious	not serious	not serious	very serious[f]	none	⊕⊕◯◯ LOW	94/3878 (2.4%)	85/3897 (2.2%)	RR 0.90 (0.67 to 1.20)	2 per 100	0 fewer per 100 (from 1 fewer to 0 fewer)
**Risk of stroke in T2DM and CKD with DPP4-Is vs. AHGs (follow-up: range 52-54 weeks)**											
1243 (4 RCTs)	not serious	not serious	not serious	very serious[g]	none	⊕⊕◯◯ LOW	9/609 (1.5%)	14/634 (2.2%)	RR 1.42 (0.62 to 3.25)	1 per 100	1 more per 100 (from 1 fewer to 3 more)

***Abbreviations:* AHG,** anti-hyperglycaemic agent; **CVD,** cardiovascular disease; **CI,** confidence interval; **CKD,** chronic kidney disease; **DPP4-Is,** dipeptidyl peptidase-4 inhibitors; **RCTs,** randomised controlled trials; **RR,** risk ratio; **T2DM,** type 2 diabetes mellitus.

Note: The GRADE scores were from the fixed-effect model.

GRADE evidence

a. Eight trials with small weight DPP4-Is vs. AHGs (in T2DM [0.2%, 0.2%, 0.1%, 0.1%, 0.1%, 0.1%, and 0.1%] and in T2DM with CVD [0.3%]) with the overall effect estimate (EE) rated as high risk of bias due to lack of blinding (open-label design) out of 95 trials, four were unpublished randomised controlled trials (RCTs).

b. The overall imprecision was precise, with no significant effect size difference (P=0.14). However, all trials reported overlapping CIs, in which 17 trials reported wide CIs. The 95% CI was consistent with the possibility of a benefit not exceeding a minimal important difference (MID), including only 72 events with a large sample size.

c. The overall imprecision was precise, with no significant effect size difference (P=0.09). However, all trials reported overlapping CIs, in which 19 trials reported wide CIs. The 95% CI was consistent with the possibility of a benefit not exceeding the MID, including only 139 events with a large sample size.

d. The overall imprecision was precise, with no significant effect size difference (P=0.72). However, all trials reported overlapping CIs, in which one trial reported a wide CI. The 95% CI was consistent with the possibility of a large benefit exceeding the MID, including only 849 events with a large sample size.

e. The overall imprecision was precise, with no significant effect size difference (P=0.21). However, all trials reported narrow and overlapping CIs. The 95% CI was consistent with the possibility of a benefit not exceeding the MID, including only 226 events with a large sample size.

f. The overall imprecision was precise, with no significant effect size difference (P=0.48). However, all trials reported overlapping CIs, in which three trials reported wide CIs. The 95%

CI was consistent with the possible benefit of not exceeding the MID, including only 179 events with a large sample size.

g. The overall imprecision was precise, with no significant effect size difference (P=0.41). However, all trials reported overlapping CIs, in which one trial reported a wide CI. The 95% CI was consistent with the possibility of a benefit not exceeding the MID, including only 23 events with a small sample size (1,243).

In summary, the overall certainty of the pooled EE had moderate imprecision without a significant effect size difference in the level of evidence (P=0.07). However, the majority of trials reported overlapping CIs, in which 41 trials reported wide CIs. In total, only one small trial (1.4%) did not cross the line of no difference (1), in which there was a significant reduction of stroke in the direction of DPP4-Is favouring active comparators of AHGs in people with T2DM. The 95% CI was consistent with the possibility of a large benefit exceeding the MID, including a total of 1,488 events with a large sample size (109,243). There was no evidence of inconsistency, statistically significant heterogeneity (P=1.00; I^2=0%), or subgroup difference (P=0.44; I^2=0%). The majority of trials had low risk of bias (98.8%). There was no evidence of detection bias. However, the magnitude of the impact of some trials that suffered from limitations likely resulted in biased assessment of the intervention effect due to performance and selection bias (eight trials with open-label design out of 95 with. The indirectness of the trials indicated a high level of evidence, with an individual trial patient, intervention, comparison, outcome, and study design (PICOS) element aligned closely to the review PICOS. Finally, there was no evidence of reporting bias; all included trials were within the two boundaries of the pyramid.

DPP4-Is and non-fatal stroke by subtypes

The incidence of stroke with DPP4-Is in patients with T2DM did not decrease (RR, 0.93; 95% CI, 0.83−1.03; P=0.16) for either ischaemic (RR, 0.95; 95% CI, 0.84−1.08; P=0.45) or haemorrhagic (RR, 0.69; 95% CI, 0.20−2.43; P=0.56) events versus placebo, or for either ischaemic (RR, 0.84; 95% CI, 0.68−1.04; P=0.11) or haemorrhagic (RR, 1.27; 95% CI, 0.53−3.03; P=0.59) events versus anti-hyperglycaemic agents (AHGs), using random-effect model (Figure S112). The GRADE scores were moderate (Table S).

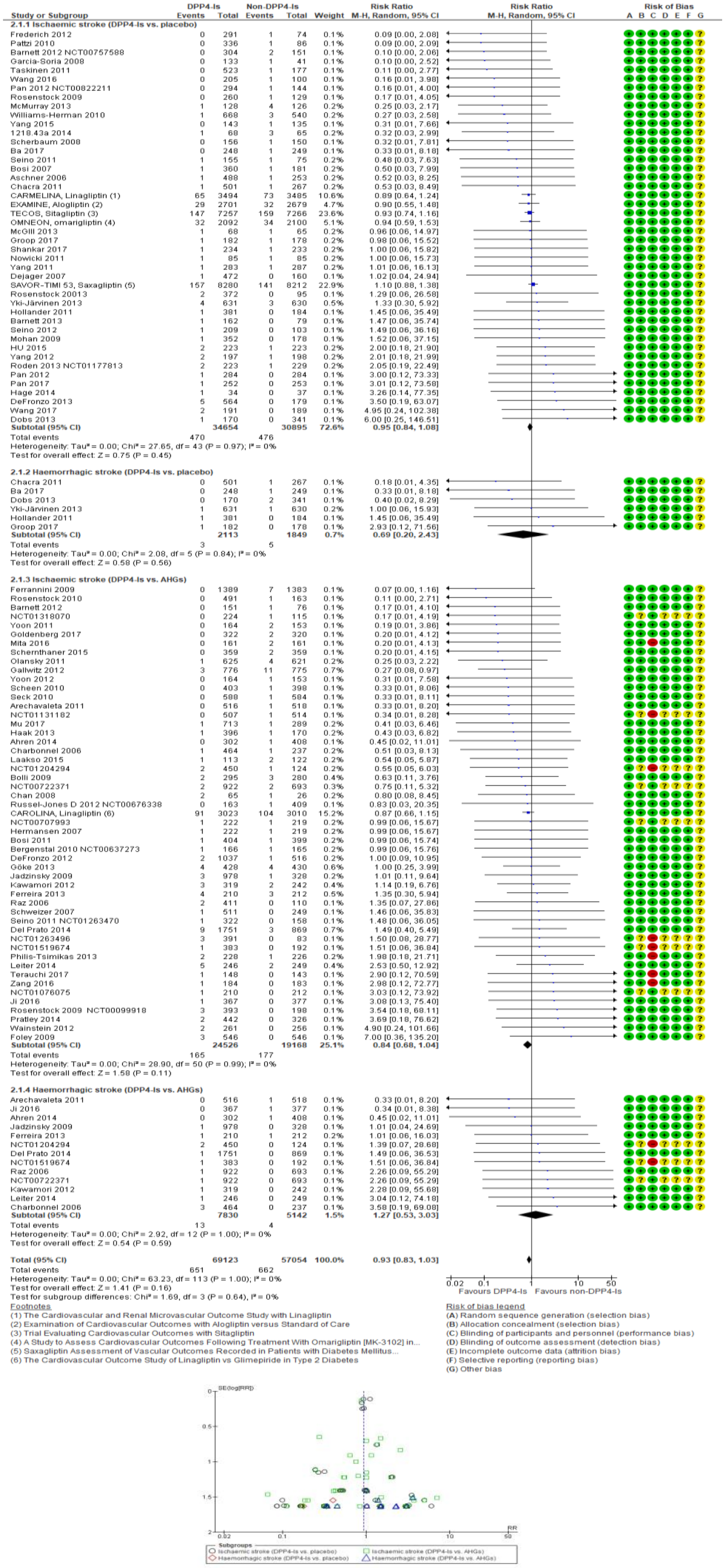

Figure S112. Forest and funnel plot of DPP4-Is and non-fatal stroke by subtypes, Random-effect model.

Table S14. GRADE evidence profile of DPP4-Is and non-fatal stroke by subtypes

Certainty assessment							Summary of findings				
**№ of participants (trials)**	**Risk of bias**	**Inconsistency**	**Indirectness**	**Imprecision**	**Publication bias**	**Overall certainty of evidence**	**Study event rates (%)**		**Relative effect (95% CI)**	**Anticipated absolute effects**	
							**With placebo / controls**	**With DPP4-Is**		**Risk with placebo / controls**	**Risk difference with DPP4-Is**
**DPP4-Is and non-fatal stroke by subtypes (follow-up: range 4-432 weeks)**											
126177 (95 RCTs)	serious[a]	not serious	not serious	not serious	none	⊕⊕⊕◯ MODERATE	662/57054 (1.2%)	651/69123 (0.9%)	RR 0.92 (0.82 to 1.02)	1 per 100	0 fewer per 100 (from 0 fewer to 0 fewer)
**Ischaemic stroke and DPP4-Is vs. placebo (follow-up: range 4-224 weeks)**											
65549 (44 RCTs)	not serious	not serious	not serious	serious[b]	none	⊕⊕⊕◯ MODERATE	476/30895 (1.5%)	470/34654 (1.4%)	RR 0.95 (0.84 to 1.07)	2 per 100	0 fewer per 100 (from 0 fewer to 0 fewer)
**Haemorrhagic stroke and DPP4-Is vs. placebo (follow-up: range 24-76 weeks)**											
3962 (6 RCTs)	not serious	not serious	not serious	very serious[c]	none	⊕⊕◯◯ LOW	5/1849 (0.3%)	3/2113 (0.1%)	RR 0.68 (0.22 to 2.12)	0 per 100	0 fewer per 100 (from 0 fewer to 0 fewer)
**Ischaemic stroke and DPP4-Is vs. AHGs (follow-up: range 4-432 weeks)**											
43694 (51 RCTs)	serious[a]	not serious	not serious	serious[d]	none	⊕⊕◯◯ LOW	177/19168 (0.9%)	165/24526 (0.7%)	RR 0.82 (0.67 to 1.01)	1 per 100	0 fewer per 100 (from 0 fewer to 0 fewer)
**Haemorrhagic stroke and DPP4-Is vs. AHGs (follow-up: range 24-162 weeks)**											
12972 (13 RCTs)	serious[a]	not serious	not serious	very serious[e]	none	⊕◯◯◯ VERY LOW	4/5142 (0.1%)	13/7830 (0.2%)	RR 1.29 (0.58 to 2.90)	0 per 100	0 fewer per 100 (from 0 fewer to 0 fewer)

Abbreviations: **AHGs,** anti-hyperglycaemic agents; **CI,** confidence interval; **DPP4-Is,** dipeptidyl peptidase-4 inhibitors; **RCTs,** randomised controlled trials; **RR,** risk ratio.

Note: The GRADE scores were from the fixed-effect model.

GRADE evidence

a. Ten trials with small weight DPP4-Is vs. AHGs (ischaemic stroke [0.4%, 0.2%, 0.2%, 0.1%, 0.1%, 0.1%, 0.1%, and 0.1%] and haemorrhagic stroke [0.1% and 0.1%]) with the overall EE rated as high risk of bias due to lack of blinding (open-label design) out of 95 trials, six of which were unpublished RCTs.

b. The overall imprecision was precise, with no significant effect size difference (P=0.40). However, all trials reported overlapping CIs, in which 20 trials reported wide CIs. The 95% CI was consistent with the possibility of a large benefit exceeding the MID, including only 846 events with a large sample size.

c. The overall imprecision was precise, with no significant effect size difference (P=0.51). However, all trials reported overlapping CIs, in which three trials reported wide CIs. The 95% CI was consistent with the possibility of a small benefit not exceeding the MID, including only eight events with a large sample size.

d. The overall imprecision was precise, with no significant size effect difference (P=0.06). However, all trials reported overlapping CIs, in which 21 trials reported wide CIs. The 95% CI was consistent with the possibility of a large benefit exceeding the MID, including only 342 events with a large sample size.

e. The overall imprecision was precise, with no significant effect size difference (P=0.54). However, all trials reported overlapping CIs, in which 11 trials reported wide CIs. The 95% CI was consistent with the possibility of a small benefit not exceeding the MID, including only 17 events with a large sample size.

In summary, the overall certainty of the pooled EE had moderate imprecision without a significant effect size difference in the level of evidence (P=0.10). However, the majority of trials reported overlapping CIs, in which 55 trials reported wide CIs. In total, only one small trial (1.5%) did not cross the line of no difference (1), in which there was a significant reduction

in non-fatal stroke in the direction of DPP4-Is favouring active comparators of AHGs. The 95% CI was consistent with the possibility of a large benefit exceeding the MID, including a total of 1,313 events with a large sample size (126,177). There was no evidence of inconsistency, statistically significant heterogeneity (P=1.00; I^2=0%), or subgroup difference (P=0.50; I^2=0%). The majority of trials had low risk of bias (98.5%). There was no evidence of detection bias. However, the magnitude of the impact of some trials that suffered from limitations likely resulted in biased assessment of the intervention effect due to performance and selection bias (ten trials with open-label design out of 95). The indirectness of the trials indicated a high level of evidence, with an individual trial PICOS element aligned closely to the review PICOS. Finally, there was no evidence of reporting bias; all included trials were within the two boundaries of the pyramid.

Fatal stroke risk

DPP4-Is and fatal stroke risk

In 10 trials (48,102 participants; 181 strokes), use of DPP4-Is did not decrease risk of fatal ischaemic or haemorrhagic stroke (RR, 0.93; 95% CI, 0.70−1.24; P=0.64) versus non-DPP4-Is using fixed- and random-effect models (Figure S113 and Figure S114). The GRADE scores were moderate (Table S).

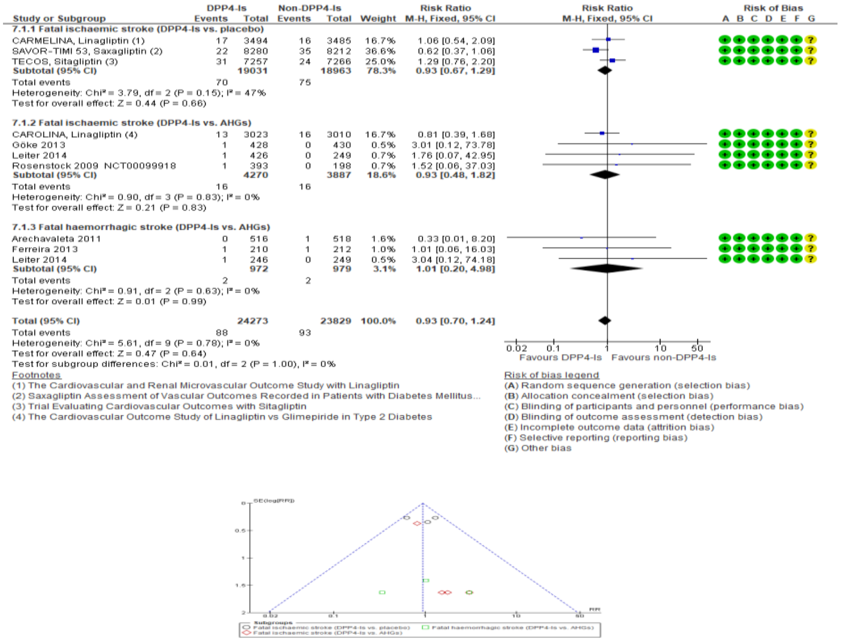

Figure S113. Forest and funnel plot of DPP4-Is and fatal stroke, Fixed-effect model.

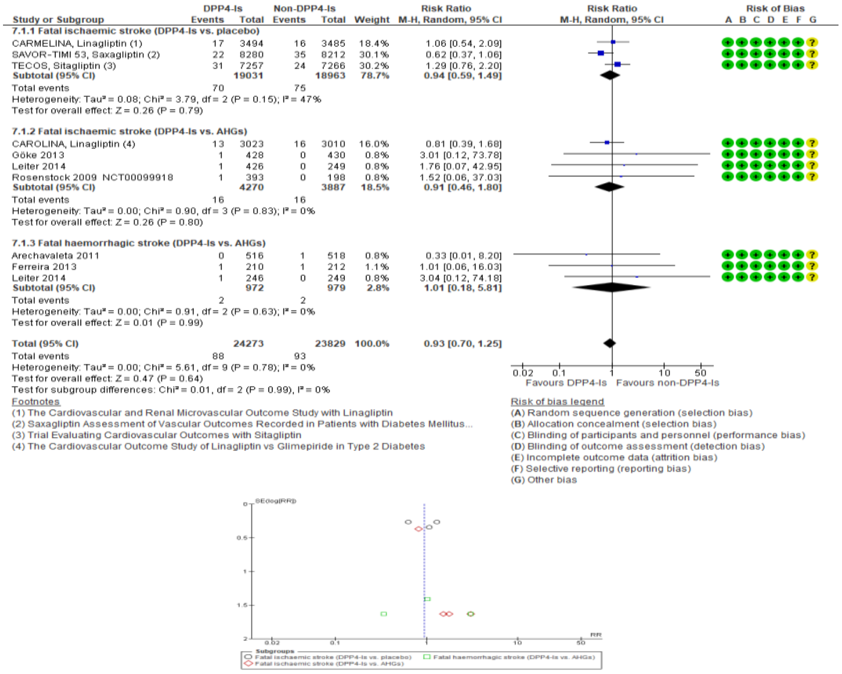

Figure S114. Forest and funnel plot of DPP4-Is and fatal stroke, Random-effect model.

Table S15. GRADE evidence profile of DPP4-Is and fatal stroke

Certainty assessment							Summary of findings				
№ of participants (trials)	Risk of bias	Inconsistency	Indirectness	Imprecision	Publication bias	Overall certainty of evidence	Study event rates (%)		Relative effect (95% CI)	Anticipated absolute effects	
							With placebo / controls	With DPP4-Is		Risk with placebo / controls	Risk difference with DPP4-Is
**DPP4-Is and fatal stroke (follow-up: range 4-224 weeks)**											
48102 (10 RCTs)	not serious	not serious	not serious	serious[a]	none	⊕⊕⊕◯ MODERATE	93/23829 (0.4%)	88/24273 (0.4%)	RR 0.93 (0.70 to 1.24)	0 per 100	0 fewer per 100 (from 0 fewer to 0 fewer)
**Ischaemic fatal stroke and DPP4-Is vs. placebo (follow-up: range 109-224 weeks)**											
37994 (3 RCTs)	not serious	not serious	not serious	very serious[b]	none	⊕⊕◯◯ LOW	75/18963 (0.4%)	70/19031 (0.4%)	RR 0.93 (0.67 to 1.29)	0 per 100	0 fewer per 100 (from 0 fewer to 0 fewer)
**Ischaemic fatal stroke and DPP4-Is vs. AHGs (follow-up: range 52-432 weeks)**											
8157 (4 RCTs)	not serious	not serious	not serious	very serious[c]	none	⊕⊕◯◯ LOW	16/3887 (0.4%)	16/4270 (0.4%)	RR 0.93 (0.48 to 1.82)	0 per 100	0 fewer per 100 (from 0 fewer to 0 fewer)
**Haemorrhagic fatal stroke and DPP4-Is vs. AHGs (follow-up: range 30-54 weeks)**											
1951 (3 RCTs)	not serious	not serious	not serious	very serious[d]	none	⊕⊕◯◯ LOW	2/979 (0.2%)	2/972 (0.2%)	RR 1.01 (0.20 to 4.98)	0 per 100	0 fewer per 100 (from 0 fewer to 1 more)

Abbreviations: **AHGs,** anti-hyperglycaemic agents; **CI,** confidence interval; **DPP4-Is,** dipeptidyl peptidase-4 inhibitors; **RCTs,** randomised controlled trials; **RR,** risk ratio.

Note: The GRADE scores were from the fixed-effect model.

GRADE evidence

a. The overall imprecision was precise, with no significant effect size difference (P=0.64). The CIs were precise and narrow. The 95% CI was consistent with the possibility of a benefit not exceeding the MID; however, it included only 181 events with a large sample size (48,102).

b. The overall imprecision was precise, with no significant effect size difference (P=0.66). However, all trials reported overlapping CIs with narrow CIs. The 95% CI was consistent with the possibility of a benefit not exceeding the MID, including only 145 events with a large sample size.

c. The overall imprecision was precise, with no significant effect size difference (P=0.83). However, all trials reported overlapping CIs, in which three trials reported wide CIs. The 95% CI was consistent with the possibility of a benefit not exceeding the MID, including only 32 events with a large sample size.

d. The overall imprecision was precise, with no significant effect size difference (P=0.99). However, all trials reported overlapping CIs, in which two trials reported wide CIs. The 95% CI was consistent with the possibility of a benefit not exceeding the MID, including only four events with a small sample size (1,951).

In summary, the overall certainty of the pooled EE had moderate imprecision without a significant effect size difference in the level of evidence (P=0.64). However, all trials reported overlapping CIs, in which five trials reported wide CIs. All trials crossed the line of no difference (1). The 95% CI was consistent with the possible benefit of not exceeding the MID, including a total of 181 events with a large sample size (48,102). There was no evidence of inconsistency, statistically significant heterogeneity (P=0.78; I^2=0%), or subgroup difference (P=1.00; I^2=0%). All trials had low risk of bias (100%). There was no evidence of detection or selection bias. The indirectness of the trials indicated a high level of evidence, with an individual trial PICOS element aligned closely to the review PICOS. Finally, there was no evidence of reporting bias; all included trials were within the two boundaries of the pyramid.

Stroke risk by clinical trial size

SGLT2-Is and stroke by clinical trial size

Use of SGLT2-Is did not decrease stroke risk in the pooled analysis of large RCTs (RR, 0.98; 95% CI, 0.88−1.09; P=0.66) or small RCTs (RR, 0.69; 95% CI, 0.46−1.05; P=0.08) versus placebo, or in small RCTs (RR, 1.01; 95% CI, 0.64−1.59; P=0.96) versus AHGs using fixed- and random-effect models (Figure S15 and Figure S16). The GRADE scores were moderate (Table S13).

Study or Subgroup	SGLT2-Is Events	SGLT2-Is Total	Non-SGLT2-Is Events	Non-SGLT2-Is Total	Weight	Risk Ratio M-H, Fixed, 95% CI
**12.1.1 SGLT2-Is vs. placebo (small RCTs)**						
Stenlof 2012	0	392	2	192	0.4%	0.10 [0.00, 2.04]
Kovacs 2014	0	333	2	165	0.4%	0.10 [0.00, 2.06]
Kaku 2014	0	174	2	87	0.4%	0.10 [0.00, 2.07]
Bailey 2010	1	409	2	137	0.4%	0.17 [0.02, 1.83]
Henry 2012 (Study1)	0	397	1	201	0.3%	0.17 [0.01, 4.13]
Cai 2018	0	108	2	108	0.3%	0.20 [0.01, 4.12]
Wilding 2014	2	610	3	197	0.6%	0.22 [0.04, 1.28]
NCT01649297	2	876	1	107	0.2%	0.24 [0.02, 2.67]
Leiter 2014 NCT01042977	1	482	4	483	0.5%	0.25 [0.03, 2.23]
Roden 2013	1	534	1	229	0.2%	0.43 [0.03, 6.83]
Kadowaki 2015	1	752	0	109	0.1%	0.44 [0.02, 10.69]
NCT01137474	1	633	1	311	0.2%	0.49 [0.03, 7.83]
Rosenstock 2014	1	375	1	188	0.2%	0.50 [0.03, 7.97]
Ji 2015	1	450	1	226	0.2%	0.50 [0.03, 7.99]
Weir 2014	3	358	1	78	0.2%	0.65 [0.07, 6.20]
Cefalu 2015	2	460	2	462	0.3%	1.00 [0.14, 7.10]
Bailey 2014	1	199	0	75	0.1%	1.14 [0.05, 27.68]
Ha¨ring 2013	1	359	0	141	0.1%	1.18 [0.05, 28.88]
Barnett 2014	5	419	3	319	0.4%	1.27 [0.31, 5.27]
Merker 2015	1	430	0	207	0.1%	1.45 [0.06, 35.39]
NCT01210001	1	333	0	165	0.1%	1.49 [0.06, 36.40]
Wilding 2013	1	313	0	156	0.1%	1.50 [0.06, 36.61]
Kohan 2014	1	168	0	84	0.1%	1.51 [0.06, 36.65]
Ji 2014	1	261	0	132	0.1%	1.52 [0.06, 37.13]
Strojek 2011	2	450	0	146	0.1%	1.63 [0.08, 33.75]
Dagogo-Jack 2018	2	309	0	153	0.1%	2.48 [0.12, 51.42]
Rosenstock 2015 NCT01011868	5	324	1	170	0.2%	2.62 [0.31, 22.28]
Yang 2018	1	139	0	133	0.1%	2.87 [0.12, 69.87]
Bolinder 2014	1	91	0	91	0.1%	3.00 [0.12, 72.69]
Bode 2015	5	208	0	104	0.1%	5.53 [0.31, 99.00]
**Subtotal (95% CI)**		**11346**		**5356**	**6.5%**	**0.69 [0.46, 1.05]**
Total events	44		30			
Heterogeneity: Chi² = 19.24, df = 29 (P = 0.92); I² = 0%						
Test for overall effect: Z = 1.75 (P = 0.08)						
**12.1.2 SGLT2-Is vs. placebo (large RCTs)**						
CREDENCE, Canagliflozin (1)	62	2202	80	2199	10.4%	0.77 [0.56, 1.07]
CANVAS, Canagliflozin	166	5795	147	4347	21.7%	0.85 [0.68, 1.05]
DECLARE-TIMI 58, Dapagliflozin (2)	235	8582	231	8578	29.9%	1.02 [0.85, 1.22]
VERTIS CV, Ertugliflozin (3)	185	5499	87	2747	15.0%	1.06 [0.83, 1.36]
EMPA-REG OUTCOME, Empagliflozin (4)	164	4687	69	2333	11.9%	1.18 [0.90, 1.56]
**Subtotal (95% CI)**		**26765**		**20204**	**88.9%**	**0.98 [0.88, 1.09]**
Total events	812		614			
Heterogeneity: Chi² = 6.05, df = 4 (P = 0.20); I² = 34%						
Test for overall effect: Z = 0.43 (P = 0.66)						
**12.1.3 SGLT2-Is vs. AHGs (small RCTs)**						
Miller 2018	0	194	1	97	0.3%	0.17 [0.01, 4.07]
Ferrannini 2013 (Study 2)	3	332	3	56	0.7%	0.17 [0.03, 0.81]
Hadjadj 2016	1	1032	1	341	0.2%	0.33 [0.02, 5.27]
Araki 2015	3	1097	0	63	0.1%	0.41 [0.02, 7.81]
Ferrannini 2013 (Study 1)	1	215	0	56	0.1%	0.79 [0.03, 19.18]
Ridderstra°le 2014	14	765	15	780	1.9%	0.95 [0.46, 1.96]
Schernthaner 2013	1	377	1	378	0.1%	1.00 [0.06, 15.97]
Rosenstock 2016	2	949	0	237	0.1%	1.25 [0.06, 26.00]
DeFronzo 2015	6	546	1	128	0.2%	1.41 [0.17, 11.58]
Rosenstock 2015	1	358	0	176	0.1%	1.48 [0.06, 36.12]
Cefalu 2013	9	968	3	482	0.5%	1.49 [0.41, 5.49]
Forst 2014	1	227	0	115	0.1%	1.53 [0.06, 37.18]
Nauck 2014	2	406	0	408	0.1%	5.02 [0.24, 104.33]
Lavalle-Gonzales 2013	4	735	0	549	0.1%	6.73 [0.36, 124.66]
**Subtotal (95% CI)**		**8201**		**3866**	**4.5%**	**1.01 [0.64, 1.59]**
Total events	48		25			
Heterogeneity: Chi² = 10.50, df = 13 (P = 0.65); I² = 0%						
Test for overall effect: Z = 0.05 (P = 0.96)						
**Total (95% CI)**		**46312**		**29426**	**100.0%**	**0.96 [0.87, 1.06]**
Total events	904		669			
Heterogeneity: Chi² = 38.13, df = 48 (P = 0.85); I² = 0%						
Test for overall effect: Z = 0.81 (P = 0.42)						
Test for subgroup differences: Chi² = 2.58, df = 2 (P = 0.27), I² = 22.6%						

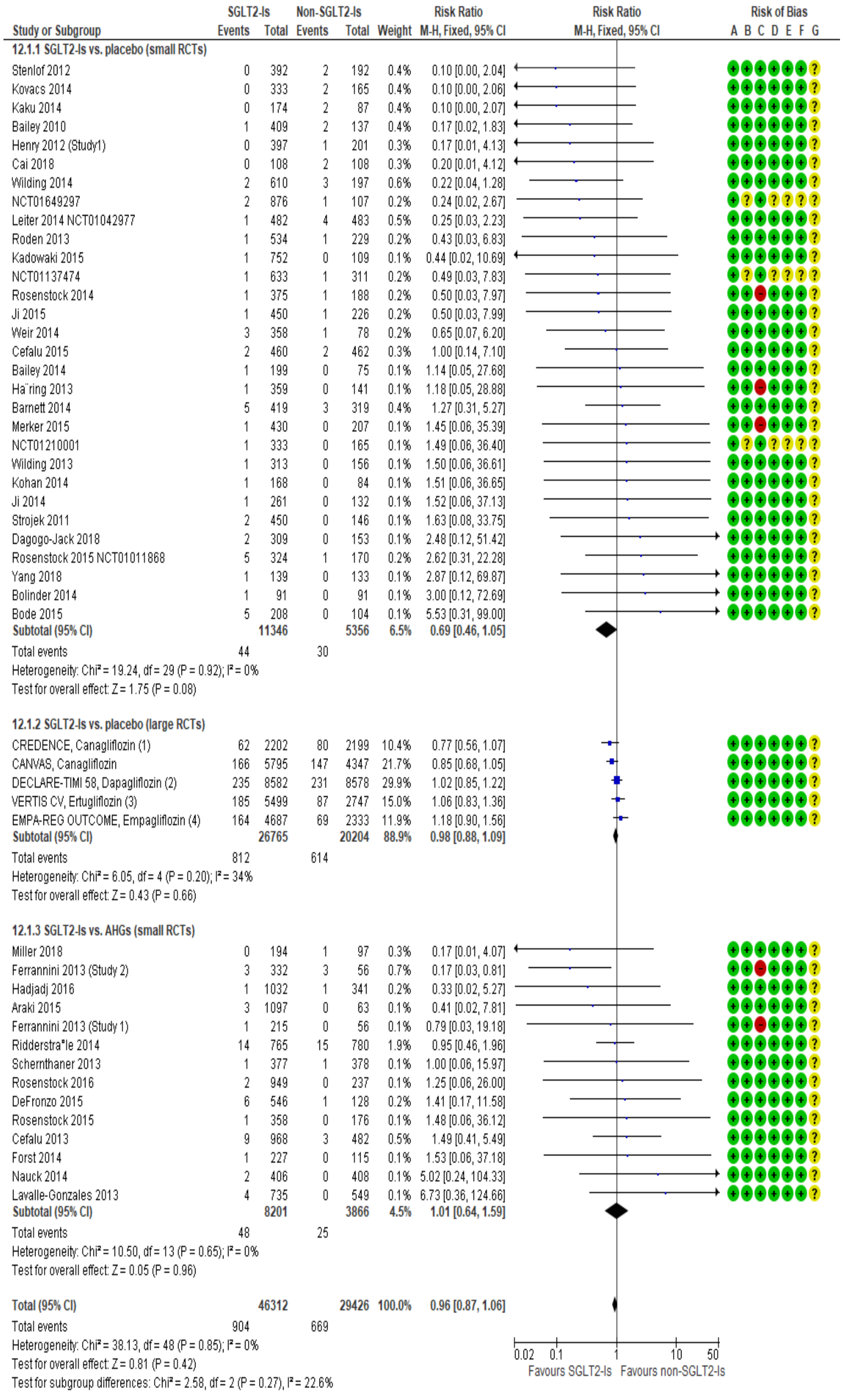

Footnotes
(1) Canagliflozin and Renal Events in Diabetes with Established Nephropathy Clinical Evaluaation
(2) Multicentre Trial to Evaluate the Effect of Dapagliflozin on the Incidence of Cardiovascular Events
(3) Evaluation of Ertugliflozin Efficacy and Safety Cardiovascular Outcomes
(4) Empagliflozin Cardiovascular Outcome Event in Type 2 Diabetes Mellitus Patients

Risk of bias legend
(A) Random sequence generation (selection bias)
(B) Allocation concealment (selection bias)
(C) Blinding of participants and personnel (performance bias)
(D) Blinding of outcome assessment (detection bias)
(E) Incomplete outcome data (attrition bias)
(F) Selective reporting (reporting bias)
(G) Other bias

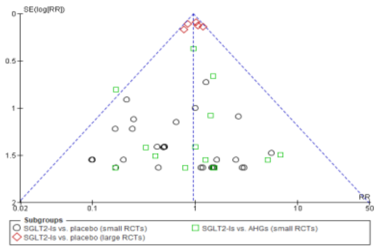

Figure S15. Forest and funnel plot of SGLT2-Is and stroke by clinical trial size, Fixed-effect model.

Study or Subgroup	SGLT2-Is Events	SGLT2-Is Total	Non-SGLT2-Is Events	Non-SGLT2-Is Total	Weight	Risk Ratio M-H, Random, 95% CI
**12.1.1 SGLT2-Is vs. placebo (small RCTs)**						
Stenlof 2012	0	392	2	192	0.1%	0.10 [0.00, 2.04]
Kovacs 2014	0	333	2	165	0.1%	0.10 [0.00, 2.06]
Kaku 2014	0	174	2	87	0.1%	0.10 [0.00, 2.07]
Bailey 2010	1	409	2	137	0.2%	0.17 [0.02, 1.83]
Henry 2012 (Study1)	0	397	1	201	0.1%	0.17 [0.01, 4.13]
Cai 2018	0	108	2	108	0.1%	0.20 [0.01, 4.12]
Wilding 2014	2	610	3	197	0.3%	0.22 [0.04, 1.28]
NCT01649297	2	876	1	107	0.2%	0.24 [0.02, 2.67]
Leiter 2014 NCT01042977	1	482	4	483	0.2%	0.25 [0.03, 2.23]
Roden 2013	1	534	1	229	0.1%	0.43 [0.03, 6.83]
Kadowaki 2015	1	752	0	109	0.1%	0.44 [0.02, 10.69]
NCT01137474	1	633	1	311	0.1%	0.49 [0.03, 7.83]
Rosenstock 2014	1	375	1	188	0.1%	0.50 [0.03, 7.97]
Ji 2015	1	450	1	226	0.1%	0.50 [0.03, 7.99]
Weir 2014	3	358	1	78	0.2%	0.65 [0.07, 6.20]
Cefalu 2015	2	460	2	462	0.3%	1.00 [0.14, 7.10]
Bailey 2014	1	199	0	75	0.1%	1.14 [0.05, 27.68]
Ha¨ring 2013	1	359	0	141	0.1%	1.18 [0.05, 28.88]
Barnett 2014	5	419	3	319	0.5%	1.27 [0.31, 5.27]
Merker 2015	1	430	0	207	0.1%	1.45 [0.06, 35.39]
NCT01210001	1	333	0	165	0.1%	1.49 [0.06, 36.40]
Wilding 2013	1	313	0	156	0.1%	1.50 [0.06, 36.61]
Kohan 2014	1	168	0	84	0.1%	1.51 [0.06, 36.65]
Ji 2014	1	261	0	132	0.1%	1.52 [0.06, 37.13]
Strojek 2011	2	450	0	146	0.1%	1.63 [0.08, 33.75]
Dagogo-Jack 2018	2	309	0	153	0.1%	2.48 [0.12, 51.42]
Rosenstock 2015 NCT01011868	5	324	1	170	0.2%	2.62 [0.31, 22.28]
Yang 2018	1	139	0	133	0.1%	2.87 [0.12, 69.87]
Bolinder 2014	1	91	0	91	0.1%	3.00 [0.12, 72.69]
Bode 2015	5	208	0	104	0.1%	5.53 [0.31, 99.00]
**Subtotal (95% CI)**		**11346**		**5356**	**4.5%**	**0.66 [0.41, 1.06]**
Total events	44		30			
Heterogeneity: Tau² = 0.00; Chi² = 19.24, df = 29 (P = 0.92); I² = 0%						
Test for overall effect: Z = 1.73 (P = 0.08)						
**12.1.2 SGLT2-Is vs. placebo (large RCTs)**						
CREDENCE, Canagliflozin (1)	62	2202	80	2199	9.5%	0.77 [0.56, 1.07]
CANVAS, Canagliflozin	166	5795	147	4347	21.1%	0.85 [0.68, 1.05]
DECLARE-TIMI 58, Dapagliflozin (2)	235	8582	231	8578	31.4%	1.02 [0.85, 1.22]
VERTIS CV, Ertugliflozin (3)	185	5499	87	2747	16.1%	1.06 [0.83, 1.36]
EMPA-REG OUTCOME, Empagliflozin (4)	164	4687	69	2333	13.2%	1.18 [0.90, 1.56]
**Subtotal (95% CI)**		**26765**		**20204**	**91.3%**	**0.97 [0.85, 1.11]**
Total events	812		614			
Heterogeneity: Tau² = 0.01; Chi² = 6.05, df = 4 (P = 0.20); I² = 34%						
Test for overall effect: Z = 0.40 (P = 0.69)						
**12.1.3 SGLT2-Is vs. AHGs (small RCTs)**						
Miller 2018	0	194	1	97	0.1%	0.17 [0.01, 4.07]
Ferrannini 2013 (Study 2)	3	332	3	56	0.4%	0.17 [0.03, 0.81]
Hadjadj 2016	1	1032	1	341	0.1%	0.33 [0.02, 5.27]
Araki 2015	3	1097	0	63	0.1%	0.41 [0.02, 7.81]
Ferrannini 2013 (Study 1)	1	215	0	56	0.1%	0.79 [0.03, 19.18]
Ridderstra°le 2014	14	765	15	780	1.9%	0.95 [0.46, 1.96]
Schernthaner 2013	1	377	1	378	0.1%	1.00 [0.06, 15.97]
Rosenstock 2016	2	949	0	237	0.1%	1.25 [0.06, 26.00]
DeFronzo 2015	6	546	1	128	0.2%	1.41 [0.17, 11.58]
Rosenstock 2015	1	358	0	176	0.1%	1.48 [0.06, 36.12]
Cefalu 2013	9	968	3	482	0.6%	1.49 [0.41, 5.49]
Forst 2014	1	227	0	115	0.1%	1.53 [0.06, 37.18]
Nauck 2014	2	406	0	408	0.1%	5.02 [0.24, 104.33]
Lavalle-Gonzales 2013	4	735	0	549	0.1%	6.73 [0.36, 124.66]
**Subtotal (95% CI)**		**8201**		**3866**	**4.3%**	**0.90 [0.55, 1.46]**
Total events	48		25			
Heterogeneity: Tau² = 0.00; Chi² = 10.50, df = 13 (P = 0.65); I² = 0%						
Test for overall effect: Z = 0.42 (P = 0.67)						
**Total (95% CI)**		**46312**		**29426**	**100.0%**	**0.96 [0.86, 1.06]**
Total events	904		669			
Heterogeneity: Tau² = 0.00; Chi² = 38.13, df = 48 (P = 0.85); I² = 0%						
Test for overall effect: Z = 0.89 (P = 0.37)						
Test for subgroup differences: Chi² = 2.48, df = 2 (P = 0.29), I² = 19.4%						

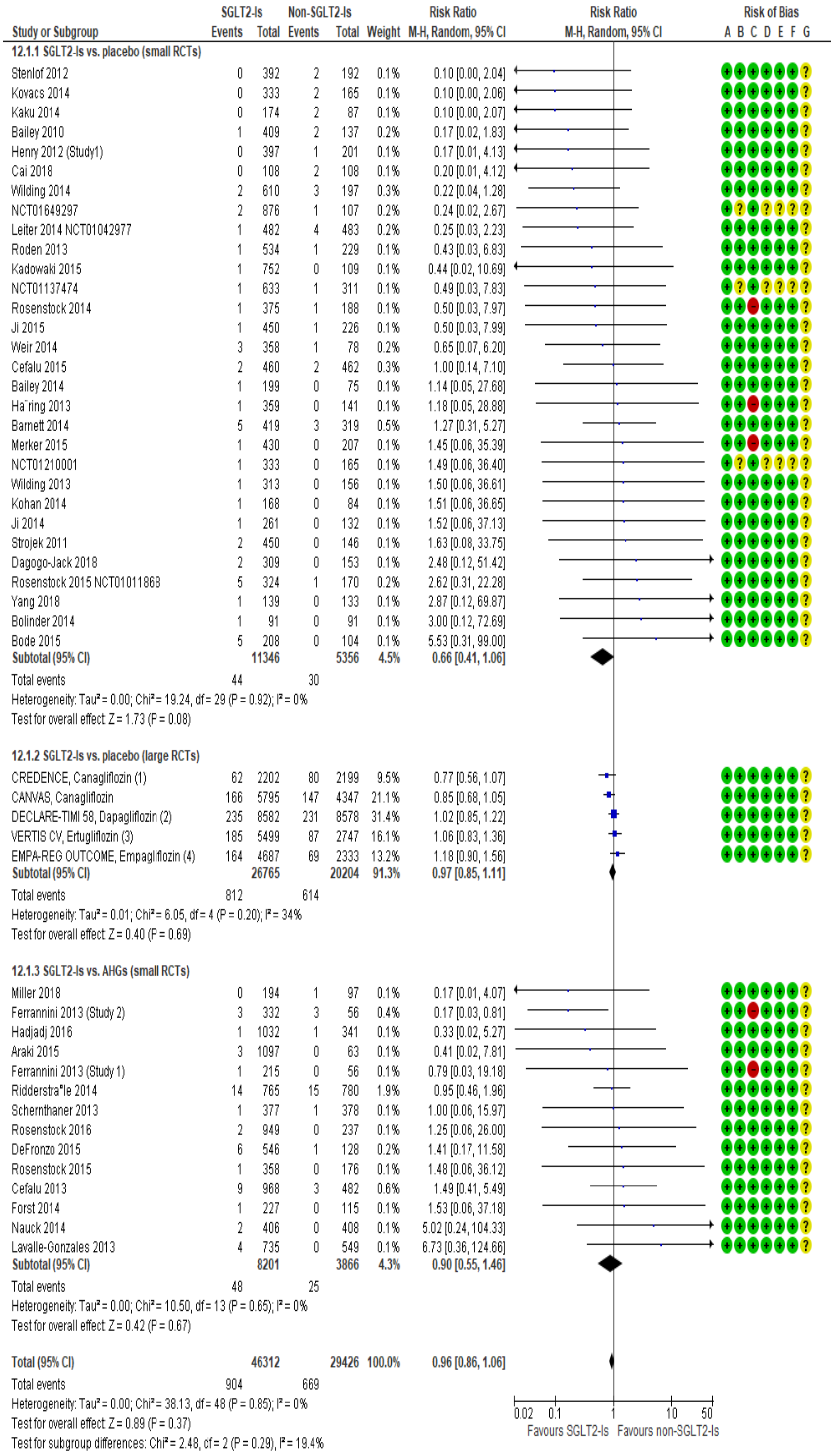

Footnotes
(1) Canagliflozin and Renal Events in Diabetes with Established Nephropathy Clinical Evaluaation
(2) Multicentre Trial to Evaluate the Effect of Dapagliflozin on the Incidence of Cardiovascular Events
(3) Evaluation of Ertugliflozin Efficacy and Safety Cardiovascular Outcomes
(4) Empagliflozin Cardiovascular Outcome Event in Type 2 Diabetes Mellitus Patients

Risk of bias legend
(A) Random sequence generation (selection bias)
(B) Allocation concealment (selection bias)
(C) Blinding of participants and personnel (performance bias)
(D) Blinding of outcome assessment (detection bias)
(E) Incomplete outcome data (attrition bias)
(F) Selective reporting (reporting bias)
(G) Other bias

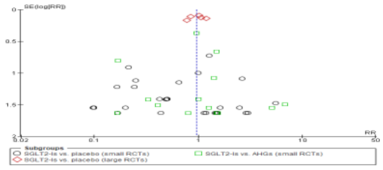

Figure S16. Forest and funnel plot of SGLT2-Is and stroke by clinical trial size, Random-effect model.

Table S13. GRADE evidence profile of SGLT2-Is and stroke by clinical trial size

Certainty assessment							Summary of findings				
№ of participants (trials)	Risk of bias	Inconsistency	Indirectness	Imprecision	Publication bias	Overall certainty of evidence	Study event rates (%)		Relative effect (95% CI)	Anticipated absolute effects	
							With placebo / controls	With SGLT2-Is		Risk with placebo / controls	Risk difference with SGLT2-Is
**Risk of stroke risk by clinical trial size (follow-up: range 12-318 weeks)**											
75738 (49 RCTs)	serious[a]	not serious	not serious	not serious	none	⨁⨁⨁◯ MODERATE	669/29426 (2.3%)	904/46312 (2.0%)	RR 0.96 (0.87 to 1.06)	2 per 100	0 fewer per 100 (from 0 fewer to 0 fewer)
**Small RCTs with SGLT2-Is vs. placebo (follow-up: range 12-318 weeks)**											
16702 (30 RCTs)	serious[a]	not serious	not serious	very serious[b]	none	⨁◯◯◯ VERY LOW	30/5356 (0.6%)	44/11346 (0.4%)	RR 0.69 (0.46 to 1.05)	1 per 100	0 fewer per 100 (from 0 fewer to 0 fewer)
**Large RCTs with SGLT2-Is vs. placebo (follow-up: range 161-219 weeks)**											
46969 (5 RCTs)	not serious	not serious	not serious	not serious	none	⨁⨁⨁⨁ HIGH	614/20204 (3.0%)	812/26765 (3.0%)	RR 0.98 (0.88 to 1.09)	3 per 100	0 fewer per 100 (from 0 fewer to 0 fewer)
**Small RCTs with SGLT2-Is vs. AHGs (follow-up: range 12-156 weeks)**											
12067 (14 RCTs)	serious[a]	not serious	not serious	very serious[c]	none	⨁◯◯◯ VERY LOW	25/3866 (0.6%)	48/8201 (0.6%)	RR 1.01 (0.64 to 1.59)	1 per 100	0 fewer per 100 (from 0 fewer to 0 fewer)

Abbreviations: **AHGs,** anti-hyperglycaemic agents; **CI,** confidence interval; **RCTs,** randomised controlled trials; **RR,** risk ratio; **SGLT2-Is,** sodium-glucose cotransporter-2 inhibitors.

Note: The GRADE scores were from the fixed-effect model.

GRADE evidence

a. Five trials with small weight SGLT2-Is vs. placebo (0.2%, 0.1%, and 0.1%) and SGLT2-Is vs. AHGs (0.7% and 0.1%) with the overall EE rated as high risk of bias due to lack of blinding (open-label design) out of 49 trials.

b. The overall imprecision was precise, with no significant effect size difference (P=0.08). However, all trials reported overlapping CIs, in which 13 trials reported wide CIs. The 95% CI was consistent with the possibility of a benefit not exceeding the MID, including only 74 events with a large sample size.

c. The overall imprecision was precise, with no significant effect size difference (P=0.96). However, all trials reported overlapping CIs, in which eight trials reported wide CIs. The 95% CI was consistent with the possibility of a benefit not exceeding the MID, including only 73 events with a large sample size.

In summary, the overall certainty of the pooled EE had moderate imprecision without a significant effect size difference in the level of evidence (P=0.42). However, the majority of trials reported overlapping CIs, in which 21 trials reported wide CIs. In total, only one small trial (0.7%) did not cross the line of no difference (1), in which there was a significant reduction in stroke in the direction of SGLT2-Is favouring active comparators of AHGs. The 95% CI was consistent with the possibility of a large benefit exceeding the MID, including a total of 1,573 events with a large sample size. There was no evidence of inconsistency, statistically significant heterogeneity (P=0.85; I^2=0%), or subgroup difference (P=0.27; I^2=22.6%). The majority of trials had low risk of bias (98.8%). There was no evidence of detection bias. However, the magnitude of the impact of some trials that suffered from limitations likely resulted in biased assessment of the intervention effect due to performance and selection bias (five trials with open-label design out of 49). The indirectness of the trials indicated a high level of evidence, with an individual trial PICOS element aligned closely to the review PICOS. Finally, there was no evidence of reporting bias. However, one trial reported an outlier outside the pyramid edge due to multiple interventional groups, consequently resulting in a very wide CI.

GLP1-Ras and stroke by clinical trial size

Use of GLP1-RAs decreased stroke risk (RR, 0.84; 95% CI, 0.77−0.93; P=0.0005) in the pooled analysis of large RCTs (RR, 0.85; 95% CI, 0.77−0.93; P=0.0008) versus placebo. In small RCTs, use of GLP1-RAs did not decrease stroke risk versus placebo (RR, 0.72; 95% CI, 0.36−1.42; P=0.34) or AHGs (RR, 0.85; 95% CI, 0.48−1.49; P=0.56) using fixed- and random-effect models (Figure S17 and Figure S18). The GRADE scores were moderate (Table S14).

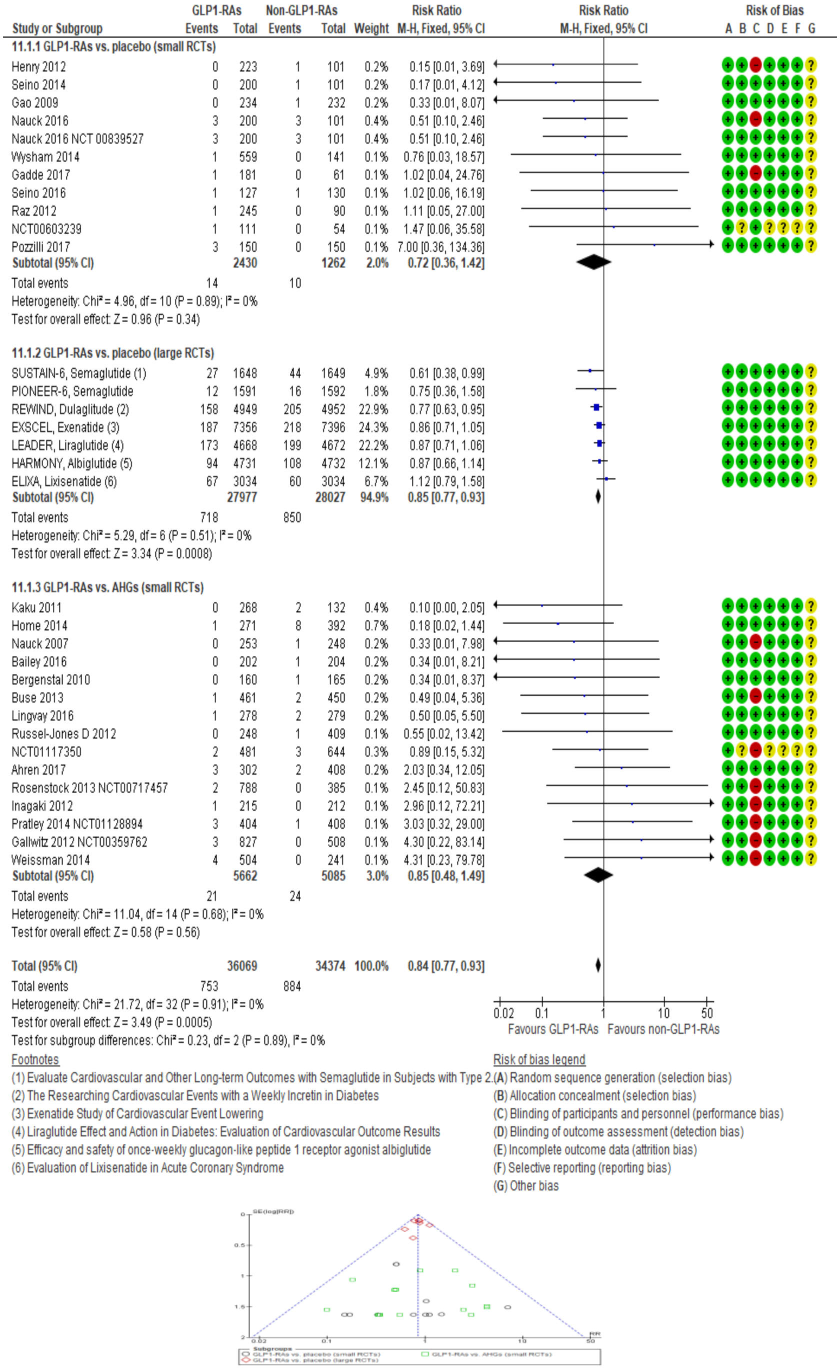

Figure S17. Forest and funnel plot of GLP1-RAs and stroke by clinical trial size, Fixed-effect model.

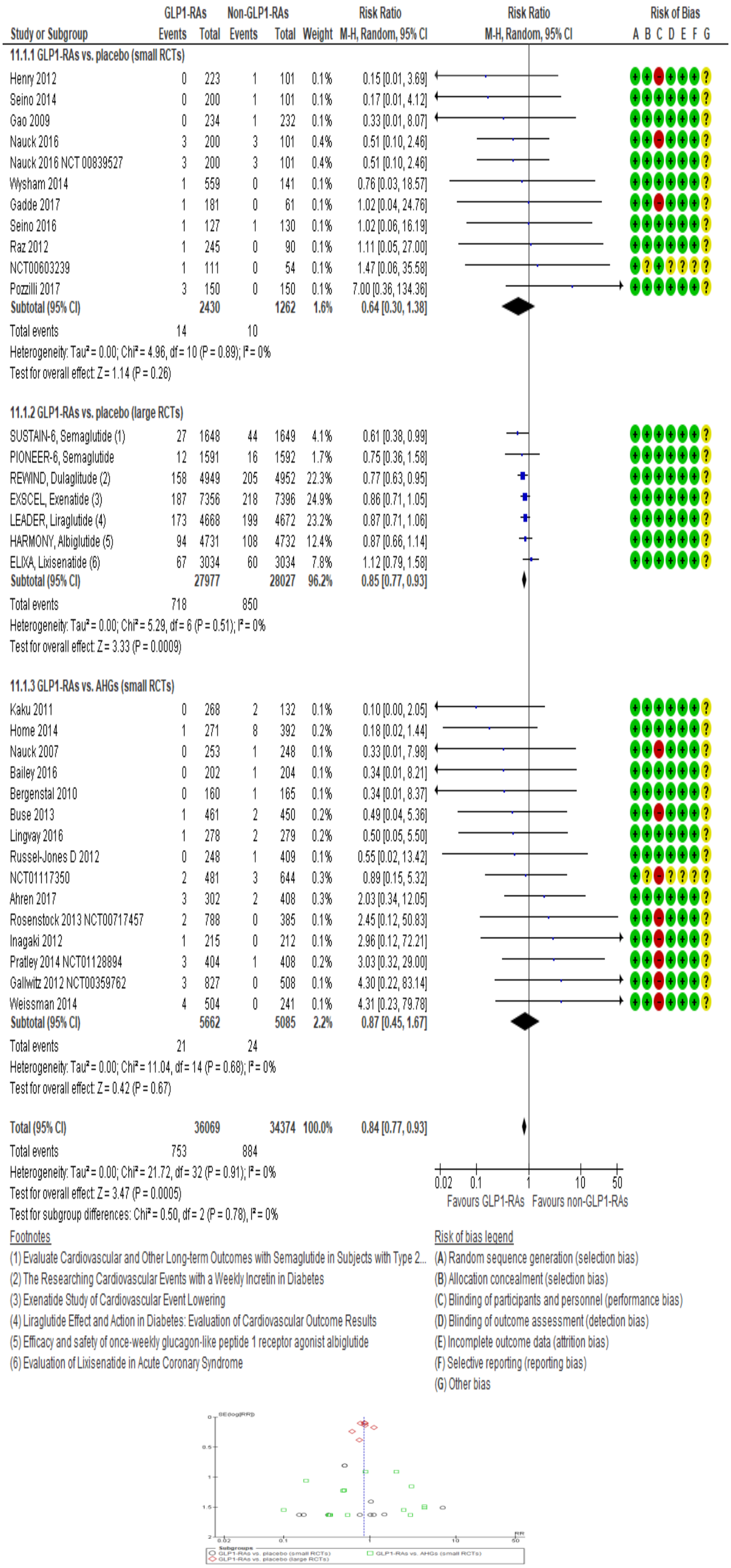

Figure S18. Forest and funnel plot of GLP1-RAs and stroke by clinical trial size, Random-effect model.

Table S14. GRADE evidence profile of GLP1-RAs and stroke by clinical trial size

Certainty assessment							Summary of findings				
№ of participants (trials)	Risk of bias	Inconsistency	Indirectness	Imprecision	Publication bias	Overall certainty of evidence	Study event rates (%)		Relative effect (95% CI)	Anticipated absolute effects	
							With placebo / controls	With GLP1-RAs		Risk with placebo / controls	Risk difference with GLP1-RAs
**Risk of stroke risk by clinical trial size (follow-up: range 16-339 weeks)**											
70443 (33 RCTs)	serious[a]	not serious	not serious	not serious	none	⊕⊕⊕◯ MODERATE	884/34374 (2.6%)	753/36069 (2.1%)	RR 0.84 (0.77 to 0.93)	3 per 100	0 fewer per 100 (from 1 fewer to 0 fewer)
**Small RCTs with GLP1-RAs vs. placebo (follow-up: range 16-156 weeks)**											
3692 (11 RCTs)	serious[a]	not serious	not serious	very serious[b]	none	⊕◯◯◯ VERY LOW	10/1262 (0.8%)	14/2430 (0.6%)	RR 0.72 (0.36 to 1.42)	1 per 100	0 fewer per 100 (from 1 fewer to 0 fewer)
**Large RCTs with GLP1-RAs vs. placebo (follow-up: range 84-339 weeks)**											
56004 (7 RCTs)	not serious	not serious	not serious	not serious	none	⊕⊕⊕⊕ HIGH	850/28027 (3.0%)	718/27977 (2.6%)	RR 0.85 (0.77 to 0.93)	3 per 100	0 fewer per 100 (from 1 fewer to 0 fewer)
**Small RCTs with GLP1-RAs vs. AHGs (follow-up: range 26-235 weeks)**											
10747 (15 RCTs)	serious[a]	not serious	not serious	very serious[c]	none	⊕◯◯◯ VERY LOW	24/5085 (0.5%)	21/5662 (0.4%)	RR 0.85 (0.48 to 1.49)	0 per 100	0 fewer per 100 (from 0 fewer to 0 fewer)

Abbreviations: **AHGs,** anti-hyperglycaemic agents; **CI,** confidence interval; **GLP1-RAs,** glucagon-like peptide-1 receptor agonists; **RCTs,** randomised controlled trials; **RR,** risk ratio.

Note: The GRADE scores were from the fixed-effect model.

GRADE evidence

a. Eleven small RCTs with small weight GLP1-RAs vs. placebo (0.4%, 0.2%, and 0.1%) and GLP1-RAs vs. AHGs (0.3%, 0.2%, 0.2%, 0.1%, 0.1%, 0.1%, 0.1%, and 0.1%) with the overall EE rated as high risk of bias due to lack of blinding (open-label design) out of 33 trials, one of which was an unpublished RCT.

b. The overall imprecision was precise, with no significant effect size difference (P=0.34). However, all trials reported overlapping CIs, in which six trials reported wide CIs. The 95% CI was consistent with the possibility of a benefit not exceeding the MID, including only 24 events with a large sample size.

c. The overall imprecision was precise, with no significant effect size difference (P=0.56). However, all trials reported overlapping CIs, in which seven trials reported wide CIs. The 95% CI was consistent with the possibility of a benefit not exceeding the MID, including only 45 events with a large sample size.

In summary, the overall certainty of the pooled EE had moderate imprecision with a significant effect size difference in the level of evidence (P=0.0005). However, the majority of trials reported overlapping CIs, in which 13 trials reported wide CIs. In total, only one large (22.9%) and one small (4.9%) trials did not cross the line of no difference (1), in which a significant reduction of stroke in the direction of GLP1-RAs favoured placebo in large RCTs. The 95% CI was consistent with the possibility of a large benefit exceeding the MID, including a total of 1,637 events with a large sample size. There was no evidence of inconsistency, statistically significant heterogeneity (P=0.91; I^2=0%), or subgroup difference (P=0.89; I^2=0%). The majority of trials had low risk of bias (98.1%). There was no evidence of detection bias. However, the magnitude of the impact of some trials that suffered from limitations likely resulted in biased assessment of the intervention effect due to performance and selection bias (11 trials with open-label design out of 33, one of which was an unpublished RCT). The indirectness of the trials indicated a high level of evidence, with an individual trial PICOS element aligned closely to the review PICOS. Finally, there was no evidence of reporting bias; all included trials were within the two boundaries of the pyramid.

DPP4-Is and stroke by clinical trial size

Use of DPP4-Is did not decrease stroke risk in the pooled analysis of small RCTs (RR, 0.71; 95% CI, 0.49−1.02; P=0.07) or large RCTs (RR, 0.97; 95% CI, 0.86−1.10; P=0.62) versus placebo, or in small RCTs (RR, 0.84; 95% CI, 0.61−1.15; P=0.27) or large RCTs (RR, 0.84; 95% CI, 0.66−1.08; P=0.17) versus AHGs using fixed- and random-effect models (Figure S19 and Figure S20). The GRADE scores were moderate (Table S15).

Study or Subgroup	DPP4-Is Events	DPP4-Is Total	Non-DPP4-Is Events	Non-DPP4-Is Total	Weight	Risk Ratio M-H, Fixed, 95% CI
**10.1.1 DPP4-Is vs. placebo (small RCTs)**						
Frederich 2012	0	291	1	74	0.3%	0.09 [0.00, 2.08]
Pattzi 2010	0	336	1	86	0.3%	0.09 [0.00, 2.09]
Barnett 2012 NCT00757588	0	304	2	151	0.4%	0.10 [0.00, 2.06]
Garcia-Soria 2008	0	133	1	41	0.3%	0.10 [0.00, 2.52]
Taskinen 2011	0	523	1	177	0.3%	0.11 [0.00, 2.77]
Wang 2016	0	205	1	100	0.3%	0.16 [0.01, 3.98]
Pan 2012 NCT00822211	0	294	1	144	0.3%	0.16 [0.01, 4.00]
Rosenstock 2009	0	260	1	129	0.3%	0.17 [0.01, 4.05]
Ba 2017	0	248	2	249	0.3%	0.20 [0.01, 4.16]
McMurray 2013	1	128	4	126	0.5%	0.25 [0.03, 2.17]
Chacra 2011	1	501	2	267	0.3%	0.27 [0.02, 2.93]
Williams-Herman 2010	1	668	3	540	0.4%	0.27 [0.03, 2.58]
Yang 2015	0	143	1	135	0.2%	0.31 [0.01, 7.66]
1218.43a 2014	1	68	3	65	0.4%	0.32 [0.03, 2.99]
Scherbaum 2008	0	156	1	150	0.2%	0.32 [0.01, 7.81]
Seino 2011	1	155	1	75	0.2%	0.48 [0.03, 7.63]
Bosi 2007	1	360	1	181	0.2%	0.50 [0.03, 7.99]
Aschner 2006	1	488	1	253	0.2%	0.52 [0.03, 8.25]
McGill 2013	1	68	1	65	0.1%	0.96 [0.06, 14.97]
Groop 2017	1	182	1	178	0.1%	0.98 [0.06, 15.52]
Shankar 2017	1	234	1	233	0.1%	1.00 [0.06, 15.82]
Nowicki 2011	1	85	1	85	0.1%	1.00 [0.06, 15.73]
Dobs 2013	1	170	2	341	0.2%	1.00 [0.09, 10.98]
Yang 2011	1	283	1	287	0.1%	1.01 [0.06, 16.13]
Dejager 2007	1	472	0	160	0.1%	1.02 [0.04, 24.94]
Ji 2016	1	367	1	377	0.1%	1.03 [0.06, 16.36]
Yki-Järvinen 2013	5	631	4	630	0.5%	1.25 [0.34, 4.63]
Rosenstock 20013	2	372	0	95	0.1%	1.29 [0.06, 26.58]
Barnett 2013	1	162	0	79	0.1%	1.47 [0.06, 35.74]
Seino 2012	1	209	0	103	0.1%	1.49 [0.06, 36.16]
Mohan 2009	1	352	0	178	0.1%	1.52 [0.06, 37.15]
Hollander 2011	4	381	1	184	0.2%	1.93 [0.22, 17.16]
HU 2015	2	223	1	223	0.1%	2.00 [0.18, 21.90]
Yang 2012	2	197	1	198	0.1%	2.01 [0.18, 21.99]
Roden 2013 NCT01177813	2	223	1	229	0.1%	2.05 [0.19, 22.49]
Pan 2012	1	284	0	284	0.1%	3.00 [0.12, 73.33]
Pan 2017	1	252	0	253	0.1%	3.01 [0.12, 73.58]
Hage 2014	1	34	0	37	0.1%	3.26 [0.14, 77.35]
DeFronzo 2013	5	564	0	179	0.1%	3.50 [0.19, 63.07]
Wang 2017	2	191	0	189	0.1%	4.95 [0.24, 102.38]
**Subtotal (95% CI)**		**11197**		**7530**	**8.0%**	**0.71 [0.49, 1.02]**
Total events	45		44			
Heterogeneity: Chi² = 24.32, df = 39 (P = 0.97); I² = 0%						
Test for overall effect: Z = 1.84 (P = 0.07)						
**10.1.2 DPP4-Is vs. placebo (large RCTs)**						
EXAMINE, Alogliptin (1)	29	2701	32	2679	4.0%	0.90 [0.55, 1.48]
CARMELINA, Linagliptin (2)	81	3494	88	3485	11.1%	0.92 [0.68, 1.24]
OMNEON, omarigliptin (3)	32	2092	34	2100	4.3%	0.94 [0.59, 1.53]
TECOS, Sitagliptin (4)	178	7257	183	7266	23.0%	0.97 [0.79, 1.19]
SAVOR-TIMI 53, Saxagliptin (5)	179	8280	176	8212	22.2%	1.01 [0.82, 1.24]
**Subtotal (95% CI)**		**23824**		**23742**	**64.7%**	**0.97 [0.86, 1.10]**
Total events	499		513			
Heterogeneity: Chi² = 0.37, df = 4 (P = 0.98); I² = 0%						
Test for overall effect: Z = 0.50 (P = 0.62)						
**10.1.3 DPP4-Is vs. AHGs (small RCTs)**						
Rosenstock 2010	0	491	1	163	0.3%	0.11 [0.00, 2.71]
Arechavaleta 2011	0	516	3	518	0.4%	0.14 [0.01, 2.77]
Barnett 2012	0	151	1	76	0.3%	0.17 [0.01, 4.10]
NCT01318070	0	224	1	115	0.2%	0.17 [0.01, 4.19]
Yoon 2011	0	164	2	153	0.3%	0.19 [0.01, 3.86]
Goldenberg 2017	0	322	2	320	0.3%	0.20 [0.01, 4.12]
Schernthaner 2015	0	359	2	359	0.3%	0.20 [0.01, 4.15]
Mita 2016	0	161	2	161	0.3%	0.20 [0.01, 4.13]
Olansky 2011	1	625	4	621	0.5%	0.25 [0.03, 2.22]
Ahren 2014	0	302	2	408	0.3%	0.27 [0.01, 5.60]
Gallwitz 2012	3	776	11	775	1.4%	0.27 [0.08, 0.97]
Yoon 2012	0	164	1	153	0.2%	0.31 [0.01, 7.58]
Scheen 2010	0	403	1	398	0.2%	0.33 [0.01, 8.06]
Seck 2010	0	588	1	584	0.2%	0.33 [0.01, 8.11]
NCT01131182	0	507	1	514	0.2%	0.34 [0.01, 8.28]
Mu 2017	1	713	1	289	0.2%	0.41 [0.03, 6.46]
Haak 2013	1	396	1	170	0.2%	0.43 [0.03, 6.82]
Laakso 2015	1	113	2	122	0.2%	0.54 [0.05, 5.87]
Bolli 2009	2	295	3	280	0.4%	0.63 [0.11, 3.76]
Chan 2008	2	65	1	26	0.2%	0.80 [0.08, 8.45]
NCT01204294	3	450	1	124	0.2%	0.83 [0.09, 7.88]
Russel-Jones D 2012 NCT00676338	0	163	1	409	0.1%	0.83 [0.03, 20.35]
Hermansen 2007	1	222	1	219	0.1%	0.99 [0.06, 15.67]
NCT00707993	1	222	1	219	0.1%	0.99 [0.06, 15.67]
Bosi 2011	1	404	1	399	0.1%	0.99 [0.06, 15.74]
Bergenstal 2010 NCT00637273	1	166	1	165	0.1%	0.99 [0.06, 15.76]
DeFronzo 2012	2	1037	1	516	0.2%	1.00 [0.09, 10.95]
Göke 2013	4	428	4	430	0.5%	1.00 [0.25, 3.99]
NCT00722371	3	922	2	693	0.3%	1.13 [0.19, 6.73]
Ferreira 2013	5	210	4	212	0.5%	1.26 [0.34, 4.63]
Jadzinsky 2009	4	978	1	328	0.2%	1.34 [0.15, 11.96]
Schweizer 2007	1	511	0	249	0.1%	1.46 [0.06, 35.83]
Seino 2011 NCT01263470	1	322	0	158	0.1%	1.48 [0.06, 36.05]
NCT01263496	3	391	0	83	0.1%	1.50 [0.08, 28.77]
Kawamori 2012	4	319	2	242	0.3%	1.52 [0.28, 8.22]
Charbonnel 2006	3	464	1	237	0.2%	1.53 [0.16, 14.65]
Philis-Tsimikas 2013	2	228	1	226	0.1%	1.98 [0.18, 21.71]
NCT01519674	2	383	0	192	0.1%	2.51 [0.12, 52.09]
Terauchi 2017	1	148	0	143	0.1%	2.90 [0.12, 70.59]
Raz 2006	5	411	0	110	0.1%	2.96 [0.17, 53.19]
Zang 2016	1	184	0	183	0.1%	2.98 [0.12, 72.77]
NCT01076075	1	210	0	212	0.1%	3.03 [0.12, 73.92]
Leiter 2014	6	246	2	249	0.3%	3.04 [0.62, 14.90]
Rosenstock 2009 NCT00099918	3	393	0	198	0.1%	3.54 [0.18, 68.11]
Pratley 2014	2	442	0	326	0.1%	3.69 [0.18, 76.62]
Wainstein 2012	2	261	0	256	0.1%	4.90 [0.24, 101.66]
Foley 2009	3	546	0	546	0.1%	7.00 [0.36, 135.20]
**Subtotal (95% CI)**		**17996**		**13529**	**10.8%**	**0.84 [0.61, 1.15]**
Total events	76		67			
Heterogeneity: Chi² = 28.22, df = 46 (P = 0.98); I² = 0%						
Test for overall effect: Z = 1.11 (P = 0.27)						
**10.1.4 DPP4-Is vs. AHGs (large RCTs)**						
Ferrannini 2009	0	1389	7	1383	0.9%	0.07 [0.00, 1.16]
CAROLINA, Linagliptin (6)	104	3023	120	3010	15.1%	0.86 [0.67, 1.12]
Del Prato 2014	10	1751	3	869	0.5%	1.65 [0.46, 6.00]
**Subtotal (95% CI)**		**6163**		**5262**	**16.6%**	**0.84 [0.66, 1.08]**
Total events	114		130			
Heterogeneity: Chi² = 4.12, df = 2 (P = 0.13); I² = 51%						
Test for overall effect: Z = 1.36 (P = 0.17)						
**Total (95% CI)**		**59180**		**50063**	**100.0%**	**0.91 [0.83, 1.01]**
Total events	734		754			
Heterogeneity: Chi² = 59.68, df = 94 (P = 1.00); I² = 0%						
Test for overall effect: Z = 1.79 (P = 0.07)						
Test for subgroup differences: Chi² = 3.48, df = 3 (P = 0.32), I² = 13.9%						

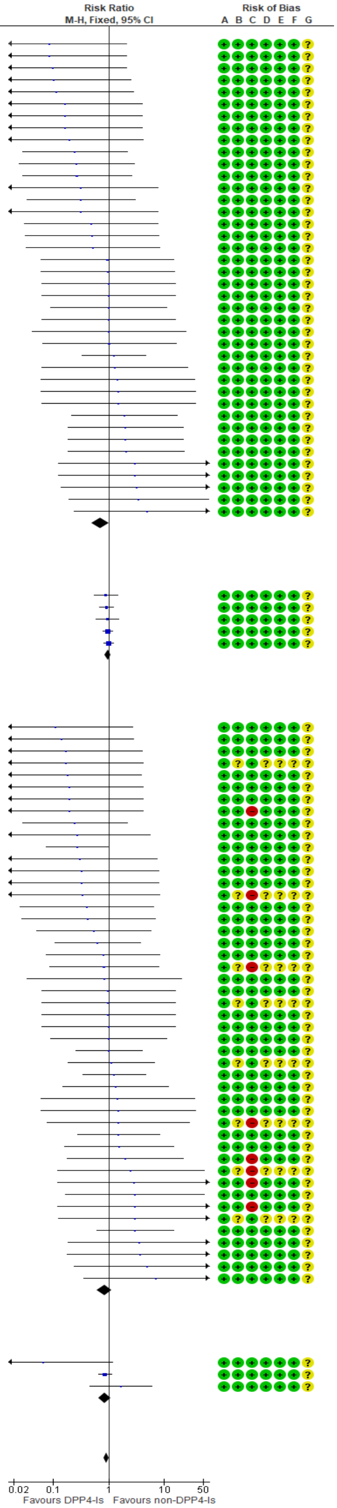

Footnotes
(1) Examination of Cardiovascular Outcomes with Alogliptin versus Standard of Care
(2) The Cardiovascular and Renal Microvascular Outcome Study with Linagliptin
(3) A Study to Assess Cardiovascular Outcomes Following Treatment with Omarigliptin [MK-3102] in...
(4) Trial Evaluating Cardiovascular Outcomes with Sitagliptin
(5) Saxagliptin Assessment of Vascular Outcomes Recorded in Patients with Diabetes Mellitus...
(6) The Cardiovascular Outcome Study of Linagliptin vs Glimepiride in Type 2 Diabetes

Risk of bias legend
(A) Random sequence generation (selection bias)
(B) Allocation concealment (selection bias)
(C) Blinding of participants and personnel (performance bias)
(D) Blinding of outcome assessment (detection bias)
(E) Incomplete outcome data (attrition bias)
(F) Selective reporting (reporting bias)
(G) Other bias

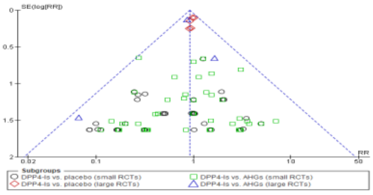

Figure S19. Forest and funnel plot of DPP4-Is and stroke by clinical trial size, Fixed-effect model.

Study or Subgroup	DPP4-Is Events	DPP4-Is Total	Non-DPP4-Is Events	Non-DPP4-Is Total	Weight	Risk Ratio M-H, Random, 95% CI
**10.1.1 DPP4-Is vs. placebo (small RCTs)**						
Frederich 2012	0	291	1	74	0.1%	0.09 [0.00, 2.08]
Pattzi 2010	0	336	1	86	0.1%	0.09 [0.00, 2.09]
Barnett 2012 NCT00757588	0	304	2	151	0.1%	0.10 [0.00, 2.06]
Garcia-Soria 2008	0	133	1	41	0.1%	0.10 [0.00, 2.52]
Taskinen 2011	0	523	1	177	0.1%	0.11 [0.00, 2.77]
Wang 2016	0	205	1	100	0.1%	0.16 [0.01, 3.98]
Pan 2012 NCT00822211	0	294	1	144	0.1%	0.16 [0.01, 4.00]
Rosenstock 2009	0	260	1	129	0.1%	0.17 [0.01, 4.05]
Ba 2017	0	248	2	249	0.1%	0.20 [0.01, 4.16]
McMurray 2013	1	128	4	126	0.2%	0.25 [0.03, 2.17]
Chacra 2011	1	501	2	267	0.2%	0.27 [0.02, 2.93]
Williams-Herman 2010	1	668	3	540	0.2%	0.27 [0.03, 2.58]
Yang 2015	0	143	1	135	0.1%	0.31 [0.01, 7.66]
1218.43a 2014	1	68	3	65	0.2%	0.32 [0.03, 2.99]
Scherbaum 2008	0	156	1	150	0.1%	0.32 [0.01, 7.81]
Seino 2011	1	155	1	75	0.1%	0.48 [0.03, 7.63]
Bosi 2007	1	360	1	181	0.1%	0.50 [0.03, 7.99]
Aschner 2006	1	488	1	253	0.1%	0.52 [0.03, 8.25]
McGill 2013	1	68	1	65	0.1%	0.96 [0.06, 14.97]
Groop 2017	1	182	1	178	0.1%	0.98 [0.06, 15.52]
Shankar 2017	1	234	1	233	0.1%	1.00 [0.06, 15.82]
Nowicki 2011	1	85	1	85	0.1%	1.00 [0.06, 15.73]
Dobs 2013	1	170	2	341	0.2%	1.00 [0.09, 10.98]
Yang 2011	1	283	1	287	0.1%	1.01 [0.06, 16.13]
Dejager 2007	1	472	0	160	0.1%	1.02 [0.04, 24.94]
Ji 2016	1	367	1	377	0.1%	1.03 [0.06, 16.36]
Yki-Järvinen 2013	5	631	4	630	0.6%	1.25 [0.34, 4.63]
Rosenstock 20013	2	372	0	95	0.1%	1.29 [0.06, 26.58]
Barnett 2013	1	162	0	79	0.1%	1.47 [0.06, 35.74]
Seino 2012	1	209	0	103	0.1%	1.49 [0.06, 36.16]
Mohan 2009	1	352	0	178	0.1%	1.52 [0.06, 37.15]
Hollander 2011	4	381	1	184	0.2%	1.93 [0.22, 17.16]
HU 2015	2	223	1	223	0.2%	2.00 [0.18, 21.90]
Yang 2012	2	197	1	198	0.2%	2.01 [0.18, 21.99]
Roden 2013 NCT01177813	2	223	1	229	0.2%	2.05 [0.19, 22.49]
Pan 2012	1	284	0	284	0.1%	3.00 [0.12, 73.33]
Pan 2017	1	252	0	253	0.1%	3.01 [0.12, 73.58]
Hage 2014	1	34	0	37	0.1%	3.26 [0.14, 77.35]
DeFronzo 2013	5	564	0	179	0.1%	3.50 [0.19, 63.07]
Wang 2017	2	191	0	189	0.1%	4.95 [0.24, 102.38]
**Subtotal (95% CI)**		**11197**		**7530**	**5.8%**	**0.71 [0.46, 1.08]**
Total events	45		44			
Heterogeneity: Tau² = 0.00; Chi² = 24.32, df = 39 (P = 0.97); I² = 0%						
Test for overall effect: Z = 1.61 (P = 0.11)						
**10.1.2 DPP4-Is vs. placebo (large RCTs)**						
EXAMINE, Alogliptin (1)	29	2701	32	2679	4.1%	0.90 [0.55, 1.48]
CARMELINA, Linagliptin (2)	81	3494	88	3485	11.6%	0.92 [0.68, 1.24]
OMNEON, omarigliptin (3)	32	2092	34	2100	4.5%	0.94 [0.59, 1.53]
TECOS, Sitagliptin (4)	178	7257	183	7266	24.9%	0.97 [0.79, 1.19]
SAVOR-TIMI 53, Saxagliptin (5)	179	8280	176	8212	24.4%	1.01 [0.82, 1.24]
**Subtotal (95% CI)**		**23824**		**23742**	**69.5%**	**0.97 [0.86, 1.10]**
Total events	499		513			
Heterogeneity: Tau² = 0.00; Chi² = 0.37, df = 4 (P = 0.98); I² = 0%						
Test for overall effect: Z = 0.49 (P = 0.62)						
**10.1.3 DPP4-Is vs. AHGs (small RCTs)**						
Rosenstock 2010	0	491	1	163	0.1%	0.11 [0.00, 2.71]
Arechavaleta 2011	0	516	3	518	0.1%	0.14 [0.01, 2.77]
Barnett 2012	0	151	1	76	0.1%	0.17 [0.01, 4.10]
NCT01318070	0	224	1	115	0.1%	0.17 [0.01, 4.19]
Yoon 2011	0	164	2	153	0.1%	0.19 [0.01, 3.86]
Goldenberg 2017	0	322	2	320	0.1%	0.20 [0.01, 4.12]
Schernthaner 2015	0	359	2	359	0.1%	0.20 [0.01, 4.15]
Mita 2016	0	161	2	161	0.1%	0.20 [0.01, 4.13]
Olansky 2011	1	625	4	621	0.2%	0.25 [0.03, 2.22]
Ahren 2014	0	302	2	408	0.1%	0.27 [0.01, 5.60]
Gallwitz 2012	3	776	11	775	0.6%	0.27 [0.08, 0.97]
Yoon 2012	0	164	1	153	0.1%	0.31 [0.01, 7.58]
Scheen 2010	0	403	1	398	0.1%	0.33 [0.01, 8.06]
Seck 2010	0	588	1	584	0.1%	0.33 [0.01, 8.11]
NCT01131182	0	507	1	514	0.1%	0.34 [0.01, 8.28]
Mu 2017	1	713	1	289	0.1%	0.41 [0.03, 6.46]
Haak 2013	1	396	1	170	0.1%	0.43 [0.03, 6.82]
Laakso 2015	1	113	2	122	0.2%	0.54 [0.05, 5.87]
Bolli 2009	2	295	3	280	0.3%	0.63 [0.11, 3.76]
Chan 2008	2	65	1	26	0.2%	0.80 [0.08, 8.45]
NCT01204294	3	450	1	124	0.2%	0.83 [0.09, 7.88]
Russel-Jones D 2012 NCT00676338	0	163	1	409	0.1%	0.83 [0.03, 20.35]
Hermansen 2007	1	222	1	219	0.1%	0.99 [0.06, 15.67]
NCT00707993	1	222	1	219	0.1%	0.99 [0.06, 15.67]
Bosi 2011	1	404	1	399	0.1%	0.99 [0.06, 15.74]
Bergenstal 2010 NCT00637273	1	166	1	165	0.1%	0.99 [0.06, 15.76]
DeFronzo 2012	2	1037	1	516	0.2%	1.00 [0.09, 10.95]
Göke 2013	4	428	4	430	0.5%	1.00 [0.25, 3.99]
NCT00722371	3	922	2	693	0.3%	1.13 [0.19, 6.73]
Ferreira 2013	5	210	4	212	0.6%	1.26 [0.34, 4.63]
Jadzinsky 2009	4	978	1	328	0.2%	1.34 [0.15, 11.96]
Schweizer 2007	1	511	0	249	0.1%	1.46 [0.06, 35.83]
Seino 2011 NCT01263470	1	322	0	158	0.1%	1.48 [0.06, 36.05]
NCT01263496	3	391	0	83	0.1%	1.50 [0.08, 28.77]
Kawamori 2012	4	319	2	242	0.4%	1.52 [0.28, 8.22]
Charbonnel 2006	3	464	1	237	0.2%	1.53 [0.16, 14.65]
Philis-Tsimikas 2013	2	228	1	226	0.2%	1.98 [0.18, 21.71]
NCT01519674	2	383	0	192	0.1%	2.51 [0.12, 52.09]
Terauchi 2017	1	148	0	143	0.1%	2.90 [0.12, 70.59]
Raz 2006	5	411	0	110	0.1%	2.96 [0.17, 53.19]
Zang 2016	1	184	0	183	0.1%	2.98 [0.12, 72.77]
NCT01076075	1	210	0	212	0.1%	3.03 [0.12, 73.92]
Leiter 2014	6	246	2	249	0.4%	3.04 [0.62, 14.90]
Rosenstock 2009 NCT00099918	3	393	0	198	0.1%	3.54 [0.18, 68.11]
Pratley 2014	2	442	0	326	0.1%	3.69 [0.18, 76.62]
Wainstein 2012	2	261	0	256	0.1%	4.90 [0.24, 101.66]
Foley 2009	3	546	0	546	0.1%	7.00 [0.36, 135.20]
**Subtotal (95% CI)**		**17996**		**13529**	**8.4%**	**0.85 [0.60, 1.20]**
Total events	76		67			
Heterogeneity: Tau² = 0.00; Chi² = 28.22, df = 46 (P = 0.98); I² = 0%						
Test for overall effect: Z = 0.94 (P = 0.35)						
**10.1.4 DPP4-Is vs. AHGs (large RCTs)**						
Ferrannini 2009	0	1389	7	1383	0.1%	0.07 [0.00, 1.16]
CAROLINA, Linagliptin (6)	104	3023	120	3010	15.6%	0.86 [0.67, 1.12]
Del Prato 2014	10	1751	3	869	0.6%	1.65 [0.46, 6.00]
**Subtotal (95% CI)**		**6163**		**5262**	**16.3%**	**0.82 [0.31, 2.17]**
Total events	114		130			
Heterogeneity: Tau² = 0.40; Chi² = 4.12, df = 2 (P = 0.13); I² = 51%						
Test for overall effect: Z = 0.41 (P = 0.68)						
**Total (95% CI)**		**59180**		**50063**	**100.0%**	**0.92 [0.83, 1.02]**
Total events	734		754			
Heterogeneity: Tau² = 0.00; Chi² = 59.68, df = 94 (P = 1.00); I² = 0%						
Test for overall effect: Z = 1.52 (P = 0.13)						
Test for subgroup differences: Chi² = 2.44, df = 3 (P = 0.49), I² = 0%						

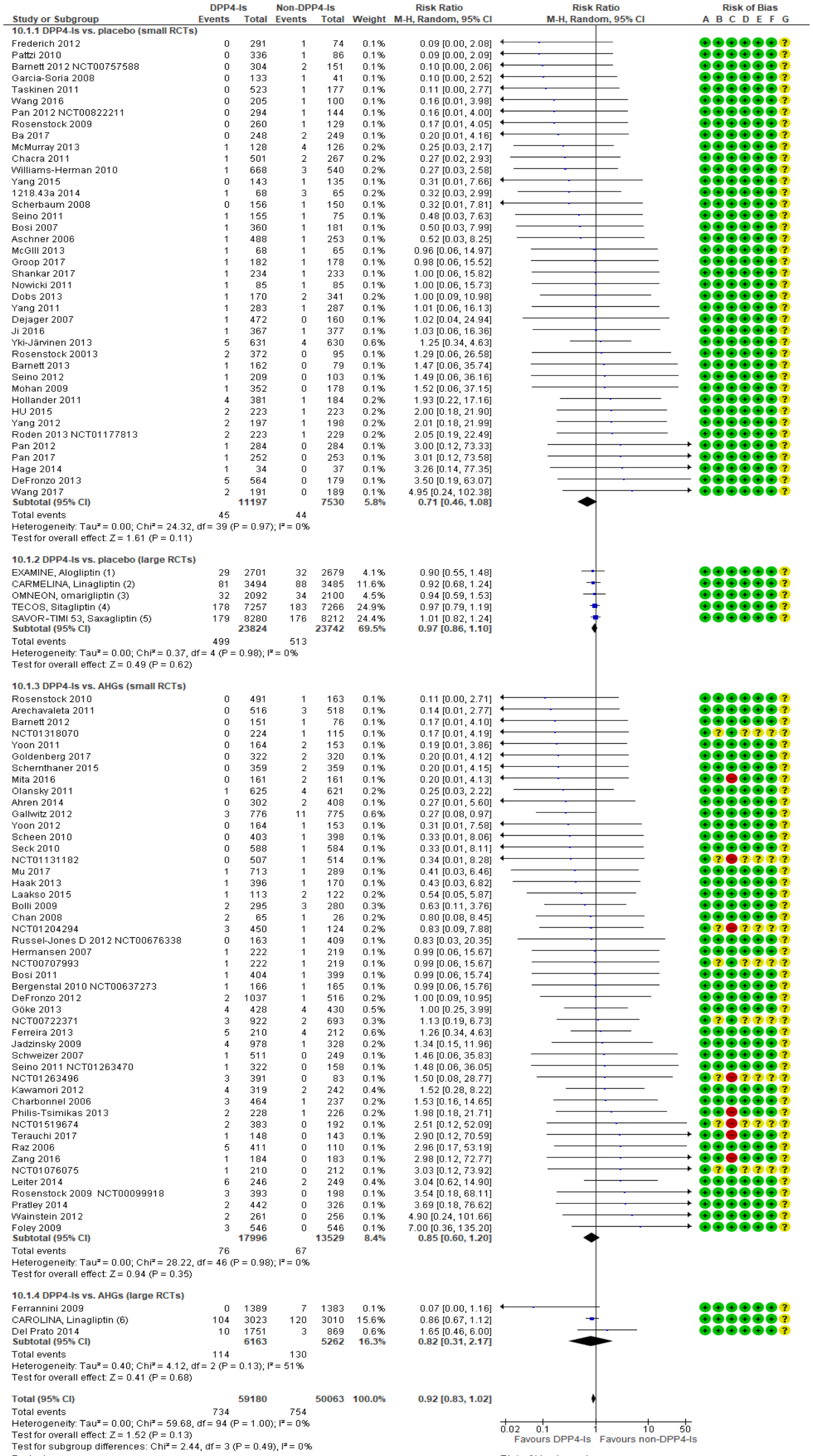

Footnotes
(1) Examination of Cardiovascular Outcomes with Alogliptin versus Standard of Care
(2) The Cardiovascular and Renal Microvascular Outcome Study with Linagliptin
(3) A Study to Assess Cardiovascular Outcomes Following Treatment with Omarigliptin [MK-3102] in...
(4) Trial Evaluating Cardiovascular Outcomes with Sitagliptin
(5) Saxagliptin Assessment of Vascular Outcomes Recorded in Patients with Diabetes Mellitus...
(6) The Cardiovascular Outcome Study of Linagliptin vs Glimepiride in Type 2 Diabetes

Risk of bias legend
(A) Random sequence generation (selection bias)
(B) Allocation concealment (selection bias)
(C) Blinding of participants and personnel (performance bias)
(D) Blinding of outcome assessment (detection bias)
(E) Incomplete outcome data (attrition bias)
(F) Selective reporting (reporting bias)
(G) Other bias

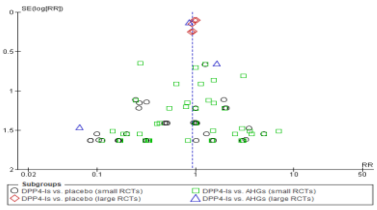

Figure S20. Forest and funnel plot of DPP4-Is and stroke by clinical trial size, Random-effect model.

Table S15. GRADE evidence profile of DPP4-Is and stroke by clinical trial size

Certainty assessment							Summary of findings				
№ of participants (trials)	Risk of bias	Inconsistency	Indirectness	Imprecision	Publication bias	Overall certainty of evidence	Study event rates (%)		Relative effect (95% CI)	Anticipated absolute effects	
							With placebo / controls	With DPP4-Is		Risk with placebo / controls	Risk difference with DPP4-Is
**Risk of stroke risk by clinical trial size (follow-up: range 4-432 weeks)**											
109243 (95 RCTs)	serious[a]	not serious	not serious	not serious	none	⨁⨁⨁◯ MODERATE	754/50063 (1.5%)	734/59180 (1.2%)	RR 0.91 (0.83 to 1.01)	1 per 100	0 fewer per 100 (from 0 fewer to 0 fewer)
**Small RCTs with DPP4-Is vs. placebo (follow-up: range 4-206 weeks)**											
18727 (40 RCTs)	not serious	not serious	not serious	very serious[b]	none	⨁⨁◯◯ LOW	44/7530 (0.6%)	45/11197 (0.4%)	RR 0.71 (0.49 to 1.02)	1 per 100	0 fewer per 100 (from 0 fewer to 0 fewer)
**Large RCTs with DPP4-Is vs. placebo (follow-up: range 78-224 weeks)**											
47566 (5 RCTs)	not serious	not serious	not serious	not serious	none	⨁⨁⨁⨁ HIGH	513/23742 (2.2%)	499/23824 (2.1%)	RR 0.97 (0.86 to 1.10)	2 per 100	0 fewer per 100 (from 0 fewer to 0 fewer)
**Small RCTs with DPP4-Is vs. AHGs (follow-up: range 4-104 weeks)**											
31525 (47 RCTs)	serious[a]	not serious	not serious	very serious[c]	none	⨁◯◯◯ VERY LOW	67/13529 (0.5%)	76/17996 (0.4%)	RR 0.84 (0.61 to 1.15)	0 per 100	0 fewer per 100 (from 0 fewer to 0 fewer)
**Large RCTs with DPP4-Is vs. AHGs (follow-up: range 24-432 weeks)**											
11425 (3 RCTs)	not serious	not serious	not serious	very serious[d]	none	⨁⨁◯◯ LOW	130/5262 (2.5%)	114/6163 (1.8%)	RR 0.84 (0.66 to 1.08)	2 per 100	0 fewer per 100 (from 1 fewer to 0 fewer)

Abbreviations: **AHGs,** anti-hyperglycaemic agents; **CI,** confidence interval; **DPP4-Is,** dipeptidyl peptidase-4 inhibitors; **RCTs,** randomised controlled trials; **RR,** risk ratio.

Note: The GRADE scores were from the fixed-effect model.

GRADE evidence

a. Eight trials with small weight DPP4-Is vs. AHGs (small RCTs [0.3%, 0.2%, 0.2%, 0.1%, 0.1%, 0.1%, 0.1%, and 0.1%]) with the overall EE rated as high risk of bias due to lack of blinding (open-label design) out of 95 trials, four of which were unpublished RCTs.

b. The overall imprecision was precise, with no significant effect size difference (P=0.07). However, all trials reported overlapping CIs, in which 20 trials reported wide CIs. The 95% CI was consistent with the possibility of a benefit not exceeding the MID, including only 89 events with a large sample size.

c. The overall imprecision was precise, with no significant effect size difference (P=0.27). However, all trials reported overlapping CIs, in which 21 trials reported wide CIs. The 95% CI was consistent with the possibility of a benefit not exceeding the MID, including only 143 events with a large sample size.

d. The overall imprecision was precise, with no significant effect size difference (P=0.17). However, all trials reported overlapping and narrow CIs. The 95% CI was consistent with the possibility of a benefit not exceeding the MID, including only 244 events with a large sample size.

In summary, the overall certainty of the pooled EE had moderate imprecision without a significant effect size difference in the level of evidence (P=0.07). However, the majority of trials reported overlapping CIs, in which 41 trials reported wide CIs. In total, only one small trial (1.4%) did not cross the line of no difference (1), in which there was a significant reduction in stroke in the direction of DPP4-Is favouring active comparators of AHGs in small RCTs. The 95% CI was consistent with the possibility of a large benefit exceeding the MID, including a total of 1,488 events with a large sample size (109,243). There was no evidence of inconsistency, statistically significant heterogeneity (P=1.00; I^2=0%), or subgroup difference (P=0.32; I^2=13.9%). The majority of trials had low risk of bias (98.8%). There was no evidence

of detection bias. However, the magnitude of the impact of some trials that suffered from limitations likely resulted in biased assessment of the intervention effect due to performance and selection bias (eight trials with open-label design out of 95). The indirectness of the trials indicated a high level of evidence, with an individual trial PICOS element aligned closely to the review PICOS. Finally, there was no evidence of reporting bias; all included trials were within the two boundaries of the pyramid.

Stroke risk by treatment subclass

SGLT2-Is and stroke by SGLT2-Is subclass

Use of SGLT2-I canagliflozin (RR, 0.82; 95% CI, 0.69−0.98; P=0.03) decreased stroke risk compared to placebo. However, use of other SGLT2-I subclasses (dapagliflozin, empagliflozin, ertugliflozin, and ipragliflozin) did not decrease stroke risk compared to placebo or AHGs using fixed- and random-effect models (Figure S21 and Figure S22). The GRADE scores were moderate (Table S169).

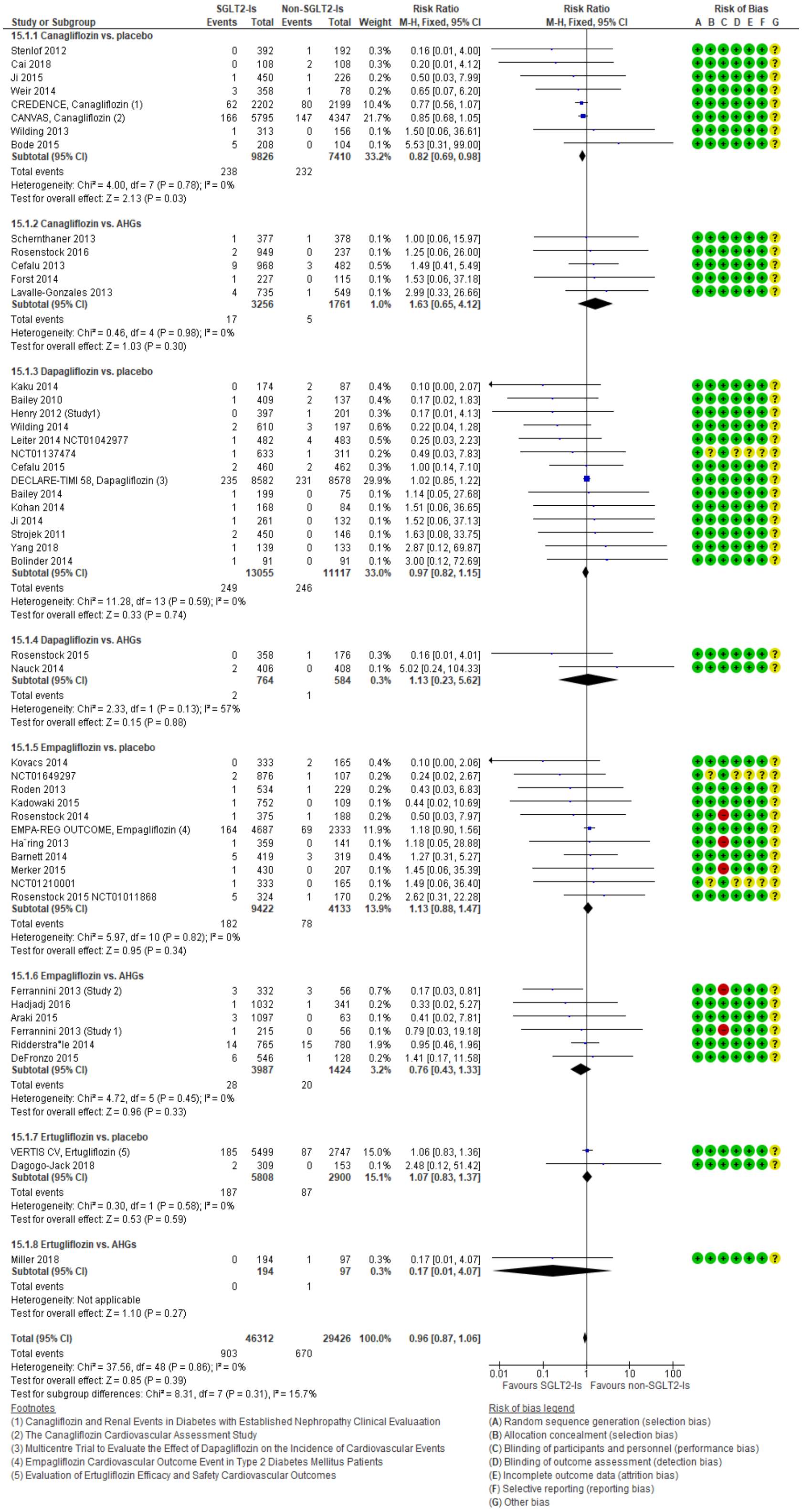

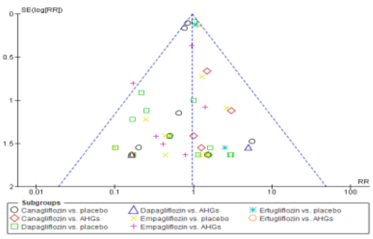

Figure S21. Forest and funnel plot of SGLT2-Is and stroke by subclass, Fixed-effect model.

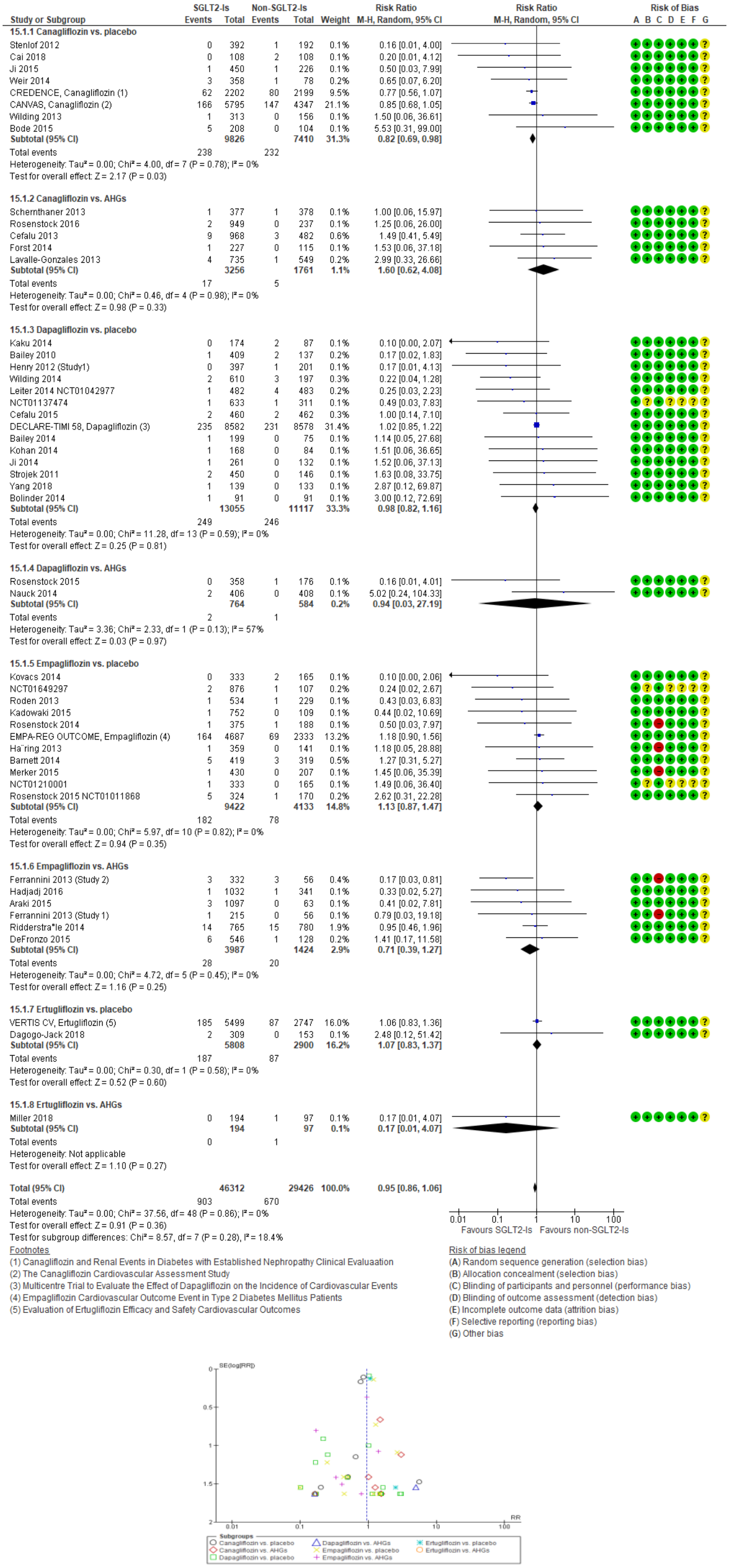

Figure S22. Forest and funnel plot of SGLT2-Is and stroke by subclass, Random-effect model.

Table S16. GRADE evidence profile of SGLT2-Is and stroke by subclass

Certainty assessment							Summary of findings				
№ of participants (trials)	Risk of bias	Inconsistency	Indirectness	Imprecision	Publication bias	Overall certainty of evidence	Study event rates (%)		Relative effect (95% CI)	Anticipated absolute effects	
							With controls	With SGLT2-Is		Risk with controls	Risk difference with SGLT2-Is
**Risk of stroke in T2DM by SGLT2-Is subclass (follow-up: range 12-318 weeks)**											
75738 (49 RCTs)	serious[a]	not serious	not serious	not serious	none	⊕⊕⊕◯ MODERATE	670/29426 (2.3%)	903/46312 (1.9%)	RR 0.96 (0.87 to 1.06)	2 per 100	0 fewer per 100 (from 0 fewer to 0 fewer)
**Canagliflozin vs. placebo (follow-up: range 18-104 weeks)**											
17236 (8 RCTs)	not serious	not serious	not serious	serious[b]	none	⊕⊕⊕◯ MODERATE	232/7410 (3.1%)	238/9826 (2.4%)	RR 0.82 (0.69 to 0.98)	3 per 100	1 fewer per 100 (from 1 fewer to 0 fewer)
**Canagliflozin vs. AHGs (follow-up: range 12-52 weeks)**											
5017 (5 RCTs)	not serious	not serious	not serious	very serious[c]	none	⊕⊕◯◯ LOW	5/1761 (0.3%)	17/3256 (0.5%)	RR 1.63 (0.65 to 4.12)	0 per 100	0 fewer per 100 (from 0 fewer to 1 more)
**Dapagliflozin vs. placebo (follow-up: range 12-104 weeks)**											
24172 (14 RCTs)	not serious	not serious	not serious	serious[d]	none	⊕⊕⊕◯ MODERATE	246/11117 (2.2%)	249/13055 (1.9%)	RR 0.97 (0.82 to 1.15)	2 per 100	0 fewer per 100 (from 0 fewer to 0 fewer)
**Dapagliflozin vs. AHGs (follow-up: range 24-156 weeks)**											
1348 (2 RCTs)	not serious	not serious	not serious	very serious[e]	none	⊕⊕◯◯ LOW	1/584 (0.2%)	2/764 (0.3%)	RR 1.13 (0.23 to 5.62)	0 per 100	0 fewer per 100 (from 0 fewer to 1 more)
**Empagliflozin vs. placebo (follow-up: range 12-161 weeks)**											
13555 (11 RCTs)	serious[a]	not serious	not serious	very serious[f]	none	⊕◯◯◯ VERY LOW	78/4133 (1.9%)	182/9422 (1.9%)	RR 1.13 (0.88 to 1.47)	2 per 100	0 fewer per 100 (from 0 fewer to 1 more)
**Empagliflozin vs. AHGs (follow-up: range 26-104 weeks)**											

Certainty assessment							Summary of findings				
5411 (6 RCTs)	serious[a]	not serious	not serious	very serious[g]	none	⊕◯◯◯ VERY LOW	20/1424 (1.4%)	28/3987 (0.7%)	RR 0.76 (0.43 to 1.33)	1 per 100	0 fewer per 100 (from 1 fewer to 0 fewer)
**Ertugliflozin vs. placebo (follow-up: range 183-318 weeks)**											
8708 (2 RCT)	not serious	not serious	not serious	very serious[h]	none	⊕⊕◯◯ LOW	87/2900 (3.0%)	187/5808 (3.2%)	RR 1.07 (0.83 to 1.37)	3 per 100	0 fewer per 100 (from 1 fewer to 1 more)
**Ertugliflozin vs. AHGs (follow-up: mean 26 weeks)**											
291 (1 RCT)	not serious	not serious	not serious	very serious[i]	none	⊕⊕◯◯ LOW	1/97 (1.0%)	0/194 (0.0%)	RR 0.17 (0.01 to 4.07)	1 per 100	1 fewer per 100 (from 1 fewer to 3 more)

Abbreviations: **AHGs,** anti-hyperglycaemic agents; **CI,** confidence interval; **RCTs,** randomised controlled trials; **RR,** risk ratio; **SGLT2-Is,** sodium-glucose cotransporter-2 inhibitors.

Note: The GRADE scores were from the fixed-effect model.

GRADE evidence

a. Five trials with small weight SGLT2-I empagliflozin vs. placebo (0.2%, 0.1%, and 0.1%) and empagliflozin vs. AHGs (0.7% and 0.1%) with the overall EE rated as high risk of bias due to lack of blinding (open-label design) out of 49 trials.

b. The overall imprecision was precise, with no significant effect size difference (P=0.03). However, all trials reported overlapping CIs, in which two trials reported wide CIs. The 95% CI was consistent with the possibility of a benefit exceeding the MID, including only 470 events with a large sample size.

c. The overall imprecision was precise, with no significant effect size difference (P=0.30). However, all trials reported overlapping CIs, in which four trials reported wide CIs. The 95% CI was consistent with the possibility of a benefit not exceeding the MID, including only 22 events with a large sample size.

d. The overall imprecision was precise, with no significant effect size difference (P=0.74). However, all trials reported overlapping CIs, in which six trials reported wide CIs. The 95%

CI was consistent with the possibility of a benefit exceeding the MID, including 495 events with a large sample size.

e. The overall imprecision was precise, with no significant effect size difference (P=0.88). However, all trials reported overlapping CIs, in which one trial reported a wide CI. The 95% CI was consistent with the possibility of a benefit not exceeding the MID, including only three events with a small sample size (1,348).

f. The overall imprecision was precise, with no significant effect size difference (P=0.34). However, all trials reported overlapping CIs, in which four trials reported wide CIs. The 95% CI was consistent with the possibility of a benefit not exceeding the MID, including only 260 events with a large sample size.

g. The overall imprecision was precise, with no significant effect size difference (P=0.33). However, all trials reported overlapping CIs, in which two trials reported wide CIs. The 95% CI was consistent with the possibility of a large benefit exceeding the MID, including only 48 events with a large sample size.

h. The overall imprecision was precise, with no significant effect size difference (P=0.59). However, all trials reported overlapping CIs, in which one trial reported wide CI. The 95% CI was consistent with the possibility of a benefit not exceeding the MID, including only 274 events with a small sample size (462).

i. The overall imprecision was precise, with no significant effect size difference (P=0.27). However, all trials reported overlapping and narrow CIs. The 95% CI was consistent with the possibility of a benefit not exceeding the MID, including only one event with a small sample size (291).

In summary, the overall certainty of the pooled EE had moderate imprecision without a significant effect size difference in the level of evidence (P=0.39). However, the majority of trials reported overlapping CIs, in which 20 trials reported wide CIs. In total, only one small

trial (0.7%) did not cross the line of no difference (1), in which there was a significant reduction of stroke in the direction of SGLT2-I empagliflozin favouring active comparators of AHGs. The 95% CI was consistent with the possibility of a large benefit exceeding the MID, including a total of 1,573 events with a large sample size (75,738). There was no evidence of inconsistency, statistically significant heterogeneity (P=0.86; I^2=0%), or subgroup difference (P=0.31; I^2=15.7%). The majority of trials had low risk of bias (98.8%). There was no evidence of detection bias. However, the magnitude of the impact of some trials that suffered from limitations likely resulted in biased assessment of the intervention effect due to performance and selection bias (five trials with open-label design out of 49). The indirectness of the trials indicated a high level of evidence, with an individual trial PICOS element aligned closely to the review PICOS. Finally, there was no evidence of reporting bias. However, one trial reported an outlier outside the pyramid edge due to multiple interventional groups, consequently resulting in a very wide CI.

GLP1-RAs and stroke by GLP1-RAs subclass

Use of GLP1-RAs dulaglutide (RR, 0.79; 95% CI, 0.64−0.96; P=0.02) and semaglutide (RR, 0.65; 95% CI, 0.44−0.97; P=0.03) decreased stroke risk compared to placebo, in fixed-effect model only. However, use of other GLP1-RA subclasses (albiglutide, exenatide, liraglutide, lixisenatide, and taspoglutide) did not decrease stroke risk compared to placebo or AHGs using fixed- and random-effect models. Figure S23 and Figure S24. The GRADE scores were moderate (Table S17).

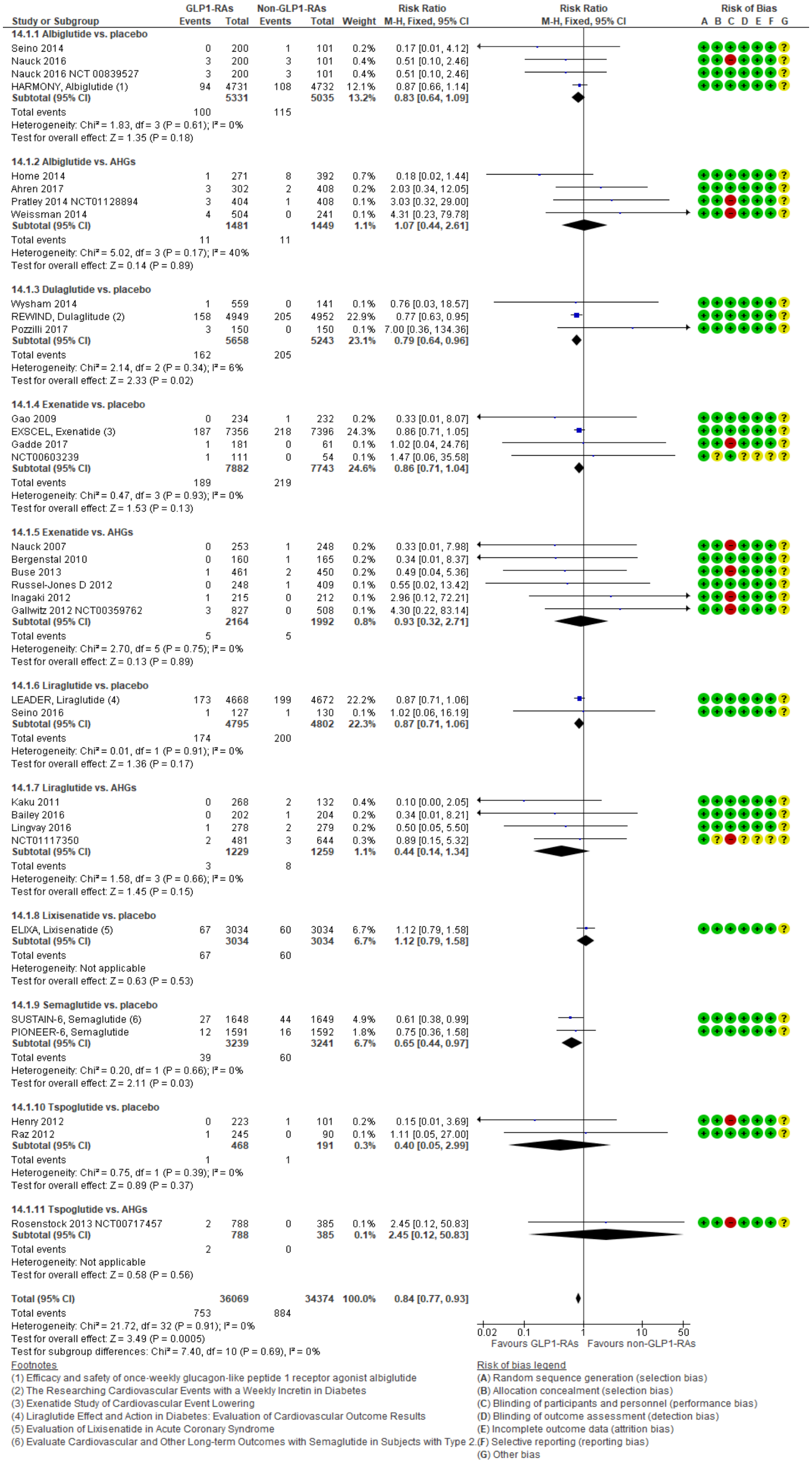

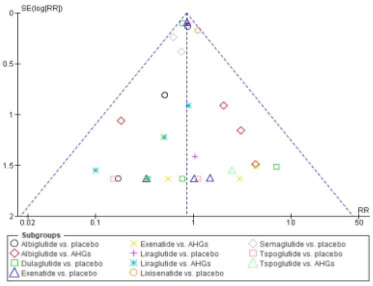

Figure S23. Forest and funnel plot of GLP1-RAs and stroke by GLP1-RAs subclass, Fixed-effect model.

Study or Subgroup	GLP1-RAs Events	GLP1-RAs Total	Non-GLP1-RAs Events	Non-GLP1-RAs Total	Weight	Risk Ratio M-H, Random, 95% CI
**14.1.1 Albiglutide vs. placebo**						
Seino 2014	0	200	1	101	0.1%	0.17 [0.01, 4.12]
Nauck 2016	3	200	3	101	0.4%	0.51 [0.10, 2.46]
Nauck 2016 NCT 00839527	3	200	3	101	0.4%	0.51 [0.10, 2.46]
HARMONY, Albiglutide (1)	94	4731	108	4732	12.4%	0.87 [0.66, 1.14]
**Subtotal (95% CI)**		**5331**		**5035**	**13.2%**	**0.83 [0.64, 1.09]**
Total events	100		115			
Heterogeneity: Tau² = 0.00; Chi² = 1.83, df = 3 (P = 0.61); I² = 0%						
Test for overall effect: Z = 1.34 (P = 0.18)						
**14.1.2 Albiglutide vs. AHGs**						
Home 2014	1	271	8	392	0.2%	0.18 [0.02, 1.44]
Ahren 2017	3	302	2	408	0.3%	2.03 [0.34, 12.05]
Pratley 2014 NCT01128894	3	404	1	408	0.2%	3.03 [0.32, 29.00]
Weissman 2014	4	504	0	241	0.1%	4.31 [0.23, 79.78]
**Subtotal (95% CI)**		**1481**		**1449**	**0.8%**	**1.33 [0.32, 5.52]**
Total events	11		11			
Heterogeneity: Tau² = 0.84; Chi² = 5.02, df = 3 (P = 0.17); I² = 40%						
Test for overall effect: Z = 0.39 (P = 0.70)						
**14.1.3 Dulaglutide vs. placebo**						
Wysham 2014	1	559	0	141	0.1%	0.76 [0.03, 18.57]
REWIND, Dulaglitude (2)	158	4949	205	4952	22.3%	0.77 [0.63, 0.95]
Pozzilli 2017	3	150	0	150	0.1%	7.00 [0.36, 134.36]
**Subtotal (95% CI)**		**5658**		**5243**	**22.5%**	**0.84 [0.47, 1.51]**
Total events	162		205			
Heterogeneity: Tau² = 0.09; Chi² = 2.14, df = 2 (P = 0.34); I² = 6%						
Test for overall effect: Z = 0.59 (P = 0.55)						
**14.1.4 Exenatide vs. placebo**						
Gao 2009	0	234	1	232	0.1%	0.33 [0.01, 8.07]
EXSCEL, Exenatide (3)	187	7356	218	7396	24.9%	0.86 [0.71, 1.05]
Gadde 2017	1	181	0	61	0.1%	1.02 [0.04, 24.76]
NCT00603239	1	111	0	54	0.1%	1.47 [0.06, 35.58]
**Subtotal (95% CI)**		**7882**		**7743**	**25.2%**	**0.86 [0.71, 1.04]**
Total events	189		219			
Heterogeneity: Tau² = 0.00; Chi² = 0.47, df = 3 (P = 0.93); I² = 0%						
Test for overall effect: Z = 1.52 (P = 0.13)						
**14.1.5 Exenatide vs. AHGs**						
Nauck 2007	0	253	1	248	0.1%	0.33 [0.01, 7.98]
Bergenstal 2010	0	160	1	165	0.1%	0.34 [0.01, 8.37]
Buse 2013	1	461	2	450	0.2%	0.49 [0.04, 5.36]
Russel-Jones D 2012	0	248	1	409	0.1%	0.55 [0.02, 13.42]
Inagaki 2012	1	215	0	212	0.1%	2.96 [0.12, 72.21]
Gallwitz 2012 NCT00359762	3	827	0	508	0.1%	4.30 [0.22, 83.14]
**Subtotal (95% CI)**		**2164**		**1992**	**0.6%**	**0.83 [0.25, 2.80]**
Total events	5		5			
Heterogeneity: Tau² = 0.00; Chi² = 2.70, df = 5 (P = 0.75); I² = 0%						
Test for overall effect: Z = 0.30 (P = 0.77)						
**14.1.6 Liraglutide vs. placebo**						
LEADER, Liraglutide (4)	173	4668	199	4672	23.2%	0.87 [0.71, 1.06]
Seino 2016	1	127	1	130	0.1%	1.02 [0.06, 16.19]
**Subtotal (95% CI)**		**4795**		**4802**	**23.3%**	**0.87 [0.71, 1.06]**
Total events	174		200			
Heterogeneity: Tau² = 0.00; Chi² = 0.01, df = 1 (P = 0.91); I² = 0%						
Test for overall effect: Z = 1.36 (P = 0.17)						
**14.1.7 Liraglutide vs. AHGs**						
Kaku 2011	0	268	2	132	0.1%	0.10 [0.00, 2.05]
Bailey 2016	0	202	1	204	0.1%	0.34 [0.01, 8.21]
Lingvay 2016	1	278	2	279	0.2%	0.50 [0.05, 5.50]
NCT01117350	2	481	3	644	0.3%	0.89 [0.15, 5.32]
**Subtotal (95% CI)**		**1229**		**1259**	**0.6%**	**0.48 [0.14, 1.58]**
Total events	3		8			
Heterogeneity: Tau² = 0.00; Chi² = 1.58, df = 3 (P = 0.66); I² = 0%						
Test for overall effect: Z = 1.21 (P = 0.23)						
**14.1.8 Lixisenatide vs. placebo**						
ELIXA, Lixisenatide (5)	67	3034	60	3034	7.8%	1.12 [0.79, 1.58]
**Subtotal (95% CI)**		**3034**		**3034**	**7.8%**	**1.12 [0.79, 1.58]**
Total events	67		60			
Heterogeneity: Not applicable						
Test for overall effect: Z = 0.63 (P = 0.53)						
**14.1.9 Semaglutide vs. placebo**						
SUSTAIN-6, Semaglutide (6)	27	1648	44	1649	4.1%	0.61 [0.38, 0.99]
PIONEER-6, Semaglutide	12	1591	16	1592	1.7%	0.75 [0.36, 1.58]
**Subtotal (95% CI)**		**3239**		**3241**	**5.8%**	**0.65 [0.44, 0.97]**
Total events	39		60			
Heterogeneity: Tau² = 0.00; Chi² = 0.20, df = 1 (P = 0.66); I² = 0%						
Test for overall effect: Z = 2.11 (P = 0.04)						
**14.1.10 Tspoglutide vs. placebo**						
Henry 2012	0	223	1	101	0.1%	0.15 [0.01, 3.69]
Raz 2012	1	245	0	90	0.1%	1.11 [0.05, 27.00]
**Subtotal (95% CI)**		**468**		**191**	**0.2%**	**0.41 [0.04, 3.92]**
Total events	1		1			
Heterogeneity: Tau² = 0.00; Chi² = 0.75, df = 1 (P = 0.39); I² = 0%						
Test for overall effect: Z = 0.77 (P = 0.44)						
**14.1.11 Tspoglutide vs. AHGs**						
Rosenstock 2013 NCT00717457	2	788	0	385	0.1%	2.45 [0.12, 50.83]
**Subtotal (95% CI)**		**788**		**385**	**0.1%**	**2.45 [0.12, 50.83]**
Total events	2		0			
Heterogeneity: Not applicable						
Test for overall effect: Z = 0.58 (P = 0.56)						
**Total (95% CI)**		**36069**		**34374**	**100.0%**	**0.84 [0.77, 0.93]**
Total events	753		884			
Heterogeneity: Tau² = 0.00; Chi² = 21.72, df = 32 (P = 0.91); I² = 0%						
Test for overall effect: Z = 3.47 (P = 0.0005)						
Test for subgroup differences: Chi² = 6.30, df = 10 (P = 0.79), I² = 0%						

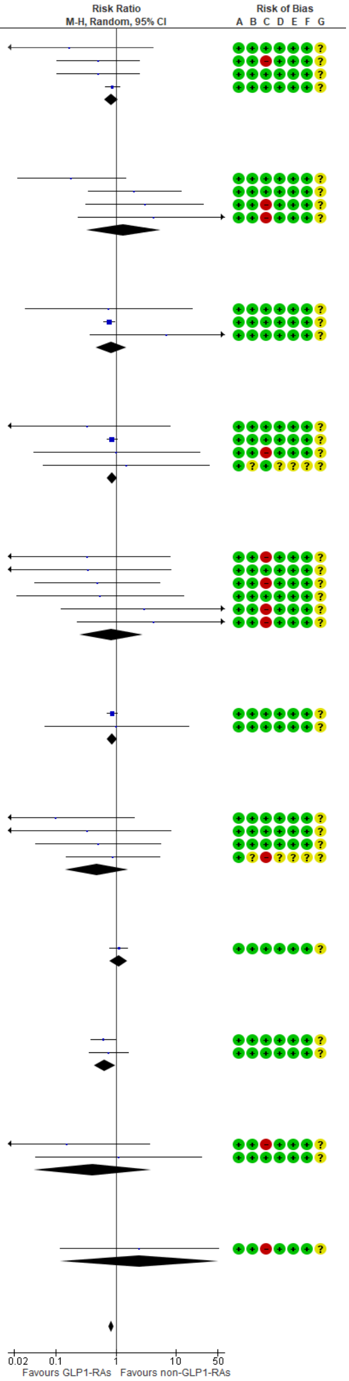

Footnotes
(1) Efficacy and safety of once-weekly glucagon-like peptide 1 receptor agonist albiglutide
(2) The Researching Cardiovascular Events with a Weekly Incretin in Diabetes
(3) Exenatide Study of Cardiovascular Event Lowering
(4) Liraglutide Effect and Action in Diabetes: Evaluation of Cardiovascular Outcome Results
(5) Evaluation of Lixisenatide in Acute Coronary Syndrome
(6) Evaluate Cardiovascular and Other Long-term Outcomes with Semaglutide in Subjects with Type 2...

Risk of bias legend
(A) Random sequence generation (selection bias)
(B) Allocation concealment (selection bias)
(C) Blinding of participants and personnel (performance bias)
(D) Blinding of outcome assessment (detection bias)
(E) Incomplete outcome data (attrition bias)
(F) Selective reporting (reporting bias)
(G) Other bias

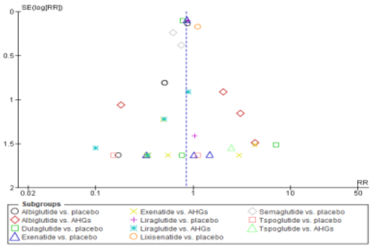

Figure S24. Forest and funnel plot of GLP1-RAs and stroke by GLP1-RAs subclass, Random-effect model.

Table S17. GRADE evidence profile of GLP1-RAs and stroke by GLP1-RAs subclass

Certainty assessment							Summary of findings				
№ of participants (trials)	Risk of bias	Inconsistency	Indirectness	Imprecision	Publication bias	Overall certainty of evidence	Study event rates (%)		Relative effect (95% CI)	Anticipated absolute effects	
							With controls	With GLP1-RAs		Risk with controls	Risk difference with GLP1-RAs
**Risk of stroke in T2DM by GLP1-RAs subclass (follow-up: range 16-339 weeks)**											
70443 (33 trials)	serious[a]	not serious	not serious	not serious	none	⨁⨁⨁◯ MODERATE	884/34374 (2.6%)	753/36069 (2.1%)	RR 0.84 (0.77 to 0.93)	3 per 100	0 fewer per 100 (from 1 fewer to 0 fewer)
**Albiglutide vs. placebo (follow-up: range 24-156 weeks)**											
10366 (4 RCTs)	serious[a]	not serious	not serious	very serious[b]	none	⨁◯◯◯ VERY LOW	115/5035 (2.3%)	100/5331 (1.9%)	RR 0.83 (0.64 to 1.09)	2 per 100	0 fewer per 100 (from 1 fewer to 0 fewer)
**Albiglutide vs. AHGs (follow-up: range 32-156 weeks)**											
2930 (4 RCTs)	serious[a]	not serious	not serious	very serious[c]	none	⨁◯◯◯ VERY LOW	11/1449 (0.8%)	11/1481 (0.7%)	RR 1.07 (0.44 to 2.61)	1 per 100	0 fewer per 100 (from 0 fewer to 1 more)
**Dulaglutide vs. placebo (follow-up: range 28-339 weeks)**											
10901 (3 RCTs)	not serious	not serious	not serious	serious[d]	none	⨁⨁⨁◯ MODERATE	205/5243 (3.9%)	162/5658 (2.9%)	RR 0.79 (0.64 to 0.96)	4 per 100	1 fewer per 100 (from 1 fewer to 0 fewer)
**Exenatide vs. placebo (follow-up: range 16-167 weeks)**											
15625 (4 RCTs)	serious[a]	not serious	not serious	serious[e]	none	⨁⨁◯◯ LOW	219/7743 (2.8%)	189/7882 (2.4%)	RR 0.86 (0.71 to 1.04)	3 per 100	0 fewer per 100 (from 1 fewer to 0 fewer)
**Exenatide vs. AHGs (follow-up: range 26-235 weeks)**											
4156 (6 RCTs)	serious[a]	not serious	not serious	very serious[f]	none	⨁◯◯◯ VERY LOW	5/1992 (0.3%)	5/2164 (0.2%)	RR 0.93 (0.32 to 2.71)	0 per 100	0 fewer per 100 (from 0 fewer to 0 fewer)
**Liraglutide vs. placebo (follow-up: range 36-198 weeks)**											

Certainty assessment							Summary of findings				
9597 (2 RCTs)	not serious	not serious	not serious	serious[g]	none	⨁⨁⨁◯ MODERATE	200/4802 (4.2%)	174/4795 (3.6%)	RR 0.87 (0.71 to 1.06)	4 per 100	1 fewer per 100 (from 1 fewer to 0 fewer)
**Liraglutide vs. AHGs (follow-up: range 62-52 weeks)**											
2488 (4 RCTs)	serious[a]	not serious	not serious	very serious[h]	none	⨁◯◯◯ VERY LOW	8/1259 (0.6%)	3/1229 (0.2%)	RR 0.44 (0.14 to 1.34)	1 per 100	0 fewer per 100 (from 1 fewer to 0 fewer)
**Lixisenatide vs. placebo (follow-up: mean 109 weeks)**											
6068 (1 RCT)	not serious	not serious	not serious	very serious[i]	none	⨁⨁◯◯ LOW	60/3034 (2.0%)	67/3034 (2.2%)	RR 1.12 (0.79 to 1.58)	2 per 100	0 fewer per 100 (from 0 fewer to 1 more)
**Semaglutide vs. placebo (follow-up: mean 104 weeks)**											
6480 (2 RCTs)	not serious	not serious	not serious	very serious[j]	none	⨁⨁◯◯ LOW	60/3241 (1.9%)	39/3239 (1.2%)	RR 0.65 (0.44 to 0.97)	2 per 100	1 fewer per 100 (from 1 fewer to 0 fewer)
**Tspoglutide vs. placebo (follow-up: mean 24 weeks)**											
659 (2 RCTs)	serious[a]	not serious	not serious	very serious[k]	none	⨁◯◯◯ VERY LOW	1/191 (0.5%)	1/468 (0.2%)	RR 0.40 (0.05 to 2.99)	1 per 100	0 fewer per 100 (from 0 fewer to 1 more)
**Tspoglutide vs. AHGs (follow-up: mean 104 weeks)**											
1173 (1 RCT)	serious[a]	not serious	not serious	very serious[l]	none	⨁◯◯◯ VERY LOW	0/385 (0.0%)	2/788 (0.3%)	RR 2.45 (0.12 to 50.83)	0 per 100	0 fewer per 100 (from 0 fewer to 0 fewer)

Abbreviations: **AHGs,** anti-hyperglycaemic agents; **CI,** confidence interval; **GLP1-RAs,** glucagon-like receptor peptide-1 agonists; **RCTs,** randomised controlled trials; **RR,** risk ratio.

Note: The GRADE scores were from the fixed-effect model.

GRADE evidence

a. Eleven trials with small weight GLP1-RAs vs. placebo (0.4%, 0.2%, and 0.1%) and GLP1-RAs vs. AHGs (0.3%, 0.2%, 0.2%, 0.1%, 0.1%, 0.1%, 0.1%, and 0.1%) with the overall EE rated as high risk of bias due to lack of blinding (open-label design) out of 33 trials, one of which was an unpublished RCT.

b. The overall imprecision was precise, with no significant effect size difference (P=0.18). However, all trials reported overlapping and narrow CIs. The 95% CI was consistent with the possibility of a benefit not exceeding the MID, including only 215 events with a large sample size.

c. The overall imprecision was precise, with no significant effect size difference (P=0.89). However, all trials reported overlapping CIs, in which three trials reported wide CIs. The 95% CI was consistent with the possibility of a benefit not exceeding the MID, including only 22 events with a large sample size.

d. The overall imprecision was precise, with a significant effect size difference (P=0.02). However, all trials reported overlapping CIs, in which two trials reported wide CIs. The 95% CI was consistent with the possibility of a benefit not exceeding the MID, including only 367 events with a large sample size.

e. The overall imprecision was precise, with no significant effect size difference (P=0.13). However, all trials reported overlapping CIs, in which two trials reported wide CIs. The 95% CI was consistent with the possibility of a benefit not exceeding the MID, including 408 events with a large sample size.

f. The overall imprecision was precise, with no significant effect size difference (P=0.89). However, all trials reported overlapping CIs, in which three trials reported wide CIs. The 95% CI was consistent with the possibility of a benefit not exceeding the MID, including only 10 events with a large sample size.

g. The overall imprecision was precise, with no significant effect size difference (P=0.17). However, all trials reported overlapping CIs, in which one trial reported a wide CI. The 95% CI was consistent with the possibility of a benefit not exceeding the MID, including only 374 events with a large sample size.

h. The overall imprecision was precise, with no significant effect size difference (P=0.15). However, all trials reported overlapping and narrow CIs. The 95% CI was consistent with the possibility of a benefit not exceeding the MID, including only 11 events with a large sample size.

i. The overall imprecision was precise, with no significant effect size difference (P=0.53). However, all trials reported overlapping and narrow CIs. The 95% CI was consistent with the possibility of a benefit not exceeding the MID, including only 127 events with a large sample size.

j. The overall imprecision was precise, with a significant effect size difference (P=0.03). However, all trials reported overlapping and narrow CIs. The 95% CI was consistent with the possibility of a benefit not exceeding the MID, including only 99 events with a large sample size.

k. The overall imprecision was precise, with no significant effect size difference (P=0.37). However, all trials reported overlapping CIs, in which one trial reported a wide CI. The 95% CI was consistent with the possibility of a benefit not exceeding the MID, including only two events with a small sample size (659).

l. The overall imprecision was precise, with no significant effect size difference (P=0.56). However, all trials reported overlapping CIs, in which one trial reported a wide CI. The 95% CI was consistent with the possibility of a benefit not exceeding the MID, including only two events with a small sample size (1,173).

In summary, the overall certainty of the pooled EE had moderate imprecision with a significant effect size difference in the level of evidence (P=0.0005). However, the majority of trials reported overlapping CIs, in which 13 trials reported wide CIs. In total, only one large (22.9%) and one small (4.9%) trials did not cross the line of no difference (1), in which a significant reduction of stroke in the direction of GLP1-RAs favoured placebo. The 95% CI was consistent

with the possibility of a benefit exceeding the MID, including a total of 1,637 events with a large sample size. There was no evidence of inconsistency, statistically significant heterogeneity (P=0.91; I^2=0%), or subgroup difference (P=0.69; I^2=0%). The majority of trials had low risk of bias (98.1%). There was no evidence of detection bias. However, the magnitude of the impact of some trials that suffered from limitations likely resulted in biased assessment of the intervention effect due to performance and selection bias (11 trials with open-label design out of 33, one of which was an unpublished RCT). The indirectness of the trials indicated a high level of evidence, with an individual trial PICOS element aligned closely to the review PICOS. Finally, there was no evidence of reporting bias; all included trials were within the two boundaries of the pyramid.

DPP4-Is and stroke by DPP4-I subclass

Use of DPP4-I dutogliptin decreased stroke risk (RR, 0.10; 95% CI, 0.01−0.90; P=0.04) compared to placebo. However, use of other DDP4-I subclasses (alogliptin, linagliptin, omarigliptin, saxagliptin, sitagliptin, and vildagliptin) did not decrease stroke risk compared to placebo or AHGs using fixed- and random-effect models (Figure S25 and Figure S26). The GRADE scores were moderate (Table S18).

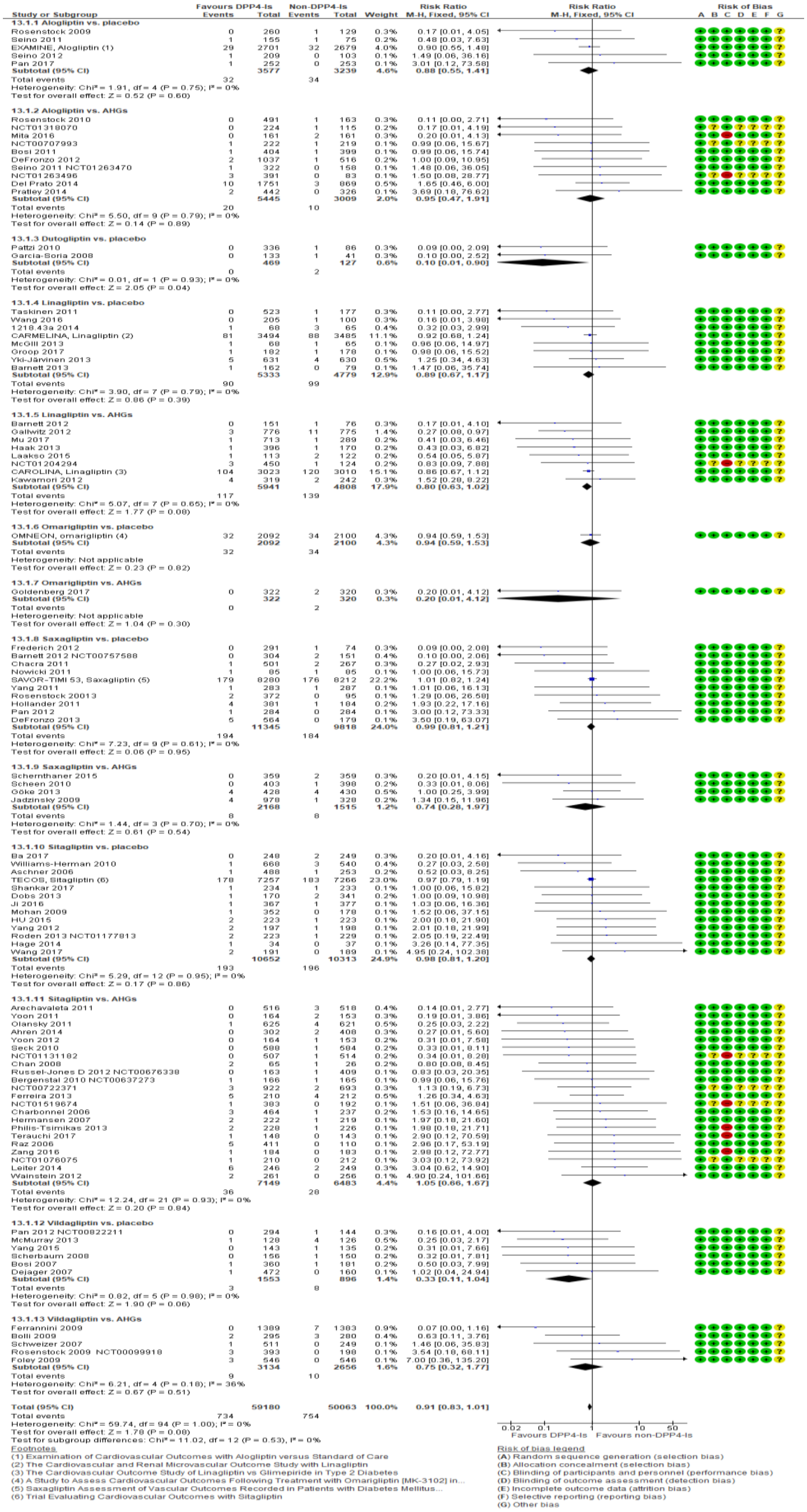

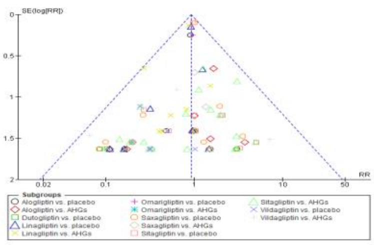

Figure S25. Forest and funnel plot of DPP4-Is and stroke by DPP4-Is subclass, Fixed-effect model.

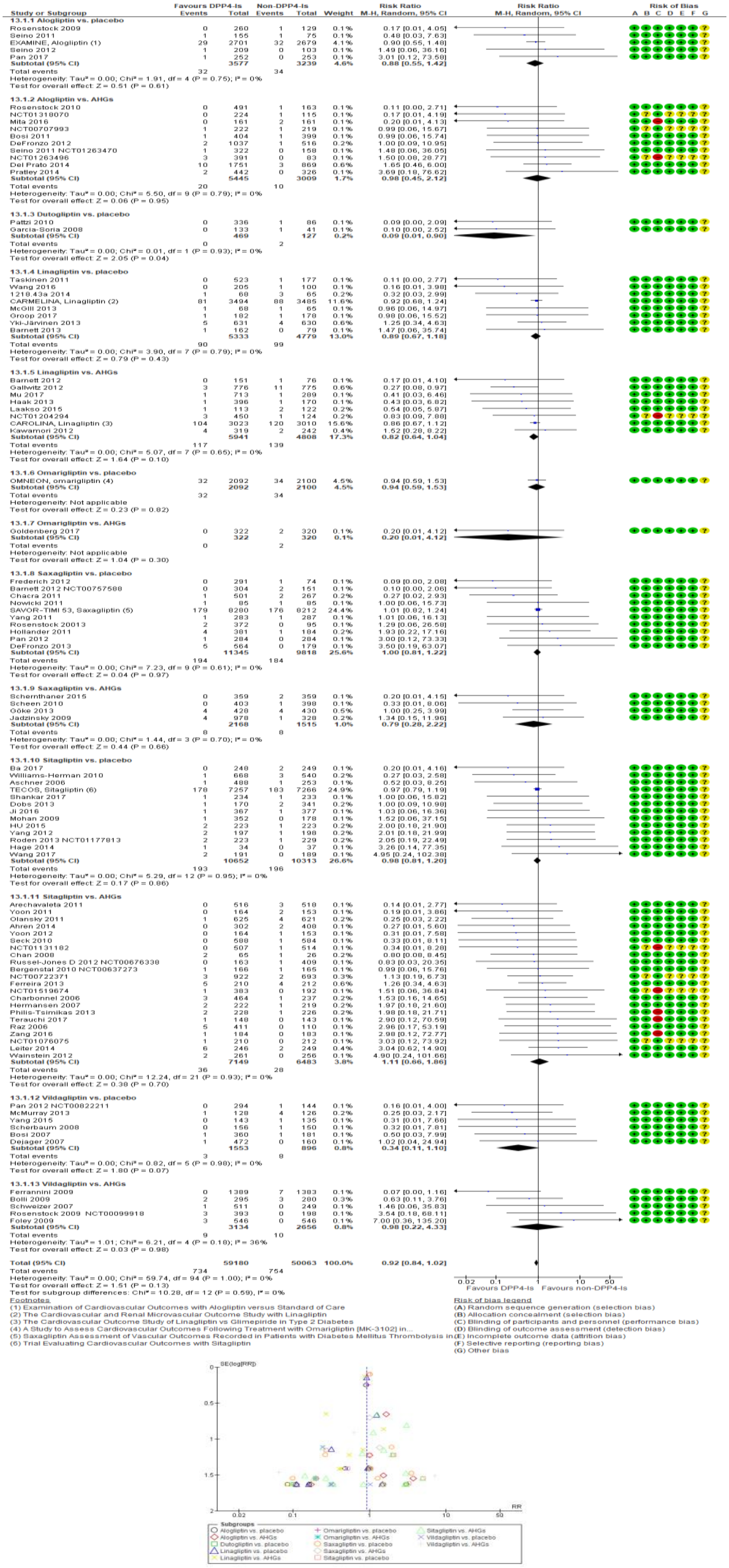

Figure S26. Forest and funnel plot of DPP4-Is and stroke by DPP4-Is subclass, Random-effect model.

Table S18. GRADE evidence profile of DPP4-Is and stroke by DPP4-Is subclass

Certainty assessment							Summary of findings				
№ of participants (trials)	Risk of bias	Inconsistency	Indirectness	Imprecision	Publication bias	Overall certainty of evidence	Study event rates (%)		Relative effect (95% CI)	Anticipated absolute effects	
							With controls	With DPP4-Is		Risk with controls	Risk difference with DPP4-Is
**Risk of stroke in T2DM by DPP4-Is subclass (follow-up: range 4-432 weeks)**											
109243 (95 RCTs)	serious[a]	not serious	not serious	not serious	none	⊕⊕⊕◯ MODERATE	754/50063 (1.5%)	734/59180 (1.2%)	RR 0.91 (0.83 to 1.01)	2 per 100	0 fewer per 100 (from 0 fewer to 0 fewer)
**Alogliptin vs. placebo (follow-up: range 12-78 weeks)**											
6816 (5 RCTs)	not serious	not serious	not serious	very serious[b]	none	⊕⊕◯◯ LOW	34/3239 (1.0%)	32/3577 (0.9%)	RR 0.88 (0.55 to 1.41)	1 per 100	0 fewer per 100 (from 0 fewer to 0 fewer)
**Alogliptin vs. AHGs (follow-up: range 12-104 weeks)**											
8454 (10 RCTs)	serious[a]	not serious	not serious	very serious[c]	none	⊕◯◯◯ VERY LOW	10/3009 (0.3%)	20/5445 (0.4%)	RR 0.95 (0.47 to 1.91)	0 per 100	0 fewer per 100 (from 0 fewer to 0 fewer)
**Dutogliptin vs. placebo (follow-up: range 4-12 weeks)**											
596 (2 RCTs)	not serious	not serious	not serious	very serious[d]	none	⊕⊕◯◯ LOW	2/127 (1.6%)	0/469 (0.0%)	RR 0.10 (0.01 to 0.90)	2 per 100	1 fewer per 100 (from 2 fewer to 0 fewer)
**Linagliptin vs. placebo (follow-up: range 24-224 weeks)**											
10112 (8 RCTs)	not serious	not serious	not serious	very serious[e]	none	⊕⊕◯◯ LOW	99/4779 (2.1%)	90/5333 (1.7%)	RR 0.89 (0.67 to 1.17)	2 per 100	0 fewer per 100 (from 1 fewer to 0 fewer)
**Linagliptin vs. AHGs (follow-up: range 24-432 weeks)**											
10749 (8 RCTs)	serious[a]	not serious	not serious	very serious[f]	none	⊕◯◯◯ VERY LOW	139/4808 (2.9%)	117/5941 (2.0%)	RR 0.80 (0.63 to 1.02)	3 per 100	1 fewer per 100 (from 1 fewer to 0 fewer)
**Omarigliptin vs. placebo (follow-up: mean 156 weeks)**											
4192 (1 RCT)	not serious	not serious	not serious	very serious[g]	none	⊕⊕◯◯ LOW	34/2100 (1.6%)	32/2092 (1.5%)	RR 0.94 (0.59 to 1.53)	2 per 100	0 fewer per 100 (from 1 fewer to 1 more)

Certainty assessment							Summary of findings				
**Omarigliptin vs. AHGs (follow-up: mean 24 weeks)**											
642 (1 RCT)	not serious	not serious	not serious	very serious[h]	none	⊕⊕◯◯ LOW	2/320 (0.6%)	0/322 (0.0%)	RR 0.20 (0.01 to 4.12)	1 per 100	1 fewer per 100 (from 1 fewer to 2 more)
**Saxagliptin vs. placebo (follow-up: range 24-206 weeks)**											
21163 (10 RCTs)	not serious	not serious	not serious	serious[i]	none	⊕⊕⊕◯ MODERATE	184/9818 (1.9%)	194/11345 (1.7%)	RR 1.99 (0.81 to 1.21)	2 per 100	0 fewer per 100 (from 0 fewer to 0 fewer)
**Saxagliptin vs. AHGs (follow-up: range 18-104 weeks)**											
3683 (4 RCTs)	not serious	not serious	not serious	very serious[j]	none	⊕⊕◯◯ LOW	8/1515 (0.5%)	8/2168 (0.4%)	RR 0.74 (0.28 to 1.97)	1 per 100	0 fewer per 100 (from 0 fewer to 1 more)
**Sitagliptin vs. placebo (follow-up: range 12-157 weeks)**											
20965 (13 RCTs)	not serious	not serious	not serious	serious[k]	none	⊕⊕⊕◯ MODERATE	196/10313 (1.9%)	193/10652 (1.8%)	RR 0.98 (0.81 to 1.20)	2 per 100	0 fewer per 100 (from 0 fewer to 0 fewer)
**Sitagliptin vs. AHGs (follow-up: range 4-104 weeks)**											
13632 (22 RCTs)	serious[a]	not serious	not serious	very serious[l]	none	⊕◯◯◯ VERY LOW	28/6483 (0.4%)	36/7149 (0.5%)	RR 1.05 (0.66 to 1.67)	0 per 100	0 fewer per 100 (from 0 fewer to 0 fewer)
**Vildagliptin vs. placebo (follow-up: range 24-52 weeks)**											
2449 (6 RCTs)	not serious	not serious	not serious	very serious[m]	none	⊕⊕◯◯ LOW	8/896 (0.9%)	3/1553 (0.2%)	RR 0.33 (0.11 to 1.04)	1 per 100	1 fewer per 100 (from 1 fewer to 0 fewer)
**Vildagliptin vs. AHGs (follow-up: range 52-104 weeks)**											
5790 (5 RCTs)	not serious	not serious	not serious	very serious[n]	none	⊕⊕◯◯ LOW	10/2656 (0.4%)	9/3134 (0.3%)	RR 0.75 (0.32 to 1.77)	0 per 100	0 fewer per 100 (from 0 fewer to 0 fewer)

Abbreviations: **AHGs,** anti-hyperglycaemic agents; **CI,** confidence interval; **DPP4-Is,** dipeptidyl peptidase-4 inhibitors; **RCTs,** randomised controlled trials; **RR,** risk ratio.

Note: The GRADE scores were from the fixed-effect model.

GRADE evidence

a. Eight trials with small weight DPP4-Is vs. AHGs (0.3%, 0.2%, 0.2%, 0.1%, 0.1%, 0.1%, 0.1%, and 0.1%) with the overall EE rated as high risk of bias due to lack of blinding (open-label design) out of 95 trials, four of which were unpublished RCTs.

b. The overall imprecision was precise, with no significant effect size difference (P=0.60). However, all trials reported overlapping CIs, in which two trials reported wide CIs. The 95% CI was consistent with the possibility of a benefit not exceeding the MID, including only 66 events with a large sample size.

c. The overall imprecision was precise, with no significant effect size difference (P=0.89). However, all trials reported overlapping CIs, in which five trials reported wide CIs. The 95% CI was consistent with the possibility of a benefit not exceeding the MID, including only 30 events with a large sample size.

d. The overall imprecision was precise, with a significant effect size difference (P=0.04). However, all trials reported overlapping and narrow CIs. The 95% CI was consistent with the possibility of a benefit not exceeding the MID, including only two events with a small sample size (596).

e. The overall imprecision was precise, with no significant effect size difference (P=0.39). However, all trials reported overlapping CIs, in which three trials reported wide CIs. The 95% CI was consistent with the possibility of a benefit not exceeding the MID, including only 189 events with a large sample size.

f. The overall imprecision was precise, with no significant effect size difference (P=0.08). However, all trials reported overlapping and narrow CIs. The 95% CI was consistent with the possibility of a benefit not exceeding the MID, including only 256 events with a large sample size.

g. The overall imprecision was precise, with no significant effect size difference (P=0.82). However, all trials reported overlapping and narrow CIs. The 95% CI was consistent with the possibility of a benefit not exceeding the MID, including only 66 events with a large sample size.

h. The overall imprecision was precise, with no significant effect size difference (P=0.30). However, all trials reported overlapping and narrow CIs. The 95% CI was consistent with the possibility of a benefit not exceeding the MID, including only two events with a small sample size (642).

i. The overall imprecision was precise, with no significant effect size difference (P=0.95). However, all trials reported overlapping CIs, in which six trials reported wide CIs. The 95% CI was consistent with the possibility of a benefit not exceeding the MID, including only 378 events with a large sample size.

j. The overall imprecision was precise, with no significant effect size difference (P=0.54). However, all trials reported overlapping CIs, in which one trial reported a wide CI. The 95% CI was consistent with the possibility of a benefit not exceeding the MID, including only 16 events with a large sample size.

k. The overall imprecision was precise, with no significant effect size difference (P=0.86). However, all trials reported overlapping CIs, in which eight trials reported wide CIs. The 95% CI was consistent with the possibility of a large benefit exceeding the MID, including only 389 events with a large sample size.

l. The overall imprecision was precise, with no significant effect size difference (P=0.84). However, all trials reported overlapping CIs, in which 12 trials reported wide CIs. The 95% CI was consistent with the possibility of a benefit not exceeding the MID, including only 64 events with a large sample size.

m. The overall imprecision was precise, with no significant effect size difference (P=0.06). However, all trials reported overlapping CIs, in which one trial reported a wide CI. The 95% CI was consistent with the possibility of a benefit not exceeding the MID, including only 11 events with a large sample size.

n. The overall imprecision was precise, with no significant effect size difference (P=0.51). However, all trials reported overlapping CIs, in which three trials reported wide CIs. The 95% CI was consistent with the possibility of a benefit not exceeding the MID, including only 19 events with a large sample size.

In summary, the overall certainty of the pooled EE had moderate imprecision without a significant effect size difference in the level of evidence (P=0.08). However, the majority of trials reported overlapping CIs, in which 41 trials reported wide CIs. In total, only one small trial (0.6%) did not cross the line of no difference (1), in which there was a significant reduction in stroke in the direction of DPP4-Is favouring active comparators of AHGs. The 95% CI was consistent with the possibility of a large benefit exceeding the MID, including a total of 1,488 events with a large sample size (109,243). There was no evidence of inconsistency, statistically significant heterogeneity (P=1.00; I^2=0%), or subgroup difference (P=0.53; I^2=0%). The majority of trials had low risk of bias (98.8%). There was no evidence of detection bias. However, the magnitude of the impact of some trials that suffered from limitations likely resulted in biased assessment of the intervention effect due to performance and selection bias (eight trials with open-label design out of 95). The indirectness of trials indicated a high level of evidence with an individual trial PICOS element aligned closely to the review PICOS. Finally, there was no evidence of reporting bias; all included trials were within the two boundaries of the pyramid.

Stroke risk by AHG subclass

SGLT2-Is and stroke by AHGs subclass

Use of SGLT2-Is did not change stroke risk (RR, 1.00; 95% CI, 0.59−1.71; P=0.99) compared to other classes of AHGs using fixed- and random-effect models (Figure S27 and Figure S28). The GRADE scores were low (Table S19).

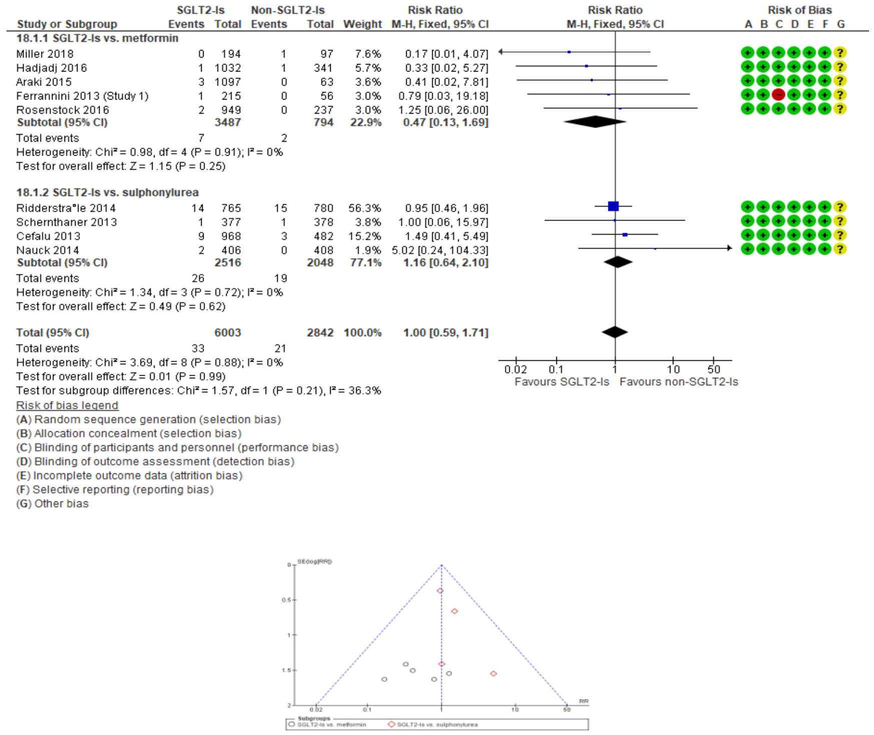

Figure S27. Forest and funnel plot of SGLT2-Is and stroke by AHGs subclass, Fixed-effect model.

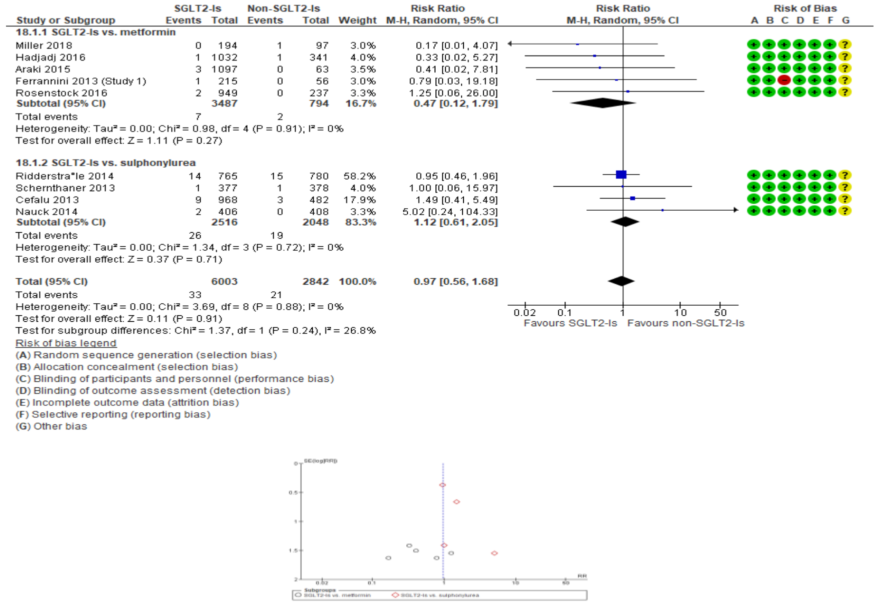

Figure S28. Forest and funnel plot of SGLT2-Is and stroke by AHGs subclass, Random-effect model.

Table S19. GRADE evidence profile of SGLT2-Is and stroke by AHGs subclass

Certainty assessment							Summary of findings				
№ of participants (trials)	Risk of bias	Inconsistency	Indirectness	Imprecision	Publication bias	Overall certainty of evidence	Study event rates (%)		Relative effect (95% CI)	Anticipated absolute effects	
							With controls	With SGLT2-Is		Risk with controls	Risk difference with SGLT2-Is
**Risk of stroke in T2DM by SGLT2-Is vs. AHGs subclass (follow-up: range 12-156 weeks)**											
8845 (9 RCTs)	serious[a]	not serious	not serious	serious[b]	none	⊕⊕◯◯ LOW	21/2842 (0.7%)	33/6003 (0.5%)	RR 1.00 (0.59 to 1.71)	1 per 100	0 fewer per 100 (from 0 fewer to 1 more)
**Metformin (follow-up: range 12-78 weeks)**											
4281 (5 RCTs)	serious[a]	not serious	not serious	serious[c]	none	⊕⊕◯◯ LOW	2/794 (0.3%)	7/3487 (0.2%)	RR 0.47 (0.13 to 1.69)	0 per 100	0 fewer per 100 (from 0 fewer to 0 fewer)
**Sulfonylurea (follow-up: range 52-156 weeks)**											
4564 (4 RCTs)	not serious	not serious	not serious	serious[d]	none	⊕⊕⊕◯ MODERATE	19/2048 (0.9%)	26/2516 (1.0%)	RR 1.16 (0.64 to 2.10)	1 per 100	0 fewer per 100 (from 0 fewer to 1 more)

Abbreviations: **AHGs,** anti-hyperglycaemic agents; **CI,** confidence interval; **RCTs,** randomised controlled trials; **RR,** risk ratio; **SGLT2-Is,** sodium-glucose cotransporter-2 inhibitors.

Note: The GRADE scores were from the fixed-effect model.

GRADE evidence

a. A trial with small weight SGLT2-I empagliflozin vs. metformin (3.0%) with the overall EE rated as having a high risk of bias due to lack of blinding (open-label design) out of nine trials.

b. The overall imprecision was precise, with no significant effect size difference (P=0.99). The CIs overlapped and were narrow. The 95% CI was consistent with the possibility of a benefit not exceeding the MID, including only 54 events with a large sample size.

c. The overall imprecision was precise, with no significant effect size difference (P=0.25). However, all trials reported overlapping CIs, in which two trials reported wide CIs. The 95%

CI was consistent with the possibility of a benefit not exceeding the MID, including only nine events with large sample sizes.

d. The overall imprecision was precise, with no significant effect size difference (P=0.62). However, all trials reported overlapping CIs, in which two trials reported wide CIs. The 95% CI was consistent with the possibility of a benefit not exceeding the MID, including only 45 events with a large sample size.

In summary, the overall certainty of the pooled EE had low imprecision without a significant effect size difference in the level of evidence (P=0.99). However, the majority of trials reported overlapping CIs, in which four trials reported wide CIs. All trials crossed the line of no difference (1). The 95% CI was consistent with the possibility of a benefit not exceeding the MID, including a total of 54 events with a large sample size (8,845). There was no evidence of inconsistency, statistically significant heterogeneity (P=0.88; I^2=0%), or subgroup difference (P=0.21; I^2=36.3%). The majority of trials had low risk of bias (97.0%). There was no evidence of detection bias. However, the magnitude of the impact of some trials that suffered from limitations likely resulted in biased assessment of the intervention effect due to performance and selection bias (one trial with an open-label design out of nine). The indirectness of the trials indicated a high level of evidence, with an individual trial PICOS element aligned closely to the review PICOS. Finally, there was no evidence of reporting bias; all included trials were within the two boundaries of the pyramid.

GLP1-RAs and stroke by AHG subclass

Use of GLP1-RAs did not decrease stroke risk (RR, 0.91; 95% CI, 0.52−1.57; P=0.73) compared to other classes of AHGs using fixed- and random-effect models (Figure S29 and Figure S30). The GRADE scores were low (Table S20).

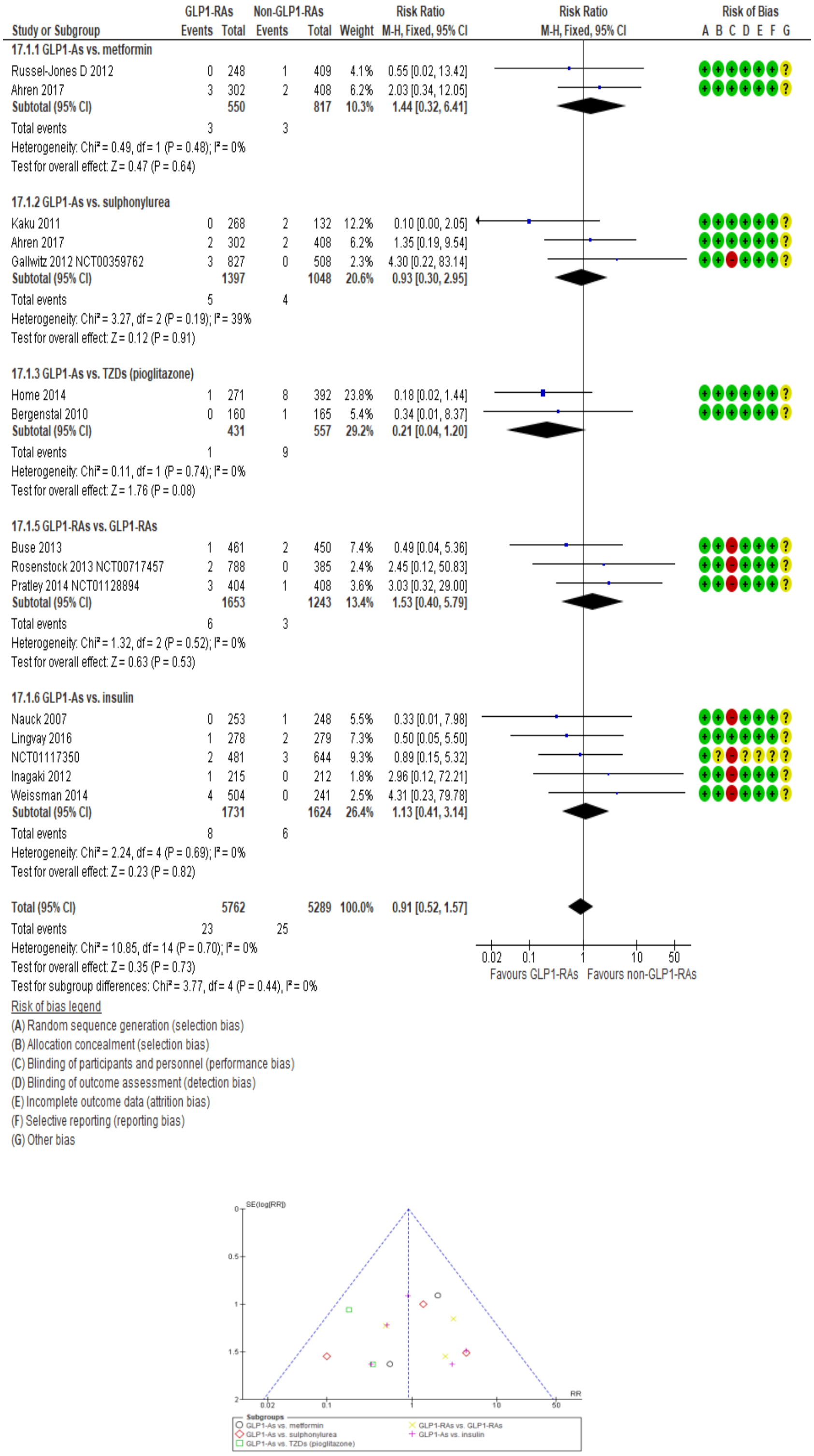

Figure S29. Forest and funnel plot of GLP1-RAs and stroke by AHGs subclass, Fixed-effect model.

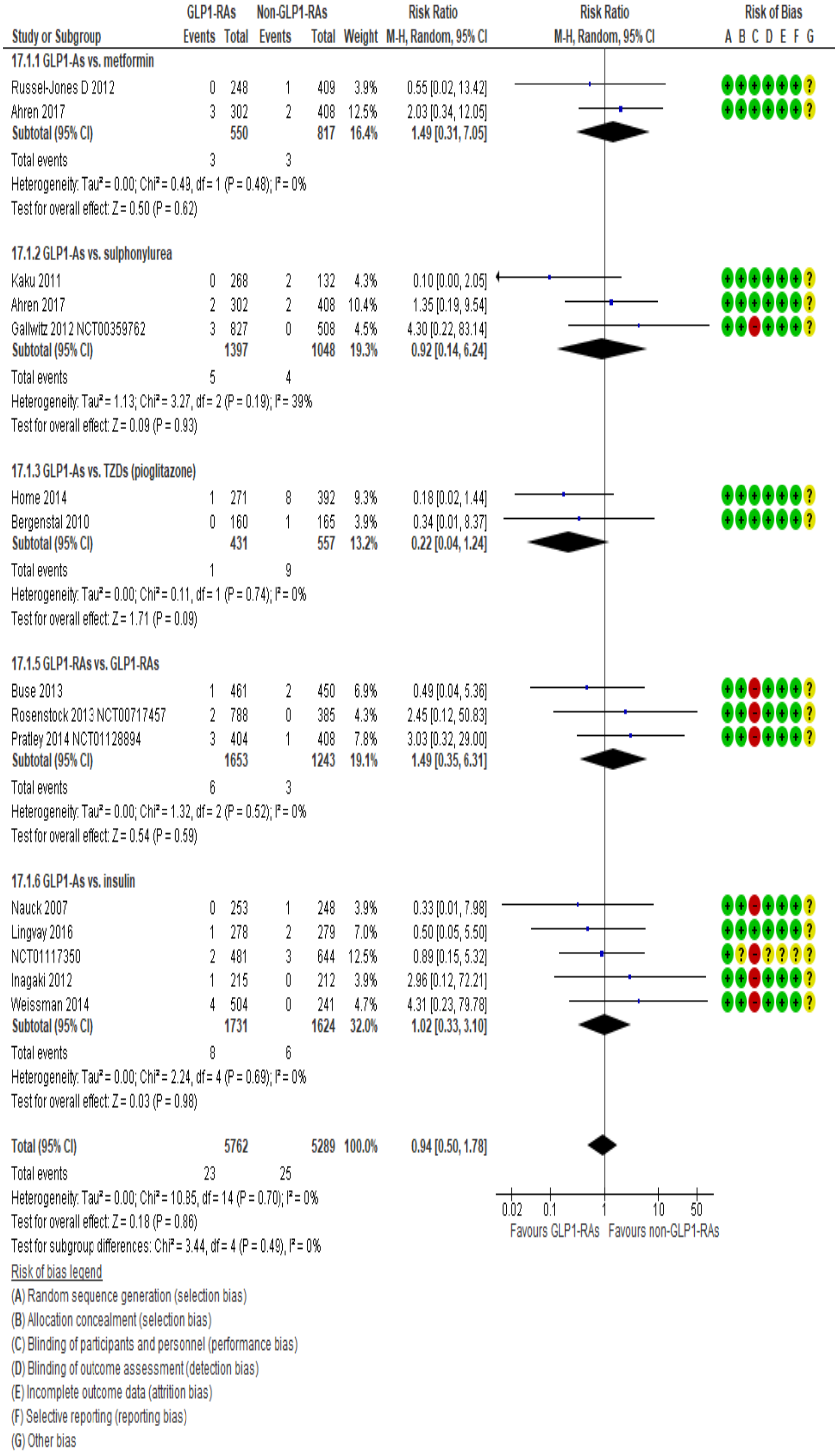

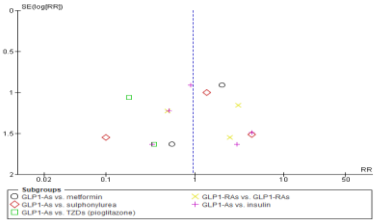

Figure S30. Forest and funnel plot of GLP1-RAs and stroke by AHGs subclass, Random-effect model.

Table S20. GRADE evidence profile of GLP1-RAs and stroke by AHGs subclass

Certainty assessment							Summary of findings				
№ of participants (trials)	Risk of bias	Inconsistency	Indirectness	Imprecision	Publication bias	Overall certainty of evidence	Study event rates (%)		Relative effect (95% CI)	Anticipated absolute effects	
							With controls	With GLP1-RAs		Risk with controls	Risk difference with GLP1-RAs
**Risk of stroke in T2DM by GLP1-RAs vs. AHGs subclass (follow-up: range 26-235 weeks)**											
11051 (15 RCTs)	serious[a]	not serious	not serious	serious[b]	none	⊕⊕◯◯ LOW	25/5289 (0.5%)	23/5762 (0.4%)	RR 0.91 (0.52 to 1.57)	0 per 100	0 fewer per 100 (from 0 fewer to 0 fewer)
**Metformin (follow-up: range 36-156 weeks)**											
1367 (2 RCTs)	not serious	not serious	not serious	very serious[c]	none	⊕⊕◯◯ LOW	3/817 (0.4%)	3/550 (0.5%)	RR 1.44 (0.32 to 6.41)	0 per 100	0 fewer per 100 (from 0 fewer to 2 more)
**Sulfonylurea (follow-up: range 36-235 weeks)**											
2445 (3 RCTs)	serious[a]	not serious	not serious	very serious[d]	none	⊕◯◯◯ VERY LOW	4/1048 (0.4%)	5/1397 (0.4%)	RR 0.93 (0.30 to 2.95)	0 per 100	0 fewer per 100 (from 0 fewer to 1 more)
**TZDs pioglitazone (follow-up: mean 156 weeks)**											
988 (2 RCTs)	not serious	not serious	not serious	very serious[e]	none	⊕⊕◯◯ LOW	9/557 (1.6%)	1/431 (0.2%)	RR 0.21 (0.04 to 1.20)	2 per 100	1 fewer per 100 (from 2 fewer to 0 fewer)
**GLP1-RAs (follow-up: range 26-104 weeks)**											
2896 (3 RCTs)	not serious	not serious	not serious	very serious[f]	none	⊕⊕◯◯ LOW	3/1243 (0.2%)	6/1653 (0.4%)	RR 1.53 (0.40 to 5.79)	0 per 100	0 fewer per 100 (from 0 fewer to 1 more)
**Insulin (follow-up: range 26-156 weeks)**											
3355 (5 RCTs)	serious[a]	not serious	not serious	very serious[g]	none	⊕◯◯◯ VERY LOW	6/1624 (0.4%)	8/1731 (0.5%)	RR 1.13 (0.41 to 3.14)	0 per 100	0 fewer per 100 (from 0 fewer to 1 more)

Abbreviations: **AHGs,** anti-hyperglycaemic agents; **CI,** confidence interval; **GLP1-RAs,** glucagon-like peptide-1 receptor agonists; **RCTs,** randomised controlled trials; **RR,** risk ratio.

Note: The GRADE scores were from the fixed-effect model.

GRADE evidence

a. Eight trials with small weight GLP1-RAs vs. AHGs (9.3%, 7.4%, 5.5%, 3.6%, 2.5%, 2.4%, 2.3%, and 1.8%) with the overall EE rated as high risk of bias due to lack of blinding (open-label design) out of 15 trials, one of which was an unpublished RCT.

b. The overall imprecision was precise, with no significant effect size difference (P=0.73). However, all trials reported overlapping and narrow CIs. The 95% CI was consistent with the possibility of a benefit not exceeding the MID, including only 48 events with a large sample size.

c. The overall imprecision was precise, with no significant effect size difference (P=0.64). However, all trials reported overlapping CIs, in which two trials reported wide CIs. The 95% CI was consistent with the possibility of a benefit not exceeding the MID, including only six events with a small sample size (1,367).

d. The overall imprecision was precise, with no significant effect size difference (P=0.91). However, all trials reported overlapping CIs, in which one trial reported a wide CI. The 95% CI was consistent with the possibility of a benefit not exceeding the MID, including only nine events with a large sample size.

e. The overall imprecision was precise, with no significant effect size difference (P=0.08). However, all trials reported overlapping and narrow CIs. The 95% CI was consistent with the possibility of a benefit not exceeding the MID, including only 10 events with a small sample size (988).

f. The overall imprecision was precise, with no significant effect size difference (P=0.53). However, all trials reported overlapping CIs, in which two trials reported wide CIs. The 95% CI was consistent with the possibility of a benefit not exceeding the MID, including only nine events with a large sample size.

g. The overall imprecision was precise, with no significant effect size difference (P=0.82). However, all trials reported overlapping CIs, in which two trials reported wide CIs. The 95% CI was consistent with the possibility of a benefit not exceeding the MID, including only 14 events with a large sample size.

In summary, the overall certainty of the pooled EE had low imprecision without a significant effect size difference in the level of evidence (P=0.73). However, the majority of trials reported overlapping CIs, in which seven trials reported wide CIs. All trials crossed the line of no difference (1). The 95% CI was consistent with the possibility of a benefit not exceeding the MID, including a total of 48 events with a large sample size. There was no evidence of inconsistency, statistically significant heterogeneity (P=0.70; I^2=0%), or subgroup difference (P=0.44; I^2=0%). The majority of trials were at low risk of bias (65.2%). There was no evidence of detection bias. However, the magnitude of the impact of some trials that suffered from limitations likely resulted in biased assessment of the intervention effect due to performance and selection bias (eight trials with open-label design out of 15, one of which was an unpublished RCT). The indirectness of the trials indicated a high level of evidence, with an individual trial PICOS element aligned closely to the review PICOS. Finally, there was no evidence of reporting bias; all included trials were within the two boundaries of the pyramid.

DPP4-Is and stroke by AHG subclass

Use of DPP4-Is decreased stroke risk (RR, 0.79; 95% CI, 0.63−0.99; P=0.04) versus sulfonylurea using fixed-effect model only. However, use of DDP4-Is did not decrease stroke risk (RR, 0.87; 95% CI, 0.72−1.04; P=0.13) compared to other classes of AHGs using fixed- and random-effect models (Figure S31 and Figure S32). The GRADE scores were moderate (Table S21).

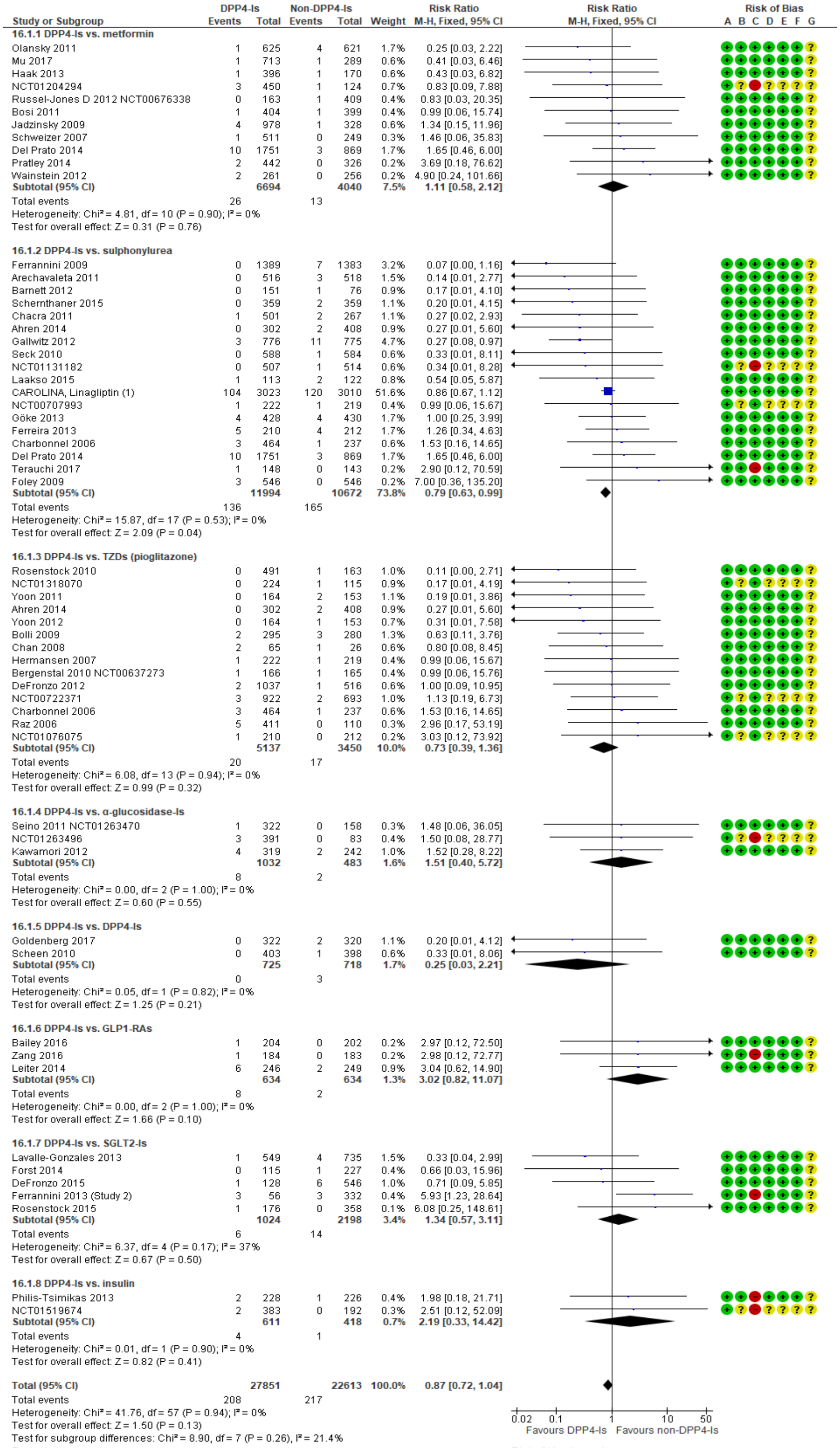

Study or Subgroup	DPP4-Is Events	DPP4-Is Total	Non-DPP4-Is Events	Non-DPP4-Is Total	Weight	Risk Ratio M-H, Fixed, 95% CI
**16.1.1 DPP4-Is vs. metformin**						
Olansky 2011	1	625	4	621	1.7%	0.25 [0.03, 2.22]
Mu 2017	1	713	1	289	0.6%	0.41 [0.03, 6.46]
Haak 2013	1	396	1	170	0.6%	0.43 [0.03, 6.82]
NCT01204294	3	450	1	124	0.7%	0.83 [0.09, 7.88]
Russel-Jones D 2012 NCT00676338	0	163	1	409	0.4%	0.83 [0.03, 20.35]
Bosi 2011	1	404	1	399	0.4%	0.99 [0.06, 15.74]
Jadzinsky 2009	4	978	1	328	0.6%	1.34 [0.15, 11.96]
Schweizer 2007	1	511	0	249	0.3%	1.46 [0.06, 35.83]
Del Prato 2014	10	1751	3	869	1.7%	1.65 [0.46, 6.00]
Pratley 2014	2	442	0	326	0.2%	3.69 [0.18, 76.62]
Wainstein 2012	2	261	0	256	0.2%	4.90 [0.24, 101.66]
**Subtotal (95% CI)**		**6694**		**4040**	**7.5%**	**1.11 [0.58, 2.12]**
Total events	26		13			
Heterogeneity: Chi² = 4.81, df = 10 (P = 0.90); I² = 0%						
Test for overall effect: Z = 0.31 (P = 0.76)						
**16.1.2 DPP4-Is vs. sulphonylurea**						
Ferrannini 2009	0	1389	7	1383	3.2%	0.07 [0.00, 1.16]
Arechavaleta 2011	0	516	3	518	1.5%	0.14 [0.01, 2.77]
Barnett 2012	0	151	1	76	0.9%	0.17 [0.01, 4.10]
Schernthaner 2015	0	359	2	359	1.1%	0.20 [0.01, 4.15]
Chacra 2011	1	501	2	267	1.1%	0.27 [0.02, 2.93]
Ahren 2014	0	302	2	408	0.9%	0.27 [0.01, 5.60]
Gallwitz 2012	3	776	11	775	4.7%	0.27 [0.08, 0.97]
Seck 2010	0	588	1	584	0.6%	0.33 [0.01, 8.11]
NCT01131182	0	507	1	514	0.6%	0.34 [0.01, 8.28]
Laakso 2015	1	113	2	122	0.8%	0.54 [0.05, 5.87]
CAROLINA, Linagliptin (1)	104	3023	120	3010	51.6%	0.86 [0.67, 1.12]
NCT00707993	1	222	1	219	0.4%	0.99 [0.06, 15.67]
Göke 2013	4	428	4	430	1.7%	1.00 [0.25, 3.99]
Ferreira 2013	5	210	4	212	1.7%	1.26 [0.34, 4.63]
Charbonnel 2006	3	464	1	237	0.6%	1.53 [0.16, 14.65]
Del Prato 2014	10	1751	3	869	1.7%	1.65 [0.46, 6.00]
Terauchi 2017	1	148	0	143	0.2%	2.90 [0.12, 70.59]
Foley 2009	3	546	0	546	0.2%	7.00 [0.36, 135.20]
**Subtotal (95% CI)**		**11994**		**10672**	**73.8%**	**0.79 [0.63, 0.99]**
Total events	136		165			
Heterogeneity: Chi² = 15.87, df = 17 (P = 0.53); I² = 0%						
Test for overall effect: Z = 2.09 (P = 0.04)						
**16.1.3 DPP4-Is vs. TZDs (pioglitazone)**						
Rosenstock 2010	0	491	1	163	1.0%	0.11 [0.00, 2.71]
NCT01318070	0	224	1	115	0.9%	0.17 [0.01, 4.19]
Yoon 2011	0	164	2	153	1.1%	0.19 [0.01, 3.86]
Ahren 2014	0	302	2	408	0.9%	0.27 [0.01, 5.60]
Yoon 2012	0	164	1	153	0.7%	0.31 [0.01, 7.58]
Bolli 2009	2	295	3	280	1.3%	0.63 [0.11, 3.76]
Chan 2008	2	65	1	26	0.6%	0.80 [0.08, 8.45]
Hermansen 2007	1	222	1	219	0.4%	0.99 [0.06, 15.67]
Bergenstal 2010 NCT00637273	1	166	1	165	0.4%	0.99 [0.06, 15.76]
DeFronzo 2012	2	1037	1	516	0.6%	1.00 [0.09, 10.95]
NCT00722371	3	922	2	693	1.0%	1.13 [0.19, 6.73]
Charbonnel 2006	3	464	1	237	0.6%	1.53 [0.16, 14.65]
Raz 2006	5	411	0	110	0.3%	2.96 [0.17, 53.19]
NCT01076075	1	210	0	212	0.2%	3.03 [0.12, 73.92]
**Subtotal (95% CI)**		**5137**		**3450**	**10.0%**	**0.73 [0.39, 1.36]**
Total events	20		17			
Heterogeneity: Chi² = 6.08, df = 13 (P = 0.94); I² = 0%						
Test for overall effect: Z = 0.99 (P = 0.32)						
**16.1.4 DPP4-Is vs. α-glucosidase-Is**						
Seino 2011 NCT01263470	1	322	0	158	0.3%	1.48 [0.06, 36.05]
NCT01263496	3	391	0	83	0.4%	1.50 [0.08, 28.77]
Kawamori 2012	4	319	2	242	1.0%	1.52 [0.28, 8.22]
**Subtotal (95% CI)**		**1032**		**483**	**1.6%**	**1.51 [0.40, 5.72]**
Total events	8		2			
Heterogeneity: Chi² = 0.00, df = 2 (P = 1.00); I² = 0%						
Test for overall effect: Z = 0.60 (P = 0.55)						
**16.1.5 DPP4-Is vs. DPP4-Is**						
Goldenberg 2017	0	322	2	320	1.1%	0.20 [0.01, 4.12]
Scheen 2010	0	403	1	398	0.6%	0.33 [0.01, 8.06]
**Subtotal (95% CI)**		**725**		**718**	**1.7%**	**0.25 [0.03, 2.21]**
Total events	0		3			
Heterogeneity: Chi² = 0.05, df = 1 (P = 0.82); I² = 0%						
Test for overall effect: Z = 1.25 (P = 0.21)						
**16.1.6 DPP4-Is vs. GLP1-RAs**						
Bailey 2016	1	204	0	202	0.2%	2.97 [0.12, 72.50]
Zang 2016	1	184	0	183	0.2%	2.98 [0.12, 72.77]
Leiter 2014	6	246	2	249	0.9%	3.04 [0.62, 14.90]
**Subtotal (95% CI)**		**634**		**634**	**1.3%**	**3.02 [0.82, 11.07]**
Total events	8		2			
Heterogeneity: Chi² = 0.00, df = 2 (P = 1.00); I² = 0%						
Test for overall effect: Z = 1.66 (P = 0.10)						
**16.1.7 DPP4-Is vs. SGLT2-Is**						
Lavalle-Gonzales 2013	1	549	4	735	1.5%	0.33 [0.04, 2.99]
Forst 2014	0	115	1	227	0.4%	0.66 [0.03, 15.96]
DeFronzo 2015	1	128	6	546	1.0%	0.71 [0.09, 5.85]
Ferrannini 2013 (Study 2)	3	56	3	332	0.4%	5.93 [1.23, 28.64]
Rosenstock 2015	1	176	0	358	0.1%	6.08 [0.25, 148.61]
**Subtotal (95% CI)**		**1024**		**2198**	**3.4%**	**1.34 [0.57, 3.11]**
Total events	6		14			
Heterogeneity: Chi² = 6.37, df = 4 (P = 0.17); I² = 37%						
Test for overall effect: Z = 0.67 (P = 0.50)						
**16.1.8 DPP4-Is vs. insulin**						
Philis-Tsimikas 2013	2	228	1	226	0.4%	1.98 [0.18, 21.71]
NCT01519674	2	383	0	192	0.3%	2.51 [0.12, 52.09]
**Subtotal (95% CI)**		**611**		**418**	**0.7%**	**2.19 [0.33, 14.42]**
Total events	4		1			
Heterogeneity: Chi² = 0.01, df = 1 (P = 0.90); I² = 0%						
Test for overall effect: Z = 0.82 (P = 0.41)						
**Total (95% CI)**		**27851**		**22613**	**100.0%**	**0.87 [0.72, 1.04]**
Total events	208		217			
Heterogeneity: Chi² = 41.76, df = 57 (P = 0.94); I² = 0%						
Test for overall effect: Z = 1.50 (P = 0.13)						
Test for subgroup differences: Chi² = 8.90, df = 7 (P = 0.26), I² = 21.4%						

Footnotes
(1) The Cardiovascular Outcome Study of Linagliptin vs Glimepiride in Type 2 Diabetes

Risk of bias legend
(A) Random sequence generation (selection bias)
(B) Allocation concealment (selection bias)
(C) Blinding of participants and personnel (performance bias)
(D) Blinding of outcome assessment (detection bias)
(E) Incomplete outcome data (attrition bias)
(F) Selective reporting (reporting bias)
(G) Other bias

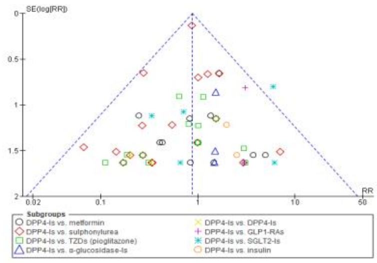

Figure S31. Forest and funnel plot of DPP4-Is and stroke by AHGs subclass, Fixed-effect model.

Study or Subgroup	DPP4-Is Events	DPP4-Is Total	Non-DPP4-Is Events	Non-DPP4-Is Total	Weight	Risk Ratio M-H, Random, 95% CI
**16.1.1 DPP4-Is vs. metformin**						
Olansky 2011	1	625	4	621	0.8%	0.25 [0.03, 2.22]
Mu 2017	1	713	1	289	0.5%	0.41 [0.03, 6.46]
Haak 2013	1	396	1	170	0.5%	0.43 [0.03, 6.82]
NCT01204294	3	450	1	124	0.8%	0.83 [0.09, 7.88]
Russel-Jones D 2012 NCT00676338	0	163	1	409	0.4%	0.83 [0.03, 20.35]
Bosi 2011	1	404	1	399	0.5%	0.99 [0.06, 15.74]
Jadzinsky 2009	4	978	1	328	0.8%	1.34 [0.15, 11.96]
Schweizer 2007	1	511	0	249	0.4%	1.46 [0.06, 35.83]
Del Prato 2014	10	1751	3	869	2.3%	1.65 [0.46, 6.00]
Pratley 2014	2	442	0	326	0.4%	3.69 [0.18, 76.62]
Wainstein 2012	2	261	0	256	0.4%	4.90 [0.24, 101.66]
**Subtotal (95% CI)**		**6694**		**4040**	**7.8%**	**1.07 [0.53, 2.17]**
Total events	26		13			
Heterogeneity: Tau² = 0.00; Chi² = 4.81, df = 10 (P = 0.90); I² = 0%						
Test for overall effect: Z = 0.19 (P = 0.85)						
**16.1.2 DPP4-Is vs. sulphonylurea**						
Ferrannini 2009	0	1389	7	1383	0.5%	0.07 [0.00, 1.16]
Arechavaleta 2011	0	516	3	518	0.4%	0.14 [0.01, 2.77]
Barnett 2012	0	151	1	76	0.4%	0.17 [0.01, 4.10]
Schernthaner 2015	0	359	2	359	0.4%	0.20 [0.01, 4.15]
Chacra 2011	1	501	2	267	0.7%	0.27 [0.02, 2.93]
Ahren 2014	0	302	2	408	0.4%	0.27 [0.01, 5.60]
Gallwitz 2012	3	776	11	775	2.4%	0.27 [0.08, 0.97]
Seck 2010	0	588	1	584	0.4%	0.33 [0.01, 8.11]
NCT01131182	0	507	1	514	0.4%	0.34 [0.01, 8.28]
Laakso 2015	1	113	2	122	0.7%	0.54 [0.05, 5.87]
CAROLINA, Linagliptin (1)	104	3023	120	3010	58.1%	0.86 [0.67, 1.12]
NCT00707993	1	222	1	219	0.5%	0.99 [0.06, 15.67]
Göke 2013	4	428	4	430	2.0%	1.00 [0.25, 3.99]
Ferreira 2013	5	210	4	212	2.3%	1.26 [0.34, 4.63]
Charbonnel 2006	3	464	1	237	0.8%	1.53 [0.16, 14.65]
Del Prato 2014	10	1751	3	869	2.3%	1.65 [0.46, 6.00]
Terauchi 2017	1	148	0	143	0.4%	2.90 [0.12, 70.59]
Foley 2009	3	546	0	546	0.4%	7.00 [0.36, 135.20]
**Subtotal (95% CI)**		**11994**		**10672**	**73.5%**	**0.82 [0.65, 1.03]**
Total events	136		165			
Heterogeneity: Tau² = 0.00; Chi² = 15.87, df = 17 (P = 0.53); I² = 0%						
Test for overall effect: Z = 1.69 (P = 0.09)						
**16.1.3 DPP4-Is vs. TZDs (pioglitazone)**						
Rosenstock 2010	0	491	1	163	0.4%	0.11 [0.00, 2.71]
NCT01318070	0	224	1	115	0.4%	0.17 [0.01, 4.19]
Yoon 2011	0	164	2	153	0.4%	0.19 [0.01, 3.86]
Ahren 2014	0	302	2	408	0.4%	0.27 [0.01, 5.60]
Yoon 2012	0	164	1	153	0.4%	0.31 [0.01, 7.58]
Bolli 2009	2	295	3	280	1.2%	0.63 [0.11, 3.76]
Chan 2008	2	65	1	26	0.7%	0.80 [0.08, 8.45]
Hermansen 2007	1	222	1	219	0.5%	0.99 [0.06, 15.67]
Bergenstal 2010 NCT00637273	1	166	1	165	0.5%	0.99 [0.06, 15.76]
DeFronzo 2012	2	1037	1	516	0.7%	1.00 [0.09, 10.95]
NCT00722371	3	922	2	693	1.2%	1.13 [0.19, 6.73]
Charbonnel 2006	3	464	1	237	0.8%	1.53 [0.16, 14.65]
Raz 2006	5	411	0	110	0.5%	2.96 [0.17, 53.19]
NCT01076075	1	210	0	212	0.4%	3.03 [0.12, 73.92]
**Subtotal (95% CI)**		**5137**		**3450**	**8.4%**	**0.74 [0.37, 1.46]**
Total events	20		17			
Heterogeneity: Tau² = 0.00; Chi² = 6.08, df = 13 (P = 0.94); I² = 0%						
Test for overall effect: Z = 0.87 (P = 0.38)						
**16.1.4 DPP4-Is vs. α-glucosidase-Is**						
Seino 2011 NCT01263470	1	322	0	158	0.4%	1.48 [0.06, 36.05]
NCT01263496	3	391	0	83	0.4%	1.50 [0.08, 28.77]
Kawamori 2012	4	319	2	242	1.4%	1.52 [0.28, 8.22]
**Subtotal (95% CI)**		**1032**		**483**	**2.2%**	**1.51 [0.40, 5.71]**
Total events	8		2			
Heterogeneity: Tau² = 0.00; Chi² = 0.00, df = 2 (P = 1.00); I² = 0%						
Test for overall effect: Z = 0.60 (P = 0.55)						
**16.1.5 DPP4-Is vs. DPP4-Is**						
Goldenberg 2017	0	322	2	320	0.4%	0.20 [0.01, 4.12]
Scheen 2010	0	403	1	398	0.4%	0.33 [0.01, 8.06]
**Subtotal (95% CI)**		**725**		**718**	**0.8%**	**0.25 [0.03, 2.28]**
Total events	0		3			
Heterogeneity: Tau² = 0.00; Chi² = 0.05, df = 1 (P = 0.82); I² = 0%						
Test for overall effect: Z = 1.23 (P = 0.22)						
**16.1.6 DPP4-Is vs. GLP1-RAs**						
Bailey 2016	1	204	0	202	0.4%	2.97 [0.12, 72.50]
Zang 2016	1	184	0	183	0.4%	2.98 [0.12, 72.77]
Leiter 2014	6	246	2	249	1.5%	3.04 [0.62, 14.90]
**Subtotal (95% CI)**		**634**		**634**	**2.3%**	**3.02 [0.82, 11.07]**
Total events	8		2			
Heterogeneity: Tau² = 0.00; Chi² = 0.00, df = 2 (P = 1.00); I² = 0%						
Test for overall effect: Z = 1.66 (P = 0.10)						
**16.1.7 DPP4-Is vs. SGLT2-Is**						
Lavalle-Gonzales 2013	1	549	4	735	0.8%	0.33 [0.04, 2.99]
Forst 2014	0	115	1	227	0.4%	0.66 [0.03, 15.96]
DeFronzo 2015	1	128	6	546	0.9%	0.71 [0.09, 5.85]
Ferrannini 2013 (Study 2)	3	56	3	332	1.6%	5.93 [1.23, 28.64]
Rosenstock 2015	1	176	0	358	0.4%	6.08 [0.25, 148.61]
**Subtotal (95% CI)**		**1024**		**2198**	**4.0%**	**1.50 [0.41, 5.52]**
Total events	6		14			
Heterogeneity: Tau² = 0.81; Chi² = 6.37, df = 4 (P = 0.17); I² = 37%						
Test for overall effect: Z = 0.61 (P = 0.54)						
**16.1.8 DPP4-Is vs. insulin**						
Philis-Tsimikas 2013	2	228	1	226	0.7%	1.98 [0.18, 21.71]
NCT01519674	2	383	0	192	0.4%	2.51 [0.12, 52.09]
**Subtotal (95% CI)**		**611**		**418**	**1.1%**	**2.17 [0.33, 14.21]**
Total events	4		1			
Heterogeneity: Tau² = 0.00; Chi² = 0.01, df = 1 (P = 0.90); I² = 0%						
Test for overall effect: Z = 0.81 (P = 0.42)						
**Total (95% CI)**		**27851**		**22613**	**100.0%**	**0.89 [0.73, 1.09]**
Total events	208		217			
Heterogeneity: Tau² = 0.00; Chi² = 41.76, df = 57 (P = 0.94); I² = 0%						
Test for overall effect: Z = 1.12 (P = 0.26)						
Test for subgroup differences: Chi² = 7.76, df = 7 (P = 0.35), I² = 9.8%						

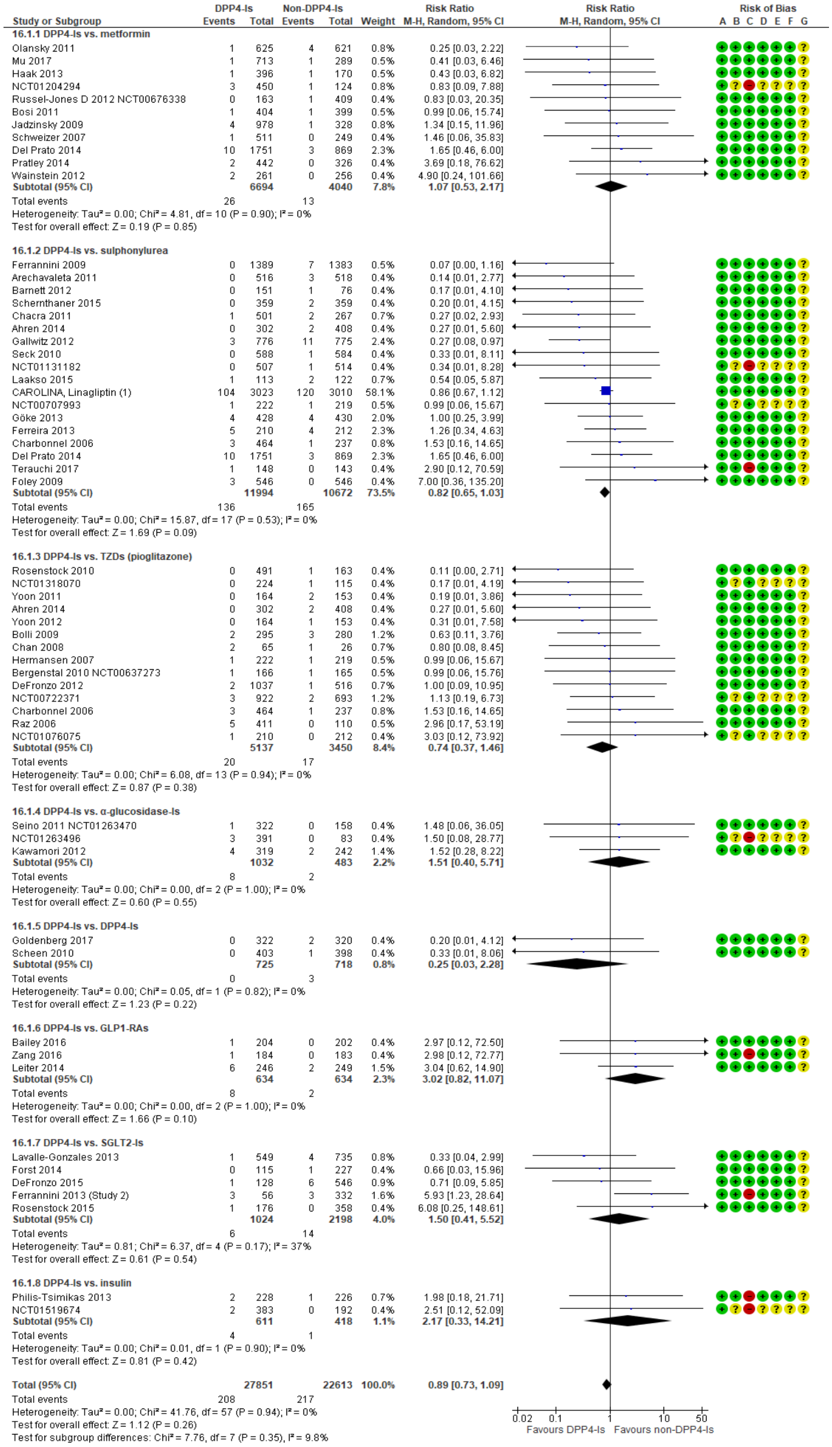

Footnotes
(1) The Cardiovascular Outcome Study of Linagliptin vs Glimepiride in Type 2 Diabetes

Risk of bias legend
(A) Random sequence generation (selection bias)
(B) Allocation concealment (selection bias)
(C) Blinding of participants and personnel (performance bias)
(D) Blinding of outcome assessment (detection bias)
(E) Incomplete outcome data (attrition bias)
(F) Selective reporting (reporting bias)
(G) Other bias

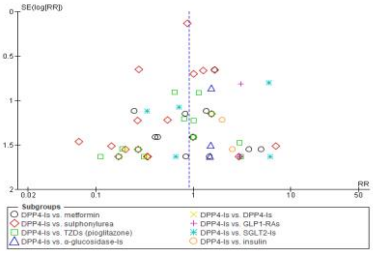

Figure S32. Forest and funnel plot of DPP4-Is and stroke by AHGs subclass, Random-effect model.

Table S21. GRADE evidence profile of DPP4-Is and stroke by AHGs subclass

Certainty assessment							Summary of findings				
№ of participants (trials)	Risk of bias	Inconsistency	Indirectness	Imprecision	Publication bias	Overall certainty of evidence	Study event rates (%)		Relative effect (95% CI)	Anticipated absolute effects	
							With controls	With DPP4-Is		Risk with controls	Risk difference with DPP4-Is
**Risk of stroke in T2DM by DPP4-Is vs. AHGs subclass (follow-up: range 4-432 weeks)**											
50464 (55 RCTs)	serious[a]	not serious	not serious	not serious	none	⊕⊕⊕◯ MODERATE	217/22613 (1.0%)	208/27851 (0.7%)	RR 0.87 (0.72 to 1.04)	1 per 100	0 fewer per 100 (from 0 fewer to 0 fewer)
**Metformin (follow-up: range 24-104 weeks)**											
10734 (11 RCTs)	serious[a]	not serious	not serious	very serious[b]	none	⊕◯◯◯ VERY LOW	13/4040 (0.3%)	26/6694 (0.4%)	RR 1.11 (0.58 to 2.12)	0 per 100	0 fewer per 100 (from 0 fewer to 0 fewer)
**Sulfonylurea (follow-up: range 4-432 weeks)**											
22666 (18 RCTs)	serious[a]	not serious	not serious	serious[c]	none	⊕⊕◯◯ LOW	165/10672 (1.5%)	136/11994 (1.1%)	RR 0.79 (0.63 to 0.99)	2 per 100	0 fewer per 100 (from 1 fewer to 0 fewer)
**TZDs pioglitazone (follow-up: range 12-104 weeks)**											
8587 (14 RCTs)	not serious	not serious	not serious	very serious[d]	none	⊕⊕◯◯ LOW	17/3450 (0.5%)	20/5137 (0.4%)	RR 0.73 (0.39 to 1.36)	0 per 100	0 fewer per 100 (from 0 fewer to 0 fewer)
**α-glucosidase-Is (follow-up: range 52-126 weeks)**											
1515 (3 RCTs)	serious[a]	not serious	not serious	very serious[e]	none	⊕◯◯◯ VERY LOW	2/483 (0.4%)	8/1032 (0.8%)	RR 1.51 (0.40 to 5.72)	0 per 100	0 fewer per 100 (from 0 fewer to 2 more)
**DPP4-Is (follow-up: range 18-24 weeks)**											
1443 (2 RCTs)	serious[a]	not serious	not serious	very serious[f]	none	⊕◯◯◯ VERY LOW	3/718 (0.4%)	0/725 (0.0%)	RR 0.25 (0.03 to 2.21)	0 per 100	0 fewer per 100 (from 0 fewer to 1 more)

Certainty assessment							Summary of findings				
**GLP1-RAs (follow-up: range 26-52 weeks)**											
1268 (3 RCTs)	serious[a]	not serious	not serious	very serious[g]	none	⨁◯◯◯ VERY LOW	2/634 (0.3%)	8/634 (1.3%)	RR 3.02 (0.82 to 11.07)	0 per 100	1 more per 100 (from 0 fewer to 3 more)
**SGLT2-Is (follow-up: range 24-52 weeks)**											
3222 (5 RCTs)	serious[a]	not serious	not serious	very serious[h]	none	⨁◯◯◯ VERY LOW	14/2198 (0.6%)	6/1024 (0.6%)	RR 1.34 (0.57 to 3.11)	1 per 100	0 fewer per 100 (from 0 fewer to 1 more)
**Insulin (follow-up: range 12-26 weeks)**											
1029 (2 RCTs)	serious[a]	not serious	not serious	very serious[i]	none	⨁◯◯◯ VERY LOW	1/418 (0.2%)	4/611 (0.7%)	RR 2.19 (0.33 to 14.42)	0 per 100	0 fewer per 100 (from 0 fewer to 3 more)

Abbreviations: **AHGs,** anti-hyperglycaemic agents; **CI,** confidence interval; **DPP4-Is,** dipeptidyl peptidase-4 inhibitors; **GLP1-RAs,** glucagon-like peptide-1 receptor agonists; **RCTs,** randomised controlled trials; **RR,** risk ratio; **SGLT2-Is,** sodium-glucose cotransporter-2 inhibitors.

Note: The GRADE scores were from the fixed-effect model.

GRADE evidence

a. Eight trials with small weight DPP4-Is vs. AHGs (0.7%, 0.6%, 0.4%, 0.4%, 0.4%, 0.3%, 0.2%, and 0.2%) with the overall EE rated as high risk of bias due to lack of blinding (open-label design) out of 55 trials, four of which were unpublished RCTs.

b. The overall imprecision was precise, with no significant effect size difference (P=0.76). However, all trials reported overlapping CIs, in which six trials reported wide CIs. The 95% CI was consistent with the possibility of a benefit not exceeding the MID, including only 39 events with a large sample size.

c. The overall imprecision was precise, with a significant effect size difference (P=0.04). However, all trials reported overlapping CIs, in which four trials reported wide CIs. The 95% CI was consistent with the possibility of a benefit not exceeding the MID, including only 301 events with a large sample size.

d. The overall imprecision was precise, with no significant effect size difference (P=0.32). However, all trials reported overlapping CIs, in which five trials reported a wide CI. The 95% CI was consistent with the possibility of a large benefit exceeding the MID, including only 37 events with a large sample size.

e. The overall imprecision was precise, with no significant effect size difference (P=0.55). However, all trials reported overlapping CIs, in which two trials reported a wide CI. The 95% CI was consistent with the possibility of a benefit not exceeding the MID, including only 10 events with a small sample size (1,515).

f. The overall imprecision was precise, with no significant effect size difference (P=0.21). However, all trials reported overlapping and narrow CIs. The 95% CI was consistent with the possibility of a benefit not exceeding the MID, including only three events with a small sample size (1,443).

g. The overall imprecision was precise, with no significant effect size difference (P=0.10). However, all trials reported overlapping CIs, in which three trials reported a wide CI. The 95% CI was consistent with the possibility of a benefit not exceeding the MID, including only 10 events with a small sample size (1,268).

h. The overall imprecision was precise, with no significant effect size difference (P=0.50). However, all trials reported overlapping CIs, in which three trials reported a wide CI. The 95% CI was consistent with the possibility of a benefit not exceeding the MID, including only 20 events with a large sample size.

i. The overall imprecision was precise, with no significant effect size difference (P=0.41). However, all trials reported overlapping CIs, in which two trials reported a wide CI. The 95% CI was consistent with the possibility of a benefit not exceeding the MID, including only five events with a small sample size (1,029).

In summary, the overall certainty of the pooled EE had moderate imprecision without a significant effect size difference in the level of evidence (P=0.13). However, the majority of trials reported overlapping CIs, in which 25 trials reported wide CIs. In total, only one small trials, 4.7% and 0.4%, did not cross the line of no difference (1), in which there was a significant reduction of stroke in the direction of DPP4-Is favouring sulfonylurea and non-DPP4-Is (SGLT2-Is), respectively. The 95% CI was consistent with the possibility of a large benefit exceeding the MID, including a total of 425 events with a large sample size (50,464). There was no evidence of inconsistency, statistically significant heterogeneity (P=0.94; I^2=0%), or subgroup difference (P=0.26; I^2=21.4%). The majority of trials had low risk of bias (96.8%). There was no evidence of detection bias. However, the magnitude of the impact of some trials that suffered from limitations likely resulted in biased assessment of the intervention effect due to performance and selection bias (eight trials with open-label design out of 55). The indirectness of the trials indicated a high level of evidence, with an individual trial PICOS element aligned closely to the review PICOS. Finally, there was no evidence of reporting bias. However, one trial reported an outlier outside the pyramid edge due to multiple interventional groups, consequently resulting in a very wide CI.

References

1. DeFronzo R, Fleck P, Wilson C, Mekki Q. Efficacy and safety of the dipeptidyl peptidase-4 inhibitor alogliptin in patients with type 2 diabetes and inadequate glycemic control: a randomized, double-blind, placebo-controlled study. Diabetes Care 2008;31:2315-17.
2. Nauck M, Ellis G, Fleck P, Wilson C, Mekki Q. Efficacy and safety of adding the dipeptidyl peptidase-4 inhibitor alogliptin to metformin therapy in patients with type 2 diabetes inadequately controlled with metformin monotherapy: a multicentre, randomised, double-blind, placebo-controlled study. Int J Clin Pract 2009;63:46-55.
3. Pan C, Han P, Ji Q, et al. Efficacy and safety of alogliptin in patients with type 2 diabetes mellitus: a multicentre randomized double-blind placebo-controlled phase 3 study in mainland China, Taiwan, and Hong Kong. Diabetes 2017;9:386-95.
4. Pratley R, Reusch J, Fleck P, Wilson C, Mekki Q. Efficacy and safety of the dipeptidyl peptidase-4 inhibitor alogliptin added to pioglitazone in patients with type 2 diabetes: a randomized, double-blind, placebo-controlled study. Curr Med Res Opin 2009;25:2361-71.
5. White W, Cannon C, Heller S, et al. Alogliptin after acute coronary syndrome in patients with type 2 diabetes. N Engl J Med 2013;369:1327-35.
6. Rosenstock J, Rendell M, Gross J, Fleck P, Wilson C, Mekki Q. Alogliptin added to insulin therapy in patients with type 2 diabetes reduces HbA(1C) without causing weight gain or increased hypoglycaemia. Diabetes Obes Metab 2009;11:1145-52.
7. Seino Y, Hiroi S, Hirayama M, Kaku K. Efficacy and safety of alogliptin added to sulfonylurea in Japanese patients with type 2 diabetes: a randomized, double-blind, placebo-controlled trial with an open-label, long-term extension study. J Diabetes Investig 2012;3:517-25.

8. Pratley R, Kipnes M, Fleck P, Wilson C, Mekki Q. Efficacy and safety of the dipeptidyl peptidase-4 inhibitor alogliptin in patients with type 2 diabetes inadequately controlled by glyburide monotherapy. Diabetes Obes Metab 2009;11:167-76.

9. Seino Y, Fujita T, Hiroi S, Hirayama M, Kaku K. Alogliptin plus voglibose in Japanese patients with type 2 diabetes: a randomized, double-blind, placebo-controlled trial with an open-label, long-term extension. Curr Med Res Opin 2011;27:21-9.

10. DeFronzo R, Burant C, Fleck P, Wilson C, Mekki Q, Pratley R. Efficacy and tolerability of the DPP-4 inhibitor alogliptin combined with pioglitazone, in metformin-treated patients with type 2 diabetes. J Clin Endocrinol Metab 2012;97:1615-22.

11. Seino Y, Fujita T, Hiroi S, Hirayama M, Kaku K. Efficacy and safety of alogliptin in Japanese patients with type 2 diabetes mellitus: a randomized, double-blind, dose-ranging comparison with placebo, followed by a long-term extension study. Curr Med Res Opin 2011;27:1781-92.

12. ClinicalTrials.gov. Long-term safety study of alogliptin in participants with type 2 diabetes in Japan. (https://clinicaltrials.gov/ct2/show/NCT01263496). Accessed November 30, 2023.

13. Pratley R, Fleck P, Wilson C. Efficacy and safety of initial combination therapy with alogliptin plus metformin versus either as monotherapy in drug-naive patients with type 2 diabetes: a randomized, double-blind, 6-month study. Diabetes Obes Metab 2014;16:613-21.

14. Bosi E, Ellis G, Wilson C, Fleck P. Alogliptin as a third oral antidiabetic drug in patients with type 2 diabetes and inadequate glycaemic control on metformin and pioglitazone: a 52-week, randomized, double-blind, active-controlled, parallel-group study. Diabetes Obes Metab 2011;13:1088-96.

15. ClinicalTrials.gov. Efficacy and safety of alogliptin compared to glipizide in elderly diabetics. (https://clinicaltrials.gov/ct2/show/NCT00707993). Accessed November 30, 2023.

16. Del Prato S, Camisasca R, Wilson C, Fleck P. Durability of the efficacy and safety of alogliptin compared with glipizide in type 2 diabetes mellitus: a 2-year study. Diabetes Obes Metab 2014;16:1239-46.

17. Rosenstock J, Inzucchi S, Seufert J, Fleck P, Wilson C, Mekki Q. Initial combination therapy with alogliptin and pioglitazone in drug-naïve patients with type 2 diabetes. Diabetes Care 2010;33:2406-8.

18. ClinicalTrials.gov. Efficacy and safety of alogliptin used combination With thiazolidine in participants with type 2 diabetes in Japan. (https://clinicaltrials.gov/ct2/show/NCT01318070). Accessed November 30, 2023.

19. Mita T, Katakami N, Yoshii H, et al. Alogliptin, a dipeptidyl peptidase 4 inhibitor, prevents the progression of carotid atherosclerosis in patients with type 2 diabetes: the study of preventive effects of alogliptin on diabetic atherosclerosis (SPEAD-A). Diabetes Care 2016;39:139-48.

20. Pattzi S, Pitale M, Alpizar C, et al. Dutogliptin, a selective DPP4 inhibitor, improves glycaemic control in patients with type 2 diabetes: a 12-week, double-blind, randomized, placebo-controlled, multicentre trial. Diabetes Obes Metab 2010;12:348-55.

21. Garcia-Soria G, Gonzalez-Galvez G, Argoud G, et al. The dipeptidyl peptidase-4 inhibitor PHX1149 improves blood glucose control in patients with type 2 diabetes mellitus. Diabetes Obes Metab 2008;10:293-300.

22. Rosenstock J, Perkovic V, Johansen O, et al. Effect of linagliptin vs placebo on major cardiovascular events in adults with type 2 diabetes and high cardiovascular and renal risk: the CARMELINA randomized clinical trial. JAMA 2019;321:69-79.

23. Del Prato S, Barnett A, Huisman H, Neubacher D, Woerle H, Dugi K. Effect of linagliptin monotherapy on glycaemic control and markers of β-cell function in patients with inadequately controlled type 2 diabetes: a randomized controlled trial. Diabetes Obes Metab 2010;13:258-67.

24. Taskinen M, Rosenstock J, Tamminen I, et al. Safety and efficacy of linagliptin as add-on therapy to metformin in patients with type 2 diabetes: a randomized, double-blind, placebo-controlled study. Diabetes Obes Metab 2011;13:65-74.

25. Haak T, Meinicke T, Jones R, Weber S, von Eynatten M, Woerle H. Initial combination of linagliptin and metformin improves glycaemic control in type 2 diabetes: a randomized, double-blind, placebo-controlled study. Diabetes Obes Metab 2012;14:565-74.

26. Søfteland E, Meier J, Vangen B, Toorawa R, Maldonado-Lutomirsky M, Broedl U. Empagliflozin as add-on therapy in patients with type 2 diabetes inadequately controlled with linagliptin and metformin: a 24-week randomized, double-blind, parallel-group trial. Diabetes Care 2017;40:201-09.

27. Owens D, Swallow R, Dugi K, Woerle H. Efficacy and safety of linagliptin in persons with type 2 diabetes inadequately controlled by a combination of metformin and sulphonylurea: a 24-week randomized study. Diabet Med 2011;28:1352-61.

28. Bajaj M, Gilman R, Patel S, Kempthorne-Rawson J, Lewis-D'Agostino D, Woerle H. Linagliptin improved glycaemic control without weight gain or hypoglycaemia in patients with type 2 diabetes inadequately controlled by a combination of metformin

and pioglitazone: a 24-week randomized, double-blind study. Diabet Med 2014;31:1505-14.

29. Barnett A, Huisman H, Jones R, von Eynatten M, Patel S, Woerle H. Linagliptin for patients aged 70 years or older with type 2 diabetes inadequately controlled with common antidiabetes treatments: a randomised, double-blind, placebo-controlled trial. Lancet 2013;382:1413-23.

30. Lewin A, Arvay L, Liu D, Patel S, von Eynatten M, Woerle H. Efficacy and tolerability of linagliptin added to a sulfonylurea regimen in patients with inadequately controlled type 2 diabetes mellitus: an 18-week, multicenter, randomized, double-blind, placebo-controlled trial. Clin Ther 2012;34:1909-19.

31. Gomis R, Espadero R, Jones R, Woerle H, Dugi K. Efficacy and safety of initial combination therapy with linagliptin and pioglitazone in patients with inadequately controlled type 2 diabetes: a randomized, double-blind, placebo-controlled study. Diabetes Obes Metab 2011;13:653-61.

32. Yki-Järvinen H, Rosenstock J, Durán-Garcia S, et al. Effects of adding linagliptin to basal insulin regimen for inadequately controlled type 2 diabetes A ≥ 52-week randomized, double-blind study. Diabetes Care 2013;36:3875-81.

33. Wang W, Yang J, Yang G, et al. Efficacy and safety of linagliptin in Asian patients with type 2 diabetes mellitus inadequately controlled by metformin: A multinational 24-week, randomized clinical trial. Diabetes 2016;8:229-37.

34. ClinicalTrials.gov. Safety and efficacy in type 2 diabetic patients with severe chronic renal impairment, 5 mg BI 1356 (linagliptin) vs. placebo, insulin background inclusive. (https://clinicaltrials.gov/ct2/show/NCT00800683). Accessed November 30, 2023.

35. McGill J, Sloan L, Newman J, et al. Long-term efficacy and safety of linagliptin in patients with type 2 diabetes and severe renal impairment. Diabetes Care 2013;36:237-44.

36. Groop P, Cooper M, Perkovic V, et al. Linagliptin and its effects on hyperglycaemia and albuminuria in patients with type 2 diabetes and renal dysfunction: the randomized MARLINA-T2D trial. Diabetes Obes Metab 2017;19:1610-9.

37. Kawamori R, Inagaki N, Araki E, Watada H, Hayashi N, Horie Y. Linagliptin monotherapy provides superior glycaemic control versus placebo or voglibose with comparable safety in Japanese patients with type 2 diabetes: a randomized, placebo and active comparator-controlled, double-blind study. Diabetes Obes Metab 2012;14:348-57.

38. Mu Y, Pan C, Fan B, et al. Efficacy and safety of linagliptin/metformin single-pill combination as initial therapy in drug-naïve Asian patients with type 2 diabetes. Diabetes Res Clin Pract 2017;124:48-56.

39. Haak T, Meinicke T, Jones R, Weber S, von Eynatten M, Woerle H. Initial combination of linagliptin and metformin in patients with type 2 diabetes: efficacy and safety in a randomised, double-blind 1-year extension study. Int J Clin Pract 2013;67.

40. ClinicalTrials.gov. The safety and efficacy of linagliptin (5mg / once daily) given for 52 weeks as add-on therapy to patients with type 2 diabetes mellitus and insufficient glycaemic control despite diet, exercise, and treatment with one approved antidiabetic drug. (https://clinicaltrials.gov/ct2/show/NCT01204294). Accessed November 30, 2023.

41. Barnett A, Patel S, Harper R, et al. Linagliptin monotherapy in type 2 diabetes patients for whom metformin is inappropriate: an 18-week randomized, double-blind, placebo-

controlled phase III trial with a 34-week active-controlled extension. Diabetes Obes Metab 2012;14:1145-54.

42. Laakso M, Rosenstock J, Groop P, et al. Treatment with the dipeptidyl peptidase-4 inhibitor linagliptin or placebo followed by glimepiride in patients with type 2 diabetes with moderate to severe renal impairment: a 52-week, randomized, double-blind clinical trial. Diabetes Care 2015;38:15-7.

43. Gallwitz B, Rosenstock J, Rauch T, et al. 2-year efficacy and safety of linagliptin compared with glimepiride in patients with type 2 diabetes inadequately controlled on metformin: a randomised, double-blind, non-inferiority trial. Lancet 2012;380:475-483.

44. Rosenstock J, Kahn S, Johansen O, et al. Effect of linagliptin vs glimepiride on major adverse cardiovascular outcomes in patients with type 2 diabetes the CAROLINA randomized clinical trial. JAMA 2019;322:1155-66.

45. Gantz I, Chen M, Suryawanshi S, et al. A randomized, placebo-controlled study of the cardiovascular safety of the once-weekly DPP-4 inhibitor omarigliptin in patients with type 2 diabetes mellitus. Cardiovasc Diabetol 2017;16:112.

46. Goldenberg R, Gantz I, Andryuk P, et al. Randomized clinical trial comparing the efficacy and safety of treatment with the once-weekly dipeptidyl peptidase-4 (DPP-4) inhibitor omarigliptin or the once-daily DPP-4 inhibitor sitagliptin in patients with type 2 diabetes inadequately controlled on metformin monotherapy. Diabetes Obes Metab 2017;19:394-400.

47. Pan C, Yang W, Tou C, Gause-Nilsson I, Zhao J. Efficacy and safety of saxagliptin in drug-naive Asian patients with type 2 diabetes mellitus: a randomized controlled trial. Diabetes Metab Res Rev 2012;28:268-75.

48. Rosenstock J, Sankoh S, List J. Glucose-lowering activity of the dipeptidyl peptidase-4 inhibitor saxagliptin in drug-naive patients with type 2 diabetes. Diabetes Obes Metab 2008;10:376-86.

49. Kumar K, Jain S, Tou C, Schu¨tzer K-M. Saxagliptin as initial therapy in treatment-naive Indian adults with type 2 diabetes mellitus inadequately controlled with diet and exercise alone: a randomized, double-blind, placebo-controlled, phase IIIb clinical study. Int J Diabetes Dev Ctries 2014;34:201-09.

50. Frederich R, McNeill R, Berglind N, Fleming D, Chen R. The efficacy and safety of the dipeptidyl peptidase-4 inhibitor saxagliptin in treatment-naïve patients with type 2 diabetes mellitus: a randomized controlled trial. Diabetology & Metabolic Syndrome 2012;4:36.

51. Rosenstock J, Gross JL, Aguilar-Salinas C, et al. Long-term 4-year safety of saxagliptin in drug-naive and metformin-treated patients with type 2 diabetes. Diabet Med 2013;30:1472-6.

52. Yang W, Pan C, Tou C, Zhao J, Gause-Nilsson I. Efficacy and safety of saxagliptin added to metformin in Asian people with type 2 diabetes mellitus: a randomized controlled trial. Diabetes Res Clin Pract 2011;94:217-24.

53. Stenlöf K, Raz I, Neutel J, Ravichandran S, Berglind N, Chen R. Saxagliptin and metformin XR combination therapy provides glycemic control over 24 hours in patients with T2DM inadequately controlled with metformin. Curr Med Res Opin 2010;26:2355-63.

54. White J, Buchanan P, Li J, Frederich R. A randomized controlled trial of the efficacy and safety of twice-daily saxagliptin plus metformin combination therapy in patients with type 2 diabetes and inadequate glycemic control on metformin monotherapy. BMC Endocr Disord 2014;14.

55. Chacra A, Tan G, Ravichandran S, List J, Chen R, CV181040 Investigators. Safety and efficacy of saxagliptin in combination with submaximal sulphonylureas versus up-titrated sulphonylureas over 76 weeks. Diabetes Vasc Dis Res 2011;8:150-9.

56. Nowicki M, Rychlik I, Haller H, et al. Long-term treatment with the dipeptidyl peptidase-4 inhibitor saxagliptin in patients with type 2 diabetes mellitus and renal impairment: a randomised controlled 52-week efficacy and safety study. Int J Clin Pract 2011;65:1230-9.

57. Hollander P, Li J, Frederich R, Allen E, R; C, CV181013 Investigators. Safety and efficacy of saxagliptin added to thiazolidinedione over 76 weeks in patients with type 2 diabetes mellitus. Diabetes Vasc Dis Res 2011;8:125-35.

58. Barnett A, Charbonnel B, Donovan M, Fleming D, Chen R. Effect of saxagliptin as add-on therapy in patients with poorly controlled type 2 diabetes on insulin alone or insulin combined with metformin. Curr Med Res Opin 2012;28:513-23.

59. Scirica B, Bhatt D, Braunwald E, et al. Saxagliptin and cardiovascular outcomes in patients with type 2 diabetes mellitus. N Engl J Med 2013;369:317-1326.

60. Henry R, Smith S, Schwartz S, et al. Effects of saxagliptin on β-cell stimulation and insulin secretion in patients with type 2 diabetes. Diabetes Obes Metab 2011;13:850-8.

61. Pfützner A, Paz-Pacheco E, Allen E, Frederich R, Chen R, CV181039 Investigators. Initial combination therapy with saxagliptin and metformin provides sustained glycaemic control and is well tolerated for up to 76 weeks. Diabetes Obes Metab 2011;13:567-76.

62. Hermans M, Delibasi T, Farmer I, et al. Effects of saxagliptin added to sub-maximal doses of metformin compared with uptitration of metformin in type 2 diabetes: the PROMPT study. Curr Med Res Opin 2012;28:1635-45.

63. Neutel J, Zhao C, Karyekar C. Adding saxagliptin to metformin Extendederelease (XR) or uptitration of metformin XR: efficacy on daily glucose measures. Diabetes Ther 2013;4:269-83.

64. Fonseca V, Zhu T, Karyekar C, Hirshberg B. Adding saxagliptin to extended-release metformin vs. uptitrating metformin dosage. Diabetes Obes Metab 2012;14:365-71.

65. Göke B, Gallwitz B, Eriksson J, Hellqvist Å, Gause-Nilsson I. Saxagliptin vs. glipizide as add-on therapy in patients with type 2 diabetes mellitus inadequately controlled on metformin alone: long-term (52-week) extension of a 52-week randomised controlled trial. Int J Clin Pract 2013;67:307-16.

66. Schernthaner G, Durán Garcia S, Hanefeld M, et al. Efficacy and tolerability of saxagliptin compared with glimepiride in elderly patients with type 2 diabetes: a randomized, controlled study (GENERATION). Diabetes Obes Metab 2015;17:630-8.

67. Scheen A, Charpentier G, Ostgren C, Hellqvist A, Gause-Nilsson I. Efficacy and safety of saxagliptin in combination with metformin compared with sitagliptin in combination with metformin in adult patients with type 2 diabetes mellitus. Diabetes Metab Res Rev 2010;26:540-9.

68. Hage C, Brismar K, Lundman P, Norhammar A, Rydén L, Mellbin L. The DPP-4 inhibitor sitagliptin and endothelial function in patients with acute coronary syndromes and newly detected glucose perturbations: A report from the BEGAMI study. Diabetes Vasc Dis Res 2014;11:290-3.

69. Mohan V, Yang W, Son H-Y, et al. Efficacy and safety of sitagliptin in the treatment of patients with type 2 diabetes in China, India, and Korea. Diabetes Res Clin Pract 2009;83:106-6.

70. Barzilai N, Guo H, Mahoney E, et al. Efficacy and tolerability of sitagliptin monotherapy in elderly patients with type 2 diabetes: a randomized, double-blind, placebo-controlled trial. Curr Med Res Opin 2011;27:1049-58.

71. Ji L, Han P, Wang X, et al. Randomized clinical trial of the safety and efficacy of sitagliptin and metformin co-administered to Chinese patients with type 2 diabetes mellitus. J Diabetes Investig 2016;7:727-36.

72. Scott R, Wu M, Sanchez M, Stein P. Efficacy and tolerability of the dipeptidyl peptidase-4 inhibitor sitagliptin as monotherapy over 12 weeks in patients with type 2 diabetes. Int J Clin Pract 2007;61:171-80.

73. HU H, Merker L, Christiansen A, et al. Empagliflozin as add-on to metformin plus sulphonylurea in patients with type 2 diabetes. Diabetes Res Clin Pract 2015;110:82-90.

74. Aschner P, Kipnes M, Lunceford J, et al. Effect of the dipeptidyl peptidase-4 inhibitor sitagliptin as monotherapy on glycemic control in patients with type 2 diabetes. Diabetes Care 2006;29:2632-7.

75. Roden M, Weng J, Eilbracht J, et al. Empagliflozin monotherapy with sitagliptin as an active comparator in patients with type 2 diabetes: a randomised, double-blind, placebo-controlled, phase 3 trial. Lancet 2013;1:208-19.

76. Hanefeld M, Herman G, Wu M, Mickel C, Sanchez M, Stein P. Once-daily sitagliptin, a dipeptidyl peptidase-4 inhibitor, for the treatment of patients with type 2 diabetes. Curr Med Res Opin 2007;23:1329-39.

77. Wang W, Ning G, Ma J, et al. A randomized clinical trial of the safety and efficacy of sitagliptin in patients with type 2 diabetes mellitus inadequately controlled by acarbose alone. Curr Med Res Opin 2017;33:693-9.

78. Yang W, Guan Y, Shentu Y, et al. The addition of sitagliptin to ongoing metformin therapy significantly improves glycemic control in Chinese patients with type 2 diabetes. Diabetes 2012;4:227-37.

79. Raz I, Chen Y, Wu M, et al. Efficacy and safety of sitagliptin added to ongoing metformin therapy in patients with type 2 diabetes. Curr Med Res Opin 2008;24: 537-50.

80. ClinicalTrials.gov. Beta-Cell function and sitagliptin trial (BEST) (BEST). (https://clinicaltrials.gov/ct2/show/NCT00420511). Accessed November 30, 2023.

81. Bergenstal R, Forti A, Chiasson J-L, Woloschak M, Boldrin M, Balena R. Efficacy and safety of taspoglutide versus sitagliptin for type 2 diabetes mellitus (T-emerge 4 trial). Diabetes Ther 2012;3:13.

82. Gadde K, Vetter M, Iqbal N, Hardy E, P; Ö, DURATION-NEO-2 study investigators. Efficacy and safety of autoinjected exenatide once-weekly suspension versus sitagliptin or placebo with metformin in patients with type 2 diabetes: The DURATION-NEO-2 randomized clinical study. Diabetes Obes Metab 2017;19:979-88.

83. Rosenstock J, Aggarwal N, Polidori D, et al. Dose-ranging effects of canagliflozin, a sodium-glucose cotransporter 2 inhibitor, as add-on to metformin in subjects with type 2 diabetes. Diabetes Care 2012;35:1232-8.

84. Rosenstock J, Seman L, Jelaska A, et al. Efficacy and safety of empagliflozin, a sodium glucose cotransporter 2 (SGLT2) inhibitor, as add-on to metformin in type 2 diabetes with mild hyperglycaemia. Diabetes Obes Metab 2013;15:1154-60.

85. Ba J, Han P, Yuan G, et al. Randomized trial assessing the safety and efficacy of sitagliptin in Chinese patients with type 2 diabetes mellitus inadequately controlled on sulfonylurea alone or combined with metformin. J Diabetes 2017;9:667-6.

86. Dobs A, Goldstein B, Aschner P, et al. Efficacy and safety of sitagliptin added to ongoing metformin and rosiglitazone combination therapy in a randomized placebo-controlled 54-week trial in patients with type 2 diabetes. Diabetes 2013;5:68-79.

87. Fonseca V, Staels B, Morgan J, et al. Efficacy and safety of sitagliptin added to ongoing metformin and pioglitazone combination therapy in a randomized, placebo-controlled, 26-week trial in patients with type 2 diabetes. J Diabetes Complications 2013;27:177-83.

88. Rosenstock J, Brazg R, Andryuk P, Lu K, P; S, Group* ftSS. Efficacy and safety of the dipeptidyl peptidase-4 inhibitor sitagliptin added to ongoing pioglitazone therapy in patients with type 2 diabetes: a 24-week, multicenter, randomized, double-blind, placebo-controlled, parallel-group study. Clin Ther 2006;28.

89. Green J, Bethel A, Armstrong P, et al. Effect of sitagliptin on cardiovascular outcomes in type 2 diabetes. N Engl J Med 2015;373:232-42.

90. Shankar R, Bao Y, Han P, et al. Sitagliptin added to stable insulin therapy with or without metformin in Chinese patients with type 2 diabetes. J Diabetes Investig 2017;8:321-9.

91. Vilsbøll T, Rosenstock J, Yki-Järvinen H, et al. Efficacy and safety of sitagliptin when added to insulin therapy in patients with type 2 diabetes. Diabetes Obes Metab 2010;12:167-77.

92. Mathieu C, Ranetti A, Li D, et al. Randomized, double-blind, phase 3 trial of triple therapy with dapagliflozin add-on to saxagliptin plus metformin in type 2 diabetes. Diabetes Care 2015;38:2009-17.

93. Williams-Herman D, Johnson J, Teng R, et al. Efficacy and safety of sitagliptin and metformin as initial combination therapy and as monotherapy over 2 years in patients with type 2 diabetes. Diabetes Obes Metab 2010;12:442-51.

94. Amin N, Wang X, Mitchell J, Lee D, Nucci G, Rusnak J. Blood pressure-lowering effect of the sodium glucose co-transporter-2 inhibitor ertugliflozin, assessed via ambulatory blood pressure monitoring in patients with type 2 diabetes and hypertension. Diabetes Obes Metab 2015;17:805-8.

95. Aschner P, Katzeff H, Guo H, et al. Efficacy and safety of monotherapy of sitagliptin compared with metformin in patients with type 2 diabetes. Diabetes Obes Metab 2010;12:252-61.

96. Olansky L, Reasner C, Seck T, et al. The effect of initial therapy with the fixed-dose combination of sitagliptin and metformin compared with metformin monotherapy in patients with type 2 diabetes mellitus. Diabetes Obes Metab 2011;13:644-52.

97. Pérez-Monteverde A, Seck T, Xu L, et al. Efficacy and safety of sitagliptin and the fixed-dose combination of sitagliptin and metformin vs. pioglitazone in drug-naive patients with type 2 diabetes. Int J Clin Pract 2011;65:930-8.

98. Wainstein J, Katz L, Engel S, et al. Initial therapy with the fixed-dose combination of sitagliptin and metformin results in greater improvement in glycaemic control compared with pioglitazone monotherapy in patients with type 2 diabetes. Diabetes Obes Metab 2012;14:409-18.

99. Russell-Jones D, Cuddihy R, Hanefeld M, et al. Efficacy and safety of exenatide once weekly versus metformin, pioglitazone, and sitagliptin used as monotherapy in drug-naive patients with type 2 diabetes (DURATION-4): a 26-week double-blind study. Diabetes Care 2012;35:252-8.

100. Raz I, Hanefeld M, Xu L, et al. Efficacy and safety of the dipeptidyl peptidase-4 inhibitor sitagliptin as monotherapy in patients with type 2 diabetes mellitus. Diabetologia 2006;49:2564-71.

101. ClinicalTrials.gov. A study to evaluate the safety and efficacy of sitagliptin 100 mg in participants with type 2 diabetes mellitus who have inadequate glycemic control (MK-0431-229). (https://clinicaltrials.gov/ct2/show/NCT01076075). Accessed November 30, 2023.
102. Hermansen K, Kipnes M, Luo E, et al. Efficacy and safety of the dipeptidyl peptidase-4 inhibitor, sitagliptin, in patients with type 2 diabetes mellitus inadequately controlled on glimepiride alone or on glimepiride and metformin. Diabetes Obes Metab 2007;9:733-45.
103. Yoon K, Steinberg H, Teng R, et al. Efficacy and safety of initial combination therapy with sitagliptin and pioglitazone in patients with type 2 diabetes: a 54-week study. Diabetes Obes Metab 2012;14.
104. Bergenstal R, Wysham C, Macconell L, et al. Efficacy and safety of exenatide once weekly versus sitagliptin or pioglitazone as an adjunct to metformin for treatment of type 2 diabetes (DURATION-2): a randomised trial. Lancet 2010;376:431-9.
105. ClinicalTrials.gov. MK0431 and pioglitazone co-administration factorial study in patients with type 2 diabetes mellitus (0431-102 AM2). (https://clinicaltrials.gov/ct2/show/NCT00722371). Accessed November 30, 2023.
106. Chan J, Scott R, Arjona Ferreira J, et al. Safety and efficacy of sitagliptin in patients with type 2 diabetes and chronic renal insufficiency. Diabetes Obes Metab 2008;10:545-55.
107. Charbonnel B, Karasik A, Liu J, Wu M, G; M, Sitagliptin Study 020 Group. Efficacy and safety of the dipeptidyl peptidase-4 inhibitor sitagliptin added to ongoing metformin therapy in patients with type 2 diabetes inadequately controlled with metformin alone. Diabetes Care 2006;29:2638-43.

108. Ahrén B, Johnson S, Stewart M, et al. HARMONY 3: 104-week randomized, double-blind, placebo- and active-controlled trial assessing the efficacy and safety of albiglutide compared with placebo, sitagliptin, and glimepiride in patients with type 2 diabetes taking metformin. Diabetes Care 2014;37:2141-8.

109. Arjona Ferreira J, Corry D, Mogensen C, et al. Efficacy and safety of sitagliptin in patients with type 2 diabetes and ESRD receiving dialysis: a 54-week randomized trial. Am J Kideny Dis 2013;61:579-87.

110. Ferreira J, Marre M, Barzilai N, et al. Efficacy and safety of sitagliptin versus glipizide in patients with type 2 diabetes and moderate-to-severe chronic renal insufficiency. Diabetes Care 2013;36:1067-73.

111. Seck T, Nauck M, Sheng D, et al. Safety and efficacy of treatment with sitagliptin or glipizide in patients with type 2 diabetes inadequately controlled on metformin: a 2-year study. Int J Clin Pract 2010;64:562-76.

112. Arechavaleta R, Seck T, Chen Y, et al. Efficacy and safety of treatment with sitagliptin or glimepiride in patients with type 2 diabetes inadequately controlled on metformin monotherapy: a randomized, double-blind, non-inferiority trial. Diabetes Obes Metab 2011;31:160-8.

113. ClinicalTrials.gov. Study of sitagliptin treatment in patients with type 2 diabetes during ramadan (0431-263). (https://clinicaltrials.gov/ct2/show/NCT01131182). Accessed November 30, 2023.

114. Terauchi Y, Yamada Y, Ishida H, et al. Efficacy and safety of sitagliptin as compared with glimepiride in Japanese patients with type 2 diabetes mellitus aged ≥ 60 years (START-J trial). Diabetes Obes Metab 2017;19:1188-92.

115. Leiter L, Carr M, Stewart M, et al. Efficacy and safety of the once-weekly GLP-1 receptor agonist albiglutide versus sitagliptin in patients with type 2 diabetes and renal impairment: a randomized phase III study. Diabetes Care 2014;37:2723-30.
116. Pratley R, Nauck M, Bailey T, et al. One year of liraglutide treatment offers sustained and more effective glycaemic control and weight reduction compared with sitagliptin, both in combination with metformin, in patients with type 2 diabetes: a randomised, parallel-group, open-label trial. Int J Clin Pract 2011;65:397-407.
117. Zang L, Liu Y, Geng J, et al. Efficacy and safety of liraglutide versus sitagliptin, both in combination with metformin, in Chinese patients with type 2 diabetes: a 26-week, open-label, randomized, active comparator clinical trial. Diabetes Obes Metab 2016;18:803-11.
118. ClinicalTrials.gov. Treatment intensification with biphasic insulin aspart 30 in subjects with type 2 diabetes inadequately controlled on sitagliptin and metformin (SIT2MIX). (https://clinicaltrials.gov/ct2/show/NCT01519674). Accessed November 30, 2023.
119. Philis-Tsimikas A, Prato D, Satman I, et al. Effect of insulin degludec versus sitagliptin in patients with type 2 diabetes uncontrolled on oral antidiabetic agents. Diabetes Obes Metab 2013;15:760-6.
120. ClinicalTrials.gov. Study to assess the efficacy and safety of sitagliptin added to the regimen of patients with type 2 diabetes mellitus with inadequate glycemic control on metformin (0431-189). (https://clinicaltrials.gov/ct2/show/NCT00875394). Accessed November 30, 2023.
121. ClinicalTrials.gov. Safety and tolerability of vildagliptin versus placebo in patients with type 2 diabetes and moderate or severe renal insufficiency. (https://clinicaltrials.gov/ct2/show/NCT00646542). Accessed November 30, 2023.

122. ClinicalTrials.gov. Efficacy and long-term safety of vildagliptin as monotherapy in patients with type 2 diabetes. (https://clinicaltrials.gov/ct2/show/NCT00821977). Accessed November 30, 2023.

123. Scherbaum W, Schweizer A, Mari A, et al. Efficacy and tolerability of vildagliptin in drug-naive patients with type 2 diabetes and mild hyperglycaemia. Diabetes Obes Metab 2008;10:675-82.

124. Dejager S, Razac S, Foley J, Schweizer A. Vildagliptin in drug-naïve patients with type 2 diabetes: a 24-week, double-blind, randomized, placebo-controlled, multiple-dose study. Horm Metab Res 2007;39:218-38.

125. McMurray J, Ponikowski P, Bolli G. The vildagliptin in ventricular dysfunction diabetes trial (VIVIDD). (https://scholar.google.com/scholar?hl=en&as_sdt=0%2C5&q=+The+Vildagliptin+in+Ventricular+Dysfunction+Diabetes+%28VIVIDD%29+trial+NCT00894868&btnG=). Accessed November 30, 2023.

126. Pan C, Xing X, Han P, et al. Efficacy and tolerability of vildagliptin as add-on therapy to metformin in Chinese patients with type 2 diabetes mellitus. Diabetes Obes Metab 2012;14:737-44.

127. ClinicalTrials.gov. Vildagliptin 100 mg once daily vs. placebo as add-on therapy in patients with type 2 diabetes inadequately controlled with metformin. (https://www.clinicaltrialsregister.eu/ctr-search/trial/2007-000538-37/results or https://clinicaltrials.gov/ct2/show/NCT00494884). Accessed November 30, 2023.

128. Bosi E, Camisasca R, Collober C, Rochotte E, Garber A. Effects of vildagliptin on glucose control over 24 weeks in patients with type 2 diabetes inadequately controlled with metformin. Diabetes Care 2007;30:890-5.

129. Yang W, Xing X, Lv X, et al. Vildagliptin added to sulfonylurea improves glycemic control without hypoglycemia and weight gain in Chinese patients with type 2 diabetes mellitus. Diabetes 2015;7:174-14.

130. Garber A, Foley J, Banerji M, et al. Effects of vildagliptin on glucose control in patients with type 2 diabetes inadequately controlled with a sulphonylurea*. Diabetes Obes Metab 2008;10:1047-56.

131. Ferrannini E, Fonseca V, Zinman B, et al. Fifty-two-week efficacy and safety of vildagliptin vs. glimepiride in patients with type 2 diabetes mellitus inadequately controlled on metformin monotherapy. Diabetes Obes Metab 2009;11:157-66.

132. Bosi E, Dotta F, Jia Y, Goodman M. Vildagliptin plus metformin combination therapy provides superior glycaemic control to individual monotherapy in treatment-naive patients with type 2 diabetes mellitus. Diabetes Obes Metab 2009;11:506-15.

133. Schweizer A, Couturier A, Foley J, Dejager S. Comparison between vildagliptin and metformin to sustain reductions in HbA(1c) over 1 year in drug-naïve patients with type 2 diabetes. Diabet Med 2007;24:955-61.

134. Pan C, Yang W, Barona J, et al. Comparison of vildagliptin and acarbose monotherapy in patients with type 2 diabetes: a 24-week, double-blind, randomized trial. Diabet Med 2008;25:435-441.

135. Rosenstock J, Niggli M, Maldonado-Lutomirsky M. Long-term 2-year safety and efficacy of vildagliptin compared with rosiglitazone in drug-naïve patients with type 2 diabetes mellitus. Diabetes Obes Metab. Diabetes Obes Metab 2009;11:571-8.

136. Bolli G, Dotta F, Colin L, Minic B, Goodman M. Comparison of vildagliptin and pioglitazone in patients with type 2 diabetes inadequately controlled with metformin. Diabetes Obes Metab 2009;11:589-95.

137. Foley J, Sreenan S. Efficacy and safety comparison between the DPP-4 inhibitor vildagliptin and the sulfonylurea gliclazide after two years of monotherapy in drug-naıve patients with type 2 diabetes. Horm Metab Res 2009;41:905-9.

138. Ferrannini E, Fonseca V, Zinman B, et al. Fifty-two-week efficacy and safety of vildagliptin vs. glimepiride in patients with type 2 diabetes mellitus inadequately controlled on metformin monotherapy. Diabetes Obes Metab 2009;11:157-66.

139. Nauck M, Stewart M, Perkins C, et al. Efficacy and safety of once-weekly GLP-1 receptor agonist albiglutide (HARMONY 2): 52 week primary endpoint results from a randomised, placebo-controlled trial in patients with type 2 diabetes mellitus inadequately controlled with diet and exercise. Diabetologia 2016;59:266-74.

140. Hernandez A, Green J, Janmohamed S, et al. Albiglutide and cardiovascular outcomes in patients with type 2 diabetes and cardiovascular disease (Harmony Outcomes): a double-blind, randomised placebo-controlled trial. Lancet 2018;392:1519-29.

141. Reusch J, Stewart M, Perkins C, et al. Efficacy and safety of once-weekly glucagon-like peptide 1 receptor agonist albiglutide (HARMONY 1 trial): 52-week primary endpoint results from a randomized, double-blind, placebo-controlled trial in patients with type 2 diabetes mellitus not controlled on pioglitazone, with or without metformin. Diabetes Obes Metab 2014;16:1257-64.

142. Seino Y, Inagaki N, Miyahara H, et al. A randomized dose-finding study demonstrating the efficacy and tolerability of albiglutide in Japanese patients with type 2 diabetes mellitus. Curr Med Res Opin 2014;30:1095-6.

143. Home P, Shamanna P, Stewart M, et al. Efficacy and tolerability of albiglutide versus placebo or pioglitazone over 1 year in people with type 2 diabetes currently taking metformin and glimepiride: HARMONY 5. Diabetes Obes Metab 2014;17:179-87.

144. Pratley R, Nauck M, Barnett A, et al. Once-weekly albiglutide versus once-daily liraglutide in patients with type 2 diabetes inadequately controlled on oral drugs (HARMONY 7): a randomised, open-label, multicentre, non-inferiority phase 3 study. Lancet 2014;2:289-97.

145. Weissman P, Carr M, Ye J, et al. HARMONY 4: randomised clinical trial comparing once-weekly albiglutide and insulin glargine in patients with type 2 diabetes inadequately controlled with metformin with or without sulfonylurea. Diabetologia 2014;57:2475-84.

146. Rosenstock J, Fonseca V, Gross J, et al. Advancing basal insulin replacement in type 2 diabetes inadequately controlled with insulin glargine plus oral agents: a comparison of adding albiglutide, a weekly GLP-1 receptor agonist, versus thrice-daily prandial insulin lispro. Diabetes Care 2014;37:2317-25.

147. Gerstein H, Colhoun H, Dagenais G, et al. Dulaglutide and cardiovascular outcomes in type 2 diabetes (REWIND): a double-blind, randomised placebo-controlled trial. Lancet 2019;394:121-30.

148. Wysham C, Blevins T, Arakaki R, et al. Efficacy and safety of dulaglutide added onto pioglitazone and metformin versus exenatide in type 2 diabetes in a randomized controlled trial (AWARD-1). Diabetes Care 2014;37:2159-67.

149. Pozzilli P, Norwood P, Jódar E, et al. Placebo-controlled, randomized trial of the addition of once-weekly glucagon-like peptide-1 receptor agonist dulaglutide to titrated daily insulin glargine in patients with type 2 diabetes (AWARD-9). Diabetes Obes Metab 2017;19:1024-31.

150. Holman R, Bethel A, Mentz R, et al. Effects of once-weekly exenatide on cardiovascular outcomes in type 2 diabetes. N Engl J Med 2017;377:1228-39.

151. Gao Y, Yoon K, Chuang L, et al. Efficacy and safety of exenatide in patients of Asian descent with type 2 diabetes inadequately controlled with metformin or metformin and a sulphonylurea. Diabetes Res Clin Pract 2009;83:69-76.
152. Gill A, Hoogwerf B, Burger J, et al. Effect of exenatide on heart rate and blood pressure in subjects with type 2 diabetes mellitus: a double-blind, placebo-controlled, randomized pilot study. Cardiovasc Diabetol 2010;9.
153. ClinicalTrials.gov. Safety and efficacy of exenatide in patients with type 2 diabetes using a thiazolidinedione or a thiazolidinedione and metformin. (https://clinicaltrials.gov/ct2/show/NCT00099320). Accessed November 30, 2023.
154. Gallwitz B, Guzman J, Dotta F, et al. Exenatide twice daily versus glimepiride for prevention of glycaemic deterioration in patients with type 2 diabetes with metformin failure (EUREXA): an open-label, randomised controlled trial. Lancet 2012;379:2270-8.
155. Buse J, Nauck M, Forst T, et al. Exenatide once weekly versus liraglutide once daily in patients with type 2 diabetes (DURATION-6): a randomised, open-label study. Lancet 2013;381:117-24.
156. Nauck M, Duran S, Kim D, et al. A comparison of twice-daily exenatide and biphasic insulin aspart in patients with type 2 diabetes who were suboptimally controlled with sulfonylurea and metformin: a non-inferiority study. Diabetologia 2007;50:259-67.
157. Inagaki N, Atsumi Y, Oura T, Saito H, Imaoka T. Efficacy and safety profile of exenatide once weekly compared with insulin once daily in Japanese patients with type 2 diabetes treated with oral antidiabetes drug(s): results from a 26-week, randomized, open-label, parallel-group, multicenter, noninferiority study. Clin Ther 2012;34:1892-1908.

158. Marso S, Daniels G, Brown-Frandsen K, et al. Liraglutide and cardiovascular outcomes in type 2 diabetes. N Engl J Med 2016;375:311-22.

159. Seino Y, Kaneko S, Fukuda S, et al. Combination therapy with liraglutide and insulin in Japanese patients with type 2 diabetes: A 36-week, randomized, double-blind, parallel-group trial. Clinical Trial 2016;7:565-73.

160. Kaku K, Rasmussen M, Nishida T, Seino Y. Fifty-two-week, randomized, multicenter trial to compare the safety and efficacy of the novel glucagon-like peptide-1 analog liraglutide vs glibenclamide in patients with type 2 diabetes. J Diabetes Investig 2011;2:441-447.

161. Bailey T, Takács R, Tinahones F, et al. Efficacy and safety of switching from sitagliptin to liraglutide in subjects with type 2 diabetes (LIRA-SWITCH): a randomized, double-blind, double-dummy, active-controlled 26-week trial. Diabetes Obes Metab 2016;18:1191-8.

162. Lingvay I, Manghi F, García-Hernández P, et al. Effect of insulin glargine up-titration vs insulin degludec/liraglutide on glycated hemoglobin levels in patients with uncontrolled type 2 diabetes: the DUAL V randomized clinical trial. JAMA 2016;15:898-907.

163. ClinicalTrials.gov. Efficacy assessment of insulin glargine versus liraglutide after oral agents failure (EAGLE). (https://clinicaltrials.gov/ct2/show/NCT01117350). Accessed November 30, 2023.

164. Pfeffer M, Claggett B, Diaz R, et al. Lixisenatide in patients with type 2 diabetes and acute coronary syndrome. N Engl J Med 2015;373:2247-57.

165. Marso S, Bain S, Consoli A, et al. Semaglutide and cardiovascular outcomes in patients with type 2 diabetes. N Engl J Med 2016;375:1834-44.

166. Husain M, Birkenfeld A, Donsmark M, et al. Oral semaglutide and cardiovascular outcomes in patients with type 2 diabetes. N Engl J Med 2019;381:841-51.

167. Raz I, Fonseca V, Kipnes M, et al. Efficacy and safety of taspoglutide monotherapy in drug-naive type 2 diabetic patients after 24 weeks of treatment. Diabetes Care 2012;35:485-7.

168. ClinicalTrials.gov. A study of taspoglutide in patients with inadequately controlled diabetes mellitus type 2 and cardiovascular disease. (https://clinicaltrials.gov/ct2/show/NCT01018173). Accessed November 30, 2023.

169. Hollander P, Lasko B, Barnett A, et al. Effects of taspoglutide on glycemic control and body weight in obese patients with type 2 diabetes (T-emerge 7 study). Obesity 2013;21:238-47.

170. Henry R, Mudaliar S, Kanitra L, Woloschak M, Balena R, for the T-Emerge 3 Study Group. Efficacy and safety of taspoglutide in patients with type 2 diabetes inadequately controlled with metformin plus pioglitazone over 24 weeks: T-emerge 3 trial The J Clin Endocrinol Metab 2012;97:2370-9.

171. Pratley R, Urosevic D, Boldrin M, Balena R, for the T-emerge 6 Study Group. Efficacy and tolerability of taspoglutide versus pioglitazone in subjects with type 2 diabetes uncontrolled with sulphonylurea or sulphonylurea-metformin therapy: a randomized, double-blind study (T-emerge 6). Diabetes Obes Metab 2013;15:234-40.

172. Rosenstock J, Balas B, Charbonnel B, et al. The fate of taspoglutide, a weekly GLP-1 receptor agonist, versus twice-daily exenatide for type 2 diabetes. Diabetes Care 2013;36:498-504.

173. Nauck M, Horton E, Andjelkovic M, et al. Taspoglutide, a once-weekly glucagon-like peptide 1 analogue, vs. insulin glargine titrated to target in patients with Type 2 diabetes: an open-label randomized trial. Diabet Med 2013;30:109-13.

174. Inagaki N, Kondo K, Yoshinari T, Maruyama N, Susuta Y, H K. Efficacy and safety of canagliflozin in Japanese patients with type 2 diabetes: a randomized, double-blind, placebo-controlled, 12-week study. Diabetes Care 2013;15:1136-45.

175. Inagaki N, Kondo K, Yoshinari T, Takahashi N, Susuta Y, Kuki H. Efficacy and safety of canagliflozin monotherapy in Japanese patients with type 2 diabetes inadequately controlled with diet and exercise: a 24-week, randomized, double-blind, placebo-controlled, Phase III study. Expert Opin Pharmacother 2014;15:1501-15.

176. Barnett A, Mithal A, Manassie J, et al. Efficacy and safety of empagliflozin added to existing antidiabetes treatment in patients with type 2 diabetes and chronic kidney disease: a randomised, double-blind, placebo-controlled trial. Lancet 2014;2:369-84.

177. Weir M, Kline I, Xie J, Edwards R, Usiskin K. Effect of canagliflozin on serum electrolytes in patients with type 2 diabetes in relation to estimated glomerular filtration rate (eGFR). Curr Med Res Opin 2014;30:1759-68.

178. Sha S, Polidori D, Heise T, et al. Effect of the sodium glucose co-transporter 2 inhibitor canagliflozin on plasma volume in patients with type 2 diabetes mellitus. Diabetes Obes Metab 2014;16:1087-95.

179. Qiu R, Capuano G, Meininger G. Efficacy and safety of twice-daily treatment with canagliflozin, a sodium glucose co-transporter 2 inhibitor, added on to metformin monotherapy in patients with type 2 diabetes mellitus. J Clin Transl Endocrinol 2014;1:54-60.

180. Ji L, Han P, Liu Y, et al. Canagliflozin in Asian patients with type 2 diabetes on metformin alone or metformin in combination with sulphonylurea. Diabetes Obes Metab 2015;17:23-31.

181. Wilding J, Charpentier G, Hollander P, et al. Efficacy and safety of canagliflozin in patients with type 2 diabetes mellitus inadequately controlled with metformin and sulphonylurea: a randomised trial. Int J Clin Pract 2013;67:1267-82.

182. Neal B, Perkovic V, Mahaffey K W, et al. Canagliflozin and cardiovascular and renal events in type 2 diabetes. N Engl J Med 2017;377:644-57.

183. Fulcher G, Matthews D, Perkovic V, et al. Efficacy and safety of canagliflozin used in conjunction with sulfonylurea in patients with type 2 diabetes mellitus: a randomized, controlled trial. Diabetes Ther 2015;6:289-302.

184. Cai J, Delahanty L, Akapame S, Slee A, Traina S. Impact of canagliflozin treatment on health-related quality of life among people with type 2 diabetes mellitus: a pooled analysis of patient-reported outcomes from randomized controlled trials. Patient 2018;11:341-52.

185. Perkovic V, Jardine M J, Neal B, et al. Canagliflozin and renal outcomes in type 2 diabetes and nephropathy. N Engl J Med 2019;380:2295-306.

186. Bode B, K S, D S, A F, Usiskin K. Efficacy and safety of canagliflozin treatment in older subjects with type 2 diabetes mellitus: a randomized trial. Hosp Pract 2015;41:72-84.

187. Lavalle-González F, Januszewicz A, J D, et al. Efficacy and safety of canagliflozin compared with placebo and sitagliptin in patients with type 2 diabetes on background metformin monotherapy: a randomised trial. Diabetologia 2013;56:2582-92.

188. Forst T, Guthrie R, Goldenberg R, et al. Efficacy and safety of canagliflozin over 52 weeks in patients with type 2 diabetes on background metformin and pioglitazone. Diabetes Obes Metab 2014;16:467-477.

189. Rosenstock J, Chuck L, González-Ortiz M, et al. Initial combination therapy with canagliflozin plus metformin versus each component as monotherapy for drug-naïve type 2 diabetes. Diabetes Care 2016;39:353-62.

190. Cefalu W, Leiter L, Yoon K, et al. Efficacy and safety of canagliflozin versus glimepiride in patients with type 2 diabetes inadequately controlled with metformin (CANTATA-SU): 52 week results from a randomised, double-blind, phase 3 non-inferiority trial. Lancet 2013;382:941-50.

191. Schernthaner G, Gross J, Rosenstock J, et al. Canagliflozin compared with sitagliptin for patients with type 2 diabetes who do not have adequate glycemic control with metformin plus sulfonylurea: a 52-week randomized trial. Diabetes Care 2013;36:2508-15.

192. Wiviott S, Raz I, Bonaca M, et al. Dapagliflozin and cardiovascular outcomes in type 2 diabetes. N Engl J Med 2019;380:347-57.

193. Kaku K, Inoue S, Matsuoka O, et al. Efficacy and safety of dapagliflozin as a monotherapy for type 2 diabetes mellitus in Japanese patients with inadequate glycaemic control: a phase II multicentre, randomized, double-blind, placebo-controlled trial. Diabetes Obes Metab 2013;15:432-440.

194. Bailey C, Iqbal N, T'joen C, List J. Dapagliflozin monotherapy in drug-naïve patients with diabetes: a randomized-controlled trial of low-dose range. Diabetes Obes Metab 2012;14:951-959.

195. Kaku K, Kiyosue A, Inoue S, et al. Efficacy and safety of dapagliflozin monotherapy in Japanese patients with type 2 diabetes inadequately controlled by diet and exercise. Diabetes Obes Metab 2014;16:1102-10.

196. Bailey C, Morales Villegas E, Woo V, Tang W, Ptaszynska A, List J. Efficacy and safety of dapagliflozin monotherapy in people with Type 2 diabetes: a randomized double-blind placebo-controlled 102-week trial. Diabet Med 2014;35:531-41.

197. Schumm-Draeger P, Burgess L, Korányi L, Hruba V, Hamer-Maansson J, de Bruin T. Twice-daily dapagliflozin co-administered with metformin in type 2 diabetes: a 16-week randomized, placebo-controlled clinical trial. Diabetes Obes Metab 2015;17:42-51.

198. Bailey C, Gross J, Pieters A, Bastien A, List J. Effect of dapagliflozin in patients with type 2 diabetes who have inadequate glycaemic control with metformin: a randomised, double-blind, placebo-controlled trial. Lancet 2010;375:2223-33.

199. Bolinder J, Ljunggren Ö, Johansson L, et al. Dapagliflozin maintains glycaemic control while reducing weight and body fat mass over 2 years in patients with type 2 diabetes mellitus inadequately controlled on metformin. Diabetes Obes Metab 2014;16:159-69.

200. Ji L, Ma J, Li H, et al. Dapagliflozin as monotherapy in drug-naive Asian patients with type 2 diabetes mellitus: a randomized, blinded, prospective phase III study. Clin Ther 2014;36:84-100.

201. Henry R, Murray A, Marmolejo M, Hennicken D, Ptaszynska A, List J. Dapagliflozin, metformin XR, or both: initial pharmacotherapy for type 2 diabetes, a randomised controlled trial. Int J Clin Pract 2012;66:446-456.

202. Heerspink H, de Zeeuw D, Wie L, Leslie B, List J. Dapagliflozin a glucose-regulating drug with diuretic properties in subjects with type 2 diabetes. Diabetes Obes Metab 2013;15:853-62.

203. Matthaei S, Bowering K, Rohwedder K, Grohl A, Parikh S, Study 05 Group. Dapagliflozin improves glycemic control and reduces body weight as add-on therapy

to metformin plus sulfonylurea: a 24-week randomized, double-blind clinical trial. Diabetes Care 2015;38:365-72.

204. Jabbour S, Hardy E, Sugg J, Parikh S, Study 10 Group. Dapagliflozin is effective as add-on therapy to sitagliptin with or without metformin: a 24-week, multicenter, randomized, double-blind, placebo-controlled study. Diabetes Care 2014;37:740-50.

205. Wilding J, Woo V, Soler N, et al. Long-term efficacy of dapagliflozin in patients with type 2 diabetes mellitus receiving high doses of insulin: a randomized trial. Ann Fam Med 2012;152:405-15.

206. Mudaliar S, Henry R, Boden G, et al. List J.AChanges in insulin sensitivity and insulin secretion with the sodium glucose cotransporter 2 inhibitor dapagliflozin. Diabetes Technol The 2014;16:137-44.

207. ClinicalTrials.gov. Effects of dapagliflozin on insulin resistance and insulin secretion in subjects with type 2 diabetes. (https://clinicaltrials.gov/ct2/show/NCT00831779). Accessed November 30, 2023.

208. Strojek K, Yoon K, Hruba V, Elze M, Langkilde A, Parikh S. Effect of dapagliflozin in patients with type 2 diabetes who have inadequate glycaemic control with glimepiride: a randomized, 24-week, double-blind, placebo-controlled trial. Diabetes Obes Metab 2011;13:928-38.

209. Kohan D, Fioretto P, Tang W, List J. Long-term study of patients with type 2 diabetes and moderate renal impairment shows that dapagliflozin reduces weight and blood pressure but does not improve glycemic control. Kidney Int 2014;85:962-97.

210. Rosenstock J, Vico M, Wei L, Salsali A, List J. Effects of dapagliflozin, an SGLT2 inhibitor, on HbA1c, body weight, and hypoglycemia risk in patients With type 2 diabetes inadequately controlled on pioglitazone monotherapy. Diabetes Care 2012;35:1473-8.

211. Weber M, Mansfield T, Cain V, Iqbal N, Parikh S, Ptaszynska A. Blood pressure and glycaemic effects of dapagliflozin versus placebo in patients with type 2 diabetes on combination antihypertensive therapy: a randomised, double-blind, placebo-controlled, phase 3 study. Lancet 2015;4:211-20.

212. ClinicalTrials.gov. A study of BMS-512148 (dapagliflozin) in patients with type 2 diabetes and inadequately controlled hypertension on an angiotensin-converting enzyme inhibitor or angiotensin receptor blocker. (https://clinicaltrials.gov/ct2/show/NCT01137474). Accessed November 30, 2023.

213. Wilding J, Woo V, Rohwedder K, Sugg J, Parikh S, Dapagliflozin 006 Study Group. Dapagliflozin in patients with type 2 diabetes receiving high doses of insulin: efficacy and safety over 2 years. Diabetes Obes Metab 2013;16:124-36.

214. Yang W, Ma J, Li Y, et al. Dapagliflozin as add-on therapy in Asian patients with type 2 diabetes inadequately controlled on insulin with or without oral antihyperglycemic drugs: a randomized controlled trial†. Diabetes 2018;10:589-99.

215. Cefalu W, Leiter L, de Bruin T, Gause-Nilsson I, Sugg J, Parikh S. Dapagliflozin's effects on glycemia and cardiovascular risk factors in high-risk patients with type 2 diabetes: a 24-week, multicenter, randomized, double-blind, placebo-controlled study with a 28-week extension. Diabetes Care 2015;38:1218-27.

216. Leiter L, Cefalu W, de Bruin T, Gause-Nilsson I, Sugg J, Parikh S. Dapagliflozin added to usual care in individuals with type 2 diabetes mellitus with preexisting cardiovascular disease: a 24-week, multicenter, randomized, double-blind, placebo-controlled study with a 28-week extension. J Am Geriatr Soc 2014;62:1252-62.

217. List J, Woo V, Morales E, Tang W, Fiedorek F. Sodium-glucose cotransport inhibition with dapagliflozin in type 2 diabetes. Diabetes Care 2009;32:650-7.

218. Nauck M, Del Prato S, Durán-García S, et al. Durability of glycaemic efficacy over 2 years with dapagliflozin versus glipizide as add-on therapies in patients whose type 2 diabetes mellitus is inadequately controlled with metformin. Diabetes Obes Metab 2014;16:1111-20.

219. Rosenstock J, Hansen L, Zee P, et al. Dual add-on therapy in type 2 diabetes poorly controlled with metformin monotherapy: a randomized double-blind trial of saxagliptin plus dapagliflozin addition versus single addition of saxagliptin or dapagliflozin to metformin. Diabetes Care 2015;38:376-83.

220. Kadowaki T, Haneda M, Inagaki N, et al. Efficacy and safety of empagliflozin monotherapy for 52 weeks in Japanese patients with type 2 diabetes: a randomized, double-blind, parallel-group study. Adv Ther 2015;32:306-18.

221. ClinicalTrials.gov. A 16 weeks study on efficacy and safety of two doses of empagliflozin (BI 10773) (once daily versus twice daily) in patients with type 2 diabetes mellitus and preexisting metformin therapy. (https://clinicaltrials.gov/ct2/show/NCT01649297). Accessed November 30, 2023.

222. Merker L, Häring H, Christiansen A, et al. Empagliflozin as add-on to metformin in people with Type 2 diabetes. Diabet Med 2015;32:1555-67.

223. Häring H, Merker L, Seewaldt-Becker E, et al. Empagliflozin as add-on to metformin plus sulfonylurea in patients with type 2 diabetes: a 24-week, randomized, double-blind, placebo-controlled trial. Diabetes Care 2013;36:3396-404.

224. Rosenstock J, Jelaska A, Zeller C, et al. Impact of empagliflozin added on to basal insulin in type 2 diabetes inadequately controlled on basal insulin: a 78-week randomized, double-blind, placebo-controlled trial. Diabetes Obes Metab 2015;17:936-48.

225. Zinman B, Wanner C, Lachin J, et al. Empagliflozin, cardiovascular outcomes, and mortality in type 2 diabetes. N Engl J Med 2015;373:2117-28.

226. Rosenstock J, Jelaska A, Frappin G, et al. Improved glucose control with weight loss, lower insulin doses, and no increased hypoglycemia with empagliflozin added to titrated multiple daily injections of insulin in obese inadequately controlled type 2 diabetes. Diabetes Care 2014;37:1815-23.

227. Kovacs C, Seshiah V, Swallow R, et al. Empagliflozin improves glycaemic and weight control as add-on therapy to pioglitazone or pioglitazone plus metformin in patients with type 2 diabetes: a 24-week, randomized, placebo-controlled trial. Diabetes Obes Metab 2014;16:147-58.

228. Tikkanen I, Narko K, Zeller C, et al. Empagliflozin reduces blood pressure in patients with type 2 diabetes and hypertension. Diabetes Care 2015;38:420-8.

229. Ferrannini E, Berk A, Hantel S, et al. Long-term safety and efficacy of empagliflozin, sitagliptin, and metformin: an active-controlled, parallel-group, randomized, 78-week open-label extension study in patients with type 2 diabetes. Diabetes Care 2013;36:4015-21.

230. Hadjadj S, Rosenstock J, Meinicke T, Woerle H, Broedl U. Initial combination of empagliflozin and metformin in Patients with type 2 diabetes. Diabetes Care 2016;39.

231. Araki E, Tanizawa Y, Tanaka Y, et al. Long-term treatment with empagliflozin as add-on to oral antidiabetes therapy in Japanese patients with type 2 diabetes mellitus. Diabetes Obes Metab 2015;17.

232. Ridderstråle M, Andersen KR, Zeller C, et al. Comparison of empagliflozin and glimepiride as add-on to metformin in patients with type 2 diabetes: a 104-week randomised, active-controlled, double-blind, phase 3 trial. Lancet 2014;2:691-700.

233. DeFronzo R, Lewin A, Patel S, et al. Combination of empagliflozin and linagliptin as second-line therapy in subjects with type 2 diabetes inadequately controlled on metformin. Diabetes Care 2015;38:384-93.

234. Cannon C, Pratley R, Dagogo-Jack S, et al. Cardiovascular outcomes with ertugliflozin in type 2 diabetes. N Engl J Med 2020;383:1425-35.

235. Dagogo-Jack S, Liu J, Eldor R, et al. Efficacy and safety of the addition of ertugliflozin in patients with type 2 diabetes mellitus inadequately controlled with metformin and sitagliptin: the VERTIS SITA2 placebo-controlled randomized study. Diabetes Obes Metab 2018;20:530-40.

236. Miller S, Krumins T, Zhou H, et al. Ertugliflozin and sitagliptin co-initiation in patients with type 2 diabetes: the VERTIS SITA randomized study. Diabetes Ther 2018;9:253-68.

237. Wilding J, Ferrannini E, Fonseca V, Wilpshaar W, Dhanjal P, Houzer A. Efficacy and safety of ipragliflozin in patients with type 2 diabetes inadequately controlled on metformin: a dose-finding study. Diabetes Obes Metab 2013;15:403-9.

238. Kashiwagi A, Kazuta K, Goto K, Yoshida S, Ueyama E, Utsuno A. Ipragliflozin in combination with metformin for the treatment of Japanese patients with type 2 diabetes: ILLUMINATE, a randomized, double-blind, placebo-controlled study. Diabetes Obes Metab 2015;17:304-8.

239. Lu C, Min K, Chuang L, Kokubo S, Yoshida S, Cha B. Efficacy, safety, and tolerability of ipragliflozin in Asian patients with type 2 diabetes mellitus and inadequate glycemic control with metformin: Results of a phase 3 randomized, placebo-controlled, double-blind, multicenter trial. J Diabetes Investig 2016;7:366-73.
